# Supplementary material for: Worldwide epidemiology of Crimean-Congo Hemorrhagic Fever Virus in humans, ticks and other animal species, a systematic review and meta-analysis
Source: PLoS Negl Trop Dis. 2021 Apr 22;15(4):e0009299. doi: 10.1371/journal.pntd.0009299 (PMC8096040; doi:10.1371/journal.pntd.0009299)
Supplement: S1 Text — (PDF) [file pntd.0009299.s001.pdf]

S1 Text: Reference list of studies on Crimean-Congo hemorrhagic fever virus global case fatality rate estimate in humans

1. (2006) Increase in cases of Crimean-Congo haemorrhagic fever, Turkey, 2006. Euro surveillance : bulletin Europeen sur les maladies transmissibles = European communicable disease bulletin 11: E060720.060722-E060720.060722.
2. Ahmeti S, Berisha L, Halili B, Ahmeti F, von Possel R, et al. (2019) Crimean-Congo Hemorrhagic Fever, Kosovo, 2013-2016. Emerging infectious diseases 25: 321-324.
3. Bakir M, Engin A, Gozel MG, Elaldi N, Kilickap S, et al. (2012) A new perspective to determine the severity of cases with Crimean-Congo hemorrhagic fever. Journal of vector borne diseases 49: 105-110.
4. Bakir M, Engin A, Kuskucu MA, Bakir S, Gündag O, et al. (2016) Relationship of plasma cell-free DNA level with mortality and prognosis in patients with Crimean-Congo hemorrhagic fever. Journal of medical virology 88: 1152-1158.
5. Bakır M, Gözel MG, Köksal I, Aşık Z, Günal Ö, et al. (2015) Validation of a severity grading score (SGS) system for predicting the course of disease and mortality in patients with Crimean-Congo hemorrhagic fever (CCHF). European journal of clinical microbiology & infectious diseases : official publication of the European Society of Clinical Microbiology 34: 325-330.
6. Bilgin G, Ataman Hatipoglu C, Altun S, Bulut C, Kinikli S, et al. (2014) An investigation of pulmonary findings of Crimean-Congo haemorrhagic fever patients. Turkish journal of medical sciences 44: 162-167.
7. Bozkurt I, Sunbul M, Yilmaz H, Esen S, Leblebicioglu H, et al. (2016) Direct healthcare costs for patients hospitalized with Crimean-Congo haemorrhagic fever can be predicted by a clinical illness severity scoring system. Pathogens and global health 110: 9-13.
8. Çelik İ, Dursun ZB, Bozkır FK, Bahadır F, Gürbüz S (2016) Evaluation of the Crimean-Congo Hemorrhagic Fever Cases Followed and Treated in Our Clinic between 2009-2013. Br J Med Med Res.
9. Cevik MA, Erbay A, Bodur H, Gülderen E, Baştuğ A, et al. (2008) Clinical and laboratory features of Crimean-Congo hemorrhagic fever: predictors of fatality. International journal of infectious diseases : IJID : official publication of the International Society for Infectious Diseases 12: 374-379.
10. Chinikar S, Ghiasi SM, Moradi M, Goya MM, Shirzadi MR, et al. (2010) Geographical distribution and surveillance of Crimean-Congo hemorrhagic fever in Iran. Vector borne and zoonotic diseases (Larchmont, NY) 10: 705-708.
11. Dilber E, Cakir M, Acar EA, Orhan F, Yaris N, et al. (2009) Crimean-Congo haemorrhagic fever among children in north-eastern Turkey. Annals of tropical paediatrics 29: 23-28.
12. Dokuzoguz B, Celikbas AK, Gök ŞE, Baykam N, Eroglu MN, et al. (2013) Severity scoring index for Crimean-Congo hemorrhagic fever and the impact of ribavirin and corticosteroids on fatality. Clinical infectious diseases : an official publication of the Infectious Diseases Society of America 57: 1270-1274.
13. Duygu F, Kaya T, Baysan P (2012) Re-evaluation of 400 Crimean-Congo hemorrhagic fever cases in an endemic area: is ribavirin treatment suitable? Vector borne and zoonotic diseases (Larchmont, NY) 12: 812-816.
14. Duygu F, Sari T, Celik H (2018) Effects of platelet function on the haemorrhagic manifestations and mortality in Crimean-Congo haemorrhagic fever. Le infezioni in medicina 26: 341-346.
15. Eren SH, Zengin S, Büyüktuna SA, Gözel MG (2016) Clinical severity in forecasting platelet to lymphocyte ratio in Crimean-Congo hemorrhagic fever patients. Journal of medical microbiology 65: 1100-1104.

16. Erenler AK, Kulaksiz F, Ülger H, Çapraz M, Tomak L, et al. (2015) Predictors of Crimean-Congo hemorrhagic fever in the Emergency Department. European review for medical and pharmacological sciences 19: 3811-3816.
17. Erenler AK, Kulaksiz F, Ülger H, Erdem M, Koçak C, et al. (2014) Characteristics of patients admitted to the emergency department due to tick bite. Tropical doctor 44: 86-88.
18. Ertugrul B, Uyar Y, Yavas K, Turan C, Oncu S, et al. (2009) An outbreak of Crimean-Congo hemorrhagic fever in western Anatolia, Turkey. International journal of infectious diseases : IJID : official publication of the International Society for Infectious Diseases 13: e431-e436.
19. Gambhir RM, Rathod MM (2019) Prognostic Factors in CCHF - An Indian Origin Study. The Journal of the Association of Physicians of India 67: 35-37.
20. Gozalan A, Esen B, Fitzner J, Tapar FS, Ozkan AP, et al. (2007) Crimean-Congo haemorrhagic fever cases in Turkey. Scandinavian journal of infectious diseases 39: 332-336.
21. Gul S, Ozturk DB, Kisa U, Kacmaz B, Yesilyurt M (2015) Procalcitonin Level and Its Predictive Effect on Mortality in Crimean-Congo Hemorrhagic Fever Patients. Japanese journal of infectious diseases 68: 511-513.
22. Hatipoglu CA, Bulut C, Yetkin MA, Ertem GT, Erdinc FS, et al. (2010) Evaluation of clinical and laboratory predictors of fatality in patients with Crimean-Congo haemorrhagic fever in a tertiary care hospital in Turkey. Scandinavian journal of infectious diseases 42: 516-521.
23. Izadi S, Salehi M (2009) Evaluation of the efficacy of ribavirin therapy on survival of Crimean-Congo hemorrhagic fever patients: a case-control study. Japanese journal of infectious diseases 62: 11-15.
24. Karakus N, Yigit S, Duygu F, Barut S, Rustemoglu A, et al. (2019) Effects of Paraoxonase-1 variants on course of severity and mortality of Crimean-Congo hemorrhagic fever. Gene 687: 188-192.
25. Kızılgun M, Ozkaya-Parlakay A, Tezer H, Gulhan B, Yuksek SK, et al. (2013) Evaluation of Crimean-Congo hemorrhagic fever virus infection in children. Vector borne and zoonotic diseases (Larchmont, NY) 13: 804-806.
26. Leblebicioglu H, Sunbul M, Guner R, Bodur H, Bulut C, et al. (2016) Healthcare-associated Crimean-Congo haemorrhagic fever in Turkey, 2002-2014: a multicentre retrospective cross-sectional study. Clinical microbiology and infection : the official publication of the European Society of Clinical Microbiology and Infectious Diseases 22: 387.e381-387.e384.
27. Mofleh J, Ahmad Z (2012) Crimean-Congo haemorrhagic fever outbreak investigation in the Western Region of Afghanistan in 2008. Eastern Mediterranean health journal = La revue de sante de la Mediterranee orientale = al-Majallah al-sihhiyah li-sharq al-mutawassit 18: 522-526.
28. Mostafavi E, Pourhossein B, Chinikar S (2014) Clinical symptoms and laboratory findings supporting early diagnosis of Crimean-Congo hemorrhagic fever in Iran. Journal of medical virology 86: 1188-1192.
29. Mourya DT, Viswanathan R, Jadhav SK, Yadav PD, Basu A, et al. (2017) Retrospective analysis of clinical information in Crimean-Congo haemorrhagic fever patients: 2014-2015, India. The Indian journal of medical research 145: 673-678.
30. Niazi A-U-R, Jawad MJ, Amirnajad A, Durr PA, Williams DT (2019) Crimean-Congo Hemorrhagic Fever, Herat Province, Afghanistan, 2017. Emerging infectious diseases 25: 1596-1598.
31. Ozbey SB, Kader Ç, Erbay A, Ergönül Ö (2014) Early use of ribavirin is beneficial in Crimean-Congo hemorrhagic fever. Vector borne and zoonotic diseases (Larchmont, NY) 14: 300-302.
32. Sahak MN, Arifi F, Saeedzai SA (2019) Descriptive epidemiology of Crimean-Congo Hemorrhagic Fever (CCHF) in Afghanistan: Reported cases to National Surveillance System, 2016-2018. International journal of infectious diseases : IJID : official publication of the International Society for Infectious Diseases 88: 135-140.
33. Sheikh AS, Sheikh AA, Sheikh NS, Rafi US, Asif M, et al. (2005) Bi-annual surge of Crimean-Congo haemorrhagic fever (CCHF): a five-year experience. International journal of infectious

diseases : IJID : official publication of the International Society for Infectious Diseases 9: 37-42.
34. Swanepoel R, Shepherd AJ, Leman PA, Shepherd SP, McGillivray GM, et al. (1987) Epidemiologic and clinical features of Crimean-Congo hemorrhagic fever in southern Africa. The American journal of tropical medicine and hygiene 36: 120-132.
35. Tasdelen Fisgin N, Doganci L, Tanyel E, Tulek N (2010) Initial high rate of misdiagnosis in Crimean Congo haemorrhagic fever patients in an endemic region of Turkey. Epidemiology and infection 138: 139-144.
36. Tasdelen Fisgin N, Tanyel E, Doganci L, Tulek N (2009) Risk factors for fatality in patients with Crimean-Congo haemorrhagic fever. Tropical doctor 39: 158-160.
37. Tuygun N, Tanir G, Caglayik DY, Uyar Y, Korukluoglu G, et al. (2012) Pediatric cases of Crimean-Congo hemorrhagic fever in Turkey. Pediatrics international : official journal of the Japan Pediatric Society 54: 402-406.
38. Yadav PD, Gurav YK, Mistry M, Shete AM, Sarkale P, et al. (2014) Emergence of Crimean-Congo hemorrhagic fever in Amreli District of Gujarat State, India, June to July 2013. International journal of infectious diseases : IJID : official publication of the International Society for Infectious Diseases 18: 97-100.
39. Yilmaz GR, Buzgan T, Irmak H, Safran A, Uzun R, et al. (2009) The epidemiology of Crimean-Congo hemorrhagic fever in Turkey, 2002-2007. International journal of infectious diseases : IJID : official publication of the International Society for Infectious Diseases 13: 380-386.
40. Yilmaz M, Elaldi N, Bagci B, Sari I, Gümüs E, et al. (2015) Effect of tumour necrosis factor-alpha and interleukin-6 promoter polymorphisms on course of Crimean-Congo hemorrhagic fever in Turkish patients. Journal of vector borne diseases 52: 30-35.
41. Ziauddin, Ullah I, Kashif M, Iqbal N, Mahmood K (2018) Clinical characteristics of crimean congo hemaorrhagic fever: Experience at a tertiary care hospital in Khyber Pakhtunkhwa. Journal of Medical Sciences (Peshawar) 26: 282-286.
42. Belet N, Top A, Terzi O, Arslan HN, Baysal K, et al. (2014) Evaluation of children with Crimean-Congo hemorrhagic fever in the central Blacksea region. The Pediatric infectious disease journal 33: e194-e197.
43. Bokaie S, Mostafavi E, Haghdoost AA, Keyvanfar H, Gooya MM, et al. (2008) Crimean Congo hemorrhagic fever in Northeast of Iran. Journal of Animal and Veterinary Advances 7: 343-350.
44. Ergonul O, Celikbas A, Baykam N, Eren S, Dokuzoguz B (2006) Analysis of risk-factors among patients with Crimean-Congo haemorrhagic fever virus infection: severity criteria revisited. Clinical microbiology and infection : the official publication of the European Society of Clinical Microbiology and Infectious Diseases 12: 551-554.
45. Fakoorziba MR, Neghab M, Alipour H, Moemenbellah-Fard MD (2006) Tick borne Crimean-Congo haemorrhagic fever in Fars province, southern Iran: Epidemiologic characteristics and vector surveillance. Pakistan Journal of Biological Sciences 9: 2681-2684.
46. Kadanali A, Özden K, Erol S (2012) Crimean-Congo hemorrhagic fever virus infection: Clinical and laboratory observations and predictors of fatality. Turkiye Klinikleri Journal of Medical Sciences 32: 432-437.
47. Majeed B, Dicker R, Nawar A, Badri S, Noah A, et al. (2012) Morbidity and mortality of Crimean-Congo hemorrhagic fever in Iraq: cases reported to the National Surveillance System, 1990-2010. Transactions of the Royal Society of Tropical Medicine and Hygiene 106: 480-483.
