# Supplementary material for: Worldwide epidemiology of Crimean-Congo Hemorrhagic Fever Virus in humans, ticks and other animal species, a systematic review and meta-analysis
Source: PLoS Negl Trop Dis. 2021 Apr 22;15(4):e0009299. doi: 10.1371/journal.pntd.0009299 (PMC8096040; doi:10.1371/journal.pntd.0009299)
Supplement: S2 Text — (PDF) [file pntd.0009299.s002.pdf]

S2 Text: Reference list of studies on Crimean-Congo hemorrhagic fever virus global prevalence in humans

1. (2006) Increase in cases of Crimean-Congo haemorrhagic fever, Turkey, 2006. Euro surveillance : bulletin Europeen sur les maladies transmissibles = European communicable disease bulletin 11: E060720.060722-E060720.060722.
2. Alam MM, Khurshid A, Rana MS, Aamir UB, Salman M, et al. (2017) Surveillance of Crimean-Congo haemorrhagic fever in Pakistan. The Lancet Infectious diseases 17: 806-806.
3. Almasri M, Ahmed QA, Turkestani A, Memish ZA (2019) Hajj abattoirs in Makkah: risk of zoonotic infections among occupational workers. Veterinary Medicine and Science 5: 428-434.
4. Aslani D, Salehi-Vaziri M, Baniasadi V, Jalali T, Azad-Manjiri S, et al. (2017) Crimean-Congo hemorrhagic fever among children in Iran. Archives of virology 162: 721-725.
5. Bakir M, Engin A, Gozel MG, Elaldi N, Kilickap S, et al. (2012) A new perspective to determine the severity of cases with Crimean-Congo hemorrhagic fever. Journal of vector borne diseases 49: 105-110.
6. Baumann J, Knüpfer M, Ouedraogo J, Traoré BY, Heitzer A, et al. (2019) Lassa and Crimean-Congo Hemorrhagic Fever Viruses, Mali. Emerging infectious diseases 25: 999-1002.
7. Belet N, Top A, Terzi O, Arslan HN, Baysal K, et al. (2014) Evaluation of children with Crimean-Congo hemorrhagic fever in the central Blacksea region. The Pediatric infectious disease journal 33: e194-e197.
8. Bob NS, Bâ H, Fall G, Ishagh E, Diallo MY, et al. (2017) Detection of the Northeastern African Rift Valley Fever Virus Lineage During the 2015 Outbreak in Mauritania. Open forum infectious diseases 4: ofx087-ofx087.
9. Bokaie S, Mostafavi E, Haghdoost AA, Keyvanfar H, Gooya MM, et al. (2008) Crimean Congo hemorrhagic fever in Northeast of Iran. Journal of Animal and Veterinary Advances 7: 343-350.
10. Bonney JHK, Osei-Kwasi M, Adiku TK, Barnor JS, Amesiya R, et al. (2013) Hospital-based surveillance for viral hemorrhagic fevers and hepatitides in Ghana. PLoS neglected tropical diseases 7: e2435-e2435.
11. Bower H, El Karsany M, Alzain M, Gannon B, Mohamed R, et al. (2019) Detection of Crimean-Congo Haemorrhagic Fever cases in a severe undifferentiated febrile illness outbreak in the Federal Republic of Sudan: A retrospective epidemiological and diagnostic cohort study. PLoS neglected tropical diseases 13: e0007571-e0007571.
12. Bukbuk DN, Dowall SD, Lewandowski K, Bosworth A, Baba SS, et al. (2016) Serological and Virological Evidence of Crimean-Congo Haemorrhagic Fever Virus Circulation in the Human Population of Borno State, Northeastern Nigeria. PLoS neglected tropical diseases 10: e0005126-e0005126.
13. Chinikar S (2007) Crimean-Congo hemorrhagic fever infection in Iran. Crimean-Congo Hemorrhagic Fever: A Global Perspective. pp. 89-98.
14. Chinikar S, Ghiasi SM, Moradi M, Goya MM, Shirzadi MR, et al. (2010) Geographical distribution and surveillance of Crimean-Congo hemorrhagic fever in Iran. Vector borne and zoonotic diseases (Larchmont, NY) 10: 705-708.
15. Dilber E, Cakir M, Acar EA, Orhan F, Yaris N, et al. (2009) Crimean-Congo haemorrhagic fever among children in north-eastern Turkey. Annals of tropical paediatrics 29: 23-28.
16. Erenler AK, Kulaksiz F, Ülger H, Çapraz M, Tomak L, et al. (2015) Predictors of Crimean-Congo hemorrhagic fever in the Emergency Department. European review for medical and pharmacological sciences 19: 3811-3816.
17. Erenler AK, Kulaksiz F, Ülger H, Erdem M, Koçak C, et al. (2014) Characteristics of patients admitted to the emergency department due to tick bite. Tropical doctor 44: 86-88.

18. Ergönül O, Celikbaş A, Dokuzoguz B, Eren S, Baykam N, et al. (2004) Characteristics of patients with Crimean-Congo hemorrhagic fever in a recent outbreak in Turkey and impact of oral ribavirin therapy. Clinical infectious diseases : an official publication of the Infectious Diseases Society of America 39: 284-287.
19. Ertugrul B, Uyar Y, Yavas K, Turan C, Oncu S, et al. (2009) An outbreak of Crimean-Congo hemorrhagic fever in western Anatolia, Turkey. International journal of infectious diseases : IJID : official publication of the International Society for Infectious Diseases 13: e431-e436.
20. Gadia CLB, Manirakiza A, Tekpa G, Konamna X, Vickos U, et al. (2017) Identification of pathogens for differential diagnosis of fever with jaundice in the Central African Republic: a retrospective assessment, 2008-2010. BMC infectious diseases 17: 735-735.
21. Gandhi S, Dave P, Patel GC, Khatri HJ, Shah N, et al. (2011) An epidemiological investigation of a multisource outbreak of Crimean-Congo hemorrhagic fever in Gujarat. The Journal of communicable diseases 43: 161-167.
22. Gozalan A, Esen B, Fitzner J, Tapar FS, Ozkan AP, et al. (2007) Crimean-Congo haemorrhagic fever cases in Turkey. Scandinavian journal of infectious diseases 39: 332-336.
23. Gozdas HT (2019) Evaluation of Crimean-Congo hemorrhagic fever suspected cases admitted to a secondary care hospital in Kastamonu, Turkey between 2014-2017. African health sciences 19: 1433-1440.
24. Hasan Z, Atkinson B, Jamil B, Samreen A, Altaf L, et al. (2014) Short report: Diagnostic testing for hemorrhagic fevers in Pakistan: 2007-2013. The American journal of tropical medicine and hygiene 91: 1243-1246.
25. Hatipoglu CA, Bulut C, Yetkin MA, Ertem GT, Erdinc FS, et al. (2010) Evaluation of clinical and laboratory predictors of fatality in patients with Crimean-Congo haemorrhagic fever in a tertiary care hospital in Turkey. Scandinavian journal of infectious diseases 42: 516-521.
26. Hekimoğlu HC, Demirci NA (2014) Evaluation of cases with a preliminary diagnosis of Crimean-Congo hemorrhagic fever and comparison of characteristics in patients admitted to a secondary care hospital in Kastamonu, Turkey. African health sciences 14: 873-881.
27. Kadanali A, Özden K, Erol S (2012) Crimean-Congo hemorrhagic fever virus infection: Clinical and laboratory observations and predictors of fatality. Turkiye Klinikleri Journal of Medical Sciences 32: 432-437.
28. Kara SS, Kara D, Fettah A (2016) Various clinical conditions can mimic Crimean-Congo hemorrhagic fever in pediatric patients in endemic regions. Journal of infection and public health 9: 626-632.
29. Karakecili F, Cikman A, Aydin M, Binay UD, Kesik OA, et al. (2018) Evaluation of epidemiological, clinical, and laboratory characteristics and mortality rate of patients with Crimean-Congo hemorrhagic fever in the northeast region of Turkey. Journal of vector borne diseases 55: 215-221.
30. Karlberg H, Sharifi-Mood B, Mousavi-Jazi M, Dilcher M, Lindegren G, et al. (2015) Molecular and serological findings in suspected patients with Crimean-Congo hemorrhagic fever virus in Iran. Journal of medical virology 87: 686-693.
31. Khan AS, Maupin GO, Rollin PE, Noor AM, Shurie HH, et al. (1997) An outbreak of Crimean-Congo hemorrhagic fever in the United Arab Emirates, 1994-1995. The American journal of tropical medicine and hygiene 57: 519-525.
32. Khurshid A, Hassan M, Alam MM, Aamir UB, Rehman L, et al. (2015) CCHF virus variants in Pakistan and Afghanistan: Emerging diversity and epidemiology. Journal of clinical virology : the official publication of the Pan American Society for Clinical Virology 67: 25-30.
33. Kilinc C, Gückan R, Capraz M, Varol K, Zengin E, et al. (2016) Examination of the specific clinical symptoms and laboratory findings of Crimean-Congo hemorrhagic fever. Journal of vector borne diseases 53: 162-167.
34. Leblebicioglu H, Sunbul M, Guner R, Bodur H, Bulut C, et al. (2016) Healthcare-associated Crimean-Congo haemorrhagic fever in Turkey, 2002-2014: a multicentre retrospective cross-

sectional study. Clinical microbiology and infection : the official publication of the European Society of Clinical Microbiology and Infectious Diseases 22: 387.e381-387.e384.
35. Malik S, Diju IU, Naz F (2011) Crimean Congo hemorrhagic fever in Hazara division. Journal of Ayub Medical College, Abbottabad : JAMC 23: 90-92.
36. Midilli K, Gargili A, Ergonul O, Sengöz G, Ozturk R, et al. (2007) Imported Crimean-Congo hemorrhagic fever cases in Istanbul. BMC infectious diseases 7: 54-54.
37. Mostafavi E, Pourhossein B, Chinikar S (2014) Clinical symptoms and laboratory findings supporting early diagnosis of Crimean-Congo hemorrhagic fever in Iran. Journal of medical virology 86: 1188-1192.
38. Mourya DT, Viswanathan R, Jadhav SK, Yadav PD, Basu A, et al. (2017) Retrospective analysis of clinical information in Crimean-Congo haemorrhagic fever patients: 2014-2015, India. The Indian journal of medical research 145: 673-678.
39. Nabeth P, Cheikh DO, Lo B, Faye O, Vall IOM, et al. (2004) Crimean-Congo hemorrhagic fever, Mauritania. Emerging infectious diseases 10: 2143-2149.
40. Nadeem M, Ali N, Anwar M, Hussain I, Mohammad T, et al. (2003) A comparision of clinical diagnosis and serological diagnosis in an epidemic of Crimean-Congo Haemorrhagic Fever. Pakistan Journal of Medical Sciences 19: 247-251.
41. Niazi A-U-R, Jawad MJ, Amirnajad A, Durr PA, Williams DT (2019) Crimean-Congo Hemorrhagic Fever, Herat Province, Afghanistan, 2017. Emerging infectious diseases 25: 1596-1598.
42. Papa A, Bino S, Papadimitriou E, Velo E, Dhimolea M, et al. (2008) Suspected Crimean Congo Haemorrhagic Fever cases in Albania. Scandinavian journal of infectious diseases 40: 978-980.
43. Rodriguez LL, Maupin GO, Ksiazek TG, Rollin PE, Khan AS, et al. (1997) Molecular investigation of a multisource outbreak of Crimean-Congo hemorrhagic fever in the United Arab Emirates. The American journal of tropical medicine and hygiene 57: 512-518.
44. Sahak MN, Arifi F, Saeedzai SA (2019) Descriptive epidemiology of Crimean-Congo Hemorrhagic Fever (CCHF) in Afghanistan: Reported cases to National Surveillance System, 2016-2018. International journal of infectious diseases : IJID : official publication of the International Society for Infectious Diseases 88: 135-140.
45. Schwarz TF, Jäger G, Gilch S, Pauli C, Eisenhut M, et al. (1996) Travel-related vector-borne virus infections in Germany. Archives of virology Supplementum 11: 57-65.
46. Schwarz TF, Nsanze H, Longson M, Nitschko H, Gilch S, et al. (1996) Polymerase chain reaction for diagnosis and identification of distinct variants of Crimean-Congo hemorrhagic fever virus in the United Arab Emirates. The American journal of tropical medicine and hygiene 55: 190-196.
47. Sharifi-Mood B, Mardani M, Keshtkar-Jahromi M, Rahnavardi M, Hatami H, et al. (2008) Clinical and epidemiologic features of Crimean-Congo hemorrhagic fever among children and adolescents from southeastern Iran. The Pediatric infectious disease journal 27: 561-563.
48. Sharifi-Mood B, Metanat M, Ghorbani-Vaghei A, Fayyaz-Jahani F, Akrami E (2009) The outcome of patients with Crimean-Congo hemorrhagic fever in Zahedan, southeast of Iran: a comparative study. Archives of Iranian medicine 12: 151-153.
49. Sheikh AS, Sheikh AA, Sheikh NS, Rafi US, Asif M, et al. (2005) Bi-annual surge of Crimean-Congo haemorrhagic fever (CCHF): a five-year experience. International journal of infectious diseases : IJID : official publication of the International Society for Infectious Diseases 9: 37-42.
50. Sow A, Loucoubar C, Diallo D, Faye O, Ndiaye Y, et al. (2016) Concurrent malaria and arbovirus infections in Kedougou, southeastern Senegal. Malaria journal 15: 47-47.
51. Sunbul M, Leblebicioglu H, Fletcher TE, Elaldi N, Ozkurt Z, et al. (2015) Crimean-Congo haemorrhagic fever and secondary bacteraemia in Turkey. The Journal of infection 71: 597-599.
52. Swanepoel R, Shepherd AJ, Leman PA, Shepherd SP (1985) Investigations following initial recognition of Crimean-Congo haemorrhagic fever in South Africa and the diagnosis of 2

further cases. South African medical journal = Suid-Afrikaanse tydskrif vir geneeskunde 68: 638-641.
53. Tanyel E, Sunbul M, Fletcher TE, Leblebicioglu H (2016) Aetiology of PCR negative suspected Crimean-Congo hemorrhagic fever cases in an endemic area. Pathogens and global health 110: 173-177.
54. Thomas S, Thomson G, Dowall S, Bruce C, Cook N, et al. (2012) Review of Crimean Congo hemorrhagic fever infection in Kosova in 2008 and 2009: prolonged viremias and virus detected in urine by PCR. Vector borne and zoonotic diseases (Larchmont, NY) 12: 800-804.
55. Tumturk A (2019) Crimean-Congo haemorrhagic fever in a middle Anatolian city: five years of experience. Tropical Doctor.
56. Tuygun N, Tanir G, Caglayik DY, Uyar Y, Korukluoglu G, et al. (2012) Pediatric cases of Crimean-Congo hemorrhagic fever in Turkey. Pediatrics international : official journal of the Japan Pediatric Society 54: 402-406.
57. Wasfi F, Dowall S, Ghabbari T, Bosworth A, Chakroun M, et al. (2016) Sero-epidemiological survey of Crimean-Congo hemorrhagic fever virus in Tunisia. Parasite (Paris, France) 23: 10-10.
58. Yadav PD, Gurav YK, Mistry M, Shete AM, Sarkale P, et al. (2014) Emergence of Crimean-Congo hemorrhagic fever in Amreli District of Gujarat State, India, June to July 2013. International journal of infectious diseases : IJID : official publication of the International Society for Infectious Diseases 18: 97-100.
59. Yaqub T, Shabbir MZ, Mukhtar N, Tahir Z, Abbas T, et al. (2017) Detection of selected arboviral infections in patients with history of persistent fever in Pakistan. Acta tropica 176: 34-38.
60. Yashina L, Petrova I, Seregin S, Vyshemirskii O, Lvov D, et al. (2003) Genetic variability of Crimean-Congo haemorrhagic fever virus in Russia and Central Asia. The Journal of general virology 84: 1199-1206.
61. Zhang Y, Ye F, Xia LX, Zhu LW, Kamara IL, et al. (2019) Next-generation Sequencing Study of Pathogens in Serum from Patients with Febrile Jaundice in Sierra Leone. Biomedical and environmental sciences : BES 32: 363-370.
62. Ziauddin, Ullah I, Kashif M, Iqbal N, Mahmood K (2018) Clinical characteristics of crimean congo hemaorrhagic fever: Experience at a tertiary care hospital in Khyber Pakhtunkhwa. Journal of Medical Sciences (Peshawar) 26: 282-286.
63. Abdiyeva K, Turebekov N, Dmitrovsky A, Tukhanova N, Shin A, et al. (2019) Seroepidemiological and molecular investigations of infections with Crimean-Congo haemorrhagic fever virus in Kazakhstan. International journal of infectious diseases : IJID : official publication of the International Society for Infectious Diseases 78: 121-127.
64. Ahmed A, Elduma A, Magboul B, Higazi T, Ali Y (2019) The First Outbreak of Dengue Fever in Greater Darfur, Western Sudan. Tropical medicine and infectious disease 4: 43.
65. Akuffo R, Brandful JAM, Zayed A, Adjei A, Watany N, et al. (2016) Crimean-Congo hemorrhagic fever virus in livestock ticks and animal handler seroprevalence at an abattoir in Ghana. BMC infectious diseases 16: 324-324.
66. Al Adhamy SB, E.A.M D, Saadallah (1992) Sero-epidemiological study of crimean-congo hemorrhagic fever [CCHF] during 36 months. Iraqi Med J 40-42: 232-235.
67. Alam MM, Khurshid A, Sharif S, Shaukat S, Rana MS, et al. (2013) Genetic analysis and epidemiology of Crimean Congo Hemorrhagic fever viruses in Baluchistan province of Pakistan. BMC infectious diseases 13: 201-201.
68. Al-Nakib W, Lloyd G, El-Mekki A, Platt G, Beeson A, et al. (1984) Preliminary report on arbovirus-antibody prevalence among patients in Kuwait: evidence of Congo/Crimean virus infection. Transactions of the Royal Society of Tropical Medicine and Hygiene 78: 474-476.
69. Altaf A, Luby S, Ahmed AJ, Zaidi N, Khan AJ, et al. (1998) Outbreak of Crimean-Congo haemorrhagic fever in Quetta, Pakistan: contact tracing and risk assessment. Tropical medicine & international health : TM & IH 3: 878-882.

70. Andriamandimby SF, Marianneau P, Rafisandratantsoa JT, Rollin PE, Heraud JM, et al. (2011) Crimean-Congo hemorrhagic fever serosurvey in at-risk professionals, Madagascar, 2008 and 2009. Journal of Clinical Virology 52: 370-372.
71. Antoniadis A, Casals J (1982) Serological evidence of human infection with Congo-Crimean hemorrhagic fever virus in Greece. The American journal of tropical medicine and hygiene 31: 1066-1067.
72. Athar MN, Khalid MA, Ahmad AM, Bashir N, Baqai HZ, et al. (2005) Crimean-Congo hemorrhagic fever outbreak in Rawalpindi, Pakistan, February 2002: contact tracing and risk assessment. The American journal of tropical medicine and hygiene 72: 471-473.
73. Bayram Y, Parlak M, Özkaçmaz A, Çıkman A, Güdücüoğlu H, et al. (2017) Seroprevalence of Crimean-Congo Hemorrhagic Fever in Turkey's Van Province. Japanese journal of infectious diseases 70: 65-68.
74. Blackburn NK, Searle L, Taylor P (1982) Viral haemorrhagic fever antibodies in Zimbabwe schoolchildren. Transactions of the Royal Society of Tropical Medicine and Hygiene 76: 803-805.
75. Bodur H, Akinci E, Ascioglu S, Öngürü P, Uyar Y (2012) Subclinical infections with Crimean-Congo hemorrhagic fever virus, Turkey. Emerging infectious diseases 18: 640-642.
76. Botros BA, Watts DM, Soliman AK, Salib AW, Moussa MI, et al. (1989) Serological evidence of dengue fever among refugees, Hargeysa, Somalia. Journal of medical virology 29: 79-81.
77. Bryan JP, Iqbal M, Ksiazek TG, Ahmed A, Duncan JF, et al. (1996) Prevalence of sand fly fever, West Nile, Crimean-Congo hemorrhagic fever, and leptospirosis antibodies in Pakistani military personnel. Military medicine 161: 149-153.
78. Bukbuk DN, Fukushi S, Tani H, Yoshikawa T, Taniguchi S, et al. (2014) Development and validation of serological assays for viral hemorrhagic fevers and determination of the prevalence of Rift Valley fever in Borno State, Nigeria. Transactions of the Royal Society of Tropical Medicine and Hygiene 108: 768-773.
79. Chantal J, Bessière MH, Le Guenno B, Magnaval JF, Dorchies P (1996) Serologic screening of certain zoonoses in the abattoir personnel in Djibouti. Bulletin de la Societe de pathologie exotique (1990) 89: 353-357.
80. Chapman LE, Wilson ML, Hall DB, LeGuenno B, Dykstra EA, et al. (1991) Risk factors for Crimean-Congo hemorrhagic fever in rural northern Senegal. The Journal of infectious diseases 164: 686-692.
81. Chinikar S, Ghiasi SM, Naddaf S, Piazak N, Moradi M, et al. (2012) Serological evaluation of Crimean-Congo hemorrhagic fever in humans with high-risk professions living in enzootic regions of Isfahan province of Iran and genetic analysis of circulating strains. Vector borne and zoonotic diseases (Larchmont, NY) 12: 733-738.
82. Chinikar S, Goya MM, Shirzadi MR, Ghiasi SM, Mirahmadi R, et al. (2008) Surveillance and laboratory detection system of Crimean-Congo haemorrhagic fever in Iran. Transboundary and emerging diseases 55: 200-204.
83. Chinikar S, Moghadam AH, Parizadeh SJ, Moradi M, Bayat N, et al. (2012) Seroepidemiology of crimean congo hemorrhagic Fever in slaughterhouse workers in north eastern iran. Iranian journal of public health 41: 72-77.
84. Christova I, Gladnishka T, Taseva E, Kalvatchev N, Tsergouli K, et al. (2013) Seroprevalence of Crimean-Congo hemorrhagic fever virus, Bulgaria. Emerging infectious diseases 19: 177-179.
85. Christova I, Panayotova E, Trifonova I, Taseva E, Hristova T, et al. (2017) Country-wide seroprevalence studies on Crimean-Congo hemorrhagic fever and hantavirus infections in general population of Bulgaria. Journal of medical virology 89: 1720-1725.
86. Christova I, Younan R, Taseva E, Gladnishka T, Trifonova I, et al. (2013) Hemorrhagic fever with renal syndrome and Crimean-Congo hemorrhagic fever as causes of acute undifferentiated febrile illness in Bulgaria. Vector borne and zoonotic diseases (Larchmont, NY) 13: 188-192.

87. Cikman A, Aydin M, Gulhan B, Karakecili F, Kesik OA, et al. (2016) Seroprevalence of Crimean-Congo Hemorrhagic Fever Virus in Erzincan Province, Turkey, Relationship with Geographic Features and Risk Factors. Vector borne and zoonotic diseases (Larchmont, NY) 16: 199-204.
88. Clements TL, Rossi CA, Irish AK, Kibuuka H, Eller LA, et al. (2019) Chikungunya and O'nyong-nyong Viruses in Uganda: Implications for Diagnostics. Open forum infectious diseases 6: ofz001-ofz001.
89. Darwish MA, Hoogstraal H, Roberts TJ, Ghazi R, Amer T (1983) A sero-epidemiological survey for Bunyaviridae and certain other arboviruses in Pakistan. Transactions of the Royal Society of Tropical Medicine and Hygiene 77: 446-450.
90. David-West TS, Cooke AR, David-West AS (1974) Seroepidemiology of Congo virus (related to the virus of Crimean haemorrhagic fever) in Nigeria. Bulletin of the World Health Organization 51: 543-546.
91. el-Azazy OM, Scrimgeour EM (1997) Crimean-Congo haemorrhagic fever virus infection in the western province of Saudi Arabia. Transactions of the Royal Society of Tropical Medicine and Hygiene 91: 275-278.
92. Enkhtsetseg A, Davadoorj R, Fernandez S, Mongkolsirichaikul D, Altantuul D, et al. (2016) Seroconversion to Causes of Febrile Illness in Mongolian Peacekeepers Deployed to South Sudan. The American journal of tropical medicine and hygiene 95: 1469-1471.
93. Ergonul O, Zeller H, Celikbas A, Dokuzoguz B (2007) The lack of Crimean-Congo hemorrhagic fever virus antibodies in healthcare workers in an endemic region. International journal of infectious diseases : IJID : official publication of the International Society for Infectious Diseases 11: 48-51.
94. Ergönül O, Zeller H, Kiliç S, Kutlu S, Kutlu M, et al. (2006) Zoonotic infections among veterinarians in Turkey: Crimean-Congo hemorrhagic fever and beyond. International journal of infectious diseases : IJID : official publication of the International Society for Infectious Diseases 10: 465-469.
95. Ertugrul B, Kirdar S, Ersoy OS, Ture M, Erol N, et al. (2012) The seroprevalence of Crimean-Congo haemorrhagic fever among inhabitants living in the endemic regions of Western Anatolia. Scandinavian journal of infectious diseases 44: 276-281.
96. Fajs L, Humolli I, Saksida A, Knap N, Jelovšek M, et al. (2014) Prevalence of Crimean-Congo hemorrhagic fever virus in healthy population, livestock and ticks in Kosovo. PloS one 9: e110982-e110982.
97. Fakoorziba MR, Neghab M, Alipour H, Moemenbellah-Fard MD (2006) Tick borne Crimean-Congo haemorrhagic fever in Fars province, southern Iran: Epidemiologic characteristics and vector surveillance. Pakistan Journal of Biological Sciences 9: 2681-2684.
98. Filipe AR, Calisher CH, Lazuick J (1985) Antibodies to Congo-Crimean haemorrhagic fever, Dhori, Thogoto and Bhanja viruses in southern Portugal. Acta virologica 29: 324-328.
99. Fisher-Hoch SP, McCormick JB, Swanepoel R, Van Middlekoop A, Harvey S, et al. (1992) Risk of human infections with Crimean-Congo hemorrhagic fever virus in a South African rural community. The American journal of tropical medicine and hygiene 47: 337-345.
100. Gargili A, Midilli K, Ergonul O, Ergin S, Alp HG, et al. (2011) Crimean-Congo hemorrhagic fever in European part of Turkey: genetic analysis of the virus strains from ticks and a seroepidemiological study in humans. Vector borne and zoonotic diseases (Larchmont, NY) 11: 747-752.
101. Gazi H, Özkütük N, Ecemis Ö, Atasoylu G, Köroglu G, et al. (2016) Seroprevalence of West Nile virus, Crimean-Congo hemorrhagic fever virus, Francisella tularensis and Borrelia burgdorferi in rural population of Manisa, western Turkey. Journal of vector borne diseases 53: 112-117.
102. Gergova I, Kamarinchev B (2014) Seroprevalence of Crimean-Congo hemorrhagic fever in southeastern Bulgaria. Japanese journal of infectious diseases 67: 397-398.
103. Gonzalez JP, Josse R, Johnson ED, Merlin M, Georges AJ, et al. (1989) Antibody prevalence against haemorrhagic fever viruses in randomized representative Central African populations. Research in virology 140: 319-331.

104. Gonzalez JP, LeGuenno B, Guillaud M, Wilson ML (1990) A fatal case of Crimean-Congo haemorrhagic fever in Mauritania: virological and serological evidence suggesting epidemic transmission. Transactions of the Royal Society of Tropical Medicine and Hygiene 84: 573-576.
105. Gozel MG, Bakir M, Oztop AY, Engin A, Dokmetas I, et al. (2014) Investigation of Crimean-Congo hemorrhagic fever virus transmission from patients to relatives: a prospective contact tracing study. The American journal of tropical medicine and hygiene 90: 160-162.
106. Gozel MG, Dokmetas I, Oztop AY, Engin A, Elaldi N, et al. (2013) Recommended precaution procedures protect healthcare workers from Crimean-Congo hemorrhagic fever virus. International journal of infectious diseases : IJID : official publication of the International Society for Infectious Diseases 17: e1046-e1050.
107. Greiner AL, Mamuchishvili N, Kakutia N, Stauffer K, Geleishvili M, et al. (2016) Crimean-Congo Hemorrhagic Fever Knowledge, Attitudes, Practices, Risk Factors, and Seroprevalence in Rural Georgian Villages with Known Transmission in 2014. PloS one 11: e0158049-e0158049.
108. Gunes T, Engin A, Poyraz O, Elaldi N, Kaya S, et al. (2009) Crimean-Congo hemorrhagic fever virus in high-risk population, Turkey. Emerging infectious diseases 15: 461-464.
109. Hassanein KM, el-Azazy OM, Yousef HM (1997) Detection of Crimean-Congo haemorrhagic fever virus antibodies in humans and imported livestock in Saudi Arabia. Transactions of the Royal Society of Tropical Medicine and Hygiene 91: 536-537.
110. Hassaniazad M, Bojdi A, Chinikar S, Bazaz SMM, Abedi F (2016) Preventive measures for Crimean-Congo hemorrhagic fever in healthcare workers; how high is the chance of transmission? Acta Medica Mediterranea 32: 2017-1024.
111. Hatami H, Qaderi S, Omid AM (2019) Investigation of Crimean-Congo hemorrhagic Fever in Patients Admitted in Antani Hospital, Kabul, Afghanistan, 2017-2018. International journal of preventive medicine 10: 117-117.
112. Head JR, Bumburidi Y, Mirzabekova G, Rakhimov K, Dzhumankulov M, et al. (2020) Risk Factors for and Seroprevalence of Tickborne Zoonotic Diseases among Livestock Owners, Kazakhstan. Emerging infectious diseases 26: 70-80.
113. Holakouie KN, Sh I, S C, A N (2004) Seroprevalence, incidence and risk factors of Crimean-Congo hemorrhagic fever in sistan-va-Baluchestan province, Iran. Iran J Public Health 33: 1-7.
114. Horváth LB (1976) Precipitating antibodies to Crimean haemorrhagic fever virus in human sera collected in Hungary. Acta microbiologica Academiae Scientiarum Hungaricae 23: 331-335.
115. Hosseini-Vasoukolaei N, Chinikar S, Telmadarraiy Z, Faghihi F, Hosseini-Vasoukolaei M (2016) Serological and molecular epidemiology of crimean-congo hemorrhagic fever in Ghaemshahr county in Mazandaran province; Iran. Tropical Biomedicine 33: 807-813.
116. Izadi S, Holakouie-Naieni K, Majdzadeh SR, Chinikar S, Nadim A, et al. (2006) Seroprevalence of Crimean-Congo hemorrhagic fever in Sistan-va-Baluchestan province of Iran. Japanese journal of infectious diseases 59: 326-328.
117. Izadi S, Salehi M, Holakouie-Naieni K, Chinikar S (2008) The risk of transmission of Crimean-Congo hemorrhagic fever virus from human cases to first-degree relatives. Japanese journal of infectious diseases 61: 494-496.
118. Johnson BK, Ocheng D, Gichogo A, Okiro M, Libondo D, et al. (1983) Antibodies against haemorrhagic fever viruses in Kenya populations. Transactions of the Royal Society of Tropical Medicine and Hygiene 77: 731-733.
119. Johnson BK, Ocheng D, Gitau LG, Gichogo A, Tukei PM, et al. (1983) Viral haemorrhagic fever surveillance in Kenya, 1980-1981. Tropical and geographical medicine 35: 43-47.
120. Johnson ED, Gonzalez JP, Georges A (1993) Haemorrhagic fever virus activity in equatorial Africa: distribution and prevalence of filovirus reactive antibody in the Central African Republic. Transactions of the Royal Society of Tropical Medicine and Hygiene 87: 530-535.
121. Kalvatchev N, Christova L, Pishmisheva M, Marinova M, Jeliazkova S, et al. (2010) Diagnostic capacity of CFA and ELISA methods for detection of antibodies against Crimean-Congo

haemorrhagic fever virus in patients serum. Problems of Infectious and Parasitic Diseases 38: 40-42.
122. Koksal I, Yilmaz G, Aksoy F, Erensoy S, Aydin H (2014) The seroprevalance of Crimean-Congo haemorrhagic fever in people living in the same environment with Crimean-Congo haemorrhagic fever patients in an endemic region in Turkey. Epidemiology and infection 142: 239-245.
123. Kuchuloria T, Imnadze P, Chokheli M, Tsertsvadze T, Endeladze M, et al. (2014) Viral hemorrhagic fever cases in the country of Georgia: Acute Febrile Illness Surveillance Study results. The American journal of tropical medicine and hygiene 91: 246-248.
124. Lani R, Mohd Rahim NF, Hassan H, Yaghoobi R, Chang LY, et al. (2015) First report on the seroprevalence of the Crimean-Congo haemorrhagic fever virus, a tick-borne virus, in Malaysia's Orang Asli population. European review for medical and pharmacological sciences 19: 461-466.
125. Lepers JP, Billon C, Pesce JL, Rollin PE, De Saint-Martin J (1988) Sero-epidemiological study in Mauritania (1985-1986): incidence of treponematosis, hepatitis B virus, HIV virus and viral hemorrhagic fevers. Bulletin de la Societe de pathologie exotique et de ses filiales 81: 24-31.
126. Lwande OW, Irura Z, Tigoi C, Chepkorir E, Orindi B, et al. (2012) Seroprevalence of Crimean Congo hemorrhagic fever virus in Ijara District, Kenya. Vector borne and zoonotic diseases (Larchmont, NY) 12: 727-732.
127. Magnaval JF, Tolou H, Gibert M, Innokentiev V, Laborde M, et al. (2011) Seroepidemiology of nine zoonoses in Viljujsk, Republic of Sakha (Northeastern Siberia, Russian Federation). Vector-Borne and Zoonotic Diseases 11: 157-160.
128. Majeed B, Dicker R, Nawar A, Badri S, Noah A, et al. (2012) Morbidity and mortality of Crimean-Congo hemorrhagic fever in Iraq: cases reported to the National Surveillance System, 1990-2010. Transactions of the Royal Society of Tropical Medicine and Hygiene 106: 480-483.
129. Maltezou HC, Maltezos E, Papa A (2009) Contact tracing and serosurvey among healthcare workers exposed to Crimean-Congo haemorrhagic fever in Greece. Scandinavian journal of infectious diseases 41: 877-880.
130. Mardani M, Rahnavardi M, Rajaeinejad M, Naini KH, Chinikar S, et al. (2007) Crimean-Congo hemorrhagic fever among health care workers in Iran: a seroprevalence study in two endemic regions. The American journal of tropical medicine and hygiene 76: 443-445.
131. Mathiot CC, Fontenille D, Georges AJ, Coulanges P (1989) Antibodies to haemorrhagic fever viruses in Madagascar populations. Transactions of the Royal Society of Tropical Medicine and Hygiene 83: 407-409.
132. McCarthy MC, Haberberger RL, Salib AW, Soliman BA, El-Tigani A, et al. (1996) Evaluation of arthropod-borne viruses and other infectious disease pathogens as the causes of febrile illnesses in the Khartoum Province of Sudan. Journal of medical virology 48: 141-146.
133. Memish ZA, Albarrak A, Almazroa MA, Al-Omar I, Alhakeem R, et al. (2011) Seroprevalence of Alkhurma and other hemorrhagic fever viruses, Saudi Arabia. Emerging infectious diseases 17: 2316-2318.
134. Midilli K, Gargili A, Ergonul O, Elevli M, Ergin S, et al. (2009) The first clinical case due to AP92 like strain of Crimean-Congo Hemorrhagic Fever virus and a field survey. BMC infectious diseases 9: 90-90.
135. Mohd Shukri M, Ling Kho K, Ghane Kisomi M, Lani R, Marlina S, et al. (2015) Seroprevalence report on tick-borne encephalitis virus and Crimean-Congo hemorrhagic fever virus among Malaysian's farm workers. BMC public health 15: 704-704.
136. Mostafavi E, Pourhossein B, Esmaeili S, Bagheri Amiri F, Khakifirouz S, et al. (2017) Seroepidemiology and risk factors of Crimean-Congo Hemorrhagic Fever among butchers and slaughterhouse workers in southeastern Iran. International journal of infectious diseases : IJID : official publication of the International Society for Infectious Diseases 64: 85-89.

137. Mourya DT, Yadav PD, Gurav YK, Pardeshi PG, Shete AM, et al. (2019) Crimean Congo hemorrhagic fever serosurvey in humans for identifying high-risk populations and high-risk areas in the endemic state of Gujarat, India. BMC infectious diseases 19: 104-104.
138. Mourya DT, Yadav PD, Shete AM, Gurav YK, Raut CG, et al. (2012) Detection, isolation and confirmation of Crimean-Congo hemorrhagic fever virus in human, ticks and animals in Ahmadabad, India, 2010-2011. PLoS neglected tropical diseases 6: e1653-e1653.
139. Muianga AF, Watson R, Varghese A, Chongo IS, Ali S, et al. (2017) First serological evidence of Crimean-Congo haemorrhagic fever in febrile patients in Mozambique. International journal of infectious diseases : IJID : official publication of the International Society for Infectious Diseases 62: 119-123.
140. Mustafa ML, Ayazi E, Mohareb E, Yingst S, Zayed A, et al. (2011) Crimean-Congo hemorrhagic fever, Afghanistan, 2009. Emerging infectious diseases 17: 1940-1941.
141. Newman ENC, Johnstone P, Bridge H, Wright D, Jameson L, et al. (2014) Seroconversion for infectious pathogens among UK military personnel deployed to Afghanistan, 2008-2011. Emerging infectious diseases 20: 2015-2022.
142. Nur YA, Groen J, Heuvelmans H, Tuynman W, Copra C, et al. (1999) An outbreak of West Nile fever among migrants in Kisangani, Democratic Republic of Congo. The American journal of tropical medicine and hygiene 61: 885-888.
143. O'Hearn AE, Voorhees MA, Fetterer DP, Wauquier N, Coomber MR, et al. (2016) Serosurveillance of viral pathogens circulating in West Africa. Virology journal 13: 163-163.
144. Ozkurt Z, Kiki I, Erol S, Erdem F, Yilmaz N, et al. (2006) Crimean-Congo hemorrhagic fever in Eastern Turkey: clinical features, risk factors and efficacy of ribavirin therapy. The Journal of infection 52: 207-215.
145. Palomar AM, Portillo A, Santibáñez S, García-Álvarez L, Muñoz-Sanz A, et al. (2017) Molecular (ticks) and serological (humans) study of Crimean-Congo hemorrhagic fever virus in the Iberian Peninsula, 2013-2015. Enfermedades infecciosas y microbiologia clinica 35: 344-347.
146. Papa A, Sidira P, Kallia S, Ntouska M, Zotos N, et al. (2013) Factors associated with IgG positivity to Crimean-Congo hemorrhagic fever virus in the area with the highest seroprevalence in Greece. Ticks and tick-borne diseases 4: 417-420.
147. Papa A, Sidira P, Tsatsaris A (2016) Spatial cluster analysis of Crimean-Congo hemorrhagic fever virus seroprevalence in humans, Greece. Parasite epidemiology and control 1: 211-218.
148. Rahden P, Adam A, Mika A, Jassoy C (2019) Elevated Human Crimean-Congo Hemorrhagic Fever Virus Seroprevalence in Khashm el Girba, Eastern Sudan. The American journal of tropical medicine and hygiene 100: 1549-1551.
149. Rodhain F, Gonzalez JP, Mercier E, Helynck B, Larouze B, et al. (1989) Arbovirus infections and viral haemorrhagic fevers in Uganda: a serological survey in Karamoja district, 1984. Transactions of the Royal Society of Tropical Medicine and Hygiene 83: 851-854.
150. Sadeuh-Mba SA, Yonga Wansi GM, Demanou M, Gessain A, Njouom R (2018) Serological evidence of rift valley fever Phlebovirus and Crimean-Congo hemorrhagic fever orthonairovirus infections among pygmies in the east region of Cameroon. Virology journal 15: 63-63.
151. Safronetz D, Sacko M, Sogoba N, Rosenke K, Martellaro C, et al. (2016) Vectorborne Infections, Mali. Emerging infectious diseases 22: 340-342.
152. Saidi S, Casals J, Faghih MA (1975) Crimean hemorrhagic fever-Congo (CHF-C) virus antibodies in man, and in domestic and small mammals, in Iran. The American journal of tropical medicine and hygiene 24: 353-357.
153. Saluzzo JF, Digoutte JP, Camicas JL, Chauvancy G (1985) Crimean-Congo haemorrhagic fever and Rift Valley fever in south-eastern Mauritania. Lancet (London, England) 1: 116-116.
154. Sargianou M, Panos G, Tsatsaris A, Gogos C, Papa A (2013) Crimean-Congo hemorrhagic fever: seroprevalence and risk factors among humans in Achaia, western Greece. International journal of infectious diseases : IJID : official publication of the International Society for Infectious Diseases 17: e1160-e1165.

155. Schoepp RJ, Rossi CA, Khan SH, Goba A, Fair JN (2014) Undiagnosed acute viral febrile illnesses, Sierra Leone. Emerging infectious diseases 20: 1176-1182.
156. Schwarz TF, Nitschko H, Jäger G, Nsanze H, Longson M, et al. (1995) Crimean-Congo haemorrhagic fever in Oman. Lancet (London, England) 346: 1230-1230.
157. Shahbazi N, Firouz SK, Karimi M, Mostafavi E (2019) Seroepidemiological survey of Crimean-Congo haemorrhagic fever among high-risk groups in the west of Iran. Journal of vector borne diseases 56: 174-177.
158. Shahhosseini N, Azari-Garmjan GA, Rezaiyan MK, Haeri A, Nowotny N, et al. (2018) Factors affecting transmission of crimean - Congo hemorrhagic fever among slaughterhouse employees: A Serosurvey in Mashhad, Iran. Jundishapur Journal of Microbiology 11.
159. Sidira P, Maltezou HC, Haidich AB, Papa A (2012) Seroepidemiological study of Crimean-Congo haemorrhagic fever in Greece, 2009-2010. Clinical microbiology and infection : the official publication of the European Society of Clinical Microbiology and Infectious Diseases 18: E16-E19.
160. Sidira P, Nikza P, Danis K, Panagiotopoulos T, Samara D, et al. (2013) Prevalence of Crimean-Congo hemorrhagic fever virus antibodies in Greek residents in the area where the AP92 strain was isolated. Hippokratia 17: 322-325.
161. Sun S, Dai X, Aishan M, Wang X, Meng W, et al. (2009) Epidemiology and phylogenetic analysis of crimean-congo hemorrhagic fever viruses in xinjiang, china. Journal of clinical microbiology 47: 2536-2543.
162. Swanepoel R, Struthers JK, Shepherd AJ, McGillivray GM, Nel MJ, et al. (1983) Crimean-congo hemorrhagic fever in South Africa. The American journal of tropical medicine and hygiene 32: 1407-1415.
163. Tarantola A, Ergonul O, Tattevin P (2007) Estimates and prevention of Crimean-Congo hemorrhagic fever risks for health-care workers. Crimean-Congo Hemorrhagic Fever: A Global Perspective. pp. 281-294.
164. Telmadarraiy Z, Moradi AR, Vatandoost R, Mostafavi E, Oshaghi MA, et al. (2008) Crimean-congo hemorrhagic fever: A seroepidemiological and molecular survey in Bahar, Hamadan province of Iran. Asian Journal of Animal and Veterinary Advances 3: 321-327.
165. Temocin F, Köse H, Sarı T, Duygu F, Şahin RO (2018) Seroprevalence of Crimean-Congo hemorrhagic fever among health care workers in a hospital in an endemic region of Turkey. Journal of infection in developing countries 12: 587-591.
166. Tigoi C, Lwande O, Orindi B, Irura Z, Ongus J, et al. (2015) Seroepidemiology of selected arboviruses in febrile patients visiting selected health facilities in the lake/river basin areas of Lake Baringo, Lake Naivasha, and Tana River, Kenya. Vector borne and zoonotic diseases (Larchmont, NY) 15: 124-132.
167. Todd CS, Mansoor GF, Buhler C, Rahimi H, Zekria R, et al. (2016) Prevalence of Zoonotic and Vector-Borne Infections Among Afghan National Army Recruits in Afghanistan. Vector borne and zoonotic diseases (Larchmont, NY) 16: 501-506.
168. Tomori O, Fabiyi A, Sorungbe A, Smith A, McCormick JB (1988) Viral hemorrhagic fever antibodies in Nigerian populations. The American journal of tropical medicine and hygiene 38: 407-410.
169. Vawda S, Goedhals D, Bester PA, Burt F (2018) Seroepidemiologic Survey of Crimean-Congo Hemorrhagic Fever Virus in Selected Risk Groups, South Africa. Emerging infectious diseases 24: 1360-1363.
170. Voorhees MA, Padilla SL, Jamsransuren D, Koehle JW, Delp KL, et al. (2018) Crimean-Congo hemorrhagic fever virus, Mongolia, 2013–2014. Emerging Infectious Diseases 24: 2202-2209.
171. Watts DM, El-Tigani A, Botros BAM, Salib AW, Olson JG, et al. (1994) Arthropod-borne viral infections associated with a fever outbreak in the Northern Province of Sudan. Journal of Tropical Medicine and Hygiene 97: 228-230.

172. Williams RJ, Al-Busaidy S, Mehta FR, Maupin GO, Wagoner KD, et al. (2000) Crimean-congo haemorrhagic fever: a seroepidemiological and tick survey in the Sultanate of Oman. Tropical medicine & international health : TM & IH 5: 99-106.
173. Wilson ML, LeGuenno B, Guillaud M, Desoutter D, Gonzalez JP, et al. (1990) Distribution of Crimean-Congo hemorrhagic fever viral antibody in Senegal: environmental and vectorial correlates. The American journal of tropical medicine and hygiene 43: 557-566.
174. Xia H, Li P, Yang J, Pan L, Zhao J, et al. (2011) Epidemiological survey of Crimean-Congo hemorrhagic fever virus in Yunnan, China, 2008. International journal of infectious diseases : IJID : official publication of the International Society for Infectious Diseases 15: e459-e463.
175. Yagci-Caglayik D, Korukluoglu G, Uyar Y (2014) Seroprevalence and risk factors of Crimean-Congo hemorrhagic fever in selected seven provinces in Turkey. Journal of medical virology 86: 306-314.
