# Supplementary material for: Worldwide epidemiology of Crimean-Congo Hemorrhagic Fever Virus in humans, ticks and other animal species, a systematic review and meta-analysis
Source: PLoS Negl Trop Dis. 2021 Apr 22;15(4):e0009299. doi: 10.1371/journal.pntd.0009299 (PMC8096040; doi:10.1371/journal.pntd.0009299)
Supplement: S3 Text — (PDF) [file pntd.0009299.s003.pdf]

S3 Text: Reference list of studies on Crimean-Congo hemorrhagic fever virus global prevalence in ticks

1. Albayrak H, Ozan E, Kurt M (2010) An antigenic investigation of Crimean-Congo hemorrhagic fever virus (CCHFV) in hard ticks from provinces in northern Turkey. Tropical animal health and production 42: 1323-1325.
2. Albayrak H, Ozan E, Kurt M (2012) Serosurvey and molecular detection of Crimean-Congo hemorrhagic fever virus (CCHFV) in northern Turkey. Tropical animal health and production 44: 1667-1671.
3. Azagi T, Klement E, Perlman G, Lustig Y, Mumcuoglu KY, et al. (2017) Francisella-Like Endosymbionts and Rickettsia Species in Local and Imported Hyalomma Ticks. Applied and environmental microbiology 83: e01302-01317.
4. Balinandi S, Patel K, Ojwang J, Kyondo J, Mulei S, et al. (2018) Investigation of an isolated case of human Crimean–Congo hemorrhagic fever in Central Uganda, 2015. International Journal of Infectious Diseases 68: 88-93.
5. Bażanów BA, Pacoń J, Gadzała Ł, Frącka A, Welz M, et al. (2017) Vector and Serologic Survey for Crimean-Congo Hemorrhagic Fever Virus in Poland. Vector borne and zoonotic diseases (Larchmont, NY) 17: 510-513.
6. Biglari P, Chinikar S, Belqeiszadeh H, Telmadarraiy Z, Mostafavi E, et al. (2016) Phylogeny of tick-derived Crimean-Congo hemorrhagic fever virus strains in Iran. Ticks and tick-borne diseases 7: 1216-1221.
7. Champour M, Chinikar S, Mohammadi G, Razmi G, Shah-Hosseini N, et al. (2016) Molecular epidemiology of Crimean–Congo hemorrhagic fever virus detected from ticks of one humped camels (Camelus dromedarius) population in northeastern Iran. Journal of Parasitic Diseases 40: 110-115.
8. Chinikar S, Ghiasi SM, Naddaf S, Piazak N, Moradi M, et al. (2012) Serological evaluation of Crimean-Congo hemorrhagic fever in humans with high-risk professions living in enzootic regions of Isfahan province of Iran and genetic analysis of circulating strains. Vector borne and zoonotic diseases (Larchmont, NY) 12: 733-738.
9. Chisholm K, Dueger E, Fahmy NT, Samaha HAT, Zayed A, et al. (2012) Crimean-congo hemorrhagic fever virus in ticks from imported livestock, Egypt. Emerging infectious diseases 18: 181-182.
10. Choubdar N, Oshaghi MA, Rafinejad J, Pourmand MR, Maleki-Ravasan N, et al. (2019) Effect of Meteorological Factors on Hyalomma Species Composition and Their Host Preference, Seasonal Prevalence and Infection Status to Crimean-Congo Haemorrhagic Fever in Iran. Journal of arthropod-borne diseases 13: 268-283.
11. Dinçer E, Brinkmann A, Hekimoğlu O, Hacıoğlu S, Földes K, et al. (2017) Generic amplification and next generation sequencing reveal Crimean-Congo hemorrhagic fever virus AP92-like strain and distinct tick phleboviruses in Anatolia, Turkey. Parasites & vectors 10: 335-335.
12. England ME, Phipps P, Medlock JM, Atkinson PM, Atkinson B, et al. (2016) Hyalomma ticks on northward migrating birds in southern Spain: Implications for the risk of entry of Crimean-Congo haemorrhagic fever virus to Great Britain. Journal of vector ecology : journal of the Society for Vector Ecology 41: 128-134.
13. Estrada-Peña A, Palomar AM, Santibáñez P, Sánchez N, Habela MA, et al. (2012) Crimean-Congo hemorrhagic fever virus in ticks, Southwestern Europe, 2010. Emerging infectious diseases 18: 179-180.
14. Faghihi F, Telmadarraiy Z, Chinikar S, Nowotny N, Fooks AR, et al. (2018) Spatial and phylodynamic survey on Crimean-Congo hemorrhagic fever virus strains in northeast of Iran. Jundishapur Journal of Microbiology 11.

15. Fajs L, Humolli I, Saksida A, Knap N, Jelovšek M, et al. (2014) Prevalence of Crimean-Congo hemorrhagic fever virus in healthy population, livestock and ticks in Kosovo. PloS one 9: e110982-e110982.
16. Fakoorziba MR, Golmohammadi P, Moradzadeh R, Moemenbellah-Fard MD, Azizi K, et al. (2012) Reverse transcription PCR-based detection of Crimean-Congo hemorrhagic fever virus isolated from ticks of domestic ruminants in Kurdistan province of Iran. Vector borne and zoonotic diseases (Larchmont, NY) 12: 794-799.
17. Fakoorziba MR, Naddaf-Sani AA, Moemenbellah-Fard MD, Azizi K, Ahmadnia S, et al. (2015) First phylogenetic analysis of a Crimean-Congo hemorrhagic fever virus genome in naturally infected Rhipicephalus appendiculatus ticks (Acari: Ixodidae). Archives of virology 160: 1197-1209.
18. Fares W, Dachraoui K, Najjar C, Younsi H, Findlay-Wilson S, et al. (2019) Absence of Crimean-Congo haemorrhagic fever virus in the tick Hyalomma aegyptium parasitizing the spur-thighed tortoise (Testudo graeca) in Tunisia. Parasite (Paris, France) 26: 35-35.
19. Farhadpour F, Telmadarraiy Z, Chinikar S, Akbarzadeh K, Moemenbellah-Fard MD, et al. (2016) Molecular detection of Crimean-Congo haemorrhagic fever virus in ticks collected from infested livestock populations in a New Endemic Area, South of Iran. Tropical medicine & international health : TM & IH 21: 340-347.
20. Fernández de Mera IG, Chaligiannis I, Hernández-Jarguín A, Villar M, Mateos-Hernández L, et al. (2017) Combination of RT-PCR and proteomics for the identification of Crimean-Congo hemorrhagic fever virus in ticks. Heliyon 3: e00353-e00353.
21. Gergova I, Kamarinchev B (2013) Comparison of the prevalence of Crimean-Congo hemorrhagic fever virus in endemic and non-endemic Bulgarian locations. Journal of vector borne diseases 50: 265-270.
22. Gergova I, Kunchev M, Kamarinchev B (2012) Crimean-Congo hemorrhagic fever virus-tick survey in endemic areas in Bulgaria. Journal of medical virology 84: 608-614.
23. Gevorgyan H, Grigoryan GG, Atoyan HA, Rukhkyan M, Hakobyan A, et al. (2019) Evidence of Crimean-Congo Haemorrhagic Fever Virus Occurrence in Ixodidae Ticks of Armenia. Journal of arthropod-borne diseases 13: 9-16.
24. Grech-Angelini S, Stachurski F, Vayssier-Taussat M, Devillers E, Casabianca F, et al. (2019) Tick-borne pathogens in ticks (Acari: Ixodidae) collected from various domestic and wild hosts in Corsica (France), a Mediterranean island environment. Transboundary and Emerging Diseases.
25. Gunes T, Poyraz O, Vatansever Z (2011) Crimean-Congo hemorrhagic fever virus in ticks collected from humans, livestock, and picnic sites in the hyperendemic region of Turkey. Vector borne and zoonotic diseases (Larchmont, NY) 11: 1411-1416.
26. Horton KC, Fahmy NT, Watany N, Zayed A, Mohamed A, et al. (2016) Crimean Congo Hemorrhagic Fever Virus and Alkhurma (Alkhumra) Virus in Ticks in Djibouti. Vector borne and zoonotic diseases (Larchmont, NY) 16: 680-682.
27. Hosseini-Vasoukolaei N, Chinikar S, Telmadarraiy Z, Faghihi F, Hosseini-Vasoukolaei M (2016) Serological and molecular epidemiology of crimean-congo hemorrhagic fever in Ghaemshahr county in Mazandaran province; Iran. Tropical Biomedicine 33: 807-813.
28. Kalaycioğlu AT, Durmaz R, Güldemir D, Korukluoğlu G, Ertek M (2013) Genetic analysis of the partial M RNA segment of Crimean-Congo hemorrhagic fever viruses in Turkey. Kafkas Universitesi Veteriner Fakultesi Dergisi 19: A147-A152.
29. Kasi KK, von Arnim F, Schulz A, Rehman A, Chudhary A, et al. (2020) Crimean-Congo haemorrhagic fever virus in ticks collected from livestock in Balochistan, Pakistan. Transboundary and emerging diseases: 10.1111/tbed.13488.
30. Kautman M, Tiar G, Papa A, Široký P (2016) AP92-like Crimean-Congo Hemorrhagic Fever Virus in Hyalomma aegyptium Ticks, Algeria. Emerging infectious diseases 22: 354-356.

31. Kayedi MH, Chinikar S, Mostafavi E, Khakifirouz S, Jalali T, et al. (2015) Crimean-Congo Hemorrhagic Fever Virus Clade IV (Asia 1) in Ticks of Western Iran. Journal of medical entomology 52: 1144-1149.
32. Khan AS, Maupin GO, Rollin PE, Noor AM, Shurie HH, et al. (1997) An outbreak of Crimean-Congo hemorrhagic fever in the United Arab Emirates, 1994-1995. The American journal of tropical medicine and hygiene 57: 519-525.
33. Leblebicioglu H, Eroglu C, Erciyas-Yavuz K, Hokelek M, Acici M, et al. (2014) Role of migratory birds in spreading Crimean-Congo hemorrhagic fever, Turkey. Emerging infectious diseases 20: 1331-1334.
34. Mancini F, Toma L, Ciervo A, Di Luca M, Faggioni G, et al. (2013) Virus investigation in ticks from migratory birds in Italy. The new microbiologica 36: 433-434.
35. Mancuso E, Toma L, Polci A, d'Alessio SG, Di Luca M, et al. (2019) Crimean-Congo Hemorrhagic Fever Virus Genome in Tick from Migratory Bird, Italy. Emerging infectious diseases 25: 1418-1420.
36. Mehravaran A, Moradi M, Telmadarraiy Z, Mostafavi E, Moradi AR, et al. (2013) Molecular detection of Crimean-Congo haemorrhagic fever (CCHF) virus in ticks from southeastern Iran. Ticks and tick-borne diseases 4: 35-38.
37. Midilli K, Gargili A, Ergonul O, Elevli M, Ergin S, et al. (2009) The first clinical case due to AP92 like strain of Crimean-Congo Hemorrhagic Fever virus and a field survey. BMC infectious diseases 9: 90-90.
38. Mohammadian M, Chinikar S, Telmadarraiy Z, Vatandoost H, Oshaghi MA, et al. (2016) Molecular Assay on Crimean Congo Hemorrhagic Fever Virus in Ticks (Ixodidae) Collected from Kermanshah Province, Western Iran. Journal of arthropod-borne diseases 10: 381-391.
39. Mourya DT, Yadav PD, Shete A, Majumdar TD, Kanani A, et al. (2014) Serosurvey of Crimean-Congo hemorrhagic fever virus in domestic animals, Gujarat, India, 2013. Vector borne and zoonotic diseases (Larchmont, NY) 14: 690-692.
40. Mourya DT, Yadav PD, Shete AM, Gurav YK, Raut CG, et al. (2012) Detection, isolation and confirmation of Crimean-Congo hemorrhagic fever virus in human, ticks and animals in Ahmadabad, India, 2010-2011. PLoS neglected tropical diseases 6: e1653-e1653.
41. Nabeth P, Cheikh DO, Lo B, Faye O, Vall IOM, et al. (2004) Crimean-Congo hemorrhagic fever, Mauritania. Emerging infectious diseases 10: 2143-2149.
42. Negredo A, Habela MÁ, Ramírez de Arellano E, Diez F, Lasala F, et al. (2019) Survey of Crimean-Congo Hemorrhagic Fever Enzootic Focus, Spain, 2011-2015. Emerging infectious diseases 25: 1177-1184.
43. Orkun Ö, Karaer Z, Çakmak A, Nalbantoğlu S (2017) Crimean-Congo hemorrhagic fever virus in ticks in Turkey: A broad range tick surveillance study. Infection, genetics and evolution : journal of molecular epidemiology and evolutionary genetics in infectious diseases 52: 59-66.
44. Ozdarendeli A, Aydin K, Tonbak S, Aktas M, Altay K, et al. (2008) Genetic analysis of the M RNA segment of Crimean-Congo hemorrhagic fever virus strains in Turkey. Archives of virology 153: 37-44.
45. Palomar AM, Portillo A, Mazuelas D, Roncero L, Arizaga J, et al. (2016) Molecular analysis of Crimean-Congo hemorrhagic fever virus and Rickettsia in Hyalomma marginatum ticks removed from patients (Spain) and birds (Spain and Morocco), 2009-2015. Ticks and tick-borne diseases 7: 983-987.
46. Palomar AM, Portillo A, Santibáñez S, García-Álvarez L, Muñoz-Sanz A, et al. (2017) Molecular (ticks) and serological (humans) study of Crimean-Congo hemorrhagic fever virus in the Iberian Peninsula, 2013-2015. Enfermedades infecciosas y microbiologia clinica 35: 344-347.
47. Panayotova E, Papa A, Trifonova I, Christova I (2016) Crimean-Congo hemorrhagic fever virus lineages Europe 1 and Europe 2 in Bulgarian ticks. Ticks and tick-borne diseases 7: 1024-1028.

48. Papa A, Kontana A, Tsioka K, Chaligiannis I, Sotiraki S (2017) Molecular detection of Crimean-Congo hemorrhagic fever virus in ticks, Greece, 2012-2014. Parasitology research 116: 3057-3063.
49. Papa A, Velo E, Kadiaj P, Tsioka K, Kontana A, et al. (2017) Crimean-Congo hemorrhagic fever virus in ticks collected from livestock in Albania. Infection, genetics and evolution : journal of molecular epidemiology and evolutionary genetics in infectious diseases 54: 496-500.
50. Papa A, Velo E, Papadimitriou E, Cahani G, Kota M, et al. (2009) Ecology of the Crimean-Congo hemorrhagic fever endemic area in Albania. Vector borne and zoonotic diseases (Larchmont, NY) 9: 713-716.
51. Pascucci I, Di Domenico M, Capobianco Dondona G, Di Gennaro A, Polci A, et al. (2019) Assessing the role of migratory birds in the introduction of ticks and tick-borne pathogens from African countries: An Italian experience. Ticks and tick-borne diseases 10: 101272-101272.
52. Rodriguez LL, Maupin GO, Ksiazek TG, Rollin PE, Khan AS, et al. (1997) Molecular investigation of a multisource outbreak of Crimean-Congo hemorrhagic fever in the United Arab Emirates. The American journal of tropical medicine and hygiene 57: 512-518.
53. Saghafipour A, Mousazadeh-Mojarrad A, Arzamani N, Telmadarraiy Z, Rajabzadeh R, et al. (2019) Molecular and seroepidemiological survey on Crimean-Congo Hemorrhagic Fever Virus in Northeast of Iran. Medical journal of the Islamic Republic of Iran 33: 41-41.
54. Saluzzo JF, Digoutte JP, Camicas JL, Chauvancy G (1985) Crimean-Congo haemorrhagic fever and Rift Valley fever in south-eastern Mauritania. Lancet (London, England) 1: 116-116.
55. Sang R, Lutomiah J, Koka H, Makio A, Chepkorir E, et al. (2011) Crimean-Congo hemorrhagic fever virus in Hyalommid ticks, northeastern Kenya. Emerging infectious diseases 17: 1502-1505.
56. Sedaghat MM, Sarani M, Chinikar S, Telmadarraiy Z, Moghaddam AS, et al. (2017) Vector prevalence and detection of Crimean-Congo haemorrhagic fever virus in Golestan Province, Iran. Journal of vector borne diseases 54: 353-357.
57. Shahhosseini N, Jafarbekloo A, Telmadarraiy Z, Chinikar S, Haeri A, et al. (2017) Co-circulation of Crimean-Congo Hemorrhagic Fever virus strains Asia 1 and 2 between the border of Iran and Pakistan. Heliyon 3: e00439-e00439.
58. Sharifinia N, Rafinejad J, Hanafi-Bojd AA, Chinikar S, Piazak N, et al. (2015) Hard ticks (Ixodidae) and Crimean-Congo hemorrhagic fever virus in south west of Iran. Acta medica Iranica 53: 177-181.
59. Shepherd AJ, Swanepoel R, Shepherd SP, Leman PA, Blackburn NK, et al. (1985) A nosocomial outbreak of Crimean-Congo haemorrhagic fever at Tygerberg Hospital. Part V. Virological and serological observations. South African medical journal = Suid-Afrikaanse tydskrif vir geneeskunde 68: 733-736.
60. Sherifi K, Cadar D, Muji S, Robaj A, Ahmeti S, et al. (2014) Crimean-Congo hemorrhagic fever virus clades V and VI (Europe 1 and 2) in ticks in Kosovo, 2012. PLoS neglected tropical diseases 8: e3168-e3168.
61. Sherifi K, Rexhepi A, Berxholi K, Mehmedi B, Gecaj RM, et al. (2018) Crimean-Congo Hemorrhagic Fever Virus and Borrelia burgdorferi sensu lato in Ticks from Kosovo and Albania. Frontiers in veterinary science 5: 38-38.
62. Shuaib YA, Elhag AM-AW, Brima YA, Abdalla MA, Bakiet AO, et al. (2020) Ixodid tick species and two tick-borne pathogens in three areas in the Sudan. Parasitology research 119: 385-394.
63. Široký P, Bělohlávek T, Papoušek I, Jandzik D, Mikulíček P, et al. (2014) Hidden threat of tortoise ticks: high prevalence of Crimean-Congo haemorrhagic fever virus in ticks Hyalomma aegyptium in the Middle East. Parasites & vectors 7: 101-101.
64. Sun S, Dai X, Aishan M, Wang X, Meng W, et al. (2009) Epidemiology and phylogenetic analysis of crimean-congo hemorrhagic fever viruses in xinjiang, china. Journal of clinical microbiology 47: 2536-2543.
65. Tahmasebi F, Ghiasi SM, Mostafavi E, Moradi M, Piazak N, et al. (2010) Molecular epidemiology of Crimean- Congo hemorrhagic fever virus genome isolated from ticks of Hamadan province of Iran. Journal of vector borne diseases 47: 211-216.

66. Tekin S, Bursali A, Mutluay N, Keskin A, Dundar E (2012) Crimean-Congo hemorrhagic fever virus in various ixodid tick species from a highly endemic area. Veterinary parasitology 186: 546-552.
67. Telmadarraiy Z, Ghiasi SM, Moradi M, Vatandoost H, Eshraghian MR, et al. (2010) A survey of Crimean-Congo haemorrhagic fever in livestock and ticks in Ardabil Province, Iran during 2004-2005. Scandinavian journal of infectious diseases 42: 137-141.
68. Telmadarraiy Z, Moradi AR, Vatandoost R, Mostafavi E, Oshaghi MA, et al. (2008) Crimean-congo hemorrhagic fever: A seroepidemiological and molecular survey in Bahar, Hamadan province of Iran. Asian Journal of Animal and Veterinary Advances 3: 321-327.
69. Tonbak S, Aktas M, Altay K, Azkur AK, Kalkan A, et al. (2006) Crimean-Congo hemorrhagic fever virus: genetic analysis and tick survey in Turkey. Journal of clinical microbiology 44: 4120-4124.
70. Williams RJ, Al-Busaidy S, Mehta FR, Maupin GO, Wagoner KD, et al. (2000) Crimean-congo haemorrhagic fever: a seroepidemiological and tick survey in the Sultanate of Oman. Tropical medicine & international health : TM & IH 5: 99-106.
71. Yaser SA, Sadegh C, Zakkyeh T, Hassan V, Maryam M, et al. (2011) Crimean--Congo hemorrhagic fever: a molecular survey on hard ticks (Ixodidae) in Yazd province, Iran. Asian Pacific journal of tropical medicine 4: 61-63.
72. Akuffo R, Brandful JAM, Zayed A, Adjei A, Watany N, et al. (2016) Crimean-Congo hemorrhagic fever virus in livestock ticks and animal handler seroprevalence at an abattoir in Ghana. BMC infectious diseases 16: 324-324.
73. Albayrak H, Ozan E, Kurt M (2010) Molecular detection of Crimean-Congo haemorrhagic fever virus (CCHFV) but not West Nile virus (WNV) in hard ticks from provinces in northern Turkey. Zoonoses and public health 57: e156-e160.
74. Bursali A, Tekin S, Keskin A, Ekici M, Dundar E (2011) Species diversity of ixodid ticks feeding on humans in Amasya, Turkey: seasonal abundance and presence of Crimean-Congo hemorrhagic fever virus. Journal of medical entomology 48: 85-93.
75. Cajimat MNB, Rodriguez SE, Schuster IUE, Swetnam DM, Ksiazek TG, et al. (2017) Genomic Characterization of Crimean-Congo Hemorrhagic Fever Virus in Hyalomma Tick from Spain, 2014. Vector borne and zoonotic diseases (Larchmont, NY) 17: 714-719.
76. Gargili A, Midilli K, Ergonul O, Ergin S, Alp HG, et al. (2011) Crimean-Congo hemorrhagic fever in European part of Turkey: genetic analysis of the virus strains from ticks and a seroepidemiological study in humans. Vector borne and zoonotic diseases (Larchmont, NY) 11: 747-752.
77. Hassanein KM, El-Azazy OM (2000) Isolation of Crimean-Congo hemorrhagic fever virus from ticks on imported Sudanese sheep in Saudi Arabia. Annals of Saudi medicine 20: 153-154.
78. Hekimoglu O, Ozer N, Ergunay K, Ozkul A (2012) Species distribution and detection of Crimean Congo Hemorrhagic Fever Virus (CCHFV) in field-collected ticks in Ankara Province, Central Anatolia, Turkey. Experimental & applied acarology 56: 75-84.
79. Kulichenko AN, Volynkina AS, Kotenev ES, Pisarenko SV, Shaposhnikova LI, et al. (2016) A new genetic variant of the Crimean–Congo hemorrhagic fever virus isolated in Crimea. Molecular Genetics, Microbiology and Virology 31: 94-101.
80. Mathiot CC, Fontenille D, Digoutte JP, Coulanges P (1988) First isolation of Congo-Crimean haemorrhagic fever virus in Madagascar. Annales de l'Institut Pasteur Virology 139: 239-241.
81. Moming A, Yue X, Shen S, Chang C, Wang C, et al. (2018) Prevalence and Phylogenetic Analysis of Crimean-Congo Hemorrhagic Fever Virus in Ticks from Different Ecosystems in Xinjiang, China. Virologica Sinica 33: 67-73.
82. Swanepoel R, Struthers JK, Shepherd AJ, McGillivray GM, Nel MJ, et al. (1983) Crimean-congo hemorrhagic fever in South Africa. The American journal of tropical medicine and hygiene 32: 1407-1415.
83. Voorhees MA, Padilla SL, Jamsransuren D, Koehle JW, Delp KL, et al. (2018) Crimean-Congo hemorrhagic fever virus, Mongolia, 2013–2014. Emerging Infectious Diseases 24: 2202-2209.

84. Wood OL, Lee VH, Ash JS, Casals J (1978) Crimean-congo hemorrhagic fever, Thogoto, dugbe, and Jos viruses isolated from ixodid ticks in Ethiopia. The American journal of tropical medicine and hygiene 27: 600-604.
85. Xia H, Li P, Yang J, Pan L, Zhao J, et al. (2011) Epidemiological survey of Crimean-Congo hemorrhagic fever virus in Yunnan, China, 2008. International journal of infectious diseases : IJID : official publication of the International Society for Infectious Diseases 15: e459-e463.
86. Yadav PD, Gurav YK, Mistry M, Shete AM, Sarkale P, et al. (2014) Emergence of Crimean-Congo hemorrhagic fever in Amreli District of Gujarat State, India, June to July 2013. International journal of infectious diseases : IJID : official publication of the International Society for Infectious Diseases 18: 97-100.
87. Yashina L, Petrova I, Seregin S, Vyshemirskii O, Lvov D, et al. (2003) Genetic variability of Crimean-Congo haemorrhagic fever virus in Russia and Central Asia. The Journal of general virology 84: 1199-1206.
88. Yesilbag K, Aydin L, Dincer E, Alpay G, Girisgin AO, et al. (2013) Tick survey and detection of Crimean-Congo hemorrhagic fever virus in tick species from a non-endemic area, South Marmara region, Turkey. Experimental & applied acarology 60: 253-261.
89. Zivcec M, Maïga O, Kelly A, Feldmann F, Sogoba N, et al. (2014) Unique strain of Crimean-Congo hemorrhagic fever virus, Mali. Emerging infectious diseases 20: 911-913.
