# Supplementary material for: Worldwide epidemiology of Crimean-Congo Hemorrhagic Fever Virus in humans, ticks and other animal species, a systematic review and meta-analysis
Source: PLoS Negl Trop Dis. 2021 Apr 22;15(4):e0009299. doi: 10.1371/journal.pntd.0009299 (PMC8096040; doi:10.1371/journal.pntd.0009299)
Supplement: S4 Text — (PDF) [file pntd.0009299.s004.pdf]

S4 Text: Reference list of studies on Crimean-Congo hemorrhagic fever virus global prevalence in other animal species

1. Albayrak H, Ozan E, Kurt M (2012) Serosurvey and molecular detection of Crimean-Congo hemorrhagic fever virus (CCHFV) in northern Turkey. Tropical animal health and production 44: 1667-1671.
2. Mahzounieh M, Dincer E, Faraji A, Akin H, Akkutay AZ, et al. (2012) Relationship between Crimean-Congo hemorrhagic fever virus strains circulating in Iran and Turkey: possibilities for transborder transmission. Vector borne and zoonotic diseases (Larchmont, NY) 12: 782-785.
3. Mourya DT, Yadav PD, Shete AM, Gurav YK, Raut CG, et al. (2012) Detection, isolation and confirmation of Crimean-Congo hemorrhagic fever virus in human, ticks and animals in Ahmadabad, India, 2010-2011. PLoS neglected tropical diseases 6: e1653-e1653.
4. Saluzzo JF, Digoutte JP, Camicas JL, Chauvancy G (1985) Crimean-Congo haemorrhagic fever and Rift Valley fever in south-eastern Mauritania. Lancet (London, England) 1: 116-116.
5. Swanepoel R, Struthers JK, Shepherd AJ, McGillivray GM, Nel MJ, et al. (1983) Crimean-congo hemorrhagic fever in South Africa. The American journal of tropical medicine and hygiene 32: 1407-1415.
6. Tuncer P, Yesilbag K, Alpay G, Dincer E, Girisgin AO, et al. (2014) Crimean-Congo Hemorrhagic Fever infection in domestic animals in Marmara region, Western Turkey. Ankara Universitesi Veteriner Fakultesi Dergisi 61: 49-53.
7. Adam IA, Mahmoud MAM, Aradaib IE (2013) A seroepidemiological survey of Crimean Congo hemorrhagic fever among cattle in North Kordufan State, Sudan. Virology journal 10: 178-178.
8. Al Adhamy SB, E.A.M D, Saadallah (1992) Sero-epidemiological study of crimean-congo hemorrhagic fever [CCHF] during 36 months. Iraqi Med J 40-42: 232-235.
9. Barthel R, Mohareb E, Younan R, Gladnishka T, Kalvatchev N, et al. (2014) Seroprevalance of Crimean–Congo haemorrhagic fever in Bulgarian livestock. Biotechnology and Biotechnological Equipment 28: 540-542.
10. Bażanów BA, Pacoń J, Gadzała Ł, Frącka A, Welz M, et al. (2017) Vector and Serologic Survey for Crimean-Congo Hemorrhagic Fever Virus in Poland. Vector borne and zoonotic diseases (Larchmont, NY) 17: 510-513.
11. Behrooz A, Hamid Reza T, Sadegh C, Mohammad D, Navid J, et al. (2006) Seroepidemiology of Crimean-Congo hemorrhagic fever in the local and imported sheep in Isfahan province, Iran, 2002. Iran J Clin Infect Dis 1: 19-23.
12. Bokaie S, Mostafavi E, Haghdoost AA, Keyvanfar H, Gooya MM, et al. (2008) Crimean Congo hemorrhagic fever in Northeast of Iran. Journal of Animal and Veterinary Advances 7: 343-350.
13. Burt FJ, Spencer DC, Leman PA, Patterson B, Swanepoel R (1996) Investigation of tick-borne viruses as pathogens of humans in South Africa and evidence of Dugbe virus infection in a patient with prolonged thrombocytopenia. Epidemiology and infection 116: 353-361.
14. Burt FJ, Swanepoel R, Braack LE (1993) Enzyme-linked immunosorbent assays for the detection of antibody to Crimean-Congo haemorrhagic fever virus in the sera of livestock and wild vertebrates. Epidemiology and infection 111: 547-557.
15. Ceianu CS, Panculescu-Gatej RI, Coudrier D, Bouloy M (2012) First serologic evidence for the circulation of Crimean-Congo hemorrhagic fever virus in Romania. Vector borne and zoonotic diseases (Larchmont, NY) 12: 718-721.
16. Champour M, Mohammadi G, Chinikar S, Razmi G, Shah-Hosseini N, et al. (2014) Seroepidemiology of Crimean-Congo hemorrhagic fever virus in one-humped camels (Camelus dromedarius) population in northeast of Iran. Journal of vector borne diseases 51: 62-65.

17. Chinikar S, Ghiasi SM, Moradi M, Goya MM, Shirzadi MR, et al. (2010) Geographical distribution and surveillance of Crimean-Congo hemorrhagic fever in Iran. Vector borne and zoonotic diseases (Larchmont, NY) 10: 705-708.
18. Chinikar S, Goya MM, Shirzadi MR, Ghiasi SM, Mirahmadi R, et al. (2008) Surveillance and laboratory detection system of Crimean-Congo haemorrhagic fever in Iran. Transboundary and emerging diseases 55: 200-204.
19. Chinikar S, Moghadam AH, Parizadeh SJ, Moradi M, Bayat N, et al. (2012) Seroepidemiology of crimean congo hemorrhagic Fever in slaughterhouse workers in north eastern iran. Iranian journal of public health 41: 72-77.
20. Christova I, Panayotova E, Groschup MH, Trifonova I, Tchakarova S, et al. (2018) High seroprevalence for Crimean–Congo haemorrhagic fever virus in ruminants in the absence of reported human cases in many regions of Bulgaria. Experimental and Applied Acarology 75: 227-234.
21. Darwish MA, Hoogstraal H, Roberts TJ, Ghazi R, Amer T (1983) A sero-epidemiological survey for Bunyaviridae and certain other arboviruses in Pakistan. Transactions of the Royal Society of Tropical Medicine and Hygiene 77: 446-450.
22. De Liberato C, Frontoso R, Magliano A, Montemaggiori A, Autorino GL, et al. (2018) Monitoring for the possible introduction of Crimean-Congo haemorrhagic fever virus in Italy based on tick sampling on migratory birds and serological survey of sheep flocks. Preventive veterinary medicine 149: 47-52.
23. Faghihi F, Telmadarraiy Z, Chinikar S, Nowotny N, Fooks AR, et al. (2018) Spatial and phylodynamic survey on Crimean-Congo hemorrhagic fever virus strains in northeast of Iran. Jundishapur Journal of Microbiology 11.
24. Fajs L, Humolli I, Saksida A, Knap N, Jelovšek M, et al. (2014) Prevalence of Crimean-Congo hemorrhagic fever virus in healthy population, livestock and ticks in Kosovo. PloS one 9: e110982-e110982.
25. Filipe AR, Calisher CH, Lazuick J (1985) Antibodies to Congo-Crimean haemorrhagic fever, Dhori, Thogoto and Bhanja viruses in southern Portugal. Acta virologica 29: 324-328.
26. Fisher-Hoch SP, McCormick JB, Swanepoel R, Van Middlekoop A, Harvey S, et al. (1992) Risk of human infections with Crimean-Congo hemorrhagic fever virus in a South African rural community. The American journal of tropical medicine and hygiene 47: 337-345.
27. Földes F, Madai M, Németh V, Zana B, Papp H, et al. (2019) Serologic survey of the Crimean-Congo haemorrhagic fever virus infection among wild rodents in Hungary. Ticks and tick-borne diseases 10: 101258-101258.
28. Gergova I, Kamarinchev B (2013) Comparison of the prevalence of Crimean-Congo hemorrhagic fever virus in endemic and non-endemic Bulgarian locations. Journal of vector borne diseases 50: 265-270.
29. Gonzalez JP, LeGuenno B, Guillaud M, Wilson ML (1990) A fatal case of Crimean-Congo haemorrhagic fever in Mauritania: virological and serological evidence suggesting epidemic transmission. Transactions of the Royal Society of Tropical Medicine and Hygiene 84: 573-576.
30. Guilherme JM, Gonella-Legall C, Legall F, Nakoume E, Vincent J (1996) Seroprevalence of five arboviruses in Zebu cattle in the Central African Republic. Transactions of the Royal Society of Tropical Medicine and Hygiene 90: 31-33.
31. Hassanein KM, el-Azazy OM, Yousef HM (1997) Detection of Crimean-Congo haemorrhagic fever virus antibodies in humans and imported livestock in Saudi Arabia. Transactions of the Royal Society of Tropical Medicine and Hygiene 91: 536-537.
32. Horton KC, Wasfy M, Samaha H, Abdel-Rahman B, Safwat S, et al. (2014) Serosurvey for zoonotic viral and bacterial pathogens among slaughtered livestock in Egypt. Vector borne and zoonotic diseases (Larchmont, NY) 14: 633-639.
33. Horváth LB (1976) Precipitating antibodies to Crimean haemorrhagic fever virus in human sera collected in Hungary. Acta microbiologica Academiae Scientiarum Hungaricae 23: 331-335.

34. Hosseini-Vasoukolaei N, Chinikar S, Telmadarraiy Z, Faghihi F, Hosseini-Vasoukolaei M (2016) Serological and molecular epidemiology of crimean-congo hemorrhagic fever in Ghaemshahr county in Mazandaran province; Iran. Tropical Biomedicine 33: 807-813.
35. Ibrahim AM, Adam IA, Osman BT, Aradaib IE (2015) Epidemiological survey of Crimean Congo hemorrhagic fever virus in cattle in East Darfur State, Sudan. Ticks and tick-borne diseases 6: 439-444.
36. Kasi KK, Sas MA, Sauter-Louis C, von Arnim F, Gethmann JM, et al. (2020) Epidemiological investigations of Crimean-Congo haemorrhagic fever virus infection in sheep and goats in Balochistan, Pakistan. Ticks and tick-borne diseases 11: 101324-101324.
37. Khan AS, Maupin GO, Rollin PE, Noor AM, Shurie HH, et al. (1997) An outbreak of Crimean-Congo hemorrhagic fever in the United Arab Emirates, 1994-1995. The American journal of tropical medicine and hygiene 57: 519-525.
38. Lotfollahzadeh S, Nikbakht Boroujeni GR, Mokhber Dezfouli MR, Bokaei S (2011) A serosurvey of Crimean-Congo haemorrhagic fever virus in dairy cattle in Iran. Zoonoses and public health 58: 54-59.
39. Maiga O, Sas MA, Rosenke K, Kamissoko B, Mertens M, et al. (2017) Serosurvey of Crimean-Congo Hemorrhagic Fever Virus in Cattle, Mali, West Africa. The American journal of tropical medicine and hygiene 96: 1341-1345.
40. Mariner JC, Morrill J, Ksiazek TG (1995) Antibodies to hemorrhagic fever viruses in domestic livestock in Niger: Rift Valley fever and Crimean-Congo hemorrhagic fever. The American journal of tropical medicine and hygiene 53: 217-221.
41. Mertens M, Schuster I, Sas MA, Vatansever Z, Hubalek Z, et al. (2016) Crimean-Congo Hemorrhagic Fever Virus in Bulgaria and Turkey. Vector borne and zoonotic diseases (Larchmont, NY) 16: 619-623.
42. Mohamed M, Said A-R, Murad A, Graham R (2008) A serological survey of Crimean-Congo haemorrhagic fever in animals in the Sharkia Governorate of Egypt. Veterinaria italiana 44: 513-517.
43. Morrill JC, Soliman AK, Imam IZ, Botros BA, Moussa MI, et al. (1990) Serological evidence of Crimean-Congo haemorrhagic fever viral infection among camels imported into Egypt. The Journal of tropical medicine and hygiene 93: 201-204.
44. Mostafavi E, Chinikar S, Esmaeili S, Amiri FB, Tabrizi AMA, et al. (2012) Seroepidemiological survey of Crimean-Congo hemorrhagic fever among sheep in Mazandaran province, northern Iran. Vector borne and zoonotic diseases (Larchmont, NY) 12: 739-742.
45. Mostafavi E, Haghdoost A, Khakifirouz S, Chinikar S (2013) Spatial analysis of Crimean Congo hemorrhagic fever in Iran. The American journal of tropical medicine and hygiene 89: 1135-1141.
46. Mourya DT, Yadav PD, Shete A, Majumdar TD, Kanani A, et al. (2014) Serosurvey of Crimean-Congo hemorrhagic fever virus in domestic animals, Gujarat, India, 2013. Vector borne and zoonotic diseases (Larchmont, NY) 14: 690-692.
47. Mourya DT, Yadav PD, Shete AM, Sathe PS, Sarkale PC, et al. (2015) Cross-sectional Serosurvey of Crimean-Congo Hemorrhagic Fever Virus IgG in Livestock, India, 2013-2014. Emerging infectious diseases 21: 1837-1839.
48. Müller MA, Devignot S, Lattwein E, Corman VM, Maganga GD, et al. (2016) Evidence for widespread infection of African bats with Crimean-Congo hemorrhagic fever-like viruses. Scientific reports 6: 26637-26637.
49. Mustafa ML, Ayazi E, Mohareb E, Yingst S, Zayed A, et al. (2011) Crimean-Congo hemorrhagic fever, Afghanistan, 2009. Emerging infectious diseases 17: 1940-1941.
50. Nabeth P, Cheikh DO, Lo B, Faye O, Vall IOM, et al. (2004) Crimean-Congo hemorrhagic fever, Mauritania. Emerging infectious diseases 10: 2143-2149.
51. Németh V, Oldal M, Egyed L, Gyuranecz M, Erdélyi K, et al. (2013) Serologic evidence of Crimean-Congo hemorrhagic fever virus infection in Hungary. Vector borne and zoonotic diseases (Larchmont, NY) 13: 270-272.

52. Papa A, Papadopoulou E, Tsioka K, Kontana A, Pappa S, et al. (2018) Isolation and whole-genome sequencing of a Crimean-Congo hemorrhagic fever virus strain, Greece. Ticks and tick-borne diseases 9: 788-791.
53. Papa A, Velo E, Papadimitriou E, Cahani G, Kota M, et al. (2009) Ecology of the Crimean-Congo hemorrhagic fever endemic area in Albania. Vector borne and zoonotic diseases (Larchmont, NY) 9: 713-716.
54. Qing T, Saijo M, Lei H, Niikura M, Maeda A, et al. (2003) Detection of immunoglobulin G to Crimean-Congo hemorrhagic fever virus in sheep sera by recombinant nucleoprotein-based enzyme-linked immunosorbent and immunofluorescence assays. Journal of virological methods 108: 111-116.
55. Raileanu C, Anita A, Porea D, Savuta G (2015) Serologic evidence of Crimean-Congo haemorrhagic fever infection in small ruminants in Southeastern Romania. Epidemiologie et Sante Animale 67: 145-149.
56. Rezazadeh F, Chinikar S, Bageri Amiri F (2012) Aseroprevalance survey of anti-CCHFV igg by ELISA in sheep from some area in northwest of Iran. Global Veterinaria 9: 655-658.
57. Saghafipour A, Mousazadeh-Mojarrad A, Arzamani N, Telmadarraiy Z, Rajabzadeh R, et al. (2019) Molecular and seroepidemiological survey on Crimean-Congo Hemorrhagic Fever Virus in Northeast of Iran. Medical journal of the Islamic Republic of Iran 33: 41-41.
58. Saidi S, Casals J, Faghih MA (1975) Crimean hemorrhagic fever-Congo (CHF-C) virus antibodies in man, and in domestic and small mammals, in Iran. The American journal of tropical medicine and hygiene 24: 353-357.
59. Sas MA, Mertens M, Isselmou E, Reimer N, El Mamy BO, et al. (2017) Crimean-Congo Hemorrhagic Fever Virus-Specific Antibody Detection in Cattle in Mauritania. Vector borne and zoonotic diseases (Larchmont, NY) 17: 582-587.
60. Sas MA, Mertens M, Kadiat JG, Schuster I, Pongombo CPS, et al. (2017) Serosurvey for Crimean-Congo hemorrhagic fever virus infections in ruminants in Katanga province, Democratic Republic of the Congo. Ticks and tick-borne diseases 8: 858-861.
61. Schuster I, Chaintoutis SC, Dovas CI, Groschup MH, Mertens M (2017) Detection of Crimean-Congo hemorrhagic fever virus-specific IgG antibodies in ruminants residing in Central and Western Macedonia, Greece. Ticks and tick-borne diseases 8: 494-498.
62. Shepherd AJ, Swanepoel R, Leman PA, Shepherd SP (1987) Field and laboratory investigation of Crimean-Congo haemorrhagic fever virus (Nairovirus, family Bunyaviridae) infection in birds. Transactions of the Royal Society of Tropical Medicine and Hygiene 81: 1004-1007.
63. Shepherd AJ, Swanepoel R, Shepherd SP, McGillivray GM, Searle LA (1987) Antibody to Crimean-Congo hemorrhagic fever virus in wild mammals from southern Africa. The American journal of tropical medicine and hygiene 36: 133-142.
64. Sherifi K, Rexhepi A, Robaj A, Hamidi A, Behluli B, et al. (2016) A survey of crimean-congo hemorrhagic fever in livestock in republic of kosova. Kafkas Universitesi Veteriner Fakultesi Dergisi 22: 301-304.
65. Shrivastava N, Shrivastava A, Ninawe SM, Sharma S, Kumar JS, et al. (2019) Development of Multispecies Recombinant Nucleoprotein-Based Indirect ELISA for High-Throughput Screening of Crimean-Congo Hemorrhagic Fever Virus-Specific Antibodies. Frontiers in microbiology 10: 1822-1822.
66. Suliman HM, Adam IA, Saeed SI, Abdelaziz SA, Haroun EM, et al. (2017) Crimean Congo hemorrhagic fever among the one-humped camel (Camelus dromedaries) in Central Sudan. Virology journal 14: 147-147.
67. Sun S, Dai X, Aishan M, Wang X, Meng W, et al. (2009) Epidemiology and phylogenetic analysis of crimean-congo hemorrhagic fever viruses in xinjiang, china. Journal of clinical microbiology 47: 2536-2543.
68. Swanepoel R, Shepherd AJ, Leman PA, Shepherd SP (1985) Investigations following initial recognition of Crimean-Congo haemorrhagic fever in South Africa and the diagnosis of 2

further cases. South African medical journal = Suid-Afrikaanse tydskrif vir geneeskunde 68: 638-641.
69. Swanepoel R, Shepherd AJ, Leman PA, Shepherd SP, McGillivray GM, et al. (1987) Epidemiologic and clinical features of Crimean-Congo hemorrhagic fever in southern Africa. The American journal of tropical medicine and hygiene 36: 120-132.
70. Tantawi HH, Shony MO, Al-Tikriti SK (1981) Antibodies to Crimean-Congo haemorrhagic fever virus in domestic animals in Iraq: a seroepidemiological survey. International journal of zoonoses 8: 115-120.
71. Telmadarraiy Z, Ghiasi SM, Moradi M, Vatandoost H, Eshraghian MR, et al. (2010) A survey of Crimean-Congo haemorrhagic fever in livestock and ticks in Ardabil Province, Iran during 2004-2005. Scandinavian journal of infectious diseases 42: 137-141.
72. Telmadarraiy Z, Moradi AR, Vatandoost R, Mostafavi E, Oshaghi MA, et al. (2008) Crimean-congo hemorrhagic fever: A seroepidemiological and molecular survey in Bahar, Hamadan province of Iran. Asian Journal of Animal and Veterinary Advances 3: 321-327.
73. Umoh JU, Ezeokoli CD, Ogwu D (1983) Prevalence of antibodies to Crimean-haemorrhagic fever-Congo virus in cattle in northern Nigeria. International journal of zoonoses 10: 151-154.
74. Wangchuk S, Pelden S, Dorji T, Tenzin S, Thapa B, et al. (2016) Crimean-Congo Hemorrhagic Fever Virus IgG in Goats, Bhutan. Emerging infectious diseases 22: 919-920.
75. Williams RJ, Al-Busaidy S, Mehta FR, Maupin GO, Wagoner KD, et al. (2000) Crimean-congo haemorrhagic fever: a seroepidemiological and tick survey in the Sultanate of Oman. Tropical medicine & international health : TM & IH 5: 99-106.
76. Wilson ML, LeGuenno B, Guillaud M, Desoutter D, Gonzalez JP, et al. (1990) Distribution of Crimean-Congo hemorrhagic fever viral antibody in Senegal: environmental and vectorial correlates. The American journal of tropical medicine and hygiene 43: 557-566.
77. Yadav PD, Gurav YK, Mistry M, Shete AM, Sarkale P, et al. (2014) Emergence of Crimean-Congo hemorrhagic fever in Amreli District of Gujarat State, India, June to July 2013. International journal of infectious diseases : IJID : official publication of the International Society for Infectious Diseases 18: 97-100.
78. Zeller HG, Cornet JP, Camicas JL (1994) Crimean-Congo haemorrhagic fever virus infection in birds: field investigations in Senegal. Research in virology 145: 105-109.
