# Supplementary material for: Worldwide epidemiology of Crimean-Congo Hemorrhagic Fever Virus in humans, ticks and other animal species, a systematic review and meta-analysis
Source: PLoS Negl Trop Dis. 2021 Apr 22;15(4):e0009299. doi: 10.1371/journal.pntd.0009299 (PMC8096040; doi:10.1371/journal.pntd.0009299)
Supplement: S2 Table — (PDF) [file pntd.0009299.s006.pdf]

S2 Table. Search strategy in Medline (Pubmed)

**Search**	**Virus**
#1	Hemorrhagic Fever Virus, Crimean-Congo OR Hemorrhagic Fever, Crimean OR Crimean Congo Hemorrhagic Fever OR Crimean Congo Hemorrhagic Fever Virus CCHF OR CCHFV
