# Supplementary material for: Worldwide epidemiology of Crimean-Congo Hemorrhagic Fever Virus in humans, ticks and other animal species, a systematic review and meta-analysis
Source: PLoS Negl Trop Dis. 2021 Apr 22;15(4):e0009299. doi: 10.1371/journal.pntd.0009299 (PMC8096040; doi:10.1371/journal.pntd.0009299)
Supplement: S4 Table — (PDF) [file pntd.0009299.s008.pdf]

S4 Table. Main reasons of exclusion of eligible studies

N°	Author, Year	Title	Reason of exclusion
1	Abass, 2015	Spatial cluster analysis of human cases of Crimean Congo hemorrhagic fever reported in Pakistan.	No data on CCHFV prevalence or case fatality rate
2	Abbas, 2017	Seasonality in hospital admissions of Crimean-Congo hemorrhagic fever and its dependence on ambient temperature-empirical evidence from Pakistan.	No data on CCHFV prevalence or case fatality rate
3	Abdelhakam, 2014	Crimean-Congo hemorrhagic fever (CCHF) in Southern Kordofan.	Case report
4	Aghamali, 2017	Summer crisis in Iran: increase in reported cases of Crimean-Congo Hemorrhagic Fever (CCHF).	No data on CCHFV prevalence or case fatality rate
5	Ahmadkhani, 2018	Space-time epidemiology of Crimean-Congo hemorrhagic fever (CCHF) in Iran.	No data on CCHFV prevalence or case fatality rate
6	Akinci, 2016	Prognostic factors, pathophysiology and novel biomarkers in Crimean-Congo hemorrhagic fever.	Review
7	Al-Abri, 2019	Clinical and molecular epidemiology of Crimean-Congo hemorrhagic fever in Oman.	Not possible to extract data on CCHFV prevalence or case fatality rate
8	Alavi-Naini, 2006	Crimean-Congo hemorrhagic fever in Southeast of Iran.	No data on CCHFV prevalence or case fatality rate
9	Algaar, 2015	Fiber-optic immunosensor for detection of Crimean-Congo hemorrhagic fever IgG antibodies in patients.	No data on CCHFV prevalence or case fatality rate
10	Al-Tikriti, 1981	Congo/Crimean haemorrhagic fever in Iraq.	Sample size < or = 10 participants
11	Andriamandimby, 2011	Crimean-Congo hemorrhagic fever serosurvey in at-risk professionals, Madagascar, 2008 and 2009.	Duplicate study
12	Aradaib, 2010	Nosocomial outbreak of Crimean-Congo hemorrhagic fever, Sudan.	Sample size < or = 10 participants
13	Aradaib, 2011	Multiple Crimean-Congo hemorrhagic fever virus strains are associated with disease outbreaks in Sudan, 2008-2009.	Sample size < or = 10 participants
14	Athar, 2003	Short report: Crimean-Congo hemorrhagic fever outbreak in Rawalpindi, Pakistan, February 2002.	Case report
15	Atkinson, 2013	Identification and analysis of Crimean-Congo hemorrhagic fever virus from human sera in Tajikistan.	No data on CCHFV prevalence or case fatality rate
16	Atkinson, 2016	Plant-produced Crimean-Congo haemorrhagic fever virus nucleoprotein for use in indirect ELISA.	No data on CCHFV prevalence or case fatality rate
17	Balinandi, 2018	Investigation of an isolated case of human Crimean–Congo hemorrhagic fever in Central Uganda, 2015.	Duplicate study
18	Baniasadi, 2019	Evaluation of first rapid diagnostic kit for Anti-Crimean-Congo Hemorrhagic Fever virus IgM antibody using clinical samples from Iran.	No data on CCHFV prevalence or case fatality rate
19	Barr, 2013	First confirmed case of Crimean-Congo haemorrhagic fever in the UK.	Case report
20	Bayram, 2017	Seroprevalence of crimean-congo hemorrhagic fever in Turkey's Van Province.	Duplicate study
21	Bazanów, 2017	Vector and Serologic Survey for Crimean-Congo Hemorrhagic Fever Virus in Poland.	Duplicate study

22	Binder, 2019	Molecular survey of flaviviruses and orthobunyaviruses in Amblyomma spp. ticks collected in Minas Gerais, Brazil.	No data on CCHFV prevalence or case fatality rate
23	Bodur, 2010	Detection of Crimean-Congo hemorrhagic fever virus genome in saliva and urine.	Duplicate study
24	Bodur, 2010	Detection of Crimean-Congo hemorrhagic fever virus genome in saliva and urine.	No data on CCHFV prevalence or case fatality rate
25	Bonney, 2017	A recombinase polymerase amplification assay for rapid detection of Crimean-Congo Haemorrhagic fever Virus infection.	No data on CCHFV prevalence or case fatality rate
26	Burney, 1980	Nosocomial outbreak of viral hemorrhagic fever caused by Crimean Hemorrhagic fever-Congo virus in Pakistan, January 1976.	Not possible to extract data on CCHFV prevalence or case fatality rate
27	Bursali, 2013	Ticks (Acari: Ixodida) infesting humans in the provinces of Kelkit Valley, a Crimean-Congo Hemorrhagic Fever endemic region in Turkey.	Not possible to extract data on CCHFV prevalence or case fatality rate
28	Burt, 1998	The use of a reverse transcription-polymerase chain reaction for the detection of viral nucleic acid in the diagnosis of Crimean-Congo haemorrhagic fever.	Samples with already known result
29	Burt, 2005	Molecular epidemiology of African and Asian Crimean-Congo haemorrhagic fever isolates.	No data on CCHFV prevalence or case fatality rate
30	Burt, 2007	Crimean-Congo hemorrhagic fever in South Africa.	Report
31	Burt, 2009	Genetic relationship in southern African Crimean-Congo haemorrhagic fever virus isolates: evidence for occurrence of reassortment.	Not possible to extract data on CCHFV prevalence or case fatality rate
32	Burt, 2013	Human defined antigenic region on the nucleoprotein of Crimean-Congo hemorrhagic fever virus identified using truncated proteins and a bioinformatics approach.	No data on CCHFV prevalence or case fatality rate
33	Camicas, 1980	Tick-borne viruses in tropical area (author's transl).	Review
34	Canakoglu, 2013	Pseudo-plaque reduction neutralization test (PPRNT) for the measurement of neutralizing antibodies to Crimean-Congo hemorrhagic fever virus.	Experimental infection
35	Centers for Disease , 1981	Crimean-Congo hemorrhagic fever--South Africa.	Case report
36	Centers for Disease , 2007	Rift Valley fever outbreak--Kenya, November 2006-January 2007.	Case report
37	Centers for Disease, 1984	Congo-Crimean hemorrhagic fever--Republic of South Africa.	Case report
38	Centers for Disease, 1985	Crimean-Congo hemorrhagic fever--Republic of South Africa.	Sample size < or = 10 participants
39	Cevik, 2008	Clinical and laboratory features of Crimean-Congo hemorrhagic fever: predictors of fatality.	Duplicate study
40	Chamberlain , 2013	Genome sequence of ex-Afghanistan Crimean-Congo hemorrhagic fever virus SCT strain, from an imported United Kingdom case in October 2012.	Case report
41	Chamberlain , 2013	Genome Sequence of Ex-Afghanistan Crimean-Congo Hemorrhagic Fever Virus SCT Strain, from an Imported United Kingdom Case in October 2012.	Duplicate study
42	Champour , 2016	Molecular epidemiology of Crimean–Congo hemorrhagic fever virus detected from ticks of one humped camels (Camelus dromedarius) population in northeastern Iran.	Duplicate study
43	Champour, 2016	Crimean-Congo Hemorrhagic Fever in the One-Humped Camel (Camelus dromedarius) in East and Northeast of Iran.	Duplicate study

44	CHEN, 2013	Molecular evolution of Crimean-Congo hemorrhagic fever virus based on complete genomes.	No data on CCHFV prevalence or case fatality rate
45	Chinikar, 2010	Phylogenetic analysis in a recent controlled outbreak of Crimean-Congo haemorrhagic fever in the south of Iran, December 2008.	Duplicate study
46	Chinikar, 2010	Crimean-Congo hemorrhagic fever in Iran and neighboring countries.	Review
47	Chinikar, 2010	Phylogenetic analysis in a recent controlled outbreak of Crimean-Congo haemorrhagic fever in the south of Iran, December 2008.	Sample size < or = 10 participants
48	Chinikar, 2013	New circulating genomic variant of Crimean-Congo hemorrhagic fever virus in Iran.	Report
49	CHINIKAR, 2016	Genetic Diversity of Crimean Congo Hemorrhagic Fever Virus Strains from Iran.	No data on CCHFV prevalence or case fatality rate
50	Chinikar, 2016	Genetic analysis of Crimean-congo hemorrhagic fever virus in Iran.	No data on CCHFV prevalence or case fatality rate
51	Chitimia-Dobler, 2016	First detection of Hyalomma rufipes in Germany.	No data on CCHFV prevalence or case fatality rate
52	Chitimia-Dobler, 2019	Crimean-Congo haemorrhagic fever virus in Hyalomma impeltatum ticks from North Kordofan, the Sudan.	Not possible to extract data on CCHFV prevalence or case fatality rate
53	Christova, 2009	Crimean-Congo hemorrhagic fever, southwestern Bulgaria.	Case report
54	Christova, 2018	High seroprevalence for Crimean–Congo haemorrhagic fever virus in ruminants in the absence of reported human cases in many regions of Bulgaria.	Duplicate study
55	Cicek-Sentürk, 2018	Retrospective investigation of 9 years of data on needlestick and sharps injuries: Effect of a hospital infection control committee.	No data on CCHFV prevalence or case fatality rate
56	Coffey, 2014	Enhanced arbovirus surveillance with deep sequencing: Identification of novel rhabdoviruses and bunyaviruses in Australian mosquitoes.	No data on CCHFV prevalence or case fatality rate
57	Conger, 2015	Health care response to CCHF in US soldier and nosocomial transmission to health care providers, Germany, 2009.	Case report
58	Cope, 1996	Assessment of arthropod vectors of infectious diseases in areas of U.S. troop deployment in the Persian Gulf.	No data on CCHFV prevalence or case fatality rate
59	Cornet, 2002	Input to the study of ticks (Acarina: Ixodina) vectors of Crimean-Congo hemorrhagic fever virus, in Senegal: 4-release and reuptake of Hyalomma truncatum Koch 1844.	No full text
60	Daldal, 2012	Crimean-Congo haemorrhagic fever: The first case in Şanliurfa'da, Turkey.	Case report
61	Darwish, 1977	A seroepidemiological survey for Crimean-Congo hemorrhagic fever virus in humans and domestic animals in Egypt.	No full text
62	Darwish, 1978	Results of a preliminary seroepidemiological survey for Crimean-Congo hemorrhagic fever virus in Egypt.	No full text
63	Davies , 1978	The serological relationships of Nairobi sheep disease virus.	No data on CCHFV prevalence or case fatality rate
64	de St Maurice, 2016	Notes from the Field: Rift Valley Fever Response - Kabale District, Uganda, March 2016.	No data on CCHFV prevalence or case fatality rate

65	Dinçer, 2017	Generic amplification and next generation sequencing reveal Crimean-Congo hemorrhagic fever virus AP92-like strain and distinct tick phleboviruses in Anatolia, Turkey.	Duplicate study
66	Drosten, 2002	Crimean-Congo hemorrhagic fever in Kosovo.	Case report
67	Drosten, 2002	Rapid detection and quantification of RNA of Ebola and Marburg viruses, Lassa virus, Crimean-Congo hemorrhagic fever virus, Rift Valley fever virus, dengue virus, and yellow fever virus by real-time reverse transcription-PCR.	No data on CCHFV prevalence or case fatality rate
68	Duh, 2006	Novel one-step real-time RT-PCR assay for rapid and specific diagnosis of Crimean-Congo hemorrhagic fever encountered in the Balkans.	No data on CCHFV prevalence or case fatality rate
69	Dunster, 2002	First documentation of human Crimean-Congo hemorrhagic fever, Kenya.	Case report
70	Duran, 2013	Evaluation of patients with Crimean-Congo hemorrhagic fever in Bolu, Turkey.	Duplicate study
71	Duran, 2013	Evaluation of patients with Crimean-Congo hemorrhagic fever in Bolu, Turkey.	Not possible to extract data on CCHFV prevalence or case fatality rate
72	Durrani, 2007	Congo crimean hemorrhagic Fever in balochistan.	No full text
73	Duygu, 2018	Cutaneous Findings of Crimean-Congo Hemorrhagic Fever: a Study of 269 Cases.	No data on CCHFV prevalence or case fatality rate
74	Duyzu, 2017	Brucellosis in Patients with Crimean-Congo Hemorrhagic Fever.	No data on CCHFV prevalence or case fatality rate
75	Eeden , 1985	A nosocomial outbreak of Crimean-Congo haemorrhagic fever at Tygerberg Hospital. Part II. Management of patients.	Case report
76	Elata, 2011	A nosocomial transmission of crimean-congo hemorrhagic fever to an attending physician in North Kordufan, Sudan.	Case report
77	El-Azazy, 1997	Crimean-Congo haemorrhagic fever virus infection in the Western Province of Saudi Arabia.	Duplicate study
78	Emmerich, 2010	Early serodiagnosis of acute human Crimean-Congo hemorrhagic fever virus infections by novel capture assays.	No data on CCHFV prevalence or case fatality rate
79	Emmerich, 2018	Viral metagenomics, genetic and evolutionary characteristics of Crimean-Congo hemorrhagic fever orthonairovirus in humans, Kosovo.	No data on CCHFV prevalence or case fatality rate
80	Emmerich, 2018	Sensitive and specific detection of Crimean-Congo Hemorrhagic Fever Virus (CCHFV)—Specific IgM and IgG antibodies in human sera using recombinant CCHFV nucleoprotein as antigen in μ-capture and IgG immune complex (IC) ELISA tests.	Samples with already known result
81	Ergonul, 2006	Analysis of risk-factors among patients with Crimean-Congo haemorrhagic fever virus infection: Severity criteria revisited.	Duplicate study
82	Ergönül, 2006	Zoonotic infections among veterinarians in Turkey: Crimean-Congo hemorrhagic fever and beyond.	Duplicate study
83	Ergonul, 2007	Clinical and pathologic features of Crimean-Congo hemorrhagic fever.	Review
84	Ertürk, 2017	Serosurvey of coxiella burnetii in high risk population in turkey, endemic to crimean-congo haemorrhagic fever virus.	Duplicate study
85	Ertürk, 2017	Serosurvey of Coxiella burnetii in high risk population in Turkey, endemic to Crimean-Congo haemorrhagic fever virus.	No data on CCHFV prevalence or case fatality rate

86	Esen, 2008	The presence of tick-borne encephalitis in an endemic area for tick-borne diseases, Turkey.	No data on CCHFV prevalence or case fatality rate
87	Fajfr, 2014	Detection panel for identification of twelve hemorrhagic viruses using real-time RT-PCR.	No data on CCHFV prevalence or case fatality rate
88	Fajs, 2014	Molecular epidemiology of Crimean-Congo hemorrhagic fever virus in Kosovo.	No data on CCHFV prevalence or case fatality rate
89	Filippone, 2013	Molecular diagnostic and genetic characterization of highly pathogenic viruses: application during Crimean-Congo haemorrhagic fever virus outbreaks in Eastern Europe and the Middle East.	No data on CCHFV prevalence or case fatality rate
90	Fisher-Hoch, 1992	Risk of human infections with Crimean-Congo hemorrhagic fever virus in a South African rural community.	Duplicate study
91	Fletcher, 2017	Infection prevention and control practice for Crimean-Congo hemorrhagic fever-A multi-center cross-sectional survey in Eurasia.	No data on CCHFV prevalence or case fatality rate
92	Flick, 2007	Molecular biology of the Crimean-Congo hemorrhagic fever virus.	Review
93	Fontenille, 1988	Hemorrhagic fever viruses in Madagascar.	No full text
94	Fontenille, 1989	Arbovirus transmission cycles in Madagascar.	No full text
95	Fontenille, 1989	Transmission cycles of arboviruses in Madagascar.	No full text
96	Gandhi, 2011	An epidemiological investigation of a multisource outbreak of crimean-congo hemorrhagic fever in Gujarat.	Duplicate study
97	García Rada, 2016	First outbreak of Crimean-Congo haemorrhagic fever in western Europe kills one man in Spain.	Case report
98	Garcia, 2006	Evaluation of a Crimean-Congo hemorrhagic fever virus recombinant antigen expressed by Semliki Forest suicide virus for IgM and IgG antibody detection in human and animal sera collected in Iran.	No data on CCHFV prevalence or case fatality rate
99	Gazi, 2016	Seroprevalence of west nile virus, crimean-congo hemorrhagic fever virus, francisella tularensis and borrelia burgdorferi in rural population of Manisa, Western Turkey.	Duplicate study
100	Gordon, 1993	Transmission of Crimean-Congo hemorrhagic fever virus in two species of Hyalomma ticks from infected adults to cofeeding immature forms.	Experimental infection
101	Gozalan, 2007	Crimean-congo haemorrhagic fever cases in Turkey.	Duplicate study
102	Gozel, 2014	Favorable outcomes for both mother and baby are possible in pregnant women with Crimean-Congo hemorrhagic fever disease: a case series and literature review.	Case report
103	Grard, 2011	Re-emergence of Crimean-Congo hemorrhagic fever virus in Central Africa.	No data on CCHFV prevalence or case fatality rate
104	Gül, 2011	Cardiac findings in children with Crimean-Congo hemorrhagic fever.	No data on CCHFV prevalence or case fatality rate
105	Güneş, 2012	The seroprevalence of Rickettsia conorii in humans living in villages of Tokat Province in Turkey, where Crimean-Congo hemorrhagic fever virus is endemic, and epidemiological similarities of both infectious agents.	No data on CCHFV prevalence or case fatality rate
106	Guo, 2017	A new strain of Crimean-Congo hemorrhagic fever virus isolated from Xinjiang, China.	No data on CCHFV prevalence or case fatality rate
107	Guven, 2017	An Unexpected Fatal CCHF Case and Management of Exposed Health Care Workers.	Case report

108	Haider, 2016	Crimean-Congo haemorrhagic fever in Pakistan.	Report
109	Hewson, 2007	Molecular epidemiology, genomics, and phylogeny of Crimean-Congo hemorrhagic fever virus.	No data on CCHFV prevalence or case fatality rate
110	Horváth, 1975	Incidence of antibodies to Crimean haemorrhagic fever in animals (author's transl).	No full text
111	Humolli, 2010	Epidemiological, serological and herd immunity of Crimean-Congo haemorrhagic fever in Kosovo.	No data on CCHFV prevalence or case fatality rate
112	Ibrahim, 2011	Detection of Crimean-Congo hemorrhagic fever, Hanta, and sandfly fever viruses by real-time RT-PCR.	No data on CCHFV prevalence or case fatality rate
113	Ijaz, 2017	Crimean-Congo Hemorrhagic Fever Virus in Pakistan.	No full text
114	Ince, 2014	Crimean-Congo hemorrhagic fever infections reported by ProMED.	Report
115	İnci, 2015	The prevalence of crimean-congo haemorrhagic fever and the significance of geographic, climatic features, and tick abundance.	No data on CCHFV prevalence or case fatality rate
116	Izadi, 2004	Crimean-Congo hemorrhagic fever in Sistan and Baluchestan Province of Iran, a case-control study on epidemiological characteristics.	No data on CCHFV prevalence or case fatality rate
117	Jääskeläinen, 2014	Development and evaluation of a real-time RT-qPCR for detection of Crimean-Congo hemorrhagic fever virus representing different genotypes.	Samples with already known result
118	Jabbari, 2006	Crimean-Congo hemorrhagic fever: case series from a medical center in Golestan province, Northeast of Iran (2004).	Case report
119	Jabbari, 2013	Facts about Crimean-Congo hemorrhagic fever and the role of intensive care in treatment and outcome.	No data on CCHFV prevalence or case fatality rate
120	Jamil, 2005	Crimean-Congo hemorrhagic fever: experience at a tertiary care hospital in Karachi, Pakistan.	Case report
121	Jauréguiberry, 2005	Imported Crimean-Congo hemorrhagic Fever.	Case report
122	Joubert, 1985	A nosocomial outbreak of Crimean-Congo haemorrhagic fever at Tygerberg Hospital. Part III. Clinical pathology and pathogenesis.	Case report
123	K.M, 1994	<The> situation of nosocomial infection from patients with congo/crimean hemorrhagic fever in Makkah.	No full text
124	Kadanali, 2009	Epidemiological risk factors for Crimean-Congo hemorrhagic fever patients.	No data on CCHFV prevalence or case fatality rate
125	Kalaycioglu, 2012	Lack of genetic diversity in Crimean-Congo hemorrhagic fever viruses in Turkey: assessment of present and future patterns of disease.	No data on CCHFV prevalence or case fatality rate
126	Kalvatchev, 2008	One step rt-pcr for rapid detection of crimean-congo haemorrhagic fever virus.	No data on CCHFV prevalence or case fatality rate
127	Kalvatchev, 2012	Current state of Crimean-Congo hemorrhagic fever in Bulgaria.	Review
128	Kamboj, 2014	Novel molecular beacon probe-based real-time RT-PCR assay for diagnosis of Crimean-Congo hemorrhagic fever encountered in India.	Samples with already known result
129	Karti, 2004	Crimean-Congo hemorrhagic fever in Turkey.	Sample size < or = 10 participants
130	Karti, 2004	Crimean-Congo hemorrhagic fever in Turkey.	Sample size < or = 10 participants

131	Kaul, 1990	Survey of ticks (Acarina: Ixodidae) for Crimean haemorrhagic fever virus activity in Jammu & Kashmir state, India.	No full text
132	Kaul, 1990	Survey of ticks (Acarina: Ixodidae) for Crimean haemorrhagic fever virus activity in Jammu & Kashmir state, India.	No full text
133	Kaya, 2011	Crimean-Congo hemorrhagic fever disease due to tick bite with very long incubation periods.	Duplicate study
134	Kaya, 2011	Crimean-Congo hemorrhagic fever disease due to tick bite with very long incubation periods.	Not possible to extract data on CCHFV prevalence or case fatality rate
135	Ke, 2011	Colorimetric nucleic acid testing assay for RNA virus detection based on circle-to-circle amplification of padlock probes.	Samples with already known result
136	Khaled, 1996	Detection of Crimean-congo hemorrhagic fever virus antibodies in man and in animals.	Duplicate study
137	Kizito, 2018	Notes from the Field: Crimean-Congo Hemorrhagic Fever Outbreak - Central Uganda, August-September 2017.	No data on CCHFV prevalence or case fatality rate
138	Knust, 2012	Crimean-Congo hemorrhagic fever, Kazakhstan, 2009-2010.	No data on CCHFV prevalence or case fatality rate
139	Koehler, 2018	Sequence Optimized Real-Time Reverse Transcription Polymerase Chain Reaction Assay for Detection of Crimean-Congo Hemorrhagic Fever Virus.	No data on CCHFV prevalence or case fatality rate
140	Kondiah, 2010	A Simple-Probe real-time PCR assay for genotyping reassorted and non-reassorted isolates of Crimean-Congo hemorrhagic fever virus in southern Africa.	No data on CCHFV prevalence or case fatality rate
141	Korkmaz, 2011	Evaluation of the patients applying to the hospital for a tick bite.	No data on CCHFV prevalence or case fatality rate
142	Kubar, 2011	Prompt administration of Crimean-Congo hemorrhagic fever (CCHF) virus hyperimmunoglobulin in patients diagnosed with CCHF and viral load monitorization by reverse transcriptase-PCR.	Experimental infection
143	Kuchuloria, 2014	Short report: Viral hemorrhagic fever cases in the country of Georgia: Acute febrile illness surveillance study results.	Duplicate study
144	Kuchuloria, 2016	Hospital-Based Surveillance for Infectious Etiologies Among Patients with Acute Febrile Illness in Georgia, 2008-2011.	Duplicate study
145	Küfeciler, 2016	Investigation of cases with tick attachment at the emergency department.	No data on CCHFV prevalence or case fatality rate
146	Kulichenko, 2016	A new genetic variant of the Crimean–Congo hemorrhagic fever virus isolated in Crimea.	Duplicate study
147	Kunchev, 2008	Probable cases of Crimean-Congo-haemorrhagic fever in Bulgaria: a preliminary report.	Sample size < or = 10 participants
148	Lani, 2015	First report on the seroprevalence of the Crimean-Congo haemorrhagic fever virus, a tick-borne virus, in Malaysia's Orang Asli population.	Duplicate study
149	Leblebicioglu, 2016	Discharge criteria for Crimean-Congo haemorrhagic fever in endemic areas.	No data on CCHFV prevalence or case fatality rate
150	Leroy, 2008	A retrospective study of 230 consecutive patients hospitalized for presumed travel-related illness (2000-2006).	Not possible to extract data on CCHFV prevalence or case fatality rate

151	Liu, 2016	Development of a TaqMan Array Card for Acute-Febrile-Illness Outbreak Investigation and Surveillance of Emerging Pathogens, Including Ebola Virus.	No data on CCHFV prevalence or case fatality rate
152	Logan, 1993	Antigen-capture enzyme-linked immunosorbent assay for detection and quantification of Crimean-Congo hemorrhagic fever virus in the tick, Hyalomma truncatum.	Experimental infection
153	Lumley, 2015	Non-fatal case of crimean-congo haemorrhagic fever imported into the United Kingdom (Ex Bulgaria), June 2014.	Case report
154	Makwana, 2015	First confirmed case of Crimean-Congo haemorrhagic fever from Sirohi district in Rajasthan State, India.	Case report
155	Mallhi, 2016	Crimean-Congo haemorrhagic fever virus and Eid-Ul-Adha festival in Pakistan.	No data on CCHFV prevalence or case fatality rate
156	Mallhi, 2017	Commentary: Surveillance of Crimean-Congo Haemorrhagic Fever in Pakistan.	Comment on an article
157	Mamuchishvili, 2015	Notes from the field: Increase in reported Crimean-Congo hemorrhagic fever cases--country of Georgia, 2014.	No data on CCHFV prevalence or case fatality rate
158	Mancini, 2013	Close contact precautions could prevent an outbreak of crimean-congo hemorrhagic Fever: a case series report from southern part of tehran.	Case report
159	Mardani, 2007	Crimean-Congo hemorrhagic fever among health care workers in Iran: a seroprevalence study in two endemic regions.	Duplicate study
160	Mardani, 2009	Short report: Crimean-Congo hemorrhagic fever virus as a nosocomial pathogen in Iran.	Duplicate study
161	Mardani, 2009	Crimean-Congo hemorrhagic fever virus as a nosocomial pathogen in Iran.	Report
162	Marriott, 1994	Detection of human antibodies to Crimean-Congo haemorrhagic fever virus using expressed viral nucleocapsid protein.	No data on CCHFV prevalence or case fatality rate
163	Masroor Alam, 2013	Crimean-congo hemorrhagic fever Asia-2 genotype, Pakistan.	Samples with already known result
164	McMullan, 2012	Using next generation sequencing to identify yellow fever virus in Uganda.	No data on CCHFV prevalence or case fatality rate
165	Mehrabi-Tavana, 2002	The seroepidemiological aspects of Crimean-Congo hemorrhagic fever in three health workers: A report from Iran.	Case report
166	Mehran, 2016	Molecular epidemiology of Crimean-Congo hemorrhagic fever virus in ticks collected from western Iran.	Not possible to extract data on CCHFV prevalence or case fatality rate
167	Mertens, 2015	Circulation of Crimean-Congo Hemorrhagic Fever Virus in the Former Yugoslav Republic of Macedonia Revealed by Screening of Cattle Sera Using a Novel Enzyme-linked Immunosorbent Assay.	Duplicate study
168	Mertens, 2015	Circulation of Crimean-Congo Hemorrhagic Fever Virus in the former Yugoslav Republic of Macedonia revealed by screening of cattle sera using a novel enzyme-linked immunosorbent assay.	Sample with already known result
169	Metanat, 2006	Clinical outcomes in Crimean-Congo Hemorrhagic Fever: A five-years experience in the treatment of patients in oral Ribavirin.	Laboratory assays used unclear or not reported
170	Metanat, 2006	The epidemiological aspect of Crimean-congo hemorrhagic fever in Southeast of Iran.	No data on CCHFV prevalence or case fatality rate

171	Metanat, 2018	Report of a family with crimean-congo hemorrhagic fever following contact with frozen meat: A case seires study.	Sample size < or = 10 participants
172	Midilli, 2007	Imported Crimean-Congo hemorrhagic fever cases in Istanbul.	Duplicate study
173	Midilli, 2009	The first clinical case due to AP92 like strain of Crimean-Congo Hemorrhagic Fever virus and a field survey.	Duplicate study
174	Milutinovic, 1997	Ecological investigations on ticks (Acari, Ixodidae) of East Serbia, with emphasis on Ixodes ricinus and Hyalomma savignyi.	No data on CCHFV prevalence or case fatality rate
175	Mishra, 2011	Crimean-Congo haemorrhagic fever in India.	Case report
176	Mofleh, 2012	Crimean-Congo haemorrhagic fever outbreak investigation in the Western Region of Afghanistan in 2008.	Duplicate study
177	Mohamed, 2017	Investigation of hemorrhagic fever viruses inside wild populations of ticks: One of the pioneer studies in Saudi Arabia.	Not possible to extract data on CCHFV prevalence or case fatality rate
178	Monlun, 1993	Surveillance of the circulation of arbovirus of medical interest in the region of eastern Senegal.	No full text
179	Morikawa, 2002	Genetic diversity of the M RNA segment among Crimean-Congo hemorrhagic fever virus isolates in China.	No data on CCHFV prevalence or case fatality rate
180	Morrill, 1990	Serological evidence of Crimean-Congo haemorrhagic fever viral infection among camels imported into Egypt.	Duplicate study
181	Morteza, 2010	Nosocomial transmission of Crimean-Congo hemorrhagic fever in a health care worker, Fars Province, Iran.	No full text
182	Mourya, 2015	Cross-sectional serosurvey of crimean-congo hemorrhagic fever virus IgG in livestock, India, 2013–2014.	Duplicate study
183	Nabeth, 2004	Human Crimean-Congo hemorrhagic fever, Sénégal.	Case report
184	Naderi, 2011	Nosocomial outbreak of Crimean-Congo haemorrhagic fever.	Case report
185	Negredo, 2019	Survey of Crimean-Congo Hemorrhagic Fever Enzootic Focus, Spain, 2011-2015.	Duplicate study
186	No author listed, 2010	Outbreak news. Cholera, Haiti, cholera, Pakistan, Crimean-Congo haemorrhagic fever (CCHF) and dengue fever, Pakistan.	No data on CCHFV prevalence or case fatality rate
187	Nurmakhanov, 2015	Crimean-Congo haemorrhagic fever virus in Kazakhstan (1948-2013).	Report
188	Nyataya, 2020	Serological Evidence of Yersiniosis, Tick-Borne Encephalitis, West Nile, Hepatitis E, Crimean-Congo Hemorrhagic Fever, Lyme Borreliosis, and Brucellosis in Febrile Patients Presenting at Diverse Hospitals in Kenya.	Not possible to extract data on CCHFV prevalence or case fatality rate
189	Oehme, 2017	Hyalomma marginatum in Tübingen, Germany.	No data on CCHFV prevalence or case fatality rate
190	Office, 2013	Crimean-Congo haemorrhagic fever [CCHF] in Pakistan.	No data on CCHFV prevalence or case fatality rate
191	Olschläger, 2011	Complete sequence and phylogenetic characterisation of Crimean-Congo hemorrhagic fever virus from Afghanistan.	No data on CCHFV prevalence or case fatality rate
192	Osman, 2013	Development and evaluation of loop-mediated isothermal amplification assay for detection of Crimean Congo hemorrhagic fever virus in Sudan.	No data on CCHFV prevalence or case fatality rate
193	Owaysee Osquee, 2017	Laboratory features of 160 CCHF confirmed cases in Zabol of Iran: A 10-year study.	No data on CCHFV prevalence or case fatality rate
194	Ozbey, 2014	Early use of ribavirin is beneficial in Crimean-Congo hemorrhagic fever.	Duplicate study

195	Ozkaya, 2010	Molecular epidemiology of Crimean-Congo hemorrhagic fever virus in Turkey: occurrence of local topotype.	No data on CCHFV prevalence or case fatality rate
196	Ozkurt, 2011	Prognostic significance of antithrombin activity in patients with crimean-congo hemorrhagic Fever.	No data on CCHFV prevalence or case fatality rate
197	Palomar, 2013	Crimean-Congo hemorrhagic fever virus in ticks from migratory birds, Morocco.	Sample size < or = 10 participants
198	Palomar, 2016	Molecular analysis of Crimean-Congo hemorrhagic fever virus and Rickettsia in Hyalomma marginatum ticks removed from patients (Spain) and birds (Spain and Morocco), 2009–2015.	Duplicate study
199	Palomar, 2017	Molecular (ticks) and serological (humans) study of Crimean-Congo hemorrhagic fever virus in the Iberian Peninsula, 2013–2015.	Duplicate study
200	Papa, 2002	Genetic detection and isolation of crimean-congo hemorrhagic fever virus, Kosovo, Yugoslavia.	Case report
201	Papa, 2002	Crimean-Congo hemorrhagic fever in Albania, 2001.	Sample size < or = 10 participants
202	Papa, 2004	Crimean-Congo hemorrhagic fever in Bulgaria.	No data on CCHFV prevalence or case fatality rate
203	Papa, 2005	Genetic characterization of the M RNA segment of a Balkan Crimean-Congo hemorrhagic fever virus strain.	No data on CCHFV prevalence or case fatality rate
204	Papa, 2010	Emergence of Crimean-Congo haemorrhagic fever in Greece.	Case report
205	Papa, 2011	Ticks parasitizing humans in Greece.	No data on CCHFV prevalence or case fatality rate
206	Papa, 2011	Crimean-Congo hemorrhagic fever virus, northeastern Greece.	Review
207	Papa, 2014	Crimean-Congo hemorrhagic fever virus, Greece.	No data on CCHFV prevalence or case fatality rate
208	Papa, 2016	Molecular epidemiology of Crimean-Congo hemorrhagic fever in Bulgaria-An update.	Duplicate study
209	Papa, 2016	Molecular epidemiology of Crimean-Congo hemorrhagic fever in Bulgaria--An update.	No data on CCHFV prevalence or case fatality rate
210	Papa, 2017	Molecular detection of Crimean-Congo hemorrhagic fever virus in ticks, Greece, 2012–2014.	Duplicate study
211	Papa, 2018	Crimean-Congo haemorrhagic fever in a Greek worker returning from Bulgaria, June 2018.	Case report
212	Patel, 2011	First Crimean-Congo hemorrhagic fever outbreak in India.	Sample size < or = 10 participants
213	Petrova, 2013	Genetic variants of the Crimean-Congo hemorrhagic fever virus circulating in endemic areas of southern Tajikistan in 2009.	Study not in english or french language
214	Pourahmad, 2011	Nosocomial transmission of Crimean-Congo hemorrhagic fever in a health care worker, Fars province, Iran.	Case report
215	Pshenichnaya, 2015	Probable Crimean-Congo hemorrhagic fever virus transmission occurred after aerosol-generating medical procedures in Russia: nosocomial cluster.	Sample size < or = 10 participants
216	Rai, 2008	Crimean-Congo hemorrhagic fever in Pakistan.	Case report
217	Rangunwala, 2014	Detection of IgG antibody against Crimean-Congo haemorrhagic fever virus using ELISA with recombinant nucleoprotein antigens from genetically diverse strains.	Report

218	Rehman, 2014	Eid-ul-Azha festival in Pakistan: a vulnerable time for Crimean-Congo hemorrhagic fever outbreak.	No data on CCHFV prevalence or case fatality rate
219	Rehman, 2018	Outbreak of Crimean-Congo haemorrhagic fever with atypical clinical presentation in the Karak District of Khyber Pakhtunkhwa, Pakistan.	Case report
220	Rezazadeh, 2013	Seroprevalance survey of anti-CCHFV IgG by ELISA in sheep from some area in Northwest of Iran.	Duplicate study
221	Rodrigues, 1986	Prevalence of Crimean haemorrhagic fever--Congo virus in Jammu & Kashmir state.	Duplicate study
222	Rodrigues, 1986	Prevalence of Crimean haemorrhagic fever--Congo virus in Jammu & Kashmir state.	No full text
223	Rodrigues, 2011	Development of a one step real time RT-PCR assay to detect and quantify Dugbe virus.	No data on CCHFV prevalence or case fatality rate
224	Sağmak Tartar, 2019	Crimean Congo Hemorrhagic Fever in Eastern Turkey: Epidemiological and Clinical Evaluation.	Duplicate study
225	Sağmak Tartar, 2019	Crimean Congo Hemorrhagic Fever in Eastern Turkey: Epidemiological and Clinical Evaluation.	Study not in english or french language
226	Saijo, 2002	Recombinant nucleoprotein-based enzyme-linked immunosorbent assay for detection of immunoglobulin G antibodies to Crimean-Congo hemorrhagic fever virus.	No data on CCHFV prevalence or case fatality rate
227	Saijo, 2002	Immunofluorescence technique using HeLa cells expressing recombinant nucleoprotein for detection of immunoglobulin G antibodies to Crimean-Congo hemorrhagic fever virus.	No data on CCHFV prevalence or case fatality rate
228	Saijo, 2005	Antigen-capture enzyme-linked immunosorbent assay for the diagnosis of crimean-congo hemorrhagic fever using a novel monoclonal antibody.	No data on CCHFV prevalence or case fatality rate
229	Saijo, 2005	Recombinant nucleoprotein-based serological diagnosis of Crimean-Congo hemorrhagic fever virus infections.	No data on CCHFV prevalence or case fatality rate
230	Saijo, 2007	Crimean-Congo hemorrhagic fever in the Xinjiang Uygur autonomous region of western China.	Review
231	Salehi-Vaziri, 2017	An outbreak of crimean-Congo hemorrhagic fever in the south west of Iran.	Case report
232	Saluzzo, 1984	Isolation of Crimean-Congo haemorrhagic fever and Rift Valley fever viruses in Upper Volta.	Report
233	Saluzzo, 1985	Haemorrhagic fever caused by Crimean Congo haemorrhagic fever virus in Mauritania.	Case report
234	Saluzzo, 1985	Comparaison de différentes techniquespour la détection du virus de la fièvre jaune dans les prélèvements humains et les lots de moustiques: Intérêt d'une méthode rapide de diagnostic par ELISA.	Sample size < or = 10 participants
235	Saluzzo, 1987	Rapid diagnosis of human Crimean-Congo hemorrhagic fever and detection of the virus in naturally infected ticks.	Not possible to extract data on CCHFV prevalence or case fatality rate
236	Sargianou, 2013	Epidemiological and behavioral factors associated with Crimean-Congo hemorrhagic fever virus infections in humans.	Review
237	Sas, 2018	A novel double-antigen sandwich ELISA for the species-independent detection of Crimean-Congo hemorrhagic fever virus-specific antibodies.	No data on CCHFV prevalence or case fatality rate
238	Schuster, 2016	Sheep and goats as indicator animals for the circulation of CCHFV in the environment.	Duplicate study
239	Schuster, 2016	A competitive ELISA for species-independent detection of Crimean-Congo hemorrhagic fever virus specific antibodies.	No data on CCHFV prevalence or case fatality rate

240	Schwarz, 1997	Clinical features of Crimean-Congo haemorrhagic fever in the United Arab Emirates.	Sample size < or = 10 participants
241	Scrimgeour, 1996	Crimean-Congo haemorrhagic fever in Oman.	Case report
242	Scrimgeour, 1996	Crimean-Congo haemorrhagic fever in Oman.	Duplicate study
243	Seregin, 2004	Study of the genetic variability of Crimean-Congo hemorrhagic fever virus in Central Asia.	No data on CCHFV prevalence or case fatality rate
244	Shahbazi, 2019	Seroepidemiological survey of Crimean-Congo haemorrhagic fever among high-risk groups in the west of Iran.	Duplicate study
245	Shahhosseini, 2017	Crimean-Congo hemorrhagic fever cases in the North of Iran have three distinct origins.	No data on CCHFV prevalence or case fatality rate
246	Shanmugam, 1976	Presence of antibody to arboviruses of the Crimean Haemorrhagic Fever-Congo (CHF-Congo) group in human beings and domestic animals in India.	No full text
247	Sharififard, 2016	Epidemiological Survey of Crimean-Congo Hemorrhagic Fever (CCHF), a Fatal Infectious Disease in Khuzestan Province, Southwest Iran, During 1999 - 2015.	Duplicate study
248	Sharifi-Mood, 2014	Prevalence of crimean-congo hemorrhagic Fever among high risk human groups.	No data on CCHFV prevalence or case fatality rate
249	Sharifinia, 2015	Hard ticks (Ixodidae) and Crimean-Congo hemorrhagic fever virus in south west of Iran.	Duplicate study
250	Shepherd, 1988	Evaluation of enzyme-linked immunosorbent assay and reversed passive hemagglutination for detection of Crimean-Congo hemorrhagic fevers virus antigen.	No data on CCHFV prevalence or case fatality rate
251	Shepherd, 1989	Viremia and antibody response of small African and laboratory animals to Crimean-Congo hemorrhagic fever virus infection.	Experimental infection
252	Shepherd, 1989	Antibody response in Crimean-Congo hemorrhagic fever.	Report
253	Shuaib, 2020	Ixodid tick species and two tick-borne pathogens in three areas in the Sudan.	Duplicate study
254	Sidira, 2013	Prevalence of Crimean-Congo hemorrhagic fever virus antibodies in Greek residents in the area where the AP92 strain was isolated.	Duplicate study
255	Singh, 2016	Molecular epidemiology of Crimean-Congo haemorrhagic fever virus in India.	Sample size < or = 10 participants
256	Sisman, 2013	Epidemiologic features and risk factors of Crimean-Congo hemorrhagic fever in Samsun province, Turkey.	No data on CCHFV prevalence or case fatality rate
257	Smirnova, 1985	Detection of Crimean haemorrhagic fever virus antigen by solid phase enzyme immunosorbent assay.	No full text
258	Suleiman, 1980	Congo/Crimean haemorrhagic fever in Dubai. An outbreak at the Rashid Hospital.	Case report
259	Swanepoel, 1983	Reversed passive hemagglutination and inhibition with Rift Valley fever and Crimean-Congo hemorrhagic fever viruses.	No data on CCHFV prevalence or case fatality rate
260	Swanepoel, 1985	A common-source outbreak of Crimean-Congo haemorrhagic fever on a dairy farm.	No full text
261	Sylla, 2009	Climatic variation and distribution of Crimean Congo hemorrhagic fever and Cowdriosis, tick-borne diseases in Senegal.	Not possible to extract data on CCHFV prevalence or case fatality rate
262	Tahmasebi, 2010	Molecular epidemiology of crimean-congo hemorrhagic fever virus genome isolated of ticks from Hamadan province of Iran.	Duplicate study

263	Tahmasebia, 2010	Molecular epidemiology of Crimean- Congo hemorrhagic fever virus genome isolated from ticks of Hamadan province of Iran.	Duplicate study
264	Tall, 2009	Crimean-Congo hemorrhagic fever in Senegal.	No full text
265	Tantawi, 1980	Crimean-Congo haemorrhagic fever virus in Iraq: isolation, identification and electron microscopy.	Sample size < or = 10 participants
266	Tarantola, 2006	Lookback exercise with imported Crimean-Congo hemorrhagic fever, Senegal and France.	Case report
267	Taseva, 2010	Features of Crimean-Congo haemorrhagic fever in patients with febrile syndrome in Bulgaria.	Study with serial samples from the same patient
268	Taverne, 2002	Tick-borne haemorrhagic fever in Iran.	Report
269	Telmadarraiy, 2015	Vectors of Crimean Congo Hemorrhagic Fever Virus in Iran.	Review
270	Tezer, 2010	Crimean-Congo hemorrhagic fever in children.	No data on CCHFV prevalence or case fatality rate
271	Thomas, 2019	Contact Tracing for an Imported Case of Crimean-Congo Hemorrhagic Fever - Experience from a Tertiary Care Center in Kerala, South India.	Case report
272	Tikriti, 1981	Congo/Crimean haemorrhagic fever in Iraq: a seroepidemiological survey.	No full text
273	Turell, 2007	Role of ticks in the transmission of Crimean-Congo hemorrhagic fever virus.	Review
274	Uyar, 2014	Investigation of hantavirus infections among CCHFV negative cases in the western black sea region of Turkey.	No data on CCHFV prevalence or case fatality rate
275	Uysal, 2015	Alterations of serum brain type natriuretic peptide (BNP) in patients with Crimean-Congo hemorrhagic fever.	Study with serial samples from the same patient
276	van de Wal, 1985	A nosocomial outbreak of Crimean-Congo haemorrhagic fever at Tygerberg Hospital. Part IV. Preventive and prophylactic measures.	No data on CCHFV prevalence or case fatality rate
277	Van Eeden, 1985	A nosocomial outbreak of Crimean-Congo haemorrhagic fever at Tygerberg Hospital. Part I. Clinical features.	Case report
278	Vashakidze, 2007	Crimean-Congo hemorrhagic fever in Turkey.	Review
279	Vashakidze, 2015	EPIDEMIOLOGY, CLINICAL AND LABORATORY FEATURES OF CRIMEAN-CONGO HEMORRHAGIC FEVER IN GEORGIA.	Not possible to extract data on CCHFV prevalence or case fatality rate
280	Vescio, 2012	Environmental correlates of Crimean-Congo haemorrhagic fever incidence in Bulgaria.	Not possible to extract data on CCHFV prevalence or case fatality rate
281	Voorhees, 2018	Crimean-Congo Hemorrhagic Fever Virus, Mongolia, 2013-2014.	Duplicate study
282	Waheed Uz Zaman, 2006	Crimean - congo haemorrhagic fever [CCHF] in Pakistan.	No full text
283	Walker, 2015	Genomic Characterization of Yogue, Kasokero, Issyk-Kul, Keterah, Gossas, and Thiafora Viruses: Nairoviruses Naturally Infecting Bats, Shrews, and Ticks.	No data on CCHFV prevalence or case fatality rate
284	WHO Eastern Mediterranean Regional Office, 2008	CCHF in Afghanistan: outbreak contained.	No data on CCHFV prevalence or case fatality rate
285	WHO Eastern Mediterranean Regional Office, 2009	Summary of outbreaks in EMR: 2008.	No data on CCHFV prevalence or case fatality rate

286	WHO Eastern Mediterranean Regional Office, 2010	CCHF in Iraq.	No data on CCHFV prevalence or case fatality rate
287	WHO Eastern Mediterranean Regional Office, 2010	Dengue fever and CCHF in Pakistan.	No data on CCHFV prevalence or case fatality rate
288	WHO Eastern Mediterranean Regional Office, 2010	Nosocomial outbreak of CCHF in Pakistan.	No data on CCHFV prevalence or case fatality rate
289	WHO Eastern Mediterranean Regional Office, 2012	CCHF In Pakistan.	No data on CCHFV prevalence or case fatality rate
290	WHO Eastern Mediterranean Regional Office, 2013	CCHF continues to claim lives in Pakistan.	Report
291	WHO Eastern Mediterranean Regional Office, 2014	CCHF in Pakistan.	Report
292	WHO Eastern Mediterranean Regional Office, 2014	CCHF remains entrenched in Pakistan.	Report
293	WHO Eastern Mediterranean Regional Office, 2014	Crimean-Congo haemorrhagic fever in Pakistan.	Report
294	WHO Eastern Mediterranean Regional Office, 2014	Crimean-Congo Hemorrhagic Fever in Pakistan, 2014.	Report
295	WHO Eastern Mediterranean Regional Office, 2016	CCHF in Pakistan: cases rise.	Report
296	WHO Eastern Mediterranean Regional Office, 2017	CCHF in Afghanistan.	Report
297	WHO Eastern Mediterranean Regional Office, 2017	Crimean-Congo hemorrhagic fever in Afghanistan.	Report
298	WHO Eastern Mediterranean Regional Office, 2017	Ebola virus disease in DRC: need for enhanced preparedness and readlness measures in EMR.	Report
299	WHO Eastern Mediterranean Regional Office, 2018	High number of CCHF cases in Afghanistan: an assessment conducted.	Report
300	Williams, 2000	Crimean-Congo haemorrhagic fever: A seroepidemiological and tick survey in the Sultanate of Oman.	Duplicate study

301	Wölfel, 2007	Virus detection and monitoring of viral load in Crimean-Congo hemorrhagic fever virus patients.	No data on CCHFV prevalence or case fatality rate
302	Wölfel, 2009	Low-density macroarray for rapid detection and identification of Crimean-Congo hemorrhagic fever virus.	No data on CCHFV prevalence or case fatality rate
303	Wu, 2014	Simultaneous detection of IgG antibodies associated with viral hemorrhagic fever by a multiplexed Luminex-based immunoassay.	No data on CCHFV prevalence or case fatality rate
304	Yadav, 2013	Genetic characterization and molecular clock analyses of the Crimean-Congo hemorrhagic fever virus from human and ticks in India, 2010-2011.	No data on CCHFV prevalence or case fatality rate
305	Yadav, 2016	Nosocomial infection of CCHF among health care workers in Rajasthan, India.	Case report
306	Yadav, 2017	Crimean-Congo Hemorrhagic Fever in Migrant Worker Returning from Oman to India, 2016.	Case report
307	Yadav, 2018	Positivity of dengue and chikungunya among Crimean-Congo hemorrhagic fever-negative cases in India: 2013-2016.	No data on CCHFV prevalence or case fatality rate
308	Yadav, 2019	Persistence of IgG antibodies in survivors of Crimean Congo hemorrhagic fever virus infection, India.	No data on CCHFV prevalence or case fatality rate
309	Yadav, 2019	Characterization of Novel Reoviruses Wad Medani Virus (Orbivirus) and Kundal Virus (Coltivirus) Collected from Hyalomma anatolicum Ticks in India during Surveillance for Crimean Congo Hemorrhagic Fever.	No data on CCHFV prevalence or case fatality rate
310	Yapar, 2005	Rapid and quantitative detection of Crimean-Congo hemorrhagic fever virus by one-step real-time reverse transcriptase-PCR.	No data on CCHFV prevalence or case fatality rate
311	Yaser, 2011	Crimean-Congo hemorrhagic fever: A molecular survey on hard ticks (Ixodidae) in Yazd province, Iran.	Duplicate study
312	Yashina, 2003	Genetic analysis of Crimean-Congo hemorrhagic fever virus in Russia.	Not possible to extract data on CCHFV prevalence or case fatality rate
313	Yashina, 2014	Crimean-Congo hemorrhagic fever virus in Stavropol krai in 2011.	No data on CCHFV prevalence or case fatality rate
314	Yen, 1985	Characteristics of Crimean-Congo hemorrhagic fever virus (Xinjiang strain) in China.	No data on CCHFV prevalence or case fatality rate
315	Yildirmak, 2016	Crimean-Congo haemorrhagic fever: transmission to visitors and healthcare workers.	Case report
316	Yilmaz, 2008	A preliminary report on Crimean-Congo haemorrhagic fever in Turkey, March - June 2008.	Report
317	Yilmaz, 2016	Prognostic impact of platelet distribution width in patients with Crimean-Congo hemorrhagic fever.	No data on CCHFV prevalence or case fatality rate
318	Zahraei, 2015	Novel, in-house, sybr green based one-step rRT-PCR: Rapid and accurate diagnosis of crimean-congo hemorrhagic fever virus in suspected patients from Iran.	Duplicate study
319	Zahraei, 2016	Novel, In-House, SYBR Green Based One-Step rRT-PCR: Rapid and Accurate Diagnosis of Crimean-Congo Hemorrhagic Fever Virus in Suspected Patients From Iran.	Samples with already known result
320	Zakhashvili, 2010	Crimean-Congo hemorrhagic fever in man, Republic of Georgia, 2009.	Case report

321	Zeller, 1997	Crimean-Congo hemorrhagic fever in ticks (Acari: Ixodidae) and ruminants: field observations of an epizootic in Bandia, Senegal (1989-1992).	Not possible to extract data on CCHFV prevalence or case fatality rate
322	Zhang, 2018	Isolation, Characterization, and Phylogenetic Analysis of Two New Crimean-Congo Hemorrhagic Fever Virus Strains from the Northern Region of Xinjiang Province, China.	No data on CCHFV prevalence or case fatality rate
323	Zivcec, 2017	Genome Sequences of Crimean-Congo Hemorrhagic Fever Virus Strains Isolated in South Africa, Namibia, and Turkey.	No data on CCHFV prevalence or case fatality rate
