# Supplementary material for: Worldwide epidemiology of Crimean-Congo Hemorrhagic Fever Virus in humans, ticks and other animal species, a systematic review and meta-analysis
Source: PLoS Negl Trop Dis. 2021 Apr 22;15(4):e0009299. doi: 10.1371/journal.pntd.0009299 (PMC8096040; doi:10.1371/journal.pntd.0009299)
Supplement: S5 Table — (PDF) [file pntd.0009299.s009.pdf]

S5 Table. Risk of bias assessment

Author	Was the study's target population a close representation of the national population in relation to LASV prevalence?	Was the sampling frame a true or close representation of the target population?	Was some form of random selection used to select the sample, OR was a census undertaken?	Were data collected directly from the subjects (as opposed to a proxy)?	Was an acceptable case definition used in the study?	Was the response rate ≥ 70 % or not significant different in relevant demographic characteristics between responders and nonresponders?	Was the CCHF detection assay shown to have reliability and validity?	Was the same mode of data collection used for all subjects?	Was the length of the study period >= 1 year?	Were the numerator(s) and denominator(s) for the CCHFV prevalence or case fatality rate appropriate?	Species	Risk of bias
Abdiyeva, 2019	No	Yes	No	Yes	Yes	Yes	Yes	Yes	No	Yes	Humans	Low risk of bias
Adam, 2013	No	Yes	Yes	Not applicable	No	No	Yes	Yes	Unclear	Yes	Other animals	Moderate risk of bias
Ahmed, 2019	No	Yes	No	Yes	No	No	Yes	Yes	No	Yes	Humans	Moderate risk of bias
Ahmeti, 2019	No	Yes	No	Yes	No	No	Yes	Yes	Yes	Yes	Humans	Moderate risk of bias
Akuffo, 2016	No	Yes	No	Yes	No	No	Unclear	Yes	No	Yes	Humans	Moderate risk of bias
Akuffo, 2016	No	Yes	No	Yes	No	No	Unclear	Yes	No	Yes	Humans	Moderate risk of bias
Akuffo, 2016	No	Yes	No	Not applicable	No	No	Unclear	Yes	No	Yes	Tick pools	Moderate risk of bias
Al Adhamy, 1992	No	Yes	No	Yes	No	No	Yes	Yes	Unclear	Yes	Humans	Moderate risk of bias
Al Adhamy, 1992	No	Yes	No	Not applicable	No	No	Yes	Yes	Unclear	Yes	Other animals	Moderate risk of bias
Alam, 2013	No	Yes	No	Yes	Yes	No	Unclear	Yes	No	Yes	Humans	Moderate risk of bias
Alam, 2017	No	Yes	No	Yes	No	No	Yes	Yes	Yes	Yes	Humans	Moderate risk of bias
Albayrak, 2010	No	Yes	No	Not applicable	No	No	Yes	Yes	No	Yes	Tick pools	Moderate risk of bias
Albayrak, 2010	No	Yes	No	Not applicable	No	No	Yes	Yes	No	Yes	Tick pools	Moderate risk of bias
Albayrak, 2010	No	Yes	No	Not applicable	No	No	Yes	Yes	No	Yes	Individual tick	Moderate risk of bias
Albayrak, 2010	No	Yes	No	Not applicable	No	No	Yes	Yes	No	Yes	Individual tick	Moderate risk of bias
Albayrak, 2012	No	Yes	No	Not applicable	No	No	Yes	Yes	No	Yes	Tick pools	Moderate risk of bias
Albayrak, 2012	No	Yes	No	Not applicable	No	No	Yes	Yes	No	Yes	Individual tick	Moderate risk of bias
Albayrak, 2012	No	Yes	No	Not applicable	No	No	Yes	Yes	No	Yes	Other animals	Moderate risk of bias
Albayrak, 2012	No	Yes	No	Not applicable	No	No	Yes	Yes	No	Yes	Other animals	Moderate risk of bias
Albayrak, 2012	No	Yes	No	Not applicable	No	No	Yes	Yes	No	Yes	Individual tick	Moderate risk of bias
Albayrak, 2012	No	Yes	No	Not applicable	No	No	Yes	Yes	No	Yes	Other animals	Moderate risk of bias
Albayrak, 2012	No	Yes	No	Not applicable	No	No	Yes	Yes	No	Yes	Other animals	Moderate risk of bias
Almasri, 2019	No	Yes	No	Yes	Unclear	Unclear	Yes	Yes	No	Yes	Humans	Moderate risk of bias
Al-Nakib, 1984	No	Yes	Yes	Yes	No	No	Yes	Yes	Yes	Yes	Humans	Low risk of bias
Altaf, 1998	No	Yes	No	Yes	Unclear	Unclear	Yes	Yes	No	Yes	Humans	Moderate risk of bias
Altaf, 1998	No	Yes	No	Yes	Unclear	Unclear	Yes	Yes	No	Yes	Humans	Moderate risk of bias
Andriamandimby, 2011	Yes	Yes	No	Yes	No	Unclear	Yes	Yes	No	Yes	Humans	Moderate risk of bias
Andriamandimby, 2011	Yes	Yes	No	Yes	No	Unclear	Yes	Yes	No	Yes	Humans	Moderate risk of bias
Antoniadis, 1982	No	Yes	No	Yes	No	No	Yes	Yes	Yes	Yes	Humans	Moderate risk of bias
Aslani, 2017	No	Yes	No	Yes	No	Unclear	Yes	Yes	Yes	Yes	Humans	Moderate risk of bias
Aslani, 2017	No	Yes	No	Yes	No	Unclear	Yes	Yes	Yes	Yes	Humans	Moderate risk of bias
Athar, 2005	No	Yes	No	Yes	No	Unclear	Yes	Yes	No	Yes	Humans	Moderate risk of bias
Aykut, 2007	No	Yes	No	Not applicable	No	No	Yes	Yes	Yes	Yes	Tick pools	Moderate risk of bias
Aykut, 2007	No	Yes	No	Not applicable	No	No	Yes	Yes	Yes	Yes	Individual tick	Moderate risk of bias
Aykut, 2007	No	Yes	No	Not applicable	No	No	Yes	Yes	Yes	Yes	Individual tick	Moderate risk of bias
Azagi, 2017	No	Yes	No	Not applicable	No	No	Yes	Yes	Yes	Yes	Individual tick	Moderate risk of bias
Azagi, 2017	No	Yes	No	Not applicable	No	No	Yes	Yes	Yes	Yes	Individual tick	Moderate risk of bias
Azagi, 2017	No	Yes	No	Not applicable	No	No	Yes	Yes	Yes	Yes	Individual tick	Moderate risk of bias
Azagi, 2017	No	Yes	No	Not applicable	No	No	Yes	Yes	Yes	Yes	Individual tick	Moderate risk of bias
Bakir, 2012	No	Yes	No	Yes	Yes	No	Yes	Yes	Yes	Yes	Humans	Low risk of bias
Bakir, 2012	No	Yes	No	Yes	Yes	No	Yes	Yes	Yes	Yes	Humans	Low risk of bias
Bakır, 2015	No	Yes	No	Yes	Yes	No	Yes	Yes	No	Yes	Humans	Moderate risk of bias
Bakir, 2016	No	Yes	No	Yes	Yes	No	Yes	Yes	No	Yes	Humans	Moderate risk of bias
Balinandi, 2018	No	Yes	Yes	Not applicable	No	Unclear	Yes	Yes	No	Yes	Individual tick	Moderate risk of bias
Balinandi, 2018	No	Yes	Yes	Not applicable	No	Unclear	Yes	Yes	No	Yes	Individual tick	Moderate risk of bias
Barthel, 2014	No	Yes	No	Not applicable	No	Unclear	Yes	Yes	No	Yes	Other animals	Moderate risk of bias
Barthel, 2014	No	Yes	No	Not applicable	No	Unclear	Yes	Yes	No	Yes	Other animals	Moderate risk of bias
Barthel, 2014	No	Yes	No	Not applicable	No	Unclear	Yes	Yes	No	Yes	Other animals	Moderate risk of bias
Batool, 2009	No	Yes	No	Yes	Yes	Unclear	Yes	Yes	Yes	Yes	Humans	Low risk of bias
Baumann, 2019	No	Yes	No	Yes	Unclear	Unclear	Yes	Yes	Yes	Yes	Humans	Moderate risk of bias
Bayram, 2017	No	Yes	Yes	Yes	No	No	Yes	Yes	No	Yes	Humans	Moderate risk of bias
Bazanów, 2017	No	Yes	No	Not applicable	No	No	Yes	Yes	Yes	Yes	Other animals	Moderate risk of bias
Bazanów, 2017	No	Yes	No	Not applicable	No	No	Yes	Yes	Yes	Yes	Individual tick	Moderate risk of bias
Bazanów, 2017	No	Yes	No	Not applicable	No	No	Yes	Yes	Yes	Yes	Individual tick	Moderate risk of bias
Behrooz, 2006	No	Yes	No	Not applicable	No	No	Yes	Yes	Yes	Yes	Other animals	Moderate risk of bias
Belet, 2014	No	Yes	No	Yes	No	No	Yes	Yes	Yes	Yes	Humans	Moderate risk of bias
Belet, 2014	No	Yes	No	Yes	No	No	Yes	Yes	Yes	Yes	Humans	Moderate risk of bias
Belet, 2014	No	Yes	No	Yes	No	No	Yes	Yes	Yes	Yes	Humans	Moderate risk of bias
Belet, 2014	No	Yes	No	Yes	No	No	Yes	Yes	Yes	Yes	Humans	Moderate risk of bias
Biglari, 2016	No	Yes	Yes	Not applicable	No	No	Yes	Yes	Yes	Yes	Individual tick	Moderate risk of bias
Biglari, 2016	No	Yes	Yes	Not applicable	No	No	Yes	Yes	Yes	Yes	Individual tick	Moderate risk of bias
Biglari, 2016	No	Yes	Yes	Not applicable	No	No	Yes	Yes	Yes	Yes	Individual tick	Moderate risk of bias
Biglari, 2016	No	Yes	Yes	Not applicable	No	No	Yes	Yes	Yes	Yes	Individual tick	Moderate risk of bias
Biglari, 2016	No	Yes	Yes	Not applicable	No	No	Yes	Yes	Yes	Yes	Individual tick	Moderate risk of bias
Bilgin, 2014	No	Yes	No	Yes	No	No	Yes	Yes	Yes	Yes	Humans	Moderate risk of bias
Blackburn, 1982	No	Yes	No	Yes	No	No	Yes	Yes	Unclear	Yes	Humans	Moderate risk of bias
Bob, 2017	No	Yes	No	Yes	Yes	No	Yes	Yes	No	Yes	Humans	Moderate risk of bias
Bob, 2017	No	Yes	No	Yes	Yes	No	Yes	Yes	No	Yes	Humans	Moderate risk of bias
Bodur, 2012	No	Yes	Yes	Yes	No	Yes	Yes	Yes	No	Yes	Humans	Low risk of bias
Bokaie, 2008	Yes	Yes	No	Yes	Yes	No	Yes	Yes	Yes	Yes	Humans	Low risk of bias
Bokaie, 2008	Yes	Yes	No	Yes	Yes	No	Yes	Yes	Yes	Yes	Humans	Low risk of bias
Bokaie, 2008	Yes	Yes	No	Yes	Yes	No	Yes	Yes	Yes	Yes	Humans	Low risk of bias
Bokaie, 2008	Yes	Yes	No	Yes	Yes	No	Yes	Yes	Yes	Yes	Humans	Low risk of bias
Bokaie, 2008	Yes	Yes	No	Yes	Yes	No	Yes	Yes	Yes	Yes	Humans	Low risk of bias
Bokaie, 2008	Yes	Yes	No	Not applicable	No	No	Yes	Yes	Yes	Yes	Other animals	Moderate risk of bias
Bokaie, 2008	Yes	Yes	No	Not applicable	No	No	Yes	Yes	Yes	Yes	Other animals	Moderate risk of bias
Bonney, 2013	No	Yes	No	Yes	Yes	No	Yes	Yes	Yes	Yes	Humans	Low risk of bias
Botros, 1989	No	Yes	No	Yes	Yes	No	Yes	Yes	Unclear	Yes	Humans	Moderate risk of bias
Bower, 2019	No	Yes	No	Yes	Yes	No	Yes	Yes	Unclear	Yes	Humans	Moderate risk of bias
Bower, 2019	No	Yes	No	Yes	Yes	No	Yes	Yes	Unclear	Yes	Humans	Moderate risk of bias
Bower, 2019	No	Yes	No	Yes	Yes	No	Yes	Yes	Unclear	Yes	Humans	Moderate risk of bias
Bozkurt, 2016	No	Yes	No	Yes	Yes	No	Yes	Yes	Yes	Yes	Humans	Low risk of bias
Bryan, 1996	No	Yes	No	Yes	No	Unclear	Yes	Yes	No	Yes	Humans	Moderate risk of bias
Bukbuk, 2014	No	Yes	Yes	Yes	No	No	Yes	Yes	No	Yes	Humans	Moderate risk of bias

Bukbuk, 2016	No	Yes	Yes	Yes	No	Unclear	Yes	Yes	Yes	Yes	Humans	Low risk of bias
Bukbuk, 2016	No	Yes	Yes	Yes	No	Unclear	Yes	Yes	Yes	Yes	Humans	Low risk of bias
Bukbuk, 2016	No	Yes	Yes	Yes	No	Unclear	Yes	Yes	Yes	Yes	Humans	Low risk of bias
Bursali, 2011	No	Yes	No	Not applicable	No	Unclear	Yes	Yes	No	Yes	Tick pools	Moderate risk of bias
Burt, 1993	No	Yes	No	Not applicable	No	No	Yes	Yes	Yes	Yes	Other animals	Moderate risk of bias
Burt, 1993	No	Yes	No	Not applicable	No	No	Yes	Yes	Yes	Yes	Other animals	Moderate risk of bias
Burt, 1993	No	Yes	No	Not applicable	No	No	Yes	Yes	Yes	Yes	Other animals	Moderate risk of bias
Burt, 1993	No	Yes	No	Not applicable	No	No	Yes	Yes	Yes	Yes	Other animals	Moderate risk of bias
Burt, 1993	No	Yes	No	Not applicable	No	No	Yes	Yes	Yes	Yes	Other animals	Moderate risk of bias
Burt, 1993	No	Yes	No	Not applicable	No	No	Yes	Yes	Yes	Yes	Other animals	Moderate risk of bias
Burt, 1993	No	Yes	No	Not applicable	No	No	Yes	Yes	Yes	Yes	Other animals	Moderate risk of bias
Burt, 1993	No	Yes	No	Not applicable	No	No	Yes	Yes	Yes	Yes	Other animals	Moderate risk of bias
Burt, 1993	No	Yes	No	Not applicable	No	No	Yes	Yes	Yes	Yes	Other animals	Moderate risk of bias
Burt, 1993	No	Yes	No	Not applicable	No	No	Yes	Yes	Yes	Yes	Other animals	Moderate risk of bias
Burt, 1993	No	Yes	No	Not applicable	No	No	Yes	Yes	Yes	Yes	Other animals	Moderate risk of bias
Burt, 1993	No	Yes	No	Not applicable	No	No	Yes	Yes	Yes	Yes	Other animals	Moderate risk of bias
Burt, 1993	No	Yes	No	Not applicable	No	No	Yes	Yes	Yes	Yes	Other animals	Moderate risk of bias
Burt, 1993	No	Yes	No	Not applicable	No	No	Yes	Yes	Yes	Yes	Other animals	Moderate risk of bias
Burt, 1993	No	Yes	No	Not applicable	No	No	Yes	Yes	Yes	Yes	Other animals	Moderate risk of bias
Burt, 1996	No	Yes	No	Not applicable	No	No	Yes	Yes	Yes	Yes	Other animals	Moderate risk of bias
Cajimat, 2017	No	Yes	No	Not applicable	No	Unclear	Yes	Yes	No	Yes	Tick pools	Moderate risk of bias
Ceianu, 2012	No	Yes	No	Not applicable	No	Unclear	Yes	Yes	No	Yes	Other animals	Moderate risk of bias
Çelik, 2016	No	Yes	No	Yes	Yes	Unclear	Yes	Yes	Yes	Yes	Humans	Low risk of bias
Cevik, 2008	No	Yes	No	Yes	Yes	Unclear	Yes	Yes	Yes	Yes	Humans	Low risk of bias
Champour, 2014	No	Yes	Yes	Not applicable	No	Unclear	Yes	Yes	No	Yes	Other animals	Moderate risk of bias
Champour, 2014	No	Yes	Yes	Not applicable	No	Unclear	Yes	Yes	No	Yes	Individual tick	Moderate risk of bias
Champour, 2014	No	Yes	Yes	Not applicable	No	Unclear	Yes	Yes	No	Yes	Individual tick	Moderate risk of bias
Champour, 2014	No	Yes	Yes	Not applicable	No	Unclear	Yes	Yes	No	Yes	Individual tick	Moderate risk of bias
Chantal, 1996	No	Yes	No	Yes	No	No	Yes	Yes	Unclear	Yes	Humans	Moderate risk of bias
Chapman, 1991	No	Yes	No	Yes	Yes	No	Yes	Yes	No	Yes	Humans	Moderate risk of bias
Chapman, 1991	No	Yes	No	Yes	Yes	No	Yes	Yes	No	Yes	Humans	Moderate risk of bias
Chinikar, 2007	No	Yes	No	Yes	Yes	No	Yes	Yes	Yes	Yes	Humans	Low risk of bias
Chinikar, 2007	No	Yes	No	Yes	Yes	No	Yes	Yes	Yes	Yes	Humans	Low risk of bias
Chinikar, 2007	No	Yes	No	Yes	Yes	No	Yes	Yes	Yes	Yes	Humans	Low risk of bias
Chinikar, 2008	No	Yes	No	Yes	Yes	No	Yes	Yes	No	Yes	Humans	Moderate risk of bias
Chinikar, 2008	No	Yes	No	Not applicable	No	No	Yes	Yes	No	Yes	Other animals	Moderate risk of bias
Chinikar, 2010	No	Yes	No	Yes	No	Unclear	Yes	Yes	Yes	Yes	Humans	Moderate risk of bias
Chinikar, 2010	No	Yes	No	Yes	No	Unclear	Yes	Yes	Yes	Yes	Humans	Moderate risk of bias
Chinikar, 2010	No	Yes	No	Not applicable	No	Unclear	Yes	Yes	Yes	Yes	Other animals	Moderate risk of bias
Chinikar, 2012	No	Yes	No	Yes	No	Unclear	Yes	Yes	Yes	Yes	Humans	Moderate risk of bias
Chinikar, 2012	No	Yes	Yes	Yes	No	No	Yes	Yes	Yes	Yes	Humans	Low risk of bias
Chinikar, 2012	No	Yes	No	Not applicable	No	Unclear	Yes	Yes	Yes	Yes	Other animals	Moderate risk of bias
Chinikar, 2012	No	Yes	No	Not applicable	No	Unclear	Yes	Yes	Yes	Yes	Individual tick	Moderate risk of bias
Chisholm, 2012	No	Yes	No	Not applicable	No	Unclear	Yes	Yes	No	Yes	Tick pools	Moderate risk of bias
Chisholm, 2012	No	Yes	No	Not applicable	No	Unclear	Yes	Yes	No	Yes	Individual tick	Moderate risk of bias
Choubdar, 2019	No	Yes	Yes	Not applicable	No	Unclear	Yes	Yes	Yes	Yes	Individual tick	Moderate risk of bias
Christova, 2013	No	Yes	No	Yes	No	Unclear	Yes	Yes	Yes	Yes	Humans	Moderate risk of bias
Christova, 2013	No	Yes	No	Yes	No	Unclear	Yes	Yes	Yes	Yes	Humans	Moderate risk of bias
Christova, 2013	No	Yes	No	Yes	No	Unclear	Yes	Yes	Yes	Yes	Humans	Moderate risk of bias
Christova, 2017	No	Yes	Yes	Yes	No	Unclear	Yes	Yes	No	Yes	Humans	Moderate risk of bias
Christova, 2018	No	Yes	No	Not applicable	No	Unclear	Yes	Yes	Yes	Yes	Other animals	Moderate risk of bias
Christova, 2018	No	Yes	No	Not applicable	No	Unclear	Yes	Yes	Yes	Yes	Other animals	Moderate risk of bias
Christova, 2018	No	Yes	No	Not applicable	No	Unclear	Yes	Yes	Yes	Yes	Other animals	Moderate risk of bias
Cikman, 2016	No	Yes	No	Yes	No	Unclear	Yes	Yes	Unclear	Yes	Humans	Moderate risk of bias
Cikman, 2016	No	Yes	No	Yes	No	Unclear	Yes	Yes	Unclear	Yes	Humans	Moderate risk of bias
Clements, 2019	No	Yes	No	Yes	No	Unclear	Yes	Yes	Yes	Yes	Humans	Moderate risk of bias
Darwish, 1983	No	Yes	No	Yes	Yes	No	Yes	Yes	Unclear	Yes	Humans	Moderate risk of bias
Darwish, 1983	No	Yes	No	Not applicable	No	No	Yes	Yes	Unclear	Yes	Other animals	Moderate risk of bias
Darwish, 1983	No	Yes	No	Not applicable	No	No	Yes	Yes	Unclear	Yes	Other animals	Moderate risk of bias
Darwish, 1983	No	Yes	No	Not applicable	No	No	Yes	Yes	Unclear	Yes	Other animals	Moderate risk of bias
Darwish, 1983	No	Yes	No	Not applicable	No	No	Yes	Yes	Unclear	Yes	Other animals	Moderate risk of bias
Darwish, 1983	No	Yes	No	Not applicable	No	No	Yes	Yes	Unclear	Yes	Other animals	Moderate risk of bias
David-West, 1974	No	Yes	No	Yes	No	Unclear	Yes	Yes	No	Yes	Humans	Moderate risk of bias
De Liberato, 2018	No	Yes	Yes	Not applicable	No	No	Yes	Yes	Yes	Yes	Other animals	Moderate risk of bias
Dilber, 2009	No	Yes	No	Yes	Yes	Unclear	Yes	Yes	Yes	Yes	Humans	Low risk of bias
Dilber, 2009	No	Yes	No	Yes	Yes	Unclear	Yes	Yes	Yes	Yes	Humans	Low risk of bias
Dinçer, 2017	No	Yes	No	Not applicable	No	No	Yes	Yes	Yes	Yes	Tick pools	Moderate risk of bias
Dinçer, 2017	No	Yes	No	Not applicable	No	No	Yes	Yes	Yes	Yes	Individual tick	Moderate risk of bias
Dinçer, 2017	No	Yes	No	Not applicable	No	No	Yes	Yes	Yes	Yes	Individual tick	Moderate risk of bias
Dinçer, 2017	No	Yes	No	Not applicable	No	No	Yes	Yes	Yes	Yes	Individual tick	Moderate risk of bias
Dokuzoguz, 2013	No	Yes	No	Yes	Yes	Unclear	Yes	Yes	Yes	Yes	Humans	Low risk of bias
Duygu, 2012	No	Yes	No	Yes	No	No	Yes	Yes	Yes	Yes	Humans	Moderate risk of bias
Duygu, 2018	No	Yes	No	Yes	Yes	No	Yes	Yes	No	Yes	Humans	Moderate risk of bias
el-Azazy, 1997	No	Yes	Yes	Yes	No	No	Yes	Yes	No	Yes	Humans	Moderate risk of bias
el-Azazy, 1997	No	Yes	Yes	Yes	No	No	Yes	Yes	No	Yes	Humans	Moderate risk of bias
England, 2016	No	Yes	No	Not applicable	No	No	Yes	Yes	No	Yes	Individual tick	Moderate risk of bias
Enkhtsetseg, 2016	No	Yes	No	Yes	No	No	Yes	Yes	Unclear	Yes	Humans	Moderate risk of bias
Eren, 2016	No	Yes	No	Yes	Yes	No	Yes	Yes	Yes	Yes	Humans	Low risk of bias
Erenler, 2014	No	Yes	No	Yes	No	No	Yes	Yes	Yes	Yes	Humans	Moderate risk of bias
Erenler, 2014	No	Yes	No	Yes	No	No	Yes	Yes	Yes	Yes	Humans	Moderate risk of bias
Erenler, 2015	No	Yes	No	Yes	Yes	No	Yes	Yes	Yes	Yes	Humans	Low risk of bias
Erenler, 2015	No	Yes	No	Yes	Yes	No	Yes	Yes	Yes	Yes	Humans	Low risk of bias
Ergönül, 2004	No	Yes	No	Yes	Yes	No	Yes	Yes	No	Yes	Humans	Moderate risk of bias
Ergönül, 2004	No	Yes	No	Yes	Yes	No	Yes	Yes	No	Yes	Humans	Moderate risk of bias
Ergönül, 2004	No	Yes	No	Yes	Yes	No	Yes	Yes	No	Yes	Humans	Moderate risk of bias
Ergonul, 2006	No	Yes	No	Yes	Yes	No	Yes	Yes	No	Yes	Humans	Moderate risk of bias
Ergonul, 2006	No	Yes	No	Yes	Yes	No	Yes	Yes	No	Yes	Humans	Moderate risk of bias
Ergonul, 2006	No	Yes	No	Yes	Yes	No	Yes	Yes	No	Yes	Humans	Moderate risk of bias
Ergönül, 2006	No	Yes	No	Yes	Yes	No	Yes	Yes	Yes	Yes	Humans	Low risk of bias
Ergonul, 2007	No	Yes	No	Yes	No	No	Yes	Yes	No	Yes	Humans	Moderate risk of bias
Ergonul, 2007	No	Yes	No	Yes	No	No	Yes	Yes	No	Yes	Humans	Moderate risk of bias
Ertugrul, 2009	No	Yes	No	Yes	Yes	No	Yes	Yes	Yes	Yes	Humans	Low risk of bias
Ertugrul, 2009	No	Yes	No	Yes	Yes	No	Yes	Yes	Yes	Yes	Humans	Low risk of bias
Ertugrul, 2012	No	Yes	No	Yes	Yes	No	Yes	Yes	Unclear	Yes	Humans	Moderate risk of bias

Estrada-Peña, 2012	No	Yes	No	Not applicable	No	Unclear	Yes	Yes	No	Yes	Individual tick	Moderate risk of bias
Faghihi, 2018	No	Yes	No	Not applicable	No	No	Yes	Yes	Yes	Yes	Other animals	Moderate risk of bias
Faghihi, 2018	No	Yes	No	Not applicable	No	No	Yes	Yes	Yes	Yes	Other animals	Moderate risk of bias
Faghihi, 2018	No	Yes	No	Not applicable	No	No	Yes	Yes	Yes	Yes	Individual tick	Moderate risk of bias
Faghihi, 2018	No	Yes	No	Not applicable	No	No	Yes	Yes	Yes	Yes	Individual tick	Moderate risk of bias
Faghihi, 2018	No	Yes	No	Not applicable	No	No	Yes	Yes	Yes	Yes	Other animals	Moderate risk of bias
Fajs, 2014	No	Yes	No	Yes	Yes	No	Yes	Yes	Yes	Yes	Humans	Low risk of bias
Fajs, 2014	No	Yes	No	Not applicable	No	No	Yes	Yes	Yes	Yes	Other animals	Moderate risk of bias
Fajs, 2014	No	Yes	No	Not applicable	No	No	Yes	Yes	Yes	Yes	Other animals	Moderate risk of bias
Fajs, 2014	No	Yes	No	Not applicable	No	No	Yes	Yes	Yes	Yes	Other animals	Moderate risk of bias
Fajs, 2014	No	Yes	No	Not applicable	No	No	Yes	Yes	Yes	Yes	Individual tick	Moderate risk of bias
Fakoorziba, 2006	No	Yes	No	Yes	Yes	No	Yes	Yes	Yes	Yes	Humans	Low risk of bias
Fakoorziba, 2006	No	Yes	No	Yes	Yes	No	Yes	Yes	Yes	Yes	Humans	Low risk of bias
Fakoorziba, 2012	No	Yes	Yes	Not applicable	No	No	Yes	Yes	No	Yes	Individual tick	Moderate risk of bias
Fakoorziba, 2012	No	Yes	Yes	Not applicable	No	No	Yes	Yes	No	Yes	Individual tick	Moderate risk of bias
Fakoorziba, 2015	No	Yes	Yes	Not applicable	No	No	Yes	Yes	No	Yes	Individual tick	Moderate risk of bias
Fakoorziba, 2015	No	Yes	Yes	Not applicable	No	No	Yes	Yes	No	Yes	Individual tick	Moderate risk of bias
Fares, 2019	No	Yes	Yes	Not applicable	No	No	Yes	Yes	No	Yes	Individual tick	Moderate risk of bias
Farhadpour, 2016	No	Yes	Yes	Not applicable	No	No	Yes	Yes	No	Yes	Individual tick	Moderate risk of bias
Farhadpour, 2016	No	Yes	Yes	Not applicable	No	No	Yes	Yes	No	Yes	Individual tick	Moderate risk of bias
Farhadpour, 2016	No	Yes	Yes	Not applicable	No	No	Yes	Yes	No	Yes	Individual tick	Moderate risk of bias
Farhadpour, 2016	No	Yes	Yes	Not applicable	No	No	Yes	Yes	No	Yes	Individual tick	Moderate risk of bias
Fernández de Mera, 2017	No	Yes	No	Not applicable	No	No	Yes	Yes	Unclear	Yes	Individual tick	Moderate risk of bias
Fernández de Mera, 2017	No	Yes	No	Not applicable	No	No	Yes	Yes	Unclear	Yes	Individual tick	Moderate risk of bias
Fernández de Mera, 2017	No	Yes	No	Not applicable	No	No	Yes	Yes	Unclear	Yes	Individual tick	Moderate risk of bias
Fernández de Mera, 2017	No	Yes	No	Not applicable	No	No	Yes	Yes	Unclear	Yes	Individual tick	Moderate risk of bias
Filipe, 1985	No	Yes	No	Yes	Yes	No	Yes	Yes	Unclear	Yes	Humans	Moderate risk of bias
Filipe, 1985	No	Yes	No	Not applicable	No	No	Yes	Yes	Unclear	Yes	Other animals	Moderate risk of bias
Fisher-Hoch, 1992	No	Yes	No	Yes	No	No	Yes	Yes	No	Yes	Humans	Moderate risk of bias
Fisher-Hoch, 1992	No	Yes	No	Yes	No	No	Yes	Yes	No	Yes	Humans	Moderate risk of bias
Fisher-Hoch, 1992	No	Yes	No	Not applicable	No	No	Yes	Yes	No	Yes	Other animals	Moderate risk of bias
Fisher-Hoch, 1992	No	Yes	No	Not applicable	No	No	Yes	Yes	No	Yes	Other animals	Moderate risk of bias
Fisher-Hoch, 1992	No	Yes	No	Not applicable	No	No	Yes	Yes	No	Yes	Other animals	Moderate risk of bias
Földes, 2019	No	Yes	No	Not applicable	No	Unclear	Yes	Yes	Yes	Yes	Other animals	Moderate risk of bias
Földes, 2019	No	Yes	No	Not applicable	No	Unclear	Yes	Yes	Yes	Yes	Other animals	Moderate risk of bias
Földes, 2019	No	Yes	No	Not applicable	No	Unclear	Yes	Yes	Yes	Yes	Other animals	Moderate risk of bias
Földes, 2019	No	Yes	No	Not applicable	No	Unclear	Yes	Yes	Yes	Yes	Other animals	Moderate risk of bias
Gadia, 2017	No	Yes	Yes	Yes	Unclear	Unclear	Yes	Yes	Yes	Yes	Humans	Low risk of bias
Gambhir, 2019	No	Yes	No	Yes	Unclear	Unclear	Yes	Yes	No	Yes	Humans	Moderate risk of bias
Gandhi, 2011	No	Yes	No	Yes	Yes	No	Yes	Yes	No	Yes	Humans	Moderate risk of bias
Gargili, 2011	No	Yes	No	Yes	No	Unclear	Yes	Yes	Yes	Yes	Humans	Moderate risk of bias
Gargili, 2011	No	Yes	No	Yes	No	Unclear	Yes	Yes	Yes	Yes	Humans	Moderate risk of bias
Gargili, 2011	No	Yes	No	Not applicable	No	Unclear	Yes	Yes	Yes	Yes	Tick pools	Moderate risk of bias
Gazi, 2016	No	Yes	Yes	Yes	No	Unclear	Yes	Yes	No	Yes	Humans	Moderate risk of bias
Gergova, 2012	No	Yes	No	Not applicable	No	Unclear	Yes	Yes	Yes	Yes	Individual tick	Moderate risk of bias
Gergova, 2012	No	Yes	No	Not applicable	No	Unclear	Yes	Yes	Yes	Yes	Individual tick	Moderate risk of bias
Gergova, 2012	No	Yes	No	Not applicable	No	Unclear	Yes	Yes	Yes	Yes	Individual tick	Moderate risk of bias
Gergova, 2012	No	Yes	No	Not applicable	No	Unclear	Yes	Yes	Yes	Yes	Individual tick	Moderate risk of bias
Gergova, 2012	No	Yes	No	Not applicable	No	Unclear	Yes	Yes	Yes	Yes	Individual tick	Moderate risk of bias
Gergova, 2012	No	Yes	No	Not applicable	No	Unclear	Yes	Yes	Yes	Yes	Individual tick	Moderate risk of bias
Gergova, 2012	No	Yes	No	Not applicable	No	Unclear	Yes	Yes	Yes	Yes	Individual tick	Moderate risk of bias
Gergova, 2012	No	Yes	No	Not applicable	No	Unclear	Yes	Yes	Yes	Yes	Individual tick	Moderate risk of bias
Gergova, 2013	No	Yes	No	Not applicable	No	Unclear	Yes	Yes	Yes	Yes	Other animals	Moderate risk of bias
Gergova, 2013	No	Yes	No	Not applicable	No	Unclear	Yes	Yes	Yes	Yes	Individual tick	Moderate risk of bias
Gergova, 2013	No	Yes	No	Not applicable	No	Unclear	Yes	Yes	Yes	Yes	Individual tick	Moderate risk of bias
Gergova, 2013	No	Yes	No	Not applicable	No	Unclear	Yes	Yes	Yes	Yes	Individual tick	Moderate risk of bias
Gergova, 2014	No	Yes	No	Yes	No	No	Yes	Yes	Yes	Yes	Humans	Moderate risk of bias
Gevorgyan, 2019	No	Yes	Yes	Not applicable	No	Unclear	Yes	Yes	No	Yes	Individual tick	Moderate risk of bias
Gevorgyan, 2019	No	Yes	Yes	Not applicable	No	Unclear	Yes	Yes	No	Yes	Individual tick	Moderate risk of bias
Gevorgyan, 2019	No	Yes	Yes	Not applicable	No	Unclear	Yes	Yes	No	Yes	Individual tick	Moderate risk of bias
Gevorgyan, 2019	No	Yes	Yes	Not applicable	No	Unclear	Yes	Yes	No	Yes	Individual tick	Moderate risk of bias
Gevorgyan, 2019	No	Yes	Yes	Not applicable	No	Unclear	Yes	Yes	No	Yes	Individual tick	Moderate risk of bias
Gevorgyan, 2019	No	Yes	Yes	Not applicable	No	Unclear	Yes	Yes	No	Yes	Individual tick	Moderate risk of bias
Gonzalan, 2007	No	Yes	No	Yes	Yes	No	Yes	Yes	Yes	Yes	Humans	Low risk of bias
Gonzalan, 2007	No	Yes	No	Yes	Yes	No	Yes	Yes	Yes	Yes	Humans	Low risk of bias
Gonzalan, 2007	No	Yes	No	Yes	Yes	No	Yes	Yes	Yes	Yes	Humans	Low risk of bias
Gonzalez, 1989	No	Yes	Yes	Yes	No	Unclear	Yes	Yes	Yes	Yes	Humans	Low risk of bias
Gonzalez, 1989	No	Yes	Yes	Yes	No	Unclear	Yes	Yes	Yes	Yes	Humans	Low risk of bias
Gonzalez, 1989	No	Yes	Yes	Yes	No	Unclear	Yes	Yes	Yes	Yes	Humans	Low risk of bias
Gonzalez, 1989	No	Yes	Yes	Yes	No	Unclear	Yes	Yes	Yes	Yes	Humans	Low risk of bias
Gonzalez, 1989	No	Yes	Yes	Yes	No	Unclear	Yes	Yes	Yes	Yes	Humans	Low risk of bias
Gonzalez, 1989	No	Yes	Yes	Yes	No	Unclear	Yes	Yes	Yes	Yes	Humans	Low risk of bias
Gonzalez, 1990	No	Yes	No	Yes	No	No	Yes	Yes	No	Yes	Humans	Moderate risk of bias
Gonzalez, 1990	No	Yes	No	Yes	No	No	Yes	Yes	No	Yes	Humans	Moderate risk of bias
Gonzalez, 1990	No	Yes	No	Not applicable	No	No	Yes	Yes	No	Yes	Other animals	Moderate risk of bias
Gonzalez, 1990	No	Yes	No	Not applicable	No	No	Yes	Yes	No	Yes	Other animals	Moderate risk of bias
Gozdas, 2014	No	Yes	No	Yes	Yes	No	Yes	Yes	Yes	Yes	Humans	Low risk of bias
Gozdas, 2014	No	Yes	No	Yes	Yes	No	Yes	Yes	Yes	Yes	Humans	Low risk of bias
Gozdas, 2019	No	Yes	No	Yes	No	Yes	Yes	Yes	Yes	Yes	Humans	Low risk of bias
Gozdas, 2019	No	Yes	No	Yes	No	Yes	Yes	Yes	Yes	Yes	Humans	Low risk of bias
Gozek, 2013	No	Yes	No	Yes	No	No	Yes	Yes	Yes	Yes	Humans	Moderate risk of bias
Gozek, 2013	No	Yes	No	Yes	No	No	Yes	Yes	Yes	Yes	Humans	Moderate risk of bias
Grech-Angelini, 2019	No	Yes	No	Not applicable	No	Unclear	Yes	Yes	Yes	Yes	Individual tick	Moderate risk of bias
Greiner, 2016	No	Yes	Yes	Yes	No	No	Yes	Yes	No	Yes	Humans	Moderate risk of bias
Greiner, 2016	No	Yes	Yes	Yes	No	No	Yes	Yes	No	Yes	Humans	Moderate risk of bias
Guilherme, 1996	No	Yes	No	Not applicable	No	Unclear	Yes	Yes	Yes	Yes	Other animals	Moderate risk of bias
Gul, 2015	No	Yes	No	Yes	No	Unclear	Yes	Yes	Unclear	Yes	Humans	Moderate risk of bias
Gunes, 2009	No	Yes	Yes	Yes	No	No	Yes	Yes	No	Yes	Humans	Moderate risk of bias
Gunes, 2009	No	Yes	Yes	Yes	No	No	Yes	Yes	No	Yes	Humans	Moderate risk of bias
Gunes, 2011	No	Yes	Yes	Not applicable	No	No	Yes	Yes	No	Yes	Tick pools	Moderate risk of bias
Gunes, 2011	No	Yes	Yes	Not applicable	No	No	Yes	Yes	No	Yes	Individual tick	Moderate risk of bias
Gunes, 2011	No	Yes	Yes	Not applicable	No	No	Yes	Yes	No	Yes	Individual tick	Moderate risk of bias
Gunes, 2011	No	Yes	Yes	Not applicable	No	No	Yes	Yes	No	Yes	Individual tick	Moderate risk of bias
Gunes, 2011	No	Yes	Yes	Not applicable	No	No	Yes	Yes	No	Yes	Individual tick	Moderate risk of bias

Gunes, 2011	No	Yes	Yes	Not applicable	No	No	Yes	Yes	No	Yes	Individual tick	Moderate risk of bias
Hasan, 2014	No	Yes	No	Yes	Yes	No	Yes	Yes	Yes	Yes	Humans	Low risk of bias
Hassanein, 1997	No	Yes	Yes	Yes	No	No	Yes	Yes	Yes	Yes	Humans	Low risk of bias
Hassanein, 1997	No	Yes	Yes	Not applicable	No	No	Yes	Yes	Yes	Yes	Other animals	Moderate risk of bias
Hassanein, 1997	No	Yes	Yes	Not applicable	No	No	Yes	Yes	Yes	Yes	Other animals	Moderate risk of bias
Hassanein, 1997	No	Yes	Yes	Not applicable	No	No	Yes	Yes	Yes	Yes	Other animals	Moderate risk of bias
Hassanein, 1997	No	Yes	Yes	Not applicable	No	No	Yes	Yes	Yes	Yes	Other animals	Moderate risk of bias
Hassanein, 2000	No	Yes	No	Not applicable (for animals)	No	No	Yes	Yes	No	Yes	Tick pools	Moderate risk of bias
Hassaniazad, 2016	No	Yes	No	Yes	Yes	No	Yes	Yes	Yes	Yes	Humans	Low risk of bias
Hatami, 2019	No	Yes	No	Yes	No	No	Yes	Yes	No	Yes	Humans	Moderate risk of bias
Hatami, 2019	No	Yes	No	Yes	No	No	Yes	Yes	No	Yes	Humans	Moderate risk of bias
Hatipoglu, 2010	No	Yes	No	Yes	No	No	Yes	Yes	Yes	Yes	Humans	Moderate risk of bias
Hatipoglu, 2010	No	Yes	No	Yes	No	No	Yes	Yes	Yes	Yes	Humans	Moderate risk of bias
Hatipoglu, 2010	No	Yes	No	Yes	No	No	Yes	Yes	Yes	Yes	Humans	Moderate risk of bias
Head, 2020	No	Yes	Yes	Yes	Yes	No	Yes	Yes	Yes	Yes	Humans	Low risk of bias
Head, 2020	No	Yes	Yes	Yes	Yes	No	Yes	Yes	Yes	Yes	Humans	Low risk of bias
Hekimoglu, 2012	No	Yes	No	Not applicable (for animals)	No	No	Yes	Yes	No	Yes	Tick pools	Moderate risk of bias
Hekimoğlu, 2014	No	Yes	No	Yes	No	No	Yes	Yes	Yes	Yes	Humans	Moderate risk of bias
Holakouie, 2004	No	Yes	Yes	Yes	No	Yes	Yes	Yes	No	Yes	Humans	Low risk of bias
Holakouie, 2004	No	Yes	Yes	Yes	No	Yes	Yes	Yes	No	Yes	Humans	Low risk of bias
Horton, 2014	No	Yes	No	Not applicable	No	No	Yes	Yes	Yes	Yes	Other animals	Moderate risk of bias
Horton, 2014	No	Yes	No	Not applicable	No	No	Yes	Yes	Yes	Yes	Other animals	Moderate risk of bias
Horton, 2014	No	Yes	No	Not applicable	No	No	Yes	Yes	Yes	Yes	Other animals	Moderate risk of bias
Horton, 2014	No	Yes	No	Not applicable	No	No	Yes	Yes	Yes	Yes	Other animals	Moderate risk of bias
Horton, 2016	No	Yes	No	Not applicable	No	No	Yes	Yes	No	Yes	Individual tick	Moderate risk of bias
Horton, 2016	No	Yes	No	Not applicable	No	No	Yes	Yes	No	Yes	Individual tick	Moderate risk of bias
Horton, 2016	No	Yes	No	Not applicable	No	No	Yes	Yes	No	Yes	Individual tick	Moderate risk of bias
Horton, 2016	No	Yes	No	Not applicable	No	No	Yes	Yes	No	Yes	Individual tick	Moderate risk of bias
Horton, 2016	No	Yes	No	Not applicable	No	No	Yes	Yes	No	Yes	Individual tick	Moderate risk of bias
Horton, 2016	No	Yes	No	Not applicable	No	No	Yes	Yes	No	Yes	Individual tick	Moderate risk of bias
Horton, 2016	No	Yes	No	Not applicable	No	No	Yes	Yes	No	Yes	Individual tick	Moderate risk of bias
Horton, 2016	No	Yes	No	Not applicable	No	No	Yes	Yes	No	Yes	Individual tick	Moderate risk of bias
Horváth, 1976	No	Yes	No	Yes	Yes	No	Yes	Yes	Unclear	Yes	Humans	Moderate risk of bias
Horváth, 1976	No	Yes	No	Not applicable	No	No	Yes	Yes	Unclear	Yes	Other animals	Moderate risk of bias
Hosseini-Vasoukolaei, 2016	No	Yes	No	Yes	Yes	No	Yes	Yes	Yes	Yes	Humans	Low risk of bias
Hosseini-Vasoukolaei, 2016	No	Yes	No	Not applicable	No	No	Yes	Yes	Yes	Yes	Individual tick	Moderate risk of bias
Hosseini-Vasoukolaei, 2016	No	Yes	No	Not applicable	No	No	Yes	Yes	Yes	Yes	Other animals	Moderate risk of bias
Ibrahim, 2015	No	Yes	Yes	Not applicable	No	No	Yes	Yes	Unclear	Yes	Other animals	Moderate risk of bias
Izadi, 2006	No	Yes	Yes	Yes	Yes	No	Yes	Yes	No	Yes	Humans	Low risk of bias
Izadi, 2008	No	Yes	No	Yes	No	Yes	Yes	Yes	No	Yes	Humans	Moderate risk of bias
Izadi, 2008	No	Yes	No	Yes	No	Yes	Yes	Yes	No	Yes	Humans	Moderate risk of bias
Izadi, 2009	No	Yes	No	Yes	Yes	Unclear	Yes	Yes	Yes	Yes	Humans	Low risk of bias
Johnson, 1983	No	Yes	No	Yes	No	Unclear	Yes	Yes	Unclear	Yes	Humans	Moderate risk of bias
Johnson, 1983	No	Yes	No	Yes	No	Unclear	Yes	Yes	Yes	Yes	Humans	Moderate risk of bias
Johnson, 1993	No	Yes	No	Yes	No	No	Yes	Yes	Unclear	Yes	Humans	Moderate risk of bias
Kadanali, 2012	No	Yes	No	Yes	Yes	No	Yes	Yes	Yes	Yes	Humans	Low risk of bias
Kadanali, 2012	No	Yes	No	Yes	Yes	No	Yes	Yes	Yes	Yes	Humans	Low risk of bias
Kadanali, 2012	No	Yes	No	Yes	Yes	No	Yes	Yes	Yes	Yes	Humans	Low risk of bias
Kadanali, 2012	No	Yes	No	Yes	Yes	No	Yes	Yes	Yes	Yes	Humans	Low risk of bias
Kalvatchev, 2010	No	Yes	No	Yes	No	No	Yes	Yes	Unclear	Yes	Humans	Moderate risk of bias
Kalvatchev, 2010	No	Yes	No	Yes	No	No	Yes	Yes	Unclear	Yes	Humans	Moderate risk of bias
Kara, 2016	No	Yes	No	Yes	Yes	No	Yes	Yes	No	Yes	Humans	Moderate risk of bias
Kara, 2016	No	Yes	No	Yes	Yes	No	Yes	Yes	No	Yes	Humans	Moderate risk of bias
Karakecili, 2018	No	Yes	No	Yes	No	No	Yes	Yes	Yes	Yes	Humans	Moderate risk of bias
Karakus, 2019	No	Yes	No	Yes	Yes	No	Yes	Yes	Unclear	Yes	Humans	Moderate risk of bias
Karlberg, 2015	No	Yes	No	Yes	Yes	No	Yes	Yes	Unclear	Yes	Humans	Moderate risk of bias
Karlberg, 2015	No	Yes	No	Yes	Yes	No	Yes	Yes	Unclear	Yes	Humans	Moderate risk of bias
Karlberg, 2015	No	Yes	No	Yes	Yes	No	Yes	Yes	Unclear	Yes	Humans	Moderate risk of bias
Kasi, 2020	No	Yes	Yes	Not applicable	No	No	Yes	Yes	No	Yes	Individual tick	Moderate risk of bias
Kasi, 2020	No	Yes	Yes	Not applicable	No	No	Yes	Yes	No	Yes	Individual tick	Moderate risk of bias
Kasi, 2020	No	Yes	Yes	Not applicable	No	No	Yes	Yes	No	Yes	Individual tick	Moderate risk of bias
Kasi, 2020	No	Yes	Yes	Not applicable	No	No	Yes	Yes	No	Yes	Individual tick	Moderate risk of bias
Kasi, 2020	No	Yes	Yes	Not applicable	No	No	Yes	Yes	No	Yes	Individual tick	Moderate risk of bias
Kasi, 2020	No	Yes	Yes	Not applicable	No	No	Yes	Yes	No	Yes	Other animals	Moderate risk of bias
Kasi, 2020	No	Yes	Yes	Not applicable	No	No	Yes	Yes	No	Yes	Other animals	Moderate risk of bias
Kautman, 2016	No	Yes	No	Not applicable	No	No	Yes	Yes	Yes	Yes	Individual tick	Moderate risk of bias
Kayedi, 2015	No	Yes	Yes	Not applicable	No	No	Yes	Yes	No	Yes	Individual tick	Moderate risk of bias
Kayedi, 2015	No	Yes	Yes	Not applicable	No	No	Yes	Yes	No	Yes	Individual tick	Moderate risk of bias
Kayedi, 2015	No	Yes	Yes	Not applicable	No	No	Yes	Yes	No	Yes	Individual tick	Moderate risk of bias
Kayedi, 2015	No	Yes	Yes	Not applicable	No	No	Yes	Yes	No	Yes	Individual tick	Moderate risk of bias
Kayedi, 2015	No	Yes	Yes	Not applicable	No	No	Yes	Yes	No	Yes	Individual tick	Moderate risk of bias
Khan, 1997	No	Yes	No	Yes	Yes	No	Yes	Yes	No	Yes	Humans	Moderate risk of bias
Khan, 1997	No	Yes	No	Yes	Yes	No	Yes	Yes	No	Yes	Humans	Moderate risk of bias
Khan, 1997	No	Yes	No	Yes	Yes	No	Yes	Yes	No	Yes	Humans	Moderate risk of bias
Khan, 1997	No	Yes	No	Yes	Yes	No	Yes	Yes	No	Yes	Humans	Moderate risk of bias
Khan, 1997	No	Yes	No	Yes	Yes	No	Yes	Yes	No	Yes	Humans	Moderate risk of bias
Khan, 1997	No	Yes	No	Yes	Yes	No	Yes	Yes	No	Yes	Humans	Moderate risk of bias
Khan, 1997	No	Yes	No	Yes	Yes	No	Yes	Yes	No	Yes	Humans	Moderate risk of bias
Khan, 1997	No	Yes	No	Yes	Yes	No	Yes	Yes	No	Yes	Humans	Moderate risk of bias
Khan, 1997	No	Yes	No	Not applicable	No	No	Yes	Yes	No	Yes	Other animals	Moderate risk of bias
Khan, 1997	No	Yes	No	Not applicable	No	No	Yes	Yes	No	Yes	Other animals	Moderate risk of bias
Khan, 1997	No	Yes	No	Not applicable	No	No	Yes	Yes	No	Yes	Other animals	Moderate risk of bias
Khan, 1997	No	Yes	No	Not applicable	No	No	Yes	Yes	No	Yes	Individual tick	Moderate risk of bias
Khan, 1997	No	Yes	No	Not applicable	No	No	Yes	Yes	No	Yes	Other animals	Moderate risk of bias
Khurshid, 2015	No	Yes	No	Yes	Yes	No	Yes	Yes	No	Yes	Humans	Moderate risk of bias
Khurshid, 2015	No	Yes	No	Yes	Yes	No	Yes	Yes	No	Yes	Humans	Moderate risk of bias
Khurshid, 2015	No	Yes	No	Yes	Yes	No	Yes	Yes	No	Yes	Humans	Moderate risk of bias
Khurshid, 2015	No	Yes	No	Yes	Yes	No	Yes	Yes	No	Yes	Humans	Moderate risk of bias
Kilinc, 2016	No	Yes	No	Yes	No	No	Yes	Yes	Yes	Yes	Humans	Moderate risk of bias
Kilinc, 2016	No	Yes	No	Yes	No	No	Yes	Yes	Yes	Yes	Humans	Moderate risk of bias
Kizilgun, 2013	No	Yes	No	Yes	Yes	No	Yes	Yes	No	Yes	Humans	Moderate risk of bias
Koksal, 2014	No	Yes	No	Yes	No	Unclear	Yes	Yes	Yes	Yes	Humans	Moderate risk of bias
Kuchuloria, 2014	No	Yes	No	Yes	No	Unclear	Yes	Yes	Yes	Yes	Humans	Moderate risk of bias
Kulichenko, 2016	No	Yes	No	Not applicable	No	Unclear	Yes	Yes	No	Yes	Tick pools	Moderate risk of bias

Lani, 2015	No	Yes	No	Yes	No	Unclear	Yes	Yes	Yes	Yes	Humans	Moderate risk of bias
Leblebicioglu, 2014	No	Yes	No	Not applicable	No	No	Yes	Yes	Yes	Yes	Individual tick	Moderate risk of bias
Leblebicioglu, 2014	No	Yes	No	Not applicable	No	No	Yes	Yes	Yes	Yes	Individual tick	Moderate risk of bias
Leblebicioglu, 2016	No	Yes	No	Yes	Yes	Yes	Yes	Yes	Yes	Yes	Humans	Low risk of bias
Leblebicioglu, 2016	No	Yes	No	Yes	Yes	Yes	Yes	Yes	Yes	Yes	Humans	Low risk of bias
Lepers, 1988	No	Yes	No	Yes	No	Unclear	Yes	Yes	No	Yes	Humans	Moderate risk of bias
Lotfollahzadeh, 2011	No	Yes	Yes	Not applicable	No	Yes	Yes	Yes	Yes	Yes	Other animals	Low risk of bias
Lwande, 2012	No	Yes	No	Yes	No	Unclear	Yes	Yes	No	Yes	Humans	Moderate risk of bias
Lwande, 2012	No	Yes	No	Yes	No	Unclear	Yes	Yes	No	Yes	Humans	Moderate risk of bias
Magnaval, 2011	No	Yes	No	Yes	No	Unclear	Yes	Yes	Yes	Yes	Humans	Moderate risk of bias
Mahzounieh, 2012	No	Yes	No	Not applicable	No	No	Yes	Yes	Unclear	Yes	Other animals	Moderate risk of bias
Mahzounieh, 2012	No	Yes	No	Not applicable	No	No	Yes	Yes	Unclear	Yes	Other animals	Moderate risk of bias
Maiga, 2017	No	Yes	No	Not applicable	No	Unclear	Yes	Yes	Yes	Yes	Other animals	Moderate risk of bias
Majeed, 2012	Yes	Yes	No	Yes	Yes	Unclear	Yes	Yes	Yes	Yes	Humans	Low risk of bias
Majeed, 2012	Yes	Yes	No	Yes	Yes	Unclear	Yes	Yes	Yes	Yes	Humans	Low risk of bias
Malik, 2011	No	Yes	No	Yes	No	No	Yes	Yes	No	Yes	Humans	Moderate risk of bias
Maltezou, 2009	No	Yes	No	Yes	Yes	No	Yes	Yes	Yes	Yes	Humans	Low risk of bias
Mancini, 2013	No	Yes	No	Not applicable	No	No	Yes	Yes	Yes	Yes	Individual tick	Moderate risk of bias
Mancuso, 2019	No	Yes	No	Not applicable	No	No	Yes	Yes	No	Yes	Individual tick	Moderate risk of bias
Mardani, 2007	No	Yes	No	Yes	Yes	No	Yes	Yes	Yes	Yes	Humans	Low risk of bias
Mardani, 2007	No	Yes	No	Yes	Yes	No	Yes	Yes	Yes	Yes	Humans	Low risk of bias
Mariner, 1995	No	Yes	Yes	Not applicable	No	No	Yes	Yes	Yes	Yes	Other animals	Moderate risk of bias
Mariner, 1995	No	Yes	Yes	Not applicable	No	No	Yes	Yes	Yes	Yes	Other animals	Moderate risk of bias
Mariner, 1995	No	Yes	Yes	Not applicable	No	No	Yes	Yes	Yes	Yes	Other animals	Moderate risk of bias
Mariner, 1995	No	Yes	Yes	Not applicable	No	No	Yes	Yes	Yes	Yes	Other animals	Moderate risk of bias
Mariner, 1995	No	Yes	Yes	Not applicable	No	No	Yes	Yes	Yes	Yes	Other animals	Moderate risk of bias
Mathiot, 1988	No	Yes	No	Not applicable	No	No	Yes	Yes	Yes	Yes	Tick pools	Moderate risk of bias
Mathiot, 1989	No	Yes	No	Yes	Yes	No	Yes	Yes	Unclear	Yes	Humans	Moderate risk of bias
McCarthy, 1996	No	Yes	No	Yes	Yes	No	Yes	Yes	No	Yes	Humans	Moderate risk of bias
Mehravaran, 2013	No	Yes	Yes	Not applicable	No	No	Yes	Yes	Unclear	Yes	Individual tick	Moderate risk of bias
Mehravaran, 2013	No	Yes	Yes	Not applicable	No	No	Yes	Yes	Unclear	Yes	Individual tick	Moderate risk of bias
Mehravaran, 2013	No	Yes	Yes	Not applicable	No	No	Yes	Yes	Unclear	Yes	Individual tick	Moderate risk of bias
Mehravaran, 2013	No	Yes	Yes	Not applicable	No	No	Yes	Yes	Unclear	Yes	Individual tick	Moderate risk of bias
Mehravaran, 2013	No	Yes	Yes	Not applicable	No	No	Yes	Yes	Unclear	Yes	Individual tick	Moderate risk of bias
Mehravaran, 2013	No	Yes	Yes	Not applicable	No	No	Yes	Yes	Unclear	Yes	Individual tick	Moderate risk of bias
Mehravaran, 2013	No	Yes	Yes	Not applicable	No	No	Yes	Yes	Unclear	Yes	Individual tick	Moderate risk of bias
Mehravaran, 2013	No	Yes	Yes	Not applicable	No	No	Yes	Yes	Unclear	Yes	Individual tick	Moderate risk of bias
Memish, 2011	No	Yes	Yes	Yes	No	No	Yes	Yes	No	Yes	Humans	Moderate risk of bias
Mertens, 2016	No	Yes	Yes	Not applicable	No	No	Yes	Yes	Yes	Yes	Other animals	Moderate risk of bias
Mertens, 2016	No	Yes	Yes	Not applicable	No	No	Yes	Yes	Yes	Yes	Other animals	Moderate risk of bias
Midilli, 2007	No	Yes	No	Yes	Yes	No	Yes	Yes	No	Yes	Humans	Moderate risk of bias
Midilli, 2007	No	Yes	No	Yes	Yes	No	Yes	Yes	No	Yes	Humans	Moderate risk of bias
Midilli, 2009	No	Yes	No	Yes	No	No	Yes	Yes	No	Yes	Humans	Moderate risk of bias
Midilli, 2009	No	Yes	No	Yes	No	No	Yes	Yes	No	Yes	Humans	Moderate risk of bias
Midilli, 2009	No	Yes	No	Not applicable	No	No	Yes	Yes	No	Yes	Individual tick	Moderate risk of bias
Midilli, 2009	No	Yes	No	Not applicable	No	No	Yes	Yes	No	Yes	Individual tick	Moderate risk of bias
Mofleh, 2012	No	Yes	No	Yes	Yes	No	Yes	Yes	No	Yes	Humans	Moderate risk of bias
Mohamed, 2008	No	Yes	Yes	Not applicable	No	No	Yes	Yes	Unclear	Yes	Other animals	Moderate risk of bias
Mohamed, 2008	No	Yes	Yes	Not applicable	No	No	Yes	Yes	Unclear	Yes	Other animals	Moderate risk of bias
Mohamed, 2008	No	Yes	Yes	Not applicable	No	No	Yes	Yes	Unclear	Yes	Other animals	Moderate risk of bias
Mohamed, 2008	No	Yes	Yes	Not applicable	No	No	Yes	Yes	Unclear	Yes	Other animals	Moderate risk of bias
Mohammadian, 2016	No	Yes	Yes	Not applicable	No	No	Yes	Yes	Yes	Yes	Individual tick	Moderate risk of bias
Mohammadian, 2016	No	Yes	Yes	Not applicable	No	No	Yes	Yes	Yes	Yes	Individual tick	Moderate risk of bias
Mohammadian, 2016	No	Yes	Yes	Not applicable	No	No	Yes	Yes	Yes	Yes	Individual tick	Moderate risk of bias
Mohd Shukri, 2015	No	Yes	No	Yes	No	No	Yes	Yes	No	Yes	Humans	Moderate risk of bias
Moming, 2018	No	Yes	No	Not applicable	No	No	Yes	Yes	No	Yes	Tick pools	Moderate risk of bias
Morrill, 1990	No	Yes	No	Not applicable	No	No	Yes	Yes	Yes	Yes	Other animals	Moderate risk of bias
Morrill, 1990	No	Yes	No	Not applicable	No	No	Yes	Yes	Yes	Yes	Other animals	Moderate risk of bias
Morrill, 1990	No	Yes	No	Not applicable	No	No	Yes	Yes	Yes	Yes	Other animals	Moderate risk of bias
Mostafavi, 2012	No	Yes	No	Not applicable	No	No	Yes	Yes	Yes	Yes	Other animals	Moderate risk of bias
Mostafavi, 2013	No	Yes	No	Not applicable	No	No	Yes	Yes	Yes	Yes	Other animals	Moderate risk of bias
Mostafavi, 2013	No	Yes	No	Not applicable	No	No	Yes	Yes	Yes	Yes	Other animals	Moderate risk of bias
Mostafavi, 2013	No	Yes	No	Not applicable	No	No	Yes	Yes	Yes	Yes	Other animals	Moderate risk of bias
Mostafavi, 2014	No	Yes	No	Yes	Yes	No	Yes	Yes	Yes	Yes	Humans	Low risk of bias
Mostafavi, 2014	No	Yes	No	Yes	Yes	No	Yes	Yes	Yes	Yes	Humans	Low risk of bias
Mostafavi, 2017	No	Yes	Yes	Yes	No	No	Yes	Yes	Yes	Yes	Humans	Low risk of bias
Mourya, 2012	No	Yes	No	Yes	No	No	Yes	Yes	No	Yes	Humans	Moderate risk of bias
Mourya, 2012	No	Yes	No	Not applicable	No	No	Yes	Yes	No	Yes	Tick pools	Moderate risk of bias
Mourya, 2012	No	Yes	No	Not applicable	No	No	Yes	Yes	No	Yes	Other animals	Moderate risk of bias
Mourya, 2012	No	Yes	No	Not applicable	No	No	Yes	Yes	No	Yes	Other animals	Moderate risk of bias
Mourya, 2012	No	Yes	No	Not applicable	No	No	Yes	Yes	No	Yes	Other animals	Moderate risk of bias
Mourya, 2012	No	Yes	No	Not applicable	No	No	Yes	Yes	No	Yes	Individual tick	Moderate risk of bias
Mourya, 2012	No	Yes	No	Not applicable	No	No	Yes	Yes	No	Yes	Individual tick	Moderate risk of bias
Mourya, 2012	No	Yes	No	Not applicable	No	No	Yes	Yes	No	Yes	Other animals	Moderate risk of bias
Mourya, 2012	No	Yes	No	Not applicable	No	No	Yes	Yes	No	Yes	Other animals	Moderate risk of bias
Mourya, 2012	No	Yes	No	Not applicable	No	No	Yes	Yes	No	Yes	Other animals	Moderate risk of bias
Mourya, 2014	No	Yes	Yes	Not applicable	No	No	Yes	Yes	Unclear	Yes	Other animals	Moderate risk of bias
Mourya, 2014	No	Yes	Yes	Not applicable	No	No	Yes	Yes	Unclear	Yes	Other animals	Moderate risk of bias
Mourya, 2014	No	Yes	Yes	Not applicable	No	No	Yes	Yes	Unclear	Yes	Individual tick	Moderate risk of bias
Mourya, 2014	No	Yes	Yes	Not applicable	No	No	Yes	Yes	Unclear	Yes	Other animals	Moderate risk of bias
Mourya, 2015	No	Yes	No	Not applicable	No	Unclear	Yes	Yes	Yes	Yes	Other animals	Moderate risk of bias
Mourya, 2015	No	Yes	No	Not applicable	No	Unclear	Yes	Yes	Yes	Yes	Other animals	Moderate risk of bias
Mourya, 2017	No	Yes	No	Yes	Yes	No	Yes	Yes	Yes	Yes	Humans	Low risk of bias
Mourya, 2017	No	Yes	No	Yes	Yes	No	Yes	Yes	Yes	Yes	Humans	Low risk of bias
Mourya, 2019	No	Yes	No	Yes	No	No	Yes	Yes	Yes	Yes	Humans	Moderate risk of bias
Mourya, 2019	No	Yes	No	Yes	No	No	Yes	Yes	Yes	Yes	Humans	Moderate risk of bias
Mourya, 2019	No	Yes	No	Yes	No	No	Yes	Yes	Yes	Yes	Humans	Moderate risk of bias
Muianga, 2017	No	Yes	No	Yes	No	No	Yes	Yes	Yes	Yes	Humans	Moderate risk of bias
Müller, 2016	No	Yes	No	Not applicable	No	No	Yes	Yes	Yes	Yes	Other animals	Moderate risk of bias
Müller, 2016	No	Yes	No	Not applicable	No	No	Yes	Yes	Yes	Yes	Other animals	Moderate risk of bias
Müller, 2016	No	Yes	No	Not applicable	No	No	Yes	Yes	Yes	Yes	Other animals	Moderate risk of bias
Müller, 2016	No	Yes	No	Not applicable	No	No	Yes	Yes	Yes	Yes	Other animals	Moderate risk of bias
Müller, 2016	No	Yes	No	Not applicable	No	No	Yes	Yes	Yes	Yes	Other animals	Moderate risk of bias
Mustafa, 2011	No	Yes	Yes	Yes	No	No	Yes	Yes	Yes	Yes	Humans	Low risk of bias

Mustafa, 2011	No	Yes	Yes	Not applicable	No	No	Yes	Yes	Yes	Yes	Other animals	Moderate risk of bias
Mustafa, 2011	No	Yes	Yes	Not applicable	No	No	Yes	Yes	Yes	Yes	Other animals	Moderate risk of bias
Nabeth, 2004	No	Yes	No	Yes	Yes	No	Yes	Yes	No	Yes	Humans	Moderate risk of bias
Nabeth, 2004	No	Yes	No	Yes	Yes	No	Yes	Yes	No	Yes	Humans	Moderate risk of bias
Nabeth, 2004	No	Yes	No	Not applicable	No	No	Yes	Yes	No	Yes	Tick pools	Moderate risk of bias
Nabeth, 2004	No	Yes	No	Not applicable	No	No	Yes	Yes	No	Yes	Other animals	Moderate risk of bias
Nabeth, 2004	No	Yes	No	Not applicable	No	No	Yes	Yes	No	Yes	Other animals	Moderate risk of bias
Nabeth, 2004	No	Yes	No	Not applicable	No	No	Yes	Yes	No	Yes	Individual tick	Moderate risk of bias
Nabeth, 2004	No	Yes	No	Not applicable	No	No	Yes	Yes	No	Yes	Individual tick	Moderate risk of bias
Nabeth, 2004	No	Yes	No	Not applicable	No	No	Yes	Yes	No	Yes	Individual tick	Moderate risk of bias
Nabeth, 2004	No	Yes	No	Yes	Yes	No	Yes	Yes	No	Yes	Other animals	Moderate risk of bias
Nabeth, 2004	No	Yes	No	Not applicable	No	No	Yes	Yes	No	Yes	Other animals	Moderate risk of bias
Nadeem, 2003	No	Yes	No	Yes	No	No	Yes	Yes	No	Yes	Humans	Moderate risk of bias
Nadeem, 2003	No	Yes	No	Yes	No	No	Yes	Yes	No	Yes	Humans	Moderate risk of bias
Negredo, 2019	No	Yes	No	Not applicable	No	No	Yes	Yes	Yes	Yes	Individual tick	Moderate risk of bias
Negredo, 2019	No	Yes	No	Not applicable	No	No	Yes	Yes	Yes	Yes	Individual tick	Moderate risk of bias
Negredo, 2019	No	Yes	No	Not applicable	No	No	Yes	Yes	Yes	Yes	Individual tick	Moderate risk of bias
Negredo, 2019	No	Yes	No	Not applicable	No	No	Yes	Yes	Yes	Yes	Individual tick	Moderate risk of bias
Negredo, 2019	No	Yes	No	Not applicable	No	No	Yes	Yes	Yes	Yes	Individual tick	Moderate risk of bias
Negredo, 2019	No	Yes	No	Not applicable	No	No	Yes	Yes	Yes	Yes	Individual tick	Moderate risk of bias
Németh, 2013	No	Yes	No	Not applicable	No	No	Yes	Yes	Yes	Yes	Other animals	Moderate risk of bias
Newman, 2014	No	Yes	No	Yes	No	No	Yes	Yes	Yes	Yes	Humans	Moderate risk of bias
Niazi, 2019	No	Yes	No	Yes	No	No	Yes	Yes	Yes	Yes	Humans	Moderate risk of bias
Niazi, 2019	No	Yes	No	Yes	No	No	Yes	Yes	Yes	Yes	Humans	Moderate risk of bias
Niazi, 2019	No	Yes	No	Yes	No	No	Yes	Yes	Yes	Yes	Humans	Moderate risk of bias
No author listed, 2006	No	Yes	No	Yes	No	No	Yes	Yes	No	Yes	Humans	Moderate risk of bias
No author listed, 2006	No	Yes	No	Yes	No	No	Yes	Yes	No	Yes	Humans	Moderate risk of bias
Nur, 1999	No	Yes	No	Yes	No	No	Yes	Yes	No	Yes	Humans	Moderate risk of bias
Nur, 1999	No	Yes	No	Yes	No	No	Yes	Yes	No	Yes	Humans	Moderate risk of bias
O'Hearn, 2016	No	Yes	No	Yes	No	No	Yes	Yes	Yes	Yes	Humans	Moderate risk of bias
Orkun, 2019	No	Yes	No	Not applicable	No	No	Yes	Yes	Yes	Yes	Tick pools	Moderate risk of bias
Orkun, 2019	No	Yes	No	Not applicable	No	No	Yes	Yes	Yes	Yes	Individual tick	Moderate risk of bias
Orkun, 2019	No	Yes	No	Not applicable	No	No	Yes	Yes	Yes	Yes	Individual tick	Moderate risk of bias
Orkun, 2019	No	Yes	No	Not applicable	No	No	Yes	Yes	Yes	Yes	Individual tick	Moderate risk of bias
Orkun, 2019	No	Yes	No	Not applicable	No	No	Yes	Yes	Yes	Yes	Individual tick	Moderate risk of bias
Orkun, 2019	No	Yes	No	Not applicable	No	No	Yes	Yes	Yes	Yes	Individual tick	Moderate risk of bias
Orkun, 2019	No	Yes	No	Not applicable	No	No	Yes	Yes	Yes	Yes	Individual tick	Moderate risk of bias
Ozbey, 2014	No	Yes	No	Yes	Yes	No	Yes	Yes	Yes	Yes	Humans	Low risk of bias
Ozdarendeli, 2008	No	Yes	No	Not applicable	No	No	Yes	Yes	No	Yes	Tick pools	Moderate risk of bias
Ozdarendeli, 2008	No	Yes	No	Not applicable	No	No	Yes	Yes	No	Yes	Individual tick	Moderate risk of bias
Ozdarendeli, 2008	No	Yes	No	Not applicable	No	No	Yes	Yes	No	Yes	Individual tick	Moderate risk of bias
Ozkurt, 2006	No	Yes	No	Yes	Yes	No	Yes	Yes	Yes	Yes	Humans	Low risk of bias
Ozkurt, 2006	No	Yes	No	Yes	Yes	No	Yes	Yes	Yes	Yes	Humans	Low risk of bias
Palomar, 2016	No	Yes	No	Not applicable	No	No	Yes	Yes	Yes	Yes	Individual tick	Moderate risk of bias
Palomar, 2016	No	Yes	No	Not applicable	No	No	Yes	Yes	Yes	Yes	Individual tick	Moderate risk of bias
Palomar, 2017	No	Yes	No	Yes	No	No	Yes	Yes	Yes	Yes	Humans	Moderate risk of bias
Palomar, 2017	No	Yes	No	Not applicable	No	No	Yes	Yes	Yes	Yes	Individual tick	Moderate risk of bias
Palomar, 2017	No	Yes	No	Not applicable	No	No	Yes	Yes	Yes	Yes	Individual tick	Moderate risk of bias
Palomar, 2017	No	Yes	No	Not applicable	No	No	Yes	Yes	Yes	Yes	Individual tick	Moderate risk of bias
Panayotova, 2016	No	Yes	No	Not applicable	No	No	Yes	Yes	No	Yes	Individual tick	Moderate risk of bias
Panayotova, 2016	No	Yes	No	Not applicable	No	No	Yes	Yes	No	Yes	Individual tick	Moderate risk of bias
Papa, 2008	No	Yes	No	Yes	No	No	Yes	Yes	Yes	Yes	Humans	Moderate risk of bias
Papa, 2008	No	Yes	No	Yes	No	No	Yes	Yes	Yes	Yes	Humans	Moderate risk of bias
Papa, 2009	No	Yes	No	Not applicable	No	No	Yes	Yes	Yes	Yes	Tick pools	Moderate risk of bias
Papa, 2009	No	Yes	No	Not applicable	No	No	Yes	Yes	Yes	Yes	Other animals	Moderate risk of bias
Papa, 2009	No	Yes	No	Not applicable	No	No	Yes	Yes	Yes	Yes	Individual tick	Moderate risk of bias
Papa, 2009	No	Yes	No	Not applicable	No	No	Yes	Yes	Yes	Yes	Other animals	Moderate risk of bias
Papa, 2009	No	Yes	No	Not applicable	No	No	Yes	Yes	Yes	Yes	Other animals	Moderate risk of bias
Papa, 2009	No	Yes	No	Not applicable	No	No	Yes	Yes	Yes	Yes	Individual tick	Moderate risk of bias
Papa, 2009	No	Yes	No	Not applicable	No	No	Yes	Yes	Yes	Yes	Individual tick	Moderate risk of bias
Papa, 2013	No	Yes	Yes	Yes	No	No	Yes	Yes	Yes	Yes	Humans	Low risk of bias
Papa, 2016	No	Yes	No	Yes	No	No	Yes	Yes	Yes	Yes	Humans	Moderate risk of bias
Papa, 2017	No	Yes	No	Not applicable	No	No	Yes	Yes	Yes	Yes	Individual tick	Moderate risk of bias
Papa, 2017	No	Yes	No	Not applicable	No	No	Yes	Yes	Yes	Yes	Individual tick	Moderate risk of bias
Papa, 2017	No	Yes	No	Not applicable	No	No	Yes	Yes	Yes	Yes	Tick pools	Moderate risk of bias
Papa, 2017	No	Yes	No	Not applicable	No	No	Yes	Yes	Yes	Yes	Individual tick	Moderate risk of bias
Papa, 2017	No	Yes	No	Not applicable	No	No	Yes	Yes	Yes	Yes	Individual tick	Moderate risk of bias
Papa, 2017	No	Yes	No	Not applicable	No	No	Yes	Yes	Yes	Yes	Individual tick	Moderate risk of bias
Papa, 2018	No	Yes	No	Not applicable	No	No	Yes	Yes	No	Yes	Other animals	Moderate risk of bias
Papa, 2018	No	Yes	No	Not applicable	No	No	Yes	Yes	No	Yes	Other animals	Moderate risk of bias
Pascucci, 2019	No	Yes	No	Not applicable	No	No	Yes	Yes	No	Yes	Individual tick	Moderate risk of bias
Pascucci, 2019	No	Yes	No	Not applicable	No	No	Yes	Yes	No	Yes	Individual tick	Moderate risk of bias
Pascucci, 2019	No	Yes	No	Not applicable	No	No	Yes	Yes	No	Yes	Individual tick	Moderate risk of bias
Qing, 2003	No	Yes	No	Not applicable	No	No	Yes	Yes	Yes	Yes	Other animals	Moderate risk of bias
Rahden, 2019	No	Yes	No	Yes	No	No	Yes	Yes	No	Yes	Humans	Moderate risk of bias
Raileanu, 2015	No	Yes	No	Not applicable	No	No	Yes	Yes	Unclear	Yes	Other animals	Moderate risk of bias
Raileanu, 2015	No	Yes	No	Not applicable	No	No	Yes	Yes	Unclear	Yes	Other animals	Moderate risk of bias
Rezazadeh, 2012	No	Yes	No	Not applicable	No	No	Yes	Yes	Unclear	Yes	Other animals	Moderate risk of bias
Rodhain, 1989	No	Yes	No	Yes	Yes	No	Yes	Yes	Yes	Yes	Humans	Low risk of bias
Rodriguez, 1997	No	Yes	No	Yes	Yes	No	Yes	Yes	Yes	Yes	Humans	Low risk of bias
Rodriguez, 1997	No	Yes	No	Yes	Yes	No	Yes	Yes	Yes	Yes	Humans	Low risk of bias
Rodriguez, 1997	No	Yes	No	Yes	Yes	No	Yes	Yes	Yes	Yes	Humans	Low risk of bias
Rodriguez, 1997	No	Yes	No	Not applicable	No	No	Yes	Yes	Yes	Yes	Individual tick	Moderate risk of bias
Rodriguez, 1997	No	Yes	No	Not applicable	No	No	Yes	Yes	Yes	Yes	Individual tick	Moderate risk of bias
Sadeuh-Mba, 2018	No	Yes	Yes	Yes	Yes	No	Yes	Yes	Yes	Yes	Humans	Low risk of bias
Safronetz, 2016	No	Yes	No	Yes	Yes	No	Yes	Yes	Yes	Yes	Humans	Low risk of bias
Safronetz, 2016	No	Yes	No	Yes	Yes	No	Yes	Yes	Yes	Yes	Humans	Low risk of bias
Saghafipour, 2019	No	Yes	Yes	Not applicable	No	No	Yes	Yes	Yes	Yes	Other animals	Moderate risk of bias
Saghafipour, 2019	No	Yes	Yes	Not applicable	No	No	Yes	Yes	Yes	Yes	Other animals	Moderate risk of bias
Saghafipour, 2019	No	Yes	Yes	Not applicable	No	No	Yes	Yes	Yes	Yes	Individual tick	Moderate risk of bias
Saghafipour, 2019	No	Yes	Yes	Not applicable	No	No	Yes	Yes	Yes	Yes	Individual tick	Moderate risk of bias
Saghafipour, 2019	No	Yes	Yes	Not applicable	No	No	Yes	Yes	Yes	Yes	Other animals	Moderate risk of bias
Sahak, 2019	Yes	Yes	No	Yes	Yes	No	Yes	Yes	No	Yes	Humans	Low risk of bias
Sahak, 2019	Yes	Yes	No	Yes	Yes	No	Yes	Yes	No	Yes	Humans	Low risk of bias

Saidi, 1975	No	Yes	No	Yes	No	No	Yes	Yes	Unclear	Yes	Humans	Moderate risk of bias
Saidi, 1975	No	Yes	No	Not applicable	No	No	Yes	Yes	Unclear	Yes	Other animals	Moderate risk of bias
Saluzzo, 1985	No	Yes	No	Yes	No	No	Yes	Yes	No	Yes	Humans	Moderate risk of bias
Saluzzo, 1985	No	Yes	No	Not applicable	No	No	Yes	Yes	No	Yes	Tick pools	Moderate risk of bias
Saluzzo, 1985	No	Yes	No	Not applicable	No	No	Yes	Yes	No	Yes	Other animals	Moderate risk of bias
Saluzzo, 1985	No	Yes	No	Not applicable	No	No	Yes	Yes	No	Yes	Other animals	Moderate risk of bias
Saluzzo, 1985	No	Yes	No	Not applicable	No	No	Yes	Yes	No	Yes	Individual tick	Moderate risk of bias
Saluzzo, 1985	No	Yes	No	Not applicable	No	No	Yes	Yes	No	Yes	Individual tick	Moderate risk of bias
Saluzzo, 1985	No	Yes	No	Not applicable	No	No	Yes	Yes	No	Yes	Other animals	Moderate risk of bias
Sang, 2011	No	Yes	No	Not applicable	No	No	Yes	Yes	No	Yes	Tick pools	Moderate risk of bias
Sang, 2011	No	Yes	No	Not applicable	No	No	Yes	Yes	No	Yes	Individual tick	Moderate risk of bias
Sang, 2011	No	Yes	No	Not applicable	No	No	Yes	Yes	No	Yes	Individual tick	Moderate risk of bias
Sang, 2011	No	Yes	No	Not applicable	No	No	Yes	Yes	No	Yes	Individual tick	Moderate risk of bias
Sang, 2011	No	Yes	No	Not applicable	No	No	Yes	Yes	No	Yes	Individual tick	Moderate risk of bias
Sang, 2011	No	Yes	No	Not applicable	No	No	Yes	Yes	No	Yes	Individual tick	Moderate risk of bias
Sargianou, 2013	No	Yes	Yes	Yes	No	No	Yes	Yes	Yes	Yes	Humans	Low risk of bias
Sas, 2017	No	Yes	No	Not applicable	No	No	Yes	Yes	No	Yes	Other animals	Moderate risk of bias
Sas, 2017	No	Yes	No	Not applicable	No	No	Yes	Yes	No	Yes	Other animals	Moderate risk of bias
Sas, 2017	No	Yes	No	Not applicable	No	No	Yes	Yes	No	Yes	Other animals	Moderate risk of bias
Sas, 2017	No	Yes	No	Not applicable	No	No	Yes	Yes	Yes	Yes	Other animals	Moderate risk of bias
Schoepp, 2014	No	Yes	No	Yes	Yes	No	Yes	Yes	Yes	Yes	Humans	Low risk of bias
Schuster, 2017	No	Yes	No	Not applicable	No	No	Yes	Yes	No	Yes	Other animals	Moderate risk of bias
Schuster, 2017	No	Yes	No	Not applicable	No	No	Yes	Yes	No	Yes	Other animals	Moderate risk of bias
Schwarz, 1995	No	Yes	No	Yes	No	No	Unclear	Yes	Yes	Yes	Humans	Moderate risk of bias
Schwarz, 1996	No	Yes	No	Yes	No	No	Yes	Yes	Unclear	Yes	Humans	Moderate risk of bias
Schwarz, 1996	No	Yes	No	Yes	No	No	Yes	Yes	Unclear	Yes	Humans	Moderate risk of bias
Schwarz, 1996	No	Yes	No	Yes	No	No	Yes	Yes	Unclear	Yes	Humans	Moderate risk of bias
Schwarz, 1996	No	Yes	No	Yes	No	No	Yes	Yes	Unclear	Yes	Humans	Moderate risk of bias
Schwarz, 1996	No	Yes	No	Yes	No	No	Yes	Yes	Unclear	Yes	Humans	Moderate risk of bias
Sedaghat, 2017	No	Yes	No	Not applicable	No	No	Yes	Yes	No	Yes	Individual tick	Moderate risk of bias
Sedaghat, 2017	No	Yes	No	Not applicable	No	No	Yes	Yes	No	Yes	Individual tick	Moderate risk of bias
Sedaghat, 2017	No	Yes	No	Not applicable	No	No	Yes	Yes	No	Yes	Individual tick	Moderate risk of bias
Sedaghat, 2017	No	Yes	No	Not applicable	No	No	Yes	Yes	No	Yes	Individual tick	Moderate risk of bias
Shahbazi, 2019	No	Yes	No	Yes	No	No	Yes	Yes	Yes	Yes	Humans	Moderate risk of bias
Shahhosseini, 2017	No	Yes	No	Not applicable	No	No	Yes	Yes	No	Yes	Individual tick	Moderate risk of bias
Shahhosseini, 2017	No	Yes	No	Not applicable	No	No	Yes	Yes	No	Yes	Individual tick	Moderate risk of bias
Shahhosseini, 2018	No	Yes	No	Yes	No	No	Yes	Yes	Yes	Yes	Humans	Moderate risk of bias
Sharifi-Mood, 2008	No	Yes	No	Yes	Yes	No	Yes	Yes	Yes	Yes	Humans	Low risk of bias
Sharifi-Mood, 2009	No	Yes	No	Yes	Yes	No	Yes	Yes	Yes	Yes	Humans	Low risk of bias
Sharifinia, 2015	No	Yes	Yes	Not applicable	No	No	Yes	Yes	Yes	Yes	Individual tick	Moderate risk of bias
Sharifinia, 2015	No	Yes	Yes	Not applicable	No	No	Yes	Yes	Yes	Yes	Individual tick	Moderate risk of bias
Sheikh, 2005	No	Yes	No	Yes	Yes	No	Yes	Yes	Yes	Yes	Humans	Low risk of bias
Sheikh, 2005	No	Yes	No	Yes	Yes	No	Yes	Yes	Yes	Yes	Humans	Low risk of bias
Sheikh, 2005	No	Yes	No	Yes	Yes	No	Yes	Yes	Yes	Yes	Humans	Low risk of bias
Sheikh, 2005	No	Yes	No	Yes	Yes	No	Yes	Yes	Yes	Yes	Humans	Low risk of bias
Shepherd, 1985	No	Yes	No	Not applicable	No	No	Yes	Yes	No	Yes	Individual tick	Moderate risk of bias
Shepherd, 1987	No	Yes	No	Not applicable	No	Unclear	Yes	Yes	Yes	Yes	Other animals	Moderate risk of bias
Shepherd, 1987	No	Yes	No	Not applicable	No	Unclear	Yes	Yes	Yes	Yes	Other animals	Moderate risk of bias
Shepherd, 1987	No	Yes	No	Not applicable	No	Unclear	Yes	Yes	Yes	Yes	Other animals	Moderate risk of bias
Shepherd, 1987	No	Yes	No	Not applicable	No	Unclear	Yes	Yes	Yes	Yes	Other animals	Moderate risk of bias
Shepherd, 1987	No	Yes	No	Not applicable	No	Unclear	Yes	Yes	Yes	Yes	Other animals	Moderate risk of bias
Shepherd, 1987	No	Yes	No	Not applicable	No	Unclear	Yes	Yes	Yes	Yes	Other animals	Moderate risk of bias
Shepherd, 1987	No	Yes	No	Not applicable	No	Unclear	Yes	Yes	Yes	Yes	Other animals	Moderate risk of bias
Shepherd, 1987	No	Yes	No	Not applicable	No	Unclear	Yes	Yes	Yes	Yes	Other animals	Moderate risk of bias
Shepherd, 1987	No	Yes	No	Not applicable	No	Unclear	Yes	Yes	Yes	Yes	Other animals	Moderate risk of bias
Shepherd, 1987	No	Yes	No	Not applicable	No	Unclear	Yes	Yes	Yes	Yes	Other animals	Moderate risk of bias
Shepherd, 1987	No	Yes	No	Not applicable	No	Unclear	Yes	Yes	Yes	Yes	Other animals	Moderate risk of bias
Shepherd, 1987	No	Yes	No	Not applicable	No	Unclear	Yes	Yes	Yes	Yes	Other animals	Moderate risk of bias
Shepherd, 1987	No	Yes	No	Not applicable	No	Unclear	Yes	Yes	Yes	Yes	Other animals	Moderate risk of bias
Shepherd, 1987	No	Yes	No	Not applicable	No	Unclear	Yes	Yes	Yes	Yes	Other animals	Moderate risk of bias
Shepherd, 1987	No	Yes	No	Not applicable	No	Unclear	Yes	Yes	Yes	Yes	Other animals	Moderate risk of bias
Shepherd, 1987	No	Yes	No	Not applicable	No	Unclear	Yes	Yes	Yes	Yes	Other animals	Moderate risk of bias
Shepherd, 1987	No	Yes	No	Not applicable	No	Unclear	Yes	Yes	Yes	Yes	Other animals	Moderate risk of bias
Shepherd, 1987	No	Yes	No	Not applicable	No	Unclear	Yes	Yes	Yes	Yes	Other animals	Moderate risk of bias
Shepherd, 1987	No	Yes	No	Not applicable	No	Unclear	Yes	Yes	Yes	Yes	Other animals	Moderate risk of bias
Shepherd, 1987	No	Yes	No	Not applicable	No	Unclear	Yes	Yes	Yes	Yes	Other animals	Moderate risk of bias
Shepherd, 1987	No	Yes	No	Not applicable	No	Unclear	Yes	Yes	Yes	Yes	Other animals	Moderate risk of bias
Shepherd, 1987	No	Yes	No	Not applicable	No	Unclear	Yes	Yes	Yes	Yes	Other animals	Moderate risk of bias
Shepherd, 1987	No	Yes	No	Not applicable	No	Unclear	Yes	Yes	Yes	Yes	Other animals	Moderate risk of bias
Shepherd, 1987	No	Yes	No	Not applicable	No	Unclear	Yes	Yes	Yes	Yes	Other animals	Moderate risk of bias
Shepherd, 1987	No	Yes	No	Not applicable	No	Unclear	Yes	Yes	Yes	Yes	Other animals	Moderate risk of bias
Shepherd, 1987	No	Yes	No	Not applicable	No	Unclear	Yes	Yes	Yes	Yes	Other animals	Moderate risk of bias
Shepherd, 1987	No	Yes	No	Not applicable	No	Unclear	Yes	Yes	Yes	Yes	Other animals	Moderate risk of bias
Shepherd, 1987	No	Yes	No	Not applicable	No	Unclear	Yes	Yes	Yes	Yes	Other animals	Moderate risk of bias
Shepherd, 1987	No	Yes	No	Not applicable	No	Unclear	Yes	Yes	Yes	Yes	Other animals	Moderate risk of bias
Shepherd, 1987	No	Yes	No	Not applicable	No	Unclear	Yes	Yes	Yes	Yes	Other animals	Moderate risk of bias
Shepherd, 1987	No	Yes	No	Not applicable	No	Unclear	Yes	Yes	Yes	Yes	Other animals	Moderate risk of bias
Shepherd, 1987	No	Yes	No	Not applicable	No	Unclear	Yes	Yes	Yes	Yes	Other animals	Moderate risk of bias
Shepherd, 1987	No	Yes	No	Not applicable	No	No	Yes	Yes	Unclear	Yes	Other animals	Moderate risk of bias
Shepherd, 1987	No	Yes	No	Not applicable	No	No	Yes	Yes	Unclear	Yes	Other animals	Moderate risk of bias
Shepherd, 1987	No	Yes	No	Not applicable	No	No	Yes	Yes	Unclear	Yes	Other animals	Moderate risk of bias
Shepherd, 1987	No	Yes	No	Not applicable	No	No	Yes	Yes	Unclear	Yes	Other animals	Moderate risk of bias
Shepherd, 1987	No	Yes	No	Not applicable	No	No	Yes	Yes	Unclear	Yes	Other animals	Moderate risk of bias
Shepherd, 1987	No	Yes	No	Not applicable	No	No	Yes	Yes	Unclear	Yes	Other animals	Moderate risk of bias
Shepherd, 1987	No	Yes	No	Not applicable	No	No	Yes	Yes	Unclear	Yes	Other animals	Moderate risk of bias
Shepherd, 1987	No	Yes	No	Not applicable	No	No	Yes	Yes	Unclear	Yes	Other animals	Moderate risk of bias
Shepherd, 1987	No	Yes	No	Not applicable	No	No	Yes	Yes	Unclear	Yes	Other animals	Moderate risk of bias
Shepherd, 1987	No	Yes	No	Not applicable	No	No	Yes	Yes	Unclear	Yes	Other animals	Moderate risk of bias
Shepherd, 1987	No	Yes	No	Not applicable	No	No	Yes	Yes	Unclear	Yes	Other animals	Moderate risk of bias
Sherifi, 2014	No	Yes	No	Not applicable	No	Unclear	Yes	Yes	No	Yes	Tick pools	Moderate risk of bias
Sherifi, 2014	No	Yes	No	Not applicable	No	Unclear	Yes	Yes	No	Yes	Individual tick	Moderate risk of bias
Sherifi, 2014	No	Yes	No	Not applicable	No	Unclear	Yes	Yes	No	Yes	Individual tick	Moderate risk of bias
Sherifi, 2016	No	Yes	No	Not applicable	No	Unclear	Yes	Yes	Yes	Yes	Other animals	Moderate risk of bias
Sherifi, 2016	No	Yes	No	Not applicable	No	Unclear	Yes	Yes	Yes	Yes	Other animals	Moderate risk of bias
Sherifi, 2016	No	Yes	No	Not applicable	No	Unclear	Yes	Yes	Yes	Yes	Other animals	Moderate risk of bias
Sherifi, 2016	No	Yes	No	Not applicable	No	Unclear	Yes	Yes	Yes	Yes	Other animals	Moderate risk of bias

Sherifi, 2018	No	Yes	Yes	Not applicable	No	Unclear	Yes	Yes	Yes	Yes	Individual tick	Moderate risk of bias
Sherifi, 2018	No	Yes	Yes	Not applicable	No	Unclear	Yes	Yes	Yes	Yes	Individual tick	Moderate risk of bias
Sherifi, 2018	No	Yes	Yes	Not applicable	No	Unclear	Yes	Yes	Yes	Yes	Individual tick	Moderate risk of bias
Sherifi, 2018	No	Yes	Yes	Not applicable	No	Unclear	Yes	Yes	Yes	Yes	Individual tick	Moderate risk of bias
Sherifi, 2018	No	Yes	Yes	Not applicable	No	Unclear	Yes	Yes	Yes	Yes	Individual tick	Moderate risk of bias
Sherifi, 2018	No	Yes	Yes	Not applicable	No	Unclear	Yes	Yes	Yes	Yes	Individual tick	Moderate risk of bias
Shrivastava, 2019	No	Yes	No	Not applicable	No	Unclear	Yes	Yes	Unclear	Yes	Other animals	Moderate risk of bias
Shrivastava, 2019	No	Yes	No	Not applicable	No	Unclear	Yes	Yes	Unclear	Yes	Other animals	Moderate risk of bias
Shrivastava, 2019	No	Yes	No	Not applicable	No	Unclear	Yes	Yes	Unclear	Yes	Other animals	Moderate risk of bias
Shuaib, 2020	No	Yes	No	Not applicable	No	Unclear	Yes	Yes	No	Yes	Individual tick	Moderate risk of bias
Sidira, 2012	No	Yes	Yes	Yes	No	Unclear	Yes	Yes	Yes	Yes	Humans	Low risk of bias
Sidira, 2013	No	Yes	Yes	Yes	No	Unclear	Yes	Yes	Yes	Yes	Humans	Low risk of bias
Sidira, 2013	No	Yes	Yes	Yes	No	Unclear	Yes	Yes	Yes	Yes	Humans	Low risk of bias
Široký, 2014	No	Yes	No	Not applicable	No	Unclear	Yes	Yes	No	Yes	Individual tick	Moderate risk of bias
Sow, 2016	No	Yes	No	Yes	No	Yes	Yes	Yes	Yes	Yes	Humans	Low risk of bias
Sow, 2016	No	Yes	No	Yes	No	Yes	Yes	Yes	Yes	Yes	Humans	Low risk of bias
Suliman, 2017	No	Yes	Yes	Not applicable	No	Unclear	Yes	Yes	No	Yes	Other animals	Moderate risk of bias
Sun, 2009	No	Yes	No	Yes	No	Unclear	Yes	Yes	Yes	Yes	Humans	Moderate risk of bias
Sun, 2009	No	Yes	No	Not applicable	No	Unclear	Yes	Yes	Yes	Yes	Tick pools	Moderate risk of bias
Sun, 2009	No	Yes	No	Not applicable	No	Unclear	Yes	Yes	Yes	Yes	Individual tick	Moderate risk of bias
Sun, 2009	No	Yes	No	Not applicable	No	Unclear	Yes	Yes	Yes	Yes	Other animals	Moderate risk of bias
Sun, 2009	No	Yes	No	Not applicable	No	Unclear	Yes	Yes	Yes	Yes	Individual tick	Moderate risk of bias
Sun, 2009	No	Yes	No	Not applicable	No	Unclear	Yes	Yes	Yes	Yes	Individual tick	Moderate risk of bias
Sunbul, 2015	No	Yes	No	Yes	Yes	Unclear	Yes	Yes	Yes	Yes	Humans	Low risk of bias
Swanepoel, 1983	No	Yes	No	Yes	No	Unclear	Yes	Yes	Yes	Yes	Humans	Moderate risk of bias
Swanepoel, 1983	No	Yes	No	Not applicable	No	Unclear	Yes	Yes	Yes	Yes	Tick pools	Moderate risk of bias
Swanepoel, 1983	No	Yes	No	Not applicable	No	Unclear	Yes	Yes	Yes	Yes	Other animals	Moderate risk of bias
Swanepoel, 1983	No	Yes	No	Not applicable	No	Unclear	Yes	Yes	Yes	Yes	Other animals	Moderate risk of bias
Swanepoel, 1983	No	Yes	No	Not applicable	No	Unclear	Yes	Yes	Yes	Yes	Other animals	Moderate risk of bias
Swanepoel, 1983	No	Yes	No	Not applicable	No	Unclear	Yes	Yes	Yes	Yes	Other animals	Moderate risk of bias
Swanepoel, 1985	No	Yes	No	Yes	No	Unclear	Yes	Yes	Yes	Yes	Humans	Moderate risk of bias
Swanepoel, 1985	No	Yes	No	Yes	No	Unclear	Yes	Yes	Yes	Yes	Humans	Moderate risk of bias
Swanepoel, 1985	No	Yes	No	Yes	No	Unclear	Yes	Yes	Yes	Yes	Humans	Moderate risk of bias
Swanepoel, 1985	No	Yes	No	Yes	No	Unclear	Yes	Yes	Yes	Yes	Humans	Moderate risk of bias
Swanepoel, 1985	No	Yes	No	Yes	No	Unclear	Yes	Yes	Yes	Yes	Humans	Moderate risk of bias
Swanepoel, 1985	No	Yes	No	Not applicable	No	Unclear	Yes	Yes	Yes	Yes	Other animals	Moderate risk of bias
Swanepoel, 1987	No	Yes	Yes	Yes	No	Unclear	Yes	Yes	Yes	Yes	Humans	Low risk of bias
Swanepoel, 1987	No	Yes	Yes	Not applicable	No	Unclear	Yes	Yes	Yes	Yes	Other animals	Moderate risk of bias
Swanepoel, 1987	No	Yes	Yes	Not applicable	No	Unclear	Yes	Yes	Yes	Yes	Other animals	Moderate risk of bias
Tahmasebi, 2010	No	Yes	No	Not applicable	No	No	Yes	Yes	Yes	Yes	Individual tick	Moderate risk of bias
Tahmasebi, 2010	No	Yes	No	Not applicable	No	No	Yes	Yes	Yes	Yes	Individual tick	Moderate risk of bias
Tahmasebi, 2010	No	Yes	No	Not applicable	No	No	Yes	Yes	Yes	Yes	Individual tick	Moderate risk of bias
Tantawi, 1981	No	Yes	No	Not applicable	No	No	Yes	Yes	Yes	Yes	Other animals	Moderate risk of bias
Tantawi, 1981	No	Yes	No	Not applicable	No	No	Yes	Yes	Yes	Yes	Other animals	Moderate risk of bias
Tantawi, 1981	No	Yes	No	Not applicable	No	No	Yes	Yes	Yes	Yes	Other animals	Moderate risk of bias
Tantawi, 1981	No	Yes	No	Not applicable	No	No	Yes	Yes	Yes	Yes	Other animals	Moderate risk of bias
Tantawi, 1981	No	Yes	No	Not applicable	No	No	Yes	Yes	Yes	Yes	Other animals	Moderate risk of bias
Tantawi, 1981	No	Yes	No	Not applicable	No	No	Yes	Yes	Yes	Yes	Other animals	Moderate risk of bias
Tanyel, 2016	No	Yes	No	Yes	Yes	No	Yes	Yes	Yes	Yes	Humans	Low risk of bias
Tarantola, 2007	No	Yes	Yes	Yes	No	No	Yes	Yes	Yes	Yes	Humans	Low risk of bias
Tarantola, 2007	No	Yes	Yes	Yes	No	No	Yes	Yes	Yes	Yes	Humans	Low risk of bias
Tarantola, 2007	No	Yes	Yes	Yes	No	No	Yes	Yes	Yes	Yes	Humans	Low risk of bias
Tasdelen Fisgin, 2009	No	Yes	No	Yes	Yes	No	Yes	Yes	Yes	Yes	Humans	Low risk of bias
Tasdelen Fisgin, 2010	No	Yes	No	Yes	Yes	No	Yes	Yes	No	Yes	Humans	Moderate risk of bias
Tekin, 2012	No	Yes	No	Not applicable	No	No	Yes	Yes	No	Yes	Individual tick	Moderate risk of bias
Tekin, 2012	No	Yes	No	Not applicable	No	No	Yes	Yes	No	Yes	Individual tick	Moderate risk of bias
Tekin, 2012	No	Yes	No	Not applicable	No	No	Yes	Yes	No	Yes	Individual tick	Moderate risk of bias
Tekin, 2012	No	Yes	No	Not applicable	No	No	Yes	Yes	No	Yes	Individual tick	Moderate risk of bias
Tekin, 2012	No	Yes	No	Not applicable	No	No	Yes	Yes	No	Yes	Individual tick	Moderate risk of bias
Tekin, 2012	No	Yes	No	Not applicable	No	No	Yes	Yes	No	Yes	Individual tick	Moderate risk of bias
Tekin, 2012	No	Yes	No	Not applicable	No	No	Yes	Yes	No	Yes	Individual tick	Moderate risk of bias
Tekin, 2012	No	Yes	No	Not applicable	No	No	Yes	Yes	No	Yes	Individual tick	Moderate risk of bias
Tekin, 2012	No	Yes	No	Not applicable	No	No	Yes	Yes	No	Yes	Individual tick	Moderate risk of bias
Tekin, 2012	No	Yes	No	Not applicable	No	No	Yes	Yes	No	Yes	Individual tick	Moderate risk of bias
Tekin, 2012	No	Yes	No	Not applicable	No	No	Yes	Yes	No	Yes	Individual tick	Moderate risk of bias
Telmadarraiy, 2008	No	Yes	No	Yes	No	No	Yes	Yes	Yes	Yes	Humans	Moderate risk of bias
Telmadarraiy, 2008	No	Yes	No	Not applicable	No	No	Yes	Yes	Yes	Yes	Individual tick	Moderate risk of bias
Telmadarraiy, 2008	No	Yes	No	Not applicable	No	No	Yes	Yes	Yes	Yes	Individual tick	Moderate risk of bias
Telmadarraiy, 2008	No	Yes	No	Not applicable	No	No	Yes	Yes	Yes	Yes	Other animals	Moderate risk of bias
Telmadarraiy, 2010	No	Yes	Yes	Not applicable	No	No	Yes	Yes	Yes	Yes	Other animals	Moderate risk of bias
Telmadarraiy, 2010	No	Yes	Yes	Not applicable	No	No	Yes	Yes	Yes	Yes	Individual tick	Moderate risk of bias
Telmadarraiy, 2010	No	Yes	Yes	Not applicable	No	No	Yes	Yes	Yes	Yes	Individual tick	Moderate risk of bias
Telmadarraiy, 2010	No	Yes	Yes	Not applicable	No	No	Yes	Yes	Yes	Yes	Individual tick	Moderate risk of bias
Telmadarraiy, 2010	No	Yes	Yes	Not applicable	No	No	Yes	Yes	Yes	Yes	Individual tick	Moderate risk of bias
Telmadarraiy, 2010	No	Yes	Yes	Not applicable	No	No	Yes	Yes	Yes	Yes	Individual tick	Moderate risk of bias
Telmadarraiy, 2010	No	Yes	Yes	Not applicable	No	No	Yes	Yes	Yes	Yes	Other animals	Moderate risk of bias
Temocin, 2018	No	Yes	No	Yes	No	No	Yes	Yes	Yes	Yes	Humans	Moderate risk of bias
Thomas, 2012	No	Yes	No	Yes	Yes	No	Yes	Yes	Yes	Yes	Humans	Low risk of bias
Tigoi, 2015	No	Yes	No	Yes	Yes	No	Yes	Yes	No	Yes	Humans	Moderate risk of bias
Tigoi, 2015	No	Yes	No	Yes	Yes	No	Yes	Yes	No	Yes	Humans	Moderate risk of bias
Todd, 2016	No	Yes	Yes	Yes	No	No	Yes	Yes	Unclear	Yes	Humans	Moderate risk of bias
Tomori, 1988	No	Yes	No	Yes	No	No	Yes	Yes	Unclear	Yes	Humans	Moderate risk of bias
Tonbak, 2006	No	Yes	No	Not applicable	No	No	Yes	Yes	Yes	Yes	Tick pools	Moderate risk of bias
Tonbak, 2006	No	Yes	No	Not applicable	No	No	Yes	Yes	Yes	Yes	Individual tick	Moderate risk of bias
Tonbak, 2006	No	Yes	No	Not applicable	No	No	Yes	Yes	Yes	Yes	Individual tick	Moderate risk of bias
Tumturk, 2019	No	Yes	No	Yes	No	No	Yes	Yes	No	Yes	Humans	Moderate risk of bias
Tumturk, 2019	No	Yes	No	Yes	No	No	Yes	Yes	No	Yes	Humans	Moderate risk of bias
Tuncer, 2014	No	Yes	No	Not applicable	No	No	Yes	Yes	Yes	Yes	Other animals	Moderate risk of bias
Tuncer, 2014	No	Yes	No	Not applicable	No	No	Yes	Yes	Yes	Yes	Other animals	Moderate risk of bias
Tuncer, 2014	No	Yes	No	Not applicable	No	No	Yes	Yes	Yes	Yes	Other animals	Moderate risk of bias
Tuncer, 2014	No	Yes	No	Not applicable	No	No	Yes	Yes	Yes	Yes	Other animals	Moderate risk of bias
Tuncer, 2014	No	Yes	No	Not applicable	No	No	Yes	Yes	Yes	Yes	Other animals	Moderate risk of bias
Tuncer, 2014	No	Yes	No	Not applicable	No	No	Yes	Yes	Yes	Yes	Other animals	Moderate risk of bias
Tuygun, 2012	No	Yes	No	Yes	Yes	No	Yes	Yes	Yes	Yes	Humans	Low risk of bias

Tuygun, 2012	No	Yes	No	Yes	Yes	No	Yes	Yes	Yes	Yes	Humans	Low risk of bias
Tuygun, 2012	No	Yes	No	Yes	Yes	No	Yes	Yes	Yes	Yes	Humans	Low risk of bias
Umoh, 1983	No	Yes	No	Not applicable	No	No	Yes	Yes	Unclear	Yes	Other animals	Moderate risk of bias
Vawda, 2018	No	Yes	No	Yes	No	No	Yes	Yes	Yes	Yes	Humans	Moderate risk of bias
Voorhees, 2018	No	Yes	No	Yes	No	No	Yes	Yes	Yes	Yes	Humans	Moderate risk of bias
Voorhees, 2018	No	Yes	No	Not applicable	No	No	Yes	Yes	Yes	Yes	Tick pools	Moderate risk of bias
Wangchuk, 2016	No	Yes	No	Not applicable	No	No	Yes	Yes	No	Yes	Other animals	Moderate risk of bias
Wangchuk, 2016	No	Yes	No	Not applicable	No	No	Yes	Yes	No	Yes	Other animals	Moderate risk of bias
Wasfi, 2016	No	Yes	No	Yes	Yes	No	Yes	Yes	No	Yes	Humans	Moderate risk of bias
Wasfi, 2016	No	Yes	No	Yes	No	No	Yes	Yes	No	Yes	Humans	Moderate risk of bias
Wasfi, 2016	No	Yes	No	Yes	Yes	No	Yes	Yes	No	Yes	Humans	Moderate risk of bias
Wasfi, 2016	No	Yes	No	Yes	Yes	No	Yes	Yes	No	Yes	Humans	Moderate risk of bias
Watts, 1994	No	Yes	No	Yes	No	No	Yes	Yes	Unclear	Yes	Humans	Moderate risk of bias
Williams, 2000	No	Yes	No	Yes	No	No	Yes	Yes	Unclear	Yes	Humans	Moderate risk of bias
Williams, 2000	No	Yes	No	Not applicable	No	No	Yes	Yes	Unclear	Yes	Tick pools	Moderate risk of bias
Williams, 2000	No	Yes	No	Not applicable	No	No	Yes	Yes	Unclear	Yes	Other animals	Moderate risk of bias
Williams, 2000	No	Yes	No	Not applicable	No	No	Yes	Yes	Unclear	Yes	Other animals	Moderate risk of bias
Williams, 2000	No	Yes	No	Not applicable	No	No	Yes	Yes	Unclear	Yes	Other animals	Moderate risk of bias
Williams, 2000	No	Yes	No	Not applicable	No	No	Yes	Yes	Unclear	Yes	Individual tick	Moderate risk of bias
Williams, 2000	No	Yes	No	Not applicable	No	No	Yes	Yes	Unclear	Yes	Individual tick	Moderate risk of bias
Williams, 2000	No	Yes	No	Not applicable	No	No	Yes	Yes	Unclear	Yes	Individual tick	Moderate risk of bias
Williams, 2000	No	Yes	No	Not applicable	No	No	Yes	Yes	Unclear	Yes	Other animals	Moderate risk of bias
Wilson, 1990	Yes	Yes	Yes	Yes	No	Yes	Yes	Yes	Yes	Yes	Humans	Low risk of bias
Wilson, 1990	Yes	Yes	Yes	Not applicable	No	Yes	Yes	Yes	Yes	Yes	Other animals	Low risk of bias
Wood, 1978	No	Yes	Yes	Not applicable	No	No	Yes	Yes	Yes	Yes	Tick pools	Moderate risk of bias
Xia, 2011	No	Yes	No	Yes	Yes	Yes	Yes	Yes	No	Yes	Humans	Low risk of bias
Xia, 2011	No	Yes	No	Not applicable	No	Yes	Yes	Yes	No	Yes	Tick pools	Moderate risk of bias
Yadav, 2014	No	Yes	No	Yes	Yes	No	Yes	Yes	No	Yes	Humans	Moderate risk of bias
Yadav, 2014	No	Yes	No	Yes	Yes	No	Yes	Yes	No	Yes	Humans	Moderate risk of bias
Yadav, 2014	No	Yes	No	Yes	Yes	No	Yes	Yes	No	Yes	Humans	Moderate risk of bias
Yadav, 2014	No	Yes	No	Yes	Yes	No	Yes	Yes	No	Yes	Humans	Moderate risk of bias
Yadav, 2014	No	Yes	No	Yes	Yes	No	Yes	Yes	No	Yes	Humans	Moderate risk of bias
Yadav, 2014	No	Yes	No	Not applicable	No	No	Yes	Yes	No	Yes	Tick pools	Moderate risk of bias
Yadav, 2014	No	Yes	No	Not applicable	No	No	Yes	Yes	No	Yes	Other animals	Moderate risk of bias
Yadav, 2014	No	Yes	No	Not applicable	No	No	Yes	Yes	No	Yes	Other animals	Moderate risk of bias
Yadav, 2014	No	Yes	No	Not applicable	No	No	Yes	Yes	No	Yes	Other animals	Moderate risk of bias
Yagci-Caglayik, 2014	No	Yes	Yes	Yes	No	No	Yes	Yes	Unclear	Yes	Humans	Moderate risk of bias
Yagub, 2017	No	Yes	No	Yes	Yes	No	Yes	Yes	Yes	Yes	Humans	Low risk of bias
Yagub, 2017	No	Yes	No	Yes	Yes	No	Yes	Yes	Yes	Yes	Humans	Low risk of bias
Yaser, 2011	No	Yes	Yes	Not applicable	No	No	Yes	Yes	Yes	Yes	Individual tick	Moderate risk of bias
Yaser, 2011	No	Yes	Yes	Not applicable	No	No	Yes	Yes	Yes	Yes	Individual tick	Moderate risk of bias
Yaser, 2011	No	Yes	Yes	Not applicable	No	No	Yes	Yes	Yes	Yes	Individual tick	Moderate risk of bias
Yaser, 2011	No	Yes	Yes	Not applicable	No	No	Yes	Yes	Yes	Yes	Individual tick	Moderate risk of bias
Yaser, 2011	No	Yes	Yes	Not applicable	No	No	Yes	Yes	Yes	Yes	Individual tick	Moderate risk of bias
Yashina, 2003	No	Yes	No	Yes	No	No	Yes	Yes	Unclear	Yes	Humans	Moderate risk of bias
Yashina, 2003	No	Yes	No	Not applicable	No	No	Yes	Yes	Unclear	Yes	Tick pools	Moderate risk of bias
Yashina, 2003	No	Yes	No	Yes	No	No	Yes	Yes	Unclear	Yes	Humans	Moderate risk of bias
Yesilbag, 2013	No	Yes	No	Not applicable	No	No	Yes	Yes	Yes	Yes	Tick pools	Moderate risk of bias
Yilmaz, 2009	No	Yes	No	Yes	Yes	No	Yes	Yes	Yes	Yes	Humans	Low risk of bias
Yilmaz, 2015	No	Yes	No	Yes	Yes	No	Yes	Yes	Yes	Yes	Humans	Low risk of bias
Zeller, 1994	No	Yes	No	Not applicable	No	No	Yes	Yes	Yes	Yes	Other animals	Moderate risk of bias
Zeller, 1994	No	Yes	No	Not applicable	No	No	Yes	Yes	Yes	Yes	Other animals	Moderate risk of bias
Zeller, 1994	No	Yes	No	Not applicable	No	No	Yes	Yes	Yes	Yes	Other animals	Moderate risk of bias
Zeller, 1994	No	Yes	No	Not applicable	No	No	Yes	Yes	Yes	Yes	Other animals	Moderate risk of bias
Zeller, 1994	No	Yes	No	Not applicable	No	No	Yes	Yes	Yes	Yes	Other animals	Moderate risk of bias
Zeller, 1994	No	Yes	No	Not applicable	No	No	Yes	Yes	Yes	Yes	Other animals	Moderate risk of bias
Zhang, 2019	No	Yes	No	Yes	No	Unclear	Yes	Yes	Yes	Yes	Humans	Moderate risk of bias
Ziauddin, 2018	No	Yes	No	Yes	Yes	Unclear	Yes	Yes	Yes	Yes	Humans	Low risk of bias
Ziauddin, 2018	No	Yes	No	Yes	Yes	Unclear	Yes	Yes	Yes	Yes	Humans	Low risk of bias
Zivcec, 2014	No	Yes	No	Not applicable	No	No	Yes	Yes	Yes	Yes	Tick pools	Moderate risk of bias
