# Supplementary material for: Worldwide epidemiology of Crimean-Congo Hemorrhagic Fever Virus in humans, ticks and other animal species, a systematic review and meta-analysis
Source: PLoS Negl Trop Dis. 2021 Apr 22;15(4):e0009299. doi: 10.1371/journal.pntd.0009299 (PMC8096040; doi:10.1371/journal.pntd.0009299)
Supplement: S6 Table — (PDF) [file pntd.0009299.s010.pdf]

S6 Table. Characteristics of included studies

**Characteristics**	**Overall (802)**	**Humans CFR (53)**	**Humans prevlence (270)**	**Individual tick (209)**	**Tick pools (36)**	**Other animals (234)**
**Year of publication; range**	1974-2020	1987-2019	1974-2020	1985-2020	1978-2019	1975-2020
**Period of inclusion of participants; range**	1964-2018	1973-2018	1977-2018	1984-2017	1974-2016	1964-2017
**Study Design**						
Case-series	1 (0.1)	1 (1.9)				
Case control	3 (0.4)	2 (3.8)	1 (0.4)			
Cohort (Baseline data)	2 (0.3)		2 (0.7)			
Community outbreak	16 (2.0)	1 (1.9)	15 (5.6)			
Cross sectional	768 (95.8)	47 (88.7)	242 (89.6)	209 (100.0)	36 (100.0)	234 (100.0)
Hospital outbreak	12 (1.5)	2 (3.8)	10 (3.7)			
**Sampling**						
Non probabilistic	645 (80.4)	52 (98.1)	226 (83.7)	136 (65.1)	33 (91.7)	198 (84.6)
Probabilistic	157 (19.6)	1 (1.9)	44 (16.3)	73 (34.9)	3 (8.3)	36 (15.4)
**Sampling method**						
Capture	255 (31.8)			209 (100.0)	36 (100.0)	10 (4.3)
Cluster sampling	4 (0.5)		4 (1.5)			
Consecutive sampling	444 (55.4)	50 (94.3)	214 (79.3)			180 (76.9)
Convenience sampling	22 (2.7)	2 (3.8)	12 (4.4)			8 (3.4)
Multistage sampling	4 (0.5)					4 (1.7)
Simple random sampling	66 (8.2)	1 (1.9)	35 (13.0)			30 (12.8)
Stratified sampling	5 (0.6)		5 (1.9)			
Systematic sampling	2 (0.3)					2 (0.9)
**Number of sites**						
Monocenter	162 (20.2)	36 (67.9)	74 (27.4)	29 (13.9)	3 (8.3)	20 (8.6)
Multicenter	609 (75.9)	12 (22.6)	182 (67.4)	180 (86.1)	32 (88.9)	203 (86.8)
Nationally representative	15 (1.9)	4 (7.6)	8 (3.0)			3 (1.3)
Unclear/ Not reported	16 (2.0)	1 (1.9)	6 (2.2)		1 (2.8)	8 (3.4)
**Timing of data collection**						
Prospectively	693 (86.4)	26 (49.1)	220 (81.5)	201 (96.2)	33 (91.7)	213 (91.0)
Retroprospectively	3 (0.4)	1 (1.9)	1 (0.4)			1 (0.4)
Retrospectively	81 (10.1)	26 (49.1)	41 (15.2)	2 (1.0)	1 (2.8)	11 (4.7)
Unclear/ Not reported	25 (3.1)		8 (3.0)	6 (2.9)	2 (5.6)	9 (3.9)
**Country**						

Characteristics	Overall (802)	Humans CFR (53)	Humans prevlence (270)	Individual tick (209)	Tick pools (36)	Other animals (234)
Afghanistan	15 (1.9)	3 (5.7)	10 (3.7)			2 (0.9)
Albania	12 (1.5)		2 (0.7)	6 (2.9)	1 (2.8)	3 (1.3)
Algeria	1 (0.1)			1 (0.5)		
Armenia	6 (0.8)			6 (2.9)		
Bhutan	2 (0.3)					2 (0.9)
Bulgaria	28 (3.5)		7 (2.6)	13 (6.2)		8 (3.4)
Cameroon	2 (0.3)		2 (0.7)			
Central Africa Republic	4 (0.5)		3 (1.1)			1 (0.4)
Chad	1 (0.1)		1 (0.4)			
China	10 (1.3)		2 (0.7)	3 (1.4)	3 (8.3)	2 (0.9)
Democratic Republic of the Congo	6 (0.8)		3 (1.1)			3 (1.3)
Djibouti	9 (1.1)		1 (0.4)	8 (3.8)		
Egypt	13 (1.6)			1 (0.5)	1 (2.8)	11 (4.7)
Equatorial Guinea	1 (0.1)		1 (0.4)			
Ethiopia	1 (0.1)				1 (2.8)	
France	1 (0.1)			1 (0.5)		
Gabon	2 (0.3)		1 (0.4)			1 (0.4)
Georgia	3 (0.4)		3 (1.1)			
Germany	1 (0.1)					1 (0.4)
Ghana	5 (0.6)		3 (1.1)		1 (2.8)	1 (0.4)
Greece	23 (2.9)		11 (4.1)	7 (3.4)	1 (2.8)	4 (1.7)
Hungary	7 (0.9)		1 (0.4)			6 (2.6)
India	35 (4.4)	3 (5.7)	10 (3.7)	3 (1.4)	2 (5.6)	17 (7.3)
Iran	126 (15.7)	7 (13.2)	33 (12.2)	62 (29.7)		24 (10.3)
Iraq	10 (1.3)	1 (1.9)	2 (0.7)			7 (3.0)
Israel	4 (0.5)			4 (1.9)		1 (0.4)
Italy	6 (0.8)			5 (2.4)		
Kazakhstan	5 (0.6)		4 (1.5)		1 (2.8)	
Kenya	12 (1.5)		6 (2.2)	5 (2.4)	1 (2.8)	
Kuwait	1 (0.1)		1 (0.4)			
Madagascar	4 (0.5)		3 (1.1)		1 (2.8)	
Malaysia	2 (0.3)		2 (0.7)			
Mali	5 (0.6)		3 (1.1)		1 (2.8)	1 (0.4)
Mauritania	25 (3.1)		8 (3.0)	5 (2.4)	2 (5.6)	10 (4.3)
Middle East	2 (0.3)		2 (0.7)			

Characteristics	Overall (802)	Humans CFR (53)	Humans prevlence (270)	Individual tick (209)	Tick pools (36)	Other animals (234)
Mongolia	2 (0.3)		1 (0.4)		1 (2.8)	
Mozambique	1 (0.1)		1 (0.4)			
Niger	5 (0.6)					5 (2.1)
Nigeria	7 (0.9)		6 (2.2)			1 (0.4)
Oman	10 (1.3)		2 (0.7)	3 (1.4)	1 (2.8)	4 (1.7)
Pakistan	33 (4.1)	2 (3.8)	19 (7.0)	5 (2.4)		7 (3.0)
Panama	1 (0.1)					1 (0.4)
Poland	3 (0.4)			2 (1.0)		1 (0.4)
Portugal	2 (0.3)		1 (0.4)			1 (0.4)
Replublic of Kosovo	19 (2.4)	1 (1.9)	2 (0.7)	8 (3.8)	1 (2.8)	7 (3.0)
Republic of the Congo	1 (0.1)					1 (0.4)
Romania	3 (0.4)					3 (1.3)
Russia	3 (0.4)		2 (0.7)		1 (2.8)	
Saudi Arabia	10 (1.3)		5 (1.9)		1 (2.8)	4 (1.7)
Senegal	12 (1.5)		5 (1.9)			7 (3.0)
Sierra Leone	3 (0.4)		3 (1.1)			
Somalia	1 (0.1)		1 (0.4)			
South Africa	48 (6.0)	1 (1.9)	9 (3.3)	1 (0.5)	1 (2.8)	36 (15.4)
South Africa, Zimbabwe	30 (3.7)					30 (12.8)
South Sudan	1 (0.1)		1 (0.4)			
Spain	15 (1.9)		1 (0.4)	13 (6.2)	1 (2.8)	
Sudan	11 (1.4)		7 (2.6)	1 (0.5)		3 (1.3)
Tunisia	5 (0.6)		4 (1.5)	1 (0.5)		
Turkey	158 (19.7)	35 (66.0)	58 (21.5)	39 (18.7)	13 (36.1)	13 (5.6)
Turkey; Syria	1 (0.1)			1 (0.5)		
Uganda	4 (0.5)		2 (0.7)	2 (1.0)		
United Arab Emirates	21 (2.6)		14 (5.2)	3 (1.4)		4 (1.7)
Zimbabwe	2 (0.3)		1 (0.4)			1 (0.4)
**Country income level**						
High-income economies	108 (13.5)		36 (13.3)	38 (18.2)	4 (11.1)	30 (12.8)
Low-income economies	62 (7.7)	3 (5.7)	38 (14.1)	3 (1.4)	3 (8.3)	15 (6.4)
Lower-middle income economies	166 (20.7)	5 (9.4)	66 (24.4)	29 (13.9)	8 (22.2)	58 (24.8)
Upper-middle-income economies	433 (54.0)	45 (84.9)	128 (47.4)	138 (66.0)	21 (58.3)	101 (43.2)
Unclear	33 (4.1)		2 (0.7)	1 (0.5)		30 (12.8)
**UNSD Region**						

**Characteristics**	**Overall (802)**	**Humans CFR (53)**	**Humans prevlence (270)**	**Individual tick (209)**	**Tick pools (36)**	**Other animals (234)**
Central Africa	17 (2.1)		11 (4.1)			6 (2.6)
Central America	1 (0.1)					1 (0.4)
Central Asia	5 (0.6)		4 (1.5)		1 (2.8)	
Eastern Africa	34 (4.2)		16 (5.9)	15 (7.2)	3 (8.3)	
Eastern Asia	12 (1.5)		3 (1.1)	3 (1.4)	4 (11.1)	2 (0.9)
Eastern Europe	44 (5.5)		10 (3.7)	15 (7.2)	1 (2.8)	18 (7.7)
Northern Africa	30 (3.7)		11 (4.1)	4 (1.9)	1 (2.8)	14 (6.0)
Southeastern Asia	2 (0.3)		2 (0.7)			
Southern Africa	79 (9.9)	1 (1.9)	9 (3.3)	1 (0.5)	1 (2.8)	67 (28.6)
Southern Asia	210 (26.2)	15 (28.3)	72 (26.7)	70 (33.5)	2 (5.6)	51 (21.8)
Southern Europe	58 (7.2)		15 (5.6)	31 (14.8)	3 (8.3)	9 (3.9)
UNSD unclassified	19 (2.4)	1 (1.9)	2 (0.7)	8 (3.8)	1 (2.8)	7 (3.0)
West Africa	62 (7.7)		28 (10.4)	5 (2.4)	4 (11.1)	25 (10.7)
Western Asia	225 (28.1)	36 (67.9)	85 (31.5)	56 (26.8)	15 (41.7)	33 (14.1)
Western Europe	2 (0.3)			1 (0.5)		1 (0.4)
Unclear	2 (0.3)		2 (0.7)			
**Age (years); Median [IQR]**	40.5 [32.4-47.1]	33.8 [44.9-48.7]	37.7 [30.8-46.2]	NA	NA	NA
**Age range (years)**						
Adults: 19+ years	43 (5.4)	5 (9.4)	38 (14.1)			
All ages	98 (12.2)	15 (28.3)	83 (30.7)			
Child: Birth-18 years	14 (1.8)	3 (5.7)	11 (4.1)			
Unclear/Not reported	647 (80.7)	30 (56.6)	138 (51.1)	209 (100.0)	36 (100.0)	234 (100.0)
**%Male. Range**	[14.5-100]	[34.8-84.4]	[14.5-100]	NA	NA	NA
**Recrutment setting**						
Rural	156 (19.5)	1 (1.9)	31 (11.5)	51 (24.4)	8 (22.2)	65 (27.8)
Unclear/Not reported	434 (54.1)	32 (60.4)	135 (50.0)	111 (53.1)	19 (52.8)	137 (58.6)
Urban	77 (9.6)	10 (18.9)	50 (18.5)	4 (1.9)	3 (8.3)	10 (4.3)
Urban/rural	135 (16.8)	10 (18.9)	54 (20.0)	43 (20.6)	6 (16.7)	22 (9.4)
**Setting**						
Community-based	588 (73.3)	1 (1.9)	108 (40.0)	209 (100.0)	36 (100.0)	234 (100.0)
Hospital-based	172 (21.5)	45 (84.9)	127 (47.0)			
Unclear/Not reported	42 (5.2)	7 (13.2)	35 (13.0)			
**Hospitalization**						
Ambulatory	36 (4.5)	2 (3.8)	34 (12.6)			

Characteristics	Overall (802)	Humans CFR (53)	Humans prevlence (270)	Individual tick (209)	Tick pools (36)	Other animals (234)
Hospitalized	89 (11.1)	35 (66.0)	54 (20.0)			
Not applicable (if not in the hospital)	583 (72.7)	2 (3.8)	102 (37.8)	209 (100.0)	36 (100.0)	234 (100.0)
Unclear/Not reported	94 (11.7)	14 (26.4)	80 (29.6)			
**Tick genotyping**						
No	119 (14.8)			105 (50.2)	14 (38.9)	
Not applicable	557 (69.5)	53 (100.0)	270 (100.0)			234 (100.0)
Unclear/ Not reported	120 (15.0)			98 (46.9)	22 (61.1)	
Yes	6 (0.8)			6 (2.9)		
**Species**						
Humans	270 (33.7)		270 (100.0)			
Humans_CFR	53 (6.6)	53 (100.0)				
Individual tick	209 (26.1)			209 (100.0)		
Other animals	234 (29.2)					234 (100.0)
Tick pools	36 (4.5)				36 (100.0)	
**Gender (Tick)**						
Amblyomma	6 (0.8)			6 (2.9)		
Argas	2 (0.3)			2 (1.0)		
Dermacentor	13 (1.6)			13 (6.2)		
Haemaphysalis	14 (1.8)			14 (6.7)		
Hyalomma	104 (13.0)			104 (49.8)		
Ixodes	9 (1.1)			9 (4.3)		
Mixed tick species	5 (0.6)			5 (2.4)		
Ornithodoros	2 (0.3)			2 (1.0)		
Rhipicephalus	51 (6.4)			51 (24.4)		
Unspecified tick	3 (0.4)			3 (1.4)		
Not applicable	593 (73.9)	53 (100.0)	270 (100.0)		36 (100.0)	234 (100.0)
**Order (Other animals)**						
Animal unspecified	4 (0.5)					4 (1.7)
Anseriformes	1 (0.1)					1 (0.4)
Apodiformes	1 (0.1)					1 (0.4)
Artiodactyla	157 (19.6)					157 (67.1)
Bird unsepecified	1 (0.1)					1 (0.4)
Bucerotiformes	1 (0.1)					1 (0.4)
Carnivora	8 (1.0)					8 (3.4)
Chiroptera	3 (0.4)					3 (1.3)

Characteristics	Overall (802)	Humans CFR (53)	Humans prevlence (270)	Individual tick (209)	Tick pools (36)	Other animals (234)
Columbiformes	2 (0.3)					2 (0.9)
Eulipotyphla	1 (0.1)					1 (0.4)
Gruiformes	1 (0.1)					1 (0.4)
Hyracoidea	1 (0.1)					1 (0.4)
Lagomorpha	8 (1.0)					8 (3.4)
Macroscelidea	2 (0.3)					2 (0.9)
Mixed animals	4 (0.5)					4 (1.7)
Passeriformes	6 (0.8)					6 (2.6)
Pelecaniformes	1 (0.1)					1 (0.4)
Perissodactyla	4 (0.5)					4 (1.7)
Primates	1 (0.1)					1 (0.4)
Proboscidea	2 (0.3)					2 (0.9)
Pteroclifores	1 (0.1)					1 (0.4)
Rodentia	21 (2.6)					21 (9.0)
Struthioniformes	2 (0.3)					2 (0.9)
Unclassified small mammals	1 (0.1)					1 (0.4)
Not applicable	568 (70.8)	53 (100.0)	270 (100.0)	209 (100.0)	36 (100.0)	
**Population characteristics**						
**Humans - Case fatality rate**						
Positive among CCHFV suspected cases	47 (88.7)	47 (88.7)				
Positive among febrile patients	2 (3.8)	2 (3.8)				
Positive among Healtcare workers	1 (1.9)	1 (1.9)				
Positive among patient with hemorrhagic symptoms	3 (5.7)	3 (5.7)				
**Humans - Prevalence**						
Apparently healthy individuals	66 (24.4)		66 (24.4)			
Blood donors	2 (0.7)		2 (0.7)			
CCHFV positive case contact	6 (2.2)		6 (2.2)			
CCHFV positive patient	3 (1.1)		3 (1.1)			
CCHFV suspected cases	73 (27.0)		73 (27.0)			
Febrile patients	44 (16.3)		44 (16.3)			
Healtcare workers	14 (5.2)		14 (5.2)			
High risk individuals	35 (13.0)		35 (13.0)			
Mixed human categories	12 (4.4)		12 (4.4)			
Patient with any illness	1 (0.4)		1 (0.4)			
Patient with hemorrhagic symptoms	14 (5.2)		14 (5.2)			

Characteristics	Overall (802)	Humans CFR (53)	Humans prevlence (270)	Individual tick (209)	Tick pools (36)	Other animals (234)
**Individual tick**						
Amblyomma cohaerens	1 (0.5)			1 (0.5)		
Amblyomma gemma	1 (0.5)			1 (0.5)		
Amblyomma lepidum	2 (1.0)			2 (1.0)		
Amblyomma variegatum	1 (0.5)			1 (0.5)		
Ambylomma variegatum	1 (0.5)			1 (0.5)		
Argas persicus	2 (1.0)			2 (1.0)		
Boophilus annulatus	1 (0.5)			1 (0.5)		
Boophilus calcaratus	1 (0.5)			1 (0.5)		
Boophilus decoloratus	1 (0.5)			1 (0.5)		
Boophilus species	2 (1.0)			2 (1.0)		
Boophylus annulatus	1 (0.5)			1 (0.5)		
Dermacentor marginatus	9 (4.3)			9 (4.3)		
Dermacentor niveus	1 (0.5)			1 (0.5)		
Dermacentor reticulatus	1 (0.5)			1 (0.5)		
Dermacentor species	1 (0.5)			1 (0.5)		
Dermacentor marginatus	1 (0.5)			1 (0.5)		
Haemaphysalis concinna	1 (0.5)			1 (0.5)		
Haemaphysalis erinacei	1 (0.5)			1 (0.5)		
Haemaphysalis inermis	1 (0.5)			1 (0.5)		
Haemaphysalis parva	2 (1.0)			2 (1.0)		
Haemaphysalis punctata	2 (1.0)			2 (1.0)		
Haemaphysalis species	1 (0.5)			1 (0.5)		
Haemaphysalis sulcata	6 (2.9)			6 (2.9)		
Hyalomma aegyptium	8 (3.8)			8 (3.8)		
Hyalomma anatolicum	15 (7.2)			15 (7.2)		
Hyalomma anatolicum anatolicum	3 (1.4)			3 (1.4)		
Hyalomma asiaticum	5 (2.4)			5 (2.4)		
Hyalomma detritum	4 (1.9)			4 (1.9)		
Hyalomma dromedari	1 (0.5)			1 (0.5)		
Hyalomma dromedarii	11 (5.3)			11 (5.3)		
Hyalomma excavatum	2 (1.0)			2 (1.0)		
Hyalomma impeltatum	2 (1.0)			2 (1.0)		
Hyalomma lusitanicum	3 (1.4)			3 (1.4)		
Hyalomma marginatum	22 (10.5)			22 (10.5)		

Characteristics	Overall (802)	Humans CFR (53)	Humans prevlence (270)	Individual tick (209)	Tick pools (36)	Other animals (234)
Hyalomma marginatum marginatum	2 (1.0)			2 (1.0)		
Hyalomma marginatum rufipes	2 (1.0)			2 (1.0)		
Hyalomma parva	1 (0.5)			1 (0.5)		
Hyalomma punctata	1 (0.5)			1 (0.5)		
Hyalomma rufipes	2 (1.0)			2 (1.0)		
Hyalomma scupense	1 (0.5)			1 (0.5)		
Hyalomma species	13 (6.2)			13 (6.2)		
Hyalomma sulcata	2 (1.0)			2 (1.0)		
Hyalomma truncatum	1 (0.5)			1 (0.5)		
Hyalomma turanicum	1 (0.5)			1 (0.5)		
Hyalomma dromedarii	1 (0.5)			1 (0.5)		
Hyalomma marginatum	1 (0.5)			1 (0.5)		
Ixodes daminii	1 (0.5)			1 (0.5)		
Ixodes ricinus	6 (2.9)			6 (2.9)		
Ixodes species	2 (1.0)			2 (1.0)		
Mixed tick species	5 (2.4)			5 (2.4)		
Ormithodoros savygni	1 (0.5)			1 (0.5)		
Ornithodoros lahorensis	1 (0.5)			1 (0.5)		
Rhipicephalus (Boophilus) annulatus	1 (0.5)			1 (0.5)		
Rhipicephalus annulatus	1 (0.5)			1 (0.5)		
Rhipicephalus appendiculatus	2 (1.0)			2 (1.0)		
Rhipicephalus bursa	11 (5.3)			11 (5.3)		
Rhipicephalus pulchellus	1 (0.5)			1 (0.5)		
Rhipicephalus sanguineus	18 (8.6)			18 (8.6)		
Rhipicephalus species	7 (3.4)			7 (3.4)		
Rhipicephalus turanicus	3 (1.4)			3 (1.4)		
Riphicephalus bursa	1 (0.5)			1 (0.5)		
Unspecified tick	3 (1.4)			3 (1.4)		
**Tick pools**						
Positive tick pool species	36 (100.0)				36 (100.0)	
**Other animals**						
Aepyceros melampus	1 (0.4)					1 (0.4)
Aethomys chrysophilus	1 (0.4)					1 (0.4)
Aethomys namaquensis	1 (0.4)					1 (0.4)
Animal unspecified	4 (1.7)					4 (1.7)

**Characteristics**	**Overall (802)**	**Humans CFR (53)**	**Humans prevlence (270)**	**Individual tick (209)**	**Tick pools (36)**	**Other animals (234)**
Apodemus agrarius	1 (0.4)					1 (0.4)
Apodemus flavicollis	1 (0.4)					1 (0.4)
Apodemus sylvaticus	1 (0.4)					1 (0.4)
Arvicanthis niloticus	1 (0.4)					1 (0.4)
Baboon	1 (0.4)					1 (0.4)
Bat	3 (1.3)					3 (1.3)
Bird unsepecified	1 (0.4)					1 (0.4)
Blue wildebeest	1 (0.4)					1 (0.4)
Bovine	3 (1.3)					3 (1.3)
Bubalornis albirostris	1 (0.4)					1 (0.4)
Buffalo	5 (2.1)					5 (2.1)
Camel	9 (3.9)					9 (3.9)
Canis familiaris	1 (0.4)					1 (0.4)
Cattle	36 (15.4)					36 (15.4)
Cattle egret	1 (0.4)					1 (0.4)
Cheetah	1 (0.4)					1 (0.4)
Cow	6 (2.6)					6 (2.6)
Crocidura species	1 (0.4)					1 (0.4)
Dasymys incomtus	1 (0.4)					1 (0.4)
Desmodillus auricularis	1 (0.4)					1 (0.4)
Elephant	1 (0.4)					1 (0.4)
Elephantulus species	1 (0.4)					1 (0.4)
Equus burchelli	1 (0.4)					1 (0.4)
Felis caracal	1 (0.4)					1 (0.4)
Genetta genetta	1 (0.4)					1 (0.4)
Giraffe	1 (0.4)					1 (0.4)
Goat	35 (15.0)					35 (15.0)
Hare	2 (0.9)					2 (0.9)
Hippopotamus	1 (0.4)					1 (0.4)
Horse	1 (0.4)					1 (0.4)
Impala	1 (0.4)					1 (0.4)
Lamprotornis species	1 (0.4)					1 (0.4)
Laughing dove	1 (0.4)					1 (0.4)
Lepus capensis	1 (0.4)					1 (0.4)
Lepus saxatilis	1 (0.4)					1 (0.4)

**Characteristics**	**Overall (802)**	**Humans CFR (53)**	**Humans prevlence (270)**	**Individual tick (209)**	**Tick pools (36)**	**Other animals (234)**
Lepus species	1 (0.4)					1 (0.4)
Lion	1 (0.4)					1 (0.4)
Little swift	1 (0.4)					1 (0.4)
Livestock	2 (0.9)					2 (0.9)
Loxodonta africana	1 (0.4)					1 (0.4)
Lupus europeus	1 (0.4)					1 (0.4)
Macroscelides proboscideus	1 (0.4)					1 (0.4)
Masked weaver	1 (0.4)					1 (0.4)
Mastomys coucha	1 (0.4)					1 (0.4)
Mastomys erytholeucus	1 (0.4)					1 (0.4)
Mastomys natalensis	1 (0.4)					1 (0.4)
Mixed animals	7 (3.0)					7 (3.0)
Moorhen	1 (0.4)					1 (0.4)
Myodes glareolus	1 (0.4)					1 (0.4)
Ostriches	2 (0.9)					2 (0.9)
Otocyon megalotis	1 (0.4)					1 (0.4)
Otomys angoniensis	1 (0.4)					1 (0.4)
Otomys unisulcatus	1 (0.4)					1 (0.4)
Passer luteus	1 (0.4)					1 (0.4)
Pedetes capensis	1 (0.4)					1 (0.4)
Phacochoerus aethiopicus	1 (0.4)					1 (0.4)
Pig	1 (0.4)					1 (0.4)
Procavia capensis	1 (0.4)					1 (0.4)
Pronolagus crassicaudatus	1 (0.4)					1 (0.4)
Pronolagus rupestris	1 (0.4)					1 (0.4)
Pterocles exustus	1 (0.4)					1 (0.4)
Rattus rattus	2 (0.9)					2 (0.9)
Red bishop	1 (0.4)					1 (0.4)
Redbilled quelea	1 (0.4)					1 (0.4)
Rhabdomys pumilio	1 (0.4)					1 (0.4)
Rodents	1 (0.4)					1 (0.4)
Sable	1 (0.4)					1 (0.4)
Sacred ibis	1 (0.4)					1 (0.4)
Sheep	48 (20.5)					48 (20.5)
Small ruminants	1 (0.4)					1 (0.4)

Characteristics	Overall (802)	Humans CFR (53)	Humans prevlence (270)	Individual tick (209)	Tick pools (36)	Other animals (234)
Streptopelia species	1 (0.4)					1 (0.4)
Tatera leucogaster	1 (0.4)					1 (0.4)
Tockus erythrorhynchus	1 (0.4)					1 (0.4)
Unclassified small mammals	1 (0.4)					1 (0.4)
Warthog	1 (0.4)					1 (0.4)
White rhino	1 (0.4)					1 (0.4)
Wild dog	1 (0.4)					1 (0.4)
Xerus inauris	1 (0.4)					1 (0.4)
Yellowbilled duck	1 (0.4)					1 (0.4)
Zebra	1 (0.4)					1 (0.4)
**Detection assay**						
Agar gel diffusion precipitation test	5 (0.6)		2 (0.7)			3 (1.3)
Agar gel diffusion precipitation test, Indirect immunofluorescence assay	3 (0.4)					3 (1.3)
Classical RT-PCR	181 (22.4)	11 (20.8)	27 (10.0)	121 (57.9)	18 (47.2)	4 (1.7)
Classical RT-PCR, Culture, Indirect ELISA	1 (0.1)	1 (1.9)				
Classical RT-PCR, Direct ELISA, Indirect ELISA	1 (0.1)	1 (1.9)				
Classical RT-PCR, Indirect ELISA	27 (3.4)	18 (34.0)	9 (3.3)			
Classical RT-PCR, Indirect ELISA, Direct ELISA	1 (0.1)	1 (1.9)				
Classical RT-PCR, Indirect ELISA, Real Time RT-PCR	2 (0.3)	1 (1.9)	1 (0.4)			
Classical RT-PCR, Real Time RT-PCR	4 (0.5)	1 (1.9)	3 (1.1)			
Classical RT-PCR; Direct ELISA; Culture	1 (0.1)		1 (0.4)			
Classical RT-PCR; Real Time RT-PCR	1 (0.1)		1 (0.4)			
Complement fixation test	14 (1.8)		2 (0.7)			12 (5.1)
Complement fixation test, Culture, Indirect immunofluorescence assay	1 (0.1)	1 (1.9)				
Complement fixation test; Immunoflorescent assay	1 (0.1)		1 (0.4)			
Culture	20 (2.5)		3 (1.1)	9 (4.3)	7 (19.4)	1 (0.4)
Culture, Classical RT-PCR, Direct ELISA	2 (0.3)			2 (1.0)		
Direct ELISA	24 (3.0)		2 (0.7)	17 (8.1)	5 (13.9)	
Enzyme immunoassay	2 (0.3)		2 (0.7)			
Hemagglutination inhibition test; Immunoflorescent assay	1 (0.1)		1 (0.4)			

**Characteristics**	**Overall (802)**	**Humans CFR (53)**	**Humans prevlence (270)**	**Individual tick (209)**	**Tick pools (36)**	**Other animals (234)**
Hemagglutination inhibition test; Indirect immunofluorescence assay	1 (0.1)		1 (0.4)			
Immunodiffusion	1 (0.1)					1 (0.4)
Immunoflorescent assay	1 (0.1)		1 (0.4)			
Immunoflorescent assay, Dot-Blot Assay	4 (0.4)					4 (1.3)
Immunofluorescence hemocytes (IFH) assay, Classical RT-PCR	11 (1.4)			11 (5.3)		
Indirect ELISA	277 (34.5)	8 (15.1)	150 (55.6)			119 (50.9)
Indirect ELISA, Classical RT-PCR, Culture	2 (0.3)		2 (0.7)			
Indirect ELISA, Complement fixation test	2 (0.3)		2 (0.7)			
Indirect ELISA, Immunoflorescent assay	1 (0.1)					1 (0.4)
Indirect ELISA, Indirect immunofluorescence assay	4 (0.5)					4 (1.7)
Indirect ELISA, Real Time RT-PCR	4 (0.5)	3 (5.7)	1 (0.4)			
Indirect ELISA; Indirect immunofluorescence assay, Recombinant antigen-assay; Indirect immunofluorescence assay, Authentic antigen-assay	1 (0.1)					1 (0.4)
Indirect immunofluorescence assay	58 (7.2)		32 (11.9)			26 (11.1)
Indirect immunofluorescence assay; Indirect ELISA	2 (0.3)		2 (0.7)			
Indirect immunofluorescence assay; Reverse passive hemagglutination inhibition assay (RPHI)	1 (0.1)		1 (0.4)			
Indirect immunofluorescence assay; Western blot	1 (0.1)		1 (0.4)			
Luminex Mag- Pix	1 (0.1)		1 (0.4)			
Neutralization test	4 (0.5)		1 (0.4)			3 (1.3)
Next-generation sequencing	1 (0.1)		1 (0.4)			
Plaque reduction neutralization test (PRNT)	1 (0.1)		1 (0.4)			
Real Time RT-PCR	75 (9.4)	5 (9.4)	14 (5.2)	49 (23.4)	6 (16.7)	1 (0.4)
Real Time RT-PCR, Indirect ELISA	2 (0.3)	2 (3.8)				
Real Time RT-PCR; Classical RT-PCR	1 (0.1)		1 (0.4)			
Real Time RT-PCR; Direct ELISA	3 (0.4)					3 (1.3)
Reverse passive hemagglutination inhibition assay (RPHI)	50 (6.2)		2 (0.7)			48 (20.5)
Unclear/Not reported	1 (0.1)		1 (0.4)			
**Target detected**						
Antibodies	139 (17.3)		40 (14.8)			99 (42.3)
Antibodies, Viral RNA	3 (0.4)	2 (3.8)	1 (0.4)			

**Characteristics**	**Overall (802)**	**Humans CFR (53)**	**Humans prevlence (270)**	**Individual tick (209)**	**Tick pools (36)**	**Other animals (234)**
Antigen	2 (0.3)		1 (0.4)	1 (0.5)		
IgG	218 (27.2)	2 (3.8)	98 (36.3)			118 (50.4)
IgG, IgM, Live virus, Viral RNA	3 (0.4)	1 (1.9)	2 (0.7)			
IgG, IgM, Viral RNA	6 (0.8)	4 (7.6)	2 (0.7)			
IgG, Viral RNA	1 (0.1)	1 (1.9)	56 (20.7)			
IgM	68 (8.5)	6 (11.3)	9 (3.3)			6 (2.6)
IgM and IgG	10 (1.3)		1 (0.4)			1 (0.4)
IgM and IgG, Viral antigen, Viral RNA	2 (0.3)	1 (1.9)	7 (2.6)			
IgM, Viral RNA	25 (3.1)	18 (34.0)	3 (1.1)			
Live virus	23 (2.9)	1 (1.9)	2 (0.7)	11 (5.3)	7 (19.4)	1 (0.4)
Viral antigen	23 (2.9)			16 (7.7)	5 (13.9)	
Viral antigen, Viral RNA	11 (1.4)			11 (5.3)		
Viral RNA	263 (32.8)	16 (30.2)	47 (17.4)	170 (81.3)	24 (66.7)	6 (2.6)
Viral RNA, IgM	1 (0.1)	1 (1.9)				
Viral RNA; Antigen	3 (0.4)					3 (1.3)
Viral RNA; Live virus; Viral antigen	1 (0.1)		1 (0.4)			
**Infection Status**						
Current infection	367 (45.8)	45 (84.9)	67 (24.8)	209 (100.0)	36 (100.0)	10 (4.3)
Past infection	367 (45.8)	2 (3.8)	147 (54.4)			218 (93.2)
Recent infection	68 (8.5)	6 (11.3)	56 (20.7)			6 (2.6)
**Sample types**						
Organ tissue	1 (0.1)					1 (0.4)
Organ tissue, Serum	4 (0.5)	3 (5.7)	1 (0.4)			
Serum	540 (67.3)	44 (83.0)	263 (97.4)			233 (99.6)
Serum, Organ tissue	4 (0.5)	2 (3.8)	2 (0.7)			
Ticks	245 (30.6)		4 (1.5)	209 (100.0)	36 (100.0)	
Unclear/Not reported	8 (1.0)	4 (7.6)				
Characteristics	Frequence					
**Risk of bias**						
Low risk of bias	122 (15.2)	30 (56.6)	90 (33.3)			2 (0.9)
Moderate risk of bias	680 (84.8)	23 (43.4)	180 (66.7)	209 (100.0)	36 (100.0)	232 (99.2)
