# Supplementary material for: Worldwide epidemiology of Crimean-Congo Hemorrhagic Fever Virus in humans, ticks and other animal species, a systematic review and meta-analysis
Source: PLoS Negl Trop Dis. 2021 Apr 22;15(4):e0009299. doi: 10.1371/journal.pntd.0009299 (PMC8096040; doi:10.1371/journal.pntd.0009299)
Supplement: S8 Table — (PDF) [file pntd.0009299.s012.pdf]

S8 Table. Subgroup analyses of worldwide case fatality rate and prevalence of Crimean-congo hemorrhagic fever virus in humans, ticks, and other animals

	Prevalence. % (95%CI)	95% Prediction interval	N Studies	N Participants	H (95%CI)	I² (95%CI)	P heterogeneity	P difference subtypes
**Case fatality rate in humans**								
**Current infections**								
**Study Design**								0,302
Case-series	9.6 [4.1-17.1]	NA	1	83	NA	NA	1	
Case control	18 [7.3-32.1]	NA	2	213	2.2 [1.1-4.6]	79.4 [11.1-95.2]	0,027	
Community outbreak	30 [5-62.5]	NA	1	10	NA	NA	1	
Cross sectional	11 [8.3-14]	[0.2-31.6]	39	7747	3.5 [3.1-3.9]	91.6 [89.5-93.3]	< 0.001	
Hospital outbreak	20.9 [2.2-49.1]	NA	2	43	1.8 [1-3.8]	68.7 [0-92.9]	0,074	
**Sampling**								0,002
Non probabilistic	11.3 [8.7-14.2]	[0.3-31.7]	44	8065	3.3 [3-3.7]	90.8 [88.6-92.6]	< 0.001	
Probabilistic	35.5 [19.4-53.3]	NA	1	31	NA	NA	1	
**Timing of data collection**								0,178
Prospectively	9.9 [6.7-13.5]	[0.3-27.6]	20	2778	2.5 [2.1-3]	84.1 [76.6-89.2]	< 0.001	
Retrospectively	13.8 [9.5-18.6]	[0.1-40.8]	24	4918	3.9 [3.4-4.5]	93.5 [91.4-95]	< 0.001	
**Country**								< 0.001
Afghanistan	34.3 [21.8-48]	[0-100]	3	381	2 [1.1-3.7]	75.9 [20.6-92.7]	0,016	
India	32.4 [18.5-47.9]	[0-100]	3	43	1 [1-1]	0 [0-0]	0,988	
Iran	15.1 [12.5-17.8]	[6.9-25.5]	4	1692	1.3 [1-2.3]	42.6 [0-80.7]	0,156	
Pakistan	18 [1-46]	NA	2	98	2.2 [1.1-4.6]	79.1 [9.5-95.2]	0,029	
Replublic of Kosovo	34.4 [18.7-51.8]	NA	1	32	NA	NA	1	
South Africa	35.5 [19.4-53.3]	NA	1	31	NA	NA	1	
Turkey	7.2 [5.7-8.8]	[1.8-15.2]	31	5819	1.8 [1.5-2.2]	69.8 [56.5-79.1]	< 0.001	
**Country income level**								< 0.001
Low-income economies	34.3 [21.8-48]	[0-100]	3	381	2 [1.1-3.7]	75.9 [20.6-92.7]	0,016	
Lower-middle income economies	24.8 [10.8-41.7]	[0-81.1]	5	141	1.8 [1.1-2.8]	67.6 [16.3-87.5]	0,015	
Upper-middle-income economies	9.2 [7.3-11.3]	[1.3-22.2]	37	7574	2.5 [2.2-2.9]	84.5 [79.6-88.3]	< 0.001	
**WHO Region**								< 0.001
Africa	35.5 [19.4-53.3]	NA	1	31	NA	NA	1	
Eastern Mediterranean	21.9 [14.3-30.5]	[1.6-54.6]	9	2171	3.9 [3.1-4.9]	93.5 [89.7-95.8]	< 0.001	

	Prevalence. % (95%CI)	95% Prediction interval	N Studies	N Participants	H (95%CI)	I² (95%CI)	P heterogeneity	P difference subtypes
Europe	7.2 [5.7-8.8]	[1.8-15.2]	31	5819	1.8 [1.5-2.2]	69.8 [56.5-79.1]	< 0.001	
South-East Asia	32.4 [18.5-47.9]	[0-100]	3	43	1 [1-1]	0 [0-0]	0,988	
**Age range (years)**								< 0.001
Adults: 19+ years	11.3 [7.2-16]	[1.6-26.9]	5	478	1.4 [1-2.3]	48.6 [0-81.2]	0,1	
Child: Birth-18 years	0.2 [0-3.5]	[0-73.4]	3	110	1.1 [1-3.5]	22.6 [0-91.9]	0,275	
**Recrutment setting**								0,075
Rural	5.4 [1.5-11.1]	NA	1	93	NA	NA	1	
Urban	11.2 [7.7-15.1]	[2.1-25.3]	10	1350	1.9 [1.4-2.6]	72.6 [48.3-85.5]	< 0.001	
**Hospitalization**								< 0.001
Ambulatory	34.7 [21.2-49.5]	NA	2	46	1	0	0,915	
Hospitalized	10 [6.8-13.7]	[0-34.2]	31	4122	3.3 [2.9-3.8]	91 [88.3-93.1]	< 0.001	
**Population characteristics**								0,379
Positive among CCHFV suspected cases	11.5 [8.9-14.4]	[0.4-32.2]	42	8048	3.4 [3.1-3.8]	91.4 [89.3-93.1]	< 0.001	
Positive among febrile patients	15.4 [0.4-41]	NA	1	13	NA	NA	1	
Positive among Healtcare workers	16 [3.8-33.4]	NA	1	25	NA	NA	1	
Positive among patient with hemorrhagic symptoms	30 [5-62.5]	NA	1	10	NA	NA	1	
**Recent infections**								
**Timing of data collection**								0,001
Prospectively	2.4 [0-6.9]	[0-21.7]	4	155	1.2 [1-1.9]	26.5 [0-72.2]	0,253	
Retrospectively	28.9 [11.6-49.5]	NA	2	24	1	0	0,498	
**Country**								< 0.001
Iran	21.7 [6.2-41.9]	NA	2	23	1	0	0,893	
Iraq	36.4 [10-67.3]	NA	1	11	NA	NA	1	
Turkey	2.1 [0.1-5.4]	[0-41.1]	3	145	1 [1-1]	0 [0-0]	0,994	
**WHO Region**								< 0.001
Eastern Mediterranean	26.2 [11.8-43.2]	[0-100]	3	34	1 [1-1.8]	0 [0-70.3]	0,705	
Europe	2.1 [0.1-5.4]	[0-41.1]	3	145	1 [1-1]	0 [0-0]	0,994	
**Population characteristics**								0,089
Positive among CCHFV suspected cases	9.8 [0.3-26.9]	[0-92.8]	4	117	2.1 [1.3-3.5]	78.1 [40.8-91.9]	0,003	
Positive among febrile patients	1.9 [0-8.1]	NA	1	52	NA	NA	1	

	Prevalence. % (95%CI)	95% Prediction interval	N Studies	N Participants	H (95%CI)	I² (95%CI)	P heterogeneity	P difference subtypes
Positive among patient with hemorrhagic symptoms	20 [0.5-51.3]	NA	1	10	NA	NA	1	
**Past infections**								
**Country**								0,023
Iran	20 [0.5-51.3]	NA	1	10	NA	NA	1	
Turkey	0 [0-5.5]	NA	1	31	NA	NA	1	
**WHO Region**								0,023
Eastern Mediterranean	20 [0.5-51.3]	NA	1	10	NA	NA	1	
Europe	0 [0-5.5]	NA	1	31	NA	NA	1	
**Population characteristics**								0,023
Positive among CCHFV suspected cases	0 [0-5.5]	NA	1	31	NA	NA	1	
Positive among patient with hemorrhagic symptoms	20 [0.5-51.3]	NA	1	10	NA	NA	1	
**CCHFV prevalence in humans**								
**Current infections**								
**Study Design**								< 0.001
Community outbreak	13.3 [1.5-32.2]	[0-89.7]	5	416	4 [2.9-5.5]	93.8 [88.4-96.7]	< 0.001	
Cross sectional	22.4 [15.1-30.7]	[0-88]	58	33961	15.4 [14.9-16]	99.6 [99.5-99.6]	< 0.001	
Hospital outbreak	51.7 [38.5-64.9]	[0-100]	3	58	1 [1-1.6]	0 [0-61.6]	0,763	
**Sampling**								< 0.001
Non probabilistic	24.1 [16.8-32.2]	[0-89.4]	64	33857	14.7 [14.2-15.2]	99.5 [99.5-99.6]	< 0.001	
Probabilistic	0.1 [0-0.7]	NA	2	578	1	0	0,546	
**Timing of data collection**								0,019
Prospectively	16.9 [9.4-26]	[0-85]	44	24439	14 [13.4-14.6]	99.5 [99.4-99.5]	< 0.001	
Retrospectively	34.8 [22.9-47.8]	[0-91.9]	21	9979	11.6 [10.8-12.5]	99.3 [99.1-99.4]	< 0.001	
**Country**								< 0.001
Afghanistan	51.6 [9.4-92.5]	[0-100]	3	972	7.3 [5.4-9.8]	98.1 [96.5-99]	< 0.001	
Albania	32.4 [17.5-49.2]	NA	1	34	NA	NA	1	
Central Africa Republic	0 [0-0.9]	NA	1	198	NA	NA	1	
Ghana	0 [0-0.6]	NA	1	285	NA	NA	1	
India	19.2 [0.1-54.1]	[0-100]	4	332	5.4 [4-7.2]	96.5 [93.6-98.1]	< 0.001	
Iran	26.5 [18.3-35.6]	[3.9-59]	7	5319	5.8 [4.7-7.1]	97 [95.5-98]	< 0.001	
Kazakhstan	12.5 [0.3-34.1]	NA	1	16	NA	NA	1	

	Prevalence. % (95%CI)	95% Prediction interval	N Studies	N Participants	H (95%CI)	I² (95%CI)	P heterogeneity	P difference subtypes
Mali	0.4 [0-1.2]	NA	1	489	NA	NA	1	
Mauritania	11.5 [0-49]	[0-100]	3	327	7.3 [5.4-9.9]	98.1 [96.6-99]	< 0.001	
Nigeria	0.3 [0-1.1]	NA	1	380	NA	NA	1	
Pakistan	11.4 [3.5-22.7]	[0-61.5]	8	2624	6.8 [5.8-8.1]	97.9 [97-98.5]	< 0.001	
Replublic of Kosovo	25 [14.4-37.3]	NA	1	56	NA	NA	1	
Russia	33.3 [11.3-59.5]	NA	1	15	NA	NA	1	
Saudi Arabia	0 [0-2.1]	NA	1	80	NA	NA	1	
Senegal	0 [0-0]	NA	1	13845	NA	NA	1	
Sierra Leone	1 [0-4.4]	NA	1	96	NA	NA	1	
South Africa	0.4 [0-1.3]	NA	2	448	1	0	0,464	
Sudan	16.9 [8.7-27.1]	NA	1	65	NA	NA	1	
Tunisia	0 [0-0.9]	NA	1	181	NA	NA	1	
Turkey	42 [27.3-57.4]	[0-99.7]	22	8608	12.4 [11.6-13.3]	99.4 [99.3-99.4]	< 0.001	
United Arab Emirates	41.4 [24.1-59.7]	[0-100]	3	48	1.2 [1-2.2]	35.8 [0-79.4]	0,211	
**Country income level**								< 0.001
High-income economies	24.7 [0-67.6]	[0-100]	4	128	4.3 [3-6]	94.5 [89-97.2]	< 0.001	
Low-income economies	17.1 [1.6-42.9]	[0-99.6]	7	1820	10.8 [9.4-12.4]	99.1 [98.9-99.3]	< 0.001	
Lower-middle income economies	8.2 [2.8-15.8]	[0-55.5]	19	17974	10 [9.2-10.9]	99 [98.8-99.2]	< 0.001	
Upper-middle-income economies	33.8 [25-43.2]	[0-87.5]	35	14496	10.7 [10.1-11.4]	99.1 [99-99.2]	< 0.001	
**WHO Region**								< 0.001
Africa	1.3 [0.1-3.5]	[0-13.8]	11	16068	4.5 [3.7-5.4]	95 [92.8-96.6]	< 0.001	
Eastern Mediterranean	20.7 [14.1-28.2]	[0-62.4]	24	9289	7.6 [6.9-8.3]	98.3 [97.9-98.5]	< 0.001	
Europe	40.1 [26.5-54.6]	[0-99.2]	25	8673	11.6 [10.9-12.4]	99.3 [99.2-99.4]	< 0.001	
South-East Asia	19.2 [0.1-54.1]	[0-100]	4	332	5.4 [4-7.2]	96.5 [93.6-98.1]	< 0.001	
**Age range (years)**								0,709
Adults: 19+ years	34.2 [0.1-85.8]	[0-100]	4	693	12.8 [10.7-15.1]	99.4 [99.1-99.6]	< 0.001	
Child: Birth-18 years	23.7 [7.8-44.5]	[0-94]	6	1691	7.7 [6.4-9.3]	98.3 [97.5-98.8]	< 0.001	
**Recrutment setting**								0,716
Rural	24.8 [0-72.6]	[0-100]	3	251	7.5 [5.6-10.1]	98.2 [96.8-99]	< 0.001	
Urban	34.1 [14.8-56.4]	[0-100]	11	5723	11.2 [10.1-12.4]	99.2 [99-99.3]	< 0.001	
**Hospitalization**								0,163
Ambulatory	11.3 [0-47.2]	[0-100]	3	14033	9.9 [7.7-12.7]	99 [98.3-99.4]	< 0.001	
Hospitalized	37 [23.8-51.2]	[0-98.7]	27	9794	12.6 [11.9-13.4]	99.4 [99.3-99.4]	< 0.001	
**Population characteristics**								< 0.001

	Prevalence. % (95%CI)	95% Prediction interval	N Studies	N Participants	H (95%CI)	I² (95%CI)	P heterogeneity	P difference subtypes
Apparently healthy individuals	1.2 [0-5]	NA	1	84	NA	NA	1	
CCHFV positive patient	14 [5.3-25.9]	[0-100]	3	4563	3.1 [1.9-5.1]	89.7 [72.3-96.2]	< 0.001	
CCHFV suspected cases	36.9 [30.1-44]	[3.9-79.4]	38	12529	7.5 [7-8.1]	98.2 [98-98.5]	< 0.001	
Febrile patients	2.2 [0.6-4.6]	[0-15.1]	14	16280	4 [3.4-4.8]	93.8 [91.1-95.6]	< 0.001	
Healtcare workers	49 [35.3-62.8]	NA	1	51	NA	NA	1	
High risk individuals	0 [0-2.1]	NA	1	80	NA	NA	1	
Patient with hemorrhagic symptoms	12.5 [0.9-32.1]	[0-91.4]	5	506	4.4 [3.3-6]	94.9 [90.9-97.2]	< 0.001	
**Recent infections**								
**Study Design**								< 0.001
Cohort (Baseline data)	0 [0-1.3]	NA	1	132	NA	NA	1	
Community outbreak	2.8 [0-9.5]	[0-35.8]	5	351	2 [1.3-3.2]	76 [41.3-90.2]	0,002	
Cross sectional	11.4 [7.4-16.1]	[0-52.2]	47	25895	8.9 [8.4-9.4]	98.7 [98.6-98.9]	< 0.001	
Hospital outbreak	56.3 [38.3-73.5]	NA	2	32	1	0	0,494	
**Sampling**								< 0.001
Non probabilistic	12.5 [8.1-17.6]	[0-58.4]	51	23587	8.6 [8.1-9.1]	98.6 [98.5-98.8]	< 0.001	
Probabilistic	1.6 [0.2-4.1]	[0-22.7]	4	2823	4 [2.8-5.7]	93.7 [87-96.9]	< 0.001	
**Timing of data collection**								0,155
Prospectively	8.7 [5.6-12.3]	[0-39.7]	44	25207	7.4 [6.9-7.9]	98.2 [97.9-98.4]	< 0.001	
Retrospectively	21.1 [5.4-42.8]	[0-96.9]	10	1186	7.5 [6.5-8.6]	98.2 [97.6-98.6]	< 0.001	
**Country**								< 0.001
Afghanistan	24.7 [5.5-50.9]	[0-100]	3	189	3.4 [2.1-5.4]	91.3 [77.5-96.6]	< 0.001	
Bulgaria	1.7 [0-5.3]	NA	2	543	1.1	16.7	0,273	
Democratic Republic of the Congo	0 [0-3.8]	NA	1	45	NA	NA	1	
Georgia	5.2 [0-40.8]	NA	2	449	3.3 [1.8-6.3]	91 [68-97.5]	0,001	
Ghana	0.9 [0-3.9]	NA	1	108	NA	NA	1	
India	5.1 [0-19.2]	[0-100]	3	123	1.9 [1-3.5]	72.1 [5.9-91.8]	0,028	
Iran	8.4 [2.1-17.8]	[0-50]	7	1598	4.6 [3.6-5.8]	95.2 [92.3-97]	< 0.001	
Iraq	28.2 [15-43.5]	NA	1	39	NA	NA	1	
Kazakhstan	0.5 [0.2-1.2]	NA	1	914	NA	NA	1	
Kenya	0.1 [0-0.4]	NA	2	896	1	0	0,417	
Madagascar	0.1 [0-0.2]	NA	1	1995	NA	NA	1	
Mali	4.8 [2.8-7.2]	NA	1	376	NA	NA	1	
Mauritania	2 [0.6-4.1]	NA	2	283	1	0	0,402	
Nigeria	4.1 [3.1-5.3]	NA	1	1189	NA	NA	1	

	Prevalence. % (95%CI)	95% Prediction interval	N Studies	N Participants	H (95%CI)	I² (95%CI)	P heterogeneity	P difference subtypes
Pakistan	19.5 [8.9-32.8]	[0-100]	3	261	2.4 [1.3-4.1]	81.9 [44.2-94.2]	0,004	
Senegal	0.1 [0-1.6]	NA	2	14128	3 [1.5-5.8]	88.7 [57.2-97]	0,003	
Sudan	4.2 [0-15]	NA	2	269	2.7 [1.3-5.3]	85.8 [42.9-96.5]	0,008	
Tunisia	2.8 [0.8-5.8]	NA	1	181	NA	NA	1	
Turkey	25.4 [11.2-42.9]	[0-95.8]	16	2776	9.5 [8.6-10.4]	98.9 [98.6-99.1]	< 0.001	
United Arab Emirates	31.2 [0-79.5]	[0-100]	3	48	3.4 [2.1-5.5]	91.5 [78.1-96.7]	< 0.001	
**Country income level**								0,003
High-income economies	31.2 [0-79.5]	[0-100]	3	48	3.4 [2.1-5.5]	91.5 [78.1-96.7]	< 0.001	
Low-income economies	7.4 [1.1-17.6]	[0-55.6]	8	2874	6.1 [5.1-7.4]	97.3 [96.2-98.2]	< 0.001	
Lower-middle income economies	3.3 [1-6.6]	[0-22.5]	15	17169	5.6 [4.9-6.4]	96.8 [95.8-97.6]	< 0.001	
Upper-middle-income economies	16.4 [9.2-25.2]	[0-72.7]	29	6319	8.2 [7.6-8.9]	98.5 [98.3-98.7]	< 0.001	
**WHO Region**								< 0.001
Africa	0.8 [0-2.1]	[0-8.8]	11	19020	5.2 [4.4-6.1]	96.3 [94.7-97.3]	< 0.001	
Eastern Mediterranean	14.2 [8.3-21.1]	[0-51.6]	20	2585	4.1 [3.6-4.8]	94.1 [92.2-95.6]	< 0.001	
Europe	18.9 [9-31.2]	[0-83.5]	21	4682	9.4 [8.6-10.2]	98.9 [98.7-99]	< 0.001	
South-East Asia	5.1 [0-19.2]	[0-100]	3	123	1.9 [1-3.5]	72.1 [5.9-91.8]	0,028	
**Age range (years)**								0,175
Adults: 19+ years	3.9 [0.1-11.5]	[0-44.1]	6	1676	5.2 [4.1-6.6]	96.3 [94-97.7]	< 0.001	
Child: Birth-18 years	37.2 [0-93]	NA	2	948	8.1 [5.4-11.9]	98.5 [96.6-99.3]	< 0.001	
**Recrutment setting**								0,422
Rural	3.5 [0.1-10.4]	[0-41.2]	7	2562	6.8 [5.6-8.1]	97.8 [96.8-98.5]	< 0.001	
Urban	6.4 [1.7-13.6]	[0-42.2]	10	1712	4.7 [3.9-5.7]	95.4 [93.3-96.9]	< 0.001	
**Hospitalization**								< 0.001
Ambulatory	0 [0-0.2]	[0-1.1]	8	15350	1.6 [1.1-2.3]	59.4 [11.4-81.4]	0,016	
Hospitalized	39.3 [23.5-56.3]	[0-97.4]	14	1009	5.3 [4.5-6.1]	96.4 [95.1-97.3]	< 0.001	
**Population characteristics**								< 0.001
Apparently healthy individuals	1.5 [0.4-3.3]	[0-9.8]	7	2366	2.6 [1.9-3.6]	85.5 [72-92.5]	< 0.001	
CCHFV positive case contact	0.3 [0-1.9]	NA	2	231	1.2	27	0,242	
CCHFV suspected cases	32.4 [21.1-44.9]	[0-89.7]	22	2663	6.1 [5.5-6.8]	97.3 [96.7-97.8]	< 0.001	
Febrile patients	4.3 [0.8-10.1]	[0-35]	10	16024	7.3 [6.3-8.4]	98.1 [97.5-98.6]	< 0.001	
Healtcare workers	0 [0-0.4]	[0-9.9]	3	488	1 [1-1]	0 [0-0]	0,934	
High risk individuals	0.2 [0-1]	[0-31]	3	3017	2.1 [1.1-3.8]	76.8 [24.3-92.9]	0,013	
Patient with hemorrhagic symptoms	8.8 [0.4-23.6]	[0-72.8]	5	308	2.6 [1.8-3.9]	85.3 [67.5-93.4]	< 0.001	
**Past infections**								

	Prevalence. % (95%CI)	95% Prediction interval	N Studies	N Participants	H (95%CI)	I² (95%CI)	P heterogeneity	P difference subtypes
**Study Design**								< 0.001
Case control	13.6 [11-16.4]	NA	1	625	NA	NA	1	
Cohort (Baseline data)	12.1 [7-18.3]	NA	1	132	NA	NA	1	
Community outbreak	3.1 [0-12.1]	[0-48.7]	5	188	2.1 [1.4-3.3]	77.4 [45.4-90.6]	0,001	
Cross sectional	4.1 [3.1-5.2]	[0-21.7]	134	72813	6.5 [6.2-6.7]	97.6 [97.4-97.8]	< 0.001	
Hospital outbreak	7.2 [0.4-19]	[0-62.9]	5	379	2.9 [2-4.3]	88.4 [75.5-94.5]	< 0.001	
**Sampling**								0,638
Non probabilistic	4.3 [3.1-5.6]	[0-24.1]	108	51693	6.5 [6.2-6.8]	97.6 [97.4-97.8]	< 0.001	
Probabilistic	4 [2.6-5.7]	[0-18.1]	38	22444	5.5 [5-6]	96.7 [96.1-97.2]	< 0.001	
**Timing of data collection**								0,021
Prospectively	4 [3.1-5.1]	[0-21.3]	129	70772	6.4 [6.1-6.7]	97.6 [97.3-97.8]	< 0.001	
Retrospectively	11.7 [4.3-21.7]	[0-54.9]	10	1584	4.5 [3.7-5.5]	95.1 [92.7-96.7]	< 0.001	
**Country**								< 0.001
Afghanistan	4.1 [0.3-11.2]	[0-57.6]	4	1714	5.4 [4-7.3]	96.5 [93.6-98.1]	< 0.001	
Albania	32.4 [17.5-49.2]	NA	1	34	NA	NA	1	
Bulgaria	3 [2-4.1]	[0.5-7]	5	3812	1.6 [1-2.6]	61.1 [0-85.4]	0,036	
Cameroon	1.4 [0-8.4]	NA	2	1289	3.8 [2.1-6.9]	93.1 [77.4-97.9]	< 0.001	
Central Africa Republic	0 [0-0.1]	NA	2	4622	1	0	0,87	
Chad	0 [0-0.5]	NA	1	334	NA	NA	1	
China	2.6 [1.1-4.7]	NA	2	4111	3.7 [2-6.8]	92.7 [75.3-97.8]	< 0.001	
Democratic Republic of the Congo	0.2 [0-0.8]	NA	2	773	1	0	0,953	
Djibouti	0 [0-1.6]	NA	1	108	NA	NA	1	
Equatorial Guinea	0.7 [0.2-1.5]	NA	1	688	NA	NA	1	
Gabon	0 [0-0.2]	NA	1	841	NA	NA	1	
Georgia	2.8 [1.4-4.5]	NA	1	435	NA	NA	1	
Ghana	3.7 [0.8-8.3]	NA	1	108	NA	NA	1	
Greece	3.4 [2.2-4.9]	[0.5-8.2]	11	5878	1.8 [1.3-2.5]	69.6 [43.4-83.7]	< 0.001	
Hungary	2.9 [1.7-4.4]	NA	1	587	NA	NA	1	
India	0.2 [0-1]	[0-33.5]	3	4978	2.9 [1.8-4.9]	88.3 [67.6-95.8]	< 0.001	
Iran	7.2 [1.8-15.2]	[0-54.3]	16	3192	6.7 [6-7.6]	97.8 [97.2-98.3]	< 0.001	
Iraq	12.2 [9.3-15.5]	NA	1	441	NA	NA	1	
Kazakhstan	5.9 [0-21.3]	NA	2	1716	9.9 [7-14]	99 [98-99.5]	< 0.001	
Kenya	7.1 [0.1-23.2]	[0-97.4]	4	3459	12.9 [10.9-15.3]	99.4 [99.2-99.6]	< 0.001	
Kuwait	3.4 [2-5.2]	NA	1	501	NA	NA	1	

	Prevalence. % (95%CI)	95% Prediction interval	N Studies	N Participants	H (95%CI)	I² (95%CI)	P heterogeneity	P difference subtypes
Madagascar	0.7 [0.4-1.1]	NA	2	2376	1	0	0,784	
Malaysia	0 [0-0.4]	NA	2	767	1	0	0,892	
Mali	2.9 [1.4-4.9]	NA	1	376	NA	NA	1	
Mauritania	7.3 [0-38.6]	[0-100]	3	1123	8.2 [6.2-10.9]	98.5 [97.4-99.2]	< 0.001	
Mongolia	1.4 [0.9-2]	NA	1	1926	NA	NA	1	
Mozambique	2.7 [1.1-4.8]	NA	1	300	NA	NA	1	
Nigeria	4.4 [0-15]	[0-80.4]	4	3413	10 [8.1-12.2]	99 [98.5-99.3]	< 0.001	
Oman	15.4 [1.7-38.5]	NA	2	401	4.9 [2.9-8.3]	95.9 [88.3-98.6]	< 0.001	
Pakistan	5 [0-16.2]	[0-60.3]	8	1144	5.5 [4.5-6.7]	96.7 [95.1-97.8]	< 0.001	
Portugal	0.8 [0-2.3]	NA	1	258	NA	NA	1	
Replublic of Kosovo	4 [2.9-5.2]	NA	1	1105	NA	NA	1	
Russia	11.1 [5.3-18.5]	NA	1	90	NA	NA	1	
Saudi Arabia	9.7 [1.5-23.4]	[0-87.2]	4	1528	5.8 [4.3-7.7]	97 [94.7-98.3]	< 0.001	
Senegal	9.1 [3.3-17.2]	NA	2	1300	3.7 [2-6.8]	92.6 [75.1-97.8]	< 0.001	
Sierra Leone	0.7 [0-3.9]	NA	2	861	2.9 [1.5-5.6]	87.8 [52.9-96.8]	0,004	
Somalia	0 [0-6.1]	NA	1	28	NA	NA	1	
South Africa	0.8 [0.3-1.6]	[0-3.5]	7	2891	1.5 [1-2.3]	56.6 [0-81.3]	0,032	
South Sudan	0 [0-0.3]	NA	1	632	NA	NA	1	
Spain	0 [0-0.8]	NA	1	228	NA	NA	1	
Sudan	4.8 [1.6-9.5]	[0-35.2]	4	1010	2.7 [1.7-4.2]	86.1 [66.1-94.3]	< 0.001	
Tunisia	1.1 [0-10.9]	NA	2	219	2.5 [1.2-5]	83.4 [30.9-96]	0,014	
Turkey	9.7 [6.4-13.5]	[0-31.5]	20	9531	5.3 [4.7-6]	96.4 [95.4-97.2]	< 0.001	
Uganda	4.4 [1.6-8.3]	NA	2	1807	1.9 [1-4]	71.7 [0-93.6]	0,06	
United Arab Emirates	3.1 [0.1-8.6]	[0-28.7]	8	699	2.6 [2-3.6]	85.7 [73.8-92.2]	< 0.001	
Zimbabwe	3.7 [2.2-5.6]	NA	1	486	NA	NA	1	
**Country income level**								0,001
High-income economies	3.9 [2.3-5.8]	[0-17]	29	10080	3.7 [3.3-4.2]	92.9 [90.8-94.4]	< 0.001	
Low-income economies	1.7 [0.7-2.9]	[0-10.7]	23	14833	4.6 [4.1-5.2]	95.3 [94-96.3]	< 0.001	
Lower-middle income economies	3.7 [1.9-6]	[0-22.5]	32	19553	6.9 [6.3-7.5]	97.9 [97.5-98.2]	< 0.001	
Upper-middle-income economies	5.9 [4.2-8]	[0-27.7]	61	29654	6.6 [6.2-7]	97.7 [97.4-98]	< 0.001	
**WHO Region**								< 0.001
Africa	2.3 [1.2-3.6]	[0-15.4]	40	27155	6.3 [5.8-6.8]	97.5 [97-97.8]	< 0.001	
Eastern Mediterranean	5.6 [3.1-8.8]	[0-38.3]	52	11509	6.1 [5.7-6.5]	97.3 [96.9-97.7]	< 0.001	
Europe	6.3 [4.7-8.2]	[0-21.6]	44	22569	4.9 [4.5-5.4]	95.9 [95.1-96.5]	< 0.001	

	Prevalence. % (95%CI)	95% Prediction interval	N Studies	N Participants	H (95%CI)	I² (95%CI)	P heterogeneity	P difference subtypes
South-East Asia	0.2 [0-1]	[0-33.5]	3	4978	2.9 [1.8-4.9]	88.3 [67.6-95.8]	< 0.001	
Western Pacific	1.2 [0.4-2.5]	[0-7.7]	5	6804	3.4 [2.4-4.8]	91.5 [83.2-95.7]	< 0.001	
**Age range (years)**								0,049
Adults: 19+ years	6.4 [3.6-9.9]	[0-30.1]	25	10562	5.9 [5.3-6.6]	97.1 [96.5-97.7]	< 0.001	
Child: Birth-18 years	2.2 [0.9-3.9]	[0-19.2]	3	528	1 [1-2.4]	0 [0-83.3]	0,537	
**Recrutment setting**								0,414
Rural	5.4 [2.8-8.8]	[0-27.8]	21	16564	7.8 [7.1-8.6]	98.4 [98-98.6]	< 0.001	
Urban	3.7 [2.3-5.5]	[0-15.4]	29	12371	4 [3.5-4.5]	93.7 [92-95.1]	< 0.001	
**Hospitalization**								0,026
Ambulatory	2.8 [1-5.3]	[0-21.8]	23	6170	4.7 [4.2-5.3]	95.5 [94.2-96.5]	< 0.001	
Hospitalized	13.7 [3.2-28.8]	[0-82.1]	13	1211	5.8 [5-6.7]	97 [96-97.8]	< 0.001	
**Population characteristics**								< 0.001
Apparently healthy individuals	2.7 [1.8-3.8]	[0-14.6]	58	42898	6 [5.7-6.4]	97.3 [96.9-97.6]	< 0.001	
Blood donors	1.1 [0-6.5]	NA	2	1172	4 [2.2-7.1]	93.7 [79.6-98]	< 0.001	
CCHFV positive case contact	9.2 [0-30.4]	[0-100]	4	431	5.1 [3.7-6.9]	96.1 [92.7-97.9]	< 0.001	
CCHFV suspected cases	32.5 [24.9-40.5]	[10.9-58.6]	10	1610	2.3 [1.7-3.1]	81.4 [66.9-89.6]	< 0.001	
Febrile patients	4.1 [1.6-7.6]	[0-27.4]	20	5888	5.4 [4.8-6.1]	96.5 [95.6-97.3]	< 0.001	
Healtcare workers	0.5 [0-1.5]	[0-4.1]	10	1220	1.4 [1-2]	49.5 [0-75.6]	0,037	
High risk individuals	3.8 [2.2-5.8]	[0-18.7]	31	16473	5.2 [4.8-5.8]	96.4 [95.6-97]	< 0.001	
Patient with any illness	3.6 [2.8-4.6]	NA	1	1657	NA	NA	1	
Patient with hemorrhagic symptoms	11.2 [0-41.8]	[0-100]	4	237	4.9 [3.6-6.7]	95.8 [92.1-97.8]	< 0.001	
**CCHFV prevalence in ticks**								
**Current infections**								
**Sampling**								0,008
Non probabilistic	1.5 [0.7-2.5]	[0-19.5]	136	22326	4 [3.8-4.2]	93.8 [93-94.4]	< 0.001	
Probabilistic	3.4 [2.1-5]	[0-19.2]	73	8791	2.6 [2.3-2.8]	85 [81.8-87.7]	< 0.001	
**Timing of data collection**								0,181
Prospectively	2.2 [1.4-3.1]	[0-21.7]	201	30846	3.9 [3.8-4.1]	93.6 [92.9-94.1]	< 0.001	
Retrospectively	0 [0-3.4]	NA	2	54	1	0	0,837	
**Country**								< 0.001
Albania	2.5 [0-10.3]	[0-45.3]	6	1088	4.8 [3.7-6.2]	95.6 [92.8-97.4]	< 0.001	
Algeria	28.6 [17.4-41.2]	NA	1	56	NA	NA	1	
Armenia	3.6 [0.4-9.2]	[0-30.5]	6	548	2.5 [1.8-3.6]	84.3 [67.5-92.4]	< 0.001	

	Prevalence. % (95%CI)	95% Prediction interval	N Studies	N Participants	H (95%CI)	I² (95%CI)	P heterogeneity	P difference subtypes
Bulgaria	1.9 [0.6-3.7]	[0-10.4]	13	2491	2.3 [1.8-2.9]	80.4 [67.5-88.2]	< 0.001	
China	0 [0-0]	[0-4.5]	3	1022	1 [1-1.5]	0 [0-57.6]	0,783	
Djibouti	14 [10-18.5]	[4.6-26.7]	8	935	1.5 [1-2.2]	54.4 [0-79.4]	0,032	
Egypt	0 [0-2.1]	NA	1	80	NA	NA	1	
France	0 [0-0.2]	NA	1	1015	NA	NA	1	
Greece	0.2 [0-2.1]	[0-3]	7	193	1 [1-1.3]	0 [0-39.9]	0,82	
India	1.4 [0-3.9]	[0-30.9]	3	214	1 [1-2.6]	0 [0-85.3]	0,492	
Iran	6.3 [4.6-8.2]	[0-19]	62	6720	1.8 [1.6-2.1]	69.3 [60.2-76.3]	< 0.001	
Israel	0 [0-0.7]	[0-3]	4	249	1 [1-1]	0 [0-0]	0,988	
Italy	0 [0-0]	[0-0.1]	5	892	1 [1-1.4]	0 [0-51.3]	0,789	
Kenya	0 [0-0]	[0-0]	5	3496	1 [1-1.8]	0 [0-69.7]	0,601	
Mauritania	0 [0-0]	[0-0.1]	5	891	1 [1-1.4]	0 [0-46.8]	0,815	
Oman	0 [0-1.2]	[0-32.4]	3	137	1 [1-1]	0 [0-0]	0,92	
Pakistan	3.7 [2.1-5.6]	[1.3-6.9]	5	525	1 [1-1]	0 [0-0]	0,95	
Poland	0 [0-0.1]	NA	2	993	1	0	0,563	
Replublic of Kosovo	1.8 [0-5.7]	[0-22.2]	8	1179	3.3 [2.5-4.3]	90.6 [83.8-94.5]	< 0.001	
South Africa	0 [0-11.9]	NA	1	14	NA	NA	1	
Spain	3.9 [0.3-10.3]	[0-41.1]	13	3971	6.8 [6-7.8]	97.8 [97.2-98.3]	< 0.001	
Sudan	0 [0-0.2]	NA	1	1005	NA	NA	1	
Tunisia	0 [0-1.4]	NA	1	120	NA	NA	1	
Turkey	0 [0-0]	[0-0]	39	2702	1 [1-1.2]	0 [0-31.8]	0,593	
Uganda	1.3 [0-10.6]	NA	2	46	1.3	36.7	0,209	
United Arab Emirates	0 [0-0]	[0-15.4]	3	290	1 [1-1.5]	0 [0-56.6]	0,787	
**Country income level**								0,071
High-income economies	0.6 [0-2]	[0-15.7]	38	7740	4 [3.6-4.5]	93.9 [92.4-95]	< 0.001	
Low-income economies	0 [0-4.3]	[0-100]	3	1051	1.7 [1-3.2]	64.9 [0-89.9]	0,058	
Lower-middle income economies	3 [0.9-6.1]	[0-27.4]	29	6317	4.4 [4-5]	94.9 [93.7-96]	< 0.001	
Upper-middle-income economies	2.3 [1.4-3.3]	[0-18]	138	15764	2.9 [2.7-3.1]	87.9 [86.1-89.4]	< 0.001	
**WHO Region**								< 0.001
Africa	3.6 [0.7-8]	[0-34.4]	22	5438	5 [4.4-5.6]	96 [94.9-96.8]	< 0.001	
Eastern Mediterranean	4.5 [2.8-6.5]	[0-24.6]	76	8877	2.9 [2.6-3.1]	87.9 [85.5-89.9]	< 0.001	
Europe	0.7 [0.1-1.6]	[0-15]	96	14142	3.5 [3.2-3.7]	91.7 [90.4-92.8]	< 0.001	
South-East Asia	1.4 [0-3.9]	[0-30.9]	3	214	1 [1-2.6]	0 [0-85.3]	0,492	
Western Pacific	0 [0-0]	[0-4.5]	3	1022	1 [1-1.5]	0 [0-57.6]	0,783	

	Prevalence. % (95%CI)	95% Prediction interval	N Studies	N Participants	H (95%CI)	I² (95%CI)	P heterogeneity	P difference subtypes
**Recrutment setting**								0,938
Rural	2.1 [0.8-3.7]	[0-19.4]	51	11352	4.2 [3.8-4.6]	94.3 [93.2-95.2]	< 0.001	
Urban	1.9 [0-7.1]	[0-37.5]	4	272	1.8 [1.1-3.1]	69.8 [13.3-89.5]	0,019	
**Gender**								< 0.001
Amblyomma	4.4 [0.5-11.2]	[0-37.4]	6	673	2.9 [2.1-4.1]	88.1 [76.5-93.9]	< 0.001	
Argas	0 [0-3.2]	NA	2	53	1	0	0,76	
Dermacentor	0.1 [0-1]	[0-5.4]	13	1836	1.9 [1.4-2.5]	71.7 [50.4-83.8]	< 0.001	
Haemaphysalis	0 [0-0.1]	[0-0.2]	14	548	1 [1-1.4]	0 [0-48]	0,591	
Hyalomma	2.5 [1.4-3.8]	[0-21.6]	104	17457	3.9 [3.6-4.1]	93.3 [92.4-94.1]	< 0.001	
Ixodes	0 [0-0.3]	[0-1.3]	9	1523	1.2 [1-1.7]	27.3 [0-66.2]	0,201	
Ornithodoros	11.4 [0-63.5]	NA	2	60	4.4 [2.5-7.6]	94.7 [83.9-98.3]	< 0.001	
Rhipicephalus	2 [0.7-3.8]	[0-20.7]	51	6847	3.2 [2.9-3.5]	90.2 [88-92.1]	< 0.001	
**Population characteristics**								< 0.001
Amblyomma cohaerens	17.6 [8.2-29.5]	NA	1	51	NA	NA	1	
Amblyomma gemma	0 [0-3.6]	NA	1	47	NA	NA	1	
Amblyomma lepidum	4.2 [0-21.7]	NA	2	404	3.6 [2-6.7]	92.3 [73.9-97.7]	< 0.001	
Amblyomma variegatum	0 [0-2.7]	NA	1	64	NA	NA	1	
Ambylomma variegatum	10.3 [5.1-16.8]	NA	1	107	NA	NA	1	
Argas persicus	0 [0-3.2]	NA	2	53	1	0	0,76	
Boophilus annulatus	0 [0-3.7]	NA	1	46	NA	NA	1	
Boophilus calcaratus	0 [0-2.2]	NA	1	76	NA	NA	1	
Boophilus decoloratus	5.6 [0-22.3]	NA	1	18	NA	NA	1	
Boophilus species	0 [0-1.3]	NA	2	96	1	0	0,621	
Boophylus annulatus	0 [0-4.5]	NA	1	38	NA	NA	1	
Dermacentor marginatus	0 [0-0]	[0-0.1]	9	924	1 [1-1.3]	0 [0-43.3]	0,761	
Dermacentor niveus	0 [0-0.3]	NA	1	644	NA	NA	1	
Dermacentor reticulatus	0 [0-1.4]	NA	1	125	NA	NA	1	
Dermacentor species	11.5 [5.8-18.7]	NA	1	96	NA	NA	1	
Dermacentor marginatus	0 [0-3.6]	NA	1	47	NA	NA	1	
Haemaphysalis concinna	0 [0-16.5]	NA	1	10	NA	NA	1	
Haemaphysalis erinacei	0 [0-3.8]	NA	1	45	NA	NA	1	
Haemaphysalis inermis	8.3 [0-32.4]	NA	1	12	NA	NA	1	
Haemaphysalis parva	0.5 [0-9]	NA	2	278	2 [1-4.2]	75.3 [0-94.4]	0,044	
Haemaphysalis punctata	0 [0-6.5]	NA	2	29	1	0	0,907	

	Prevalence. % (95%CI)	95% Prediction interval	N Studies	N Participants	H (95%CI)	I² (95%CI)	P heterogeneity	P difference subtypes
Haemaphysalis species	0 [0-7]	NA	1	24	NA	NA	1	
Haemaphysalis sulcata	0.1 [0-2.2]	[0-3.6]	6	150	1 [1-1]	0 [0-0]	0,961	
Hyalomma aegyptium	3.2 [0-15.5]	[0-67.5]	8	619	4.9 [3.9-6]	95.8 [93.6-97.2]	< 0.001	
Hyalomma anatolicum	6.5 [3.5-10]	[0.1-18.6]	15	572	1.3 [1-1.8]	45.1 [0-70]	0,03	
Hyalomma anatolicum anatolicum	0 [0-0.2]	[0-20.4]	3	218	1 [1-1.4]	0 [0-52.1]	0,805	
Hyalomma asiaticum	3.7 [1.3-6.9]	[0.3-9.2]	5	239	1 [1-1.8]	0 [0-69]	0,612	
Hyalomma detritum	5.7 [0-21.7]	[0-93.8]	4	461	3.7 [2.6-5.4]	92.9 [84.9-96.6]	< 0.001	
Hyalomma dromedari	0 [0-1.2]	NA	1	140	NA	NA	1	
Hyalomma dromedarii	1.1 [0-4.5]	[0-20.4]	11	1741	3.4 [2.7-4.3]	91.4 [86.7-94.5]	< 0.001	
Hyalomma excavatum	2.7 [0-11.2]	NA	2	145	1.2	29.5	0,234	
Hyalomma impeltatum	0 [0-2.2]	NA	2	85	1	0	0,836	
Hyalomma lusitanicum	3 [0-10.5]	[0-100]	3	1826	6 [4.2-8.4]	97.2 [94.5-98.6]	< 0.001	
Hyalomma marginatum	3 [0.5-6.7]	[0-27.9]	22	4184	4.4 [3.9-5]	94.9 [93.3-96.1]	< 0.001	
Hyalomma marginatum marginatum	4.5 [2.7-6.6]	NA	2	445	1	0	0,598	
Hyalomma marginatum rufipes	0 [0-0.6]	NA	2	356	1	0	0,763	
Hyalomma parva	0 [0-5.7]	NA	1	30	NA	NA	1	
Hyalomma punctata	0 [0-5.5]	NA	1	31	NA	NA	1	
Hyalomma rufipes	0 [0-0.3]	NA	2	441	1	0	0,62	
Hyalomma scupense	2.4 [0-9.9]	NA	1	42	NA	NA	1	
Hyalomma species	3.9 [0.4-9.7]	[0-36.2]	13	5742	5.8 [5-6.7]	97 [96-97.8]	< 0.001	
Hyalomma sulcata	0 [0-7.3]	NA	2	26	1	0	0,959	
Hyalomma truncatum	0 [0-6.1]	NA	1	28	NA	NA	1	
Hyalomma turanicum	5.1 [0.1-14.8]	NA	1	39	NA	NA	1	
Hyalomma dromedarii	0 [0-4.6]	NA	1	37	NA	NA	1	
Hyalomma marginatum	0 [0-16.5]	NA	1	10	NA	NA	1	
Ixodes daminii	0 [0-8]	NA	1	21	NA	NA	1	
Ixodes ricinus	0 [0-0.5]	[0-2.3]	6	1334	1.3 [1-2]	39.2 [0-75.9]	0,144	
Ixodes species	0.4 [0-2.3]	NA	2	168	1	0	0,742	
Ormithodoros savygni	0 [0-4.9]	NA	1	35	NA	NA	1	
Ornithodoros lahorensis	36 [18.1-56]	NA	1	25	NA	NA	1	
Rhipicephalus (Boophilus) annulatus	0 [0-4.3]	NA	1	40	NA	NA	1	
Rhipicephalus annulatus	9.2 [3.2-17.7]	NA	1	65	NA	NA	1	
Rhipicephalus appendiculatus	0 [0-1.1]	NA	2	114	1	0	0,63	
Rhipicephalus bursa	4.1 [0.6-9.8]	[0-33.3]	11	1114	3.4 [2.7-4.2]	91.1 [86.1-94.3]	< 0.001	

	Prevalence. % (95%CI)	95% Prediction interval	N Studies	N Participants	H (95%CI)	I² (95%CI)	P heterogeneity	P difference subtypes
Rhipicephalus pulchellus	0 [0-0.1]	NA	1	3243	NA	NA	1	
Rhipicephalus sanguineus	3.3 [1.1-6.5]	[0-19.3]	18	1190	2 [1.6-2.6]	76 [62.2-84.8]	< 0.001	
Rhipicephalus species	1.5 [0-7.7]	[0-30.4]	7	334	2 [1.4-2.9]	75.3 [47.8-88.3]	< 0.001	
Rhipicephalus turanicus	0 [0-1.4]	[0-52.3]	3	455	1.3 [1-2.5]	44.6 [0-83.5]	0,164	
Riphicephalus bursa	0 [0-9.3]	NA	1	18	NA	NA	1	
**CCHFV prevalence in other animal species**								
**Current infections**								
**Timing of data collection**								0,001
Prospectively	5.5 [3.2-8.3]	[0.9-12.7]	9	738	1.2 [1-1.8]	34.1 [0-69.6]	0,145	
Retrospectively	0 [0-2.4]	NA	1	72	NA	NA	1	
**Country**								0,001
India	0 [0-2.4]	NA	1	72	NA	NA	1	
Mauritania	27.3 [4.4-57.9]	NA	1	11	NA	NA	1	
South Africa	0 [0-16.5]	NA	1	10	NA	NA	1	
Turkey	5.8 [3.8-8.1]	[2.3-10.6]	7	717	1.1 [1-1.6]	18.1 [0-61.9]	0,292	
**Country income level**								0,89
Lower-middle income economies	6.5 [0-51.1]	NA	2	83	3.2 [1.7-6.1]	90.3 [64.9-97.3]	0,001	
Upper-middle-income economies	5.3 [3.5-7.5]	[2.4-9.1]	8	727	1.1 [1-1.9]	10.4 [0-71]	0,349	
**WHO Region**								0,004
Africa	9.5 [0-48.5]	NA	2	21	2 [1-4.1]	73.7 [0-94.1]	0,051	
Europe	5.8 [3.8-8.1]	[2.3-10.6]	7	717	1.1 [1-1.6]	18.1 [0-61.9]	0,292	
South-East Asia	0 [0-2.4]	NA	1	72	NA	NA	1	
**Order**								0,89
Artiodactyla	5.3 [3.5-7.5]	[2.4-9.1]	8	727	1.1 [1-1.9]	10.4 [0-71]	0,349	
Rodentia	6.5 [0-51.1]	NA	2	83	3.2 [1.7-6.1]	90.3 [64.9-97.3]	0,001	
**Population characteristics**								0,002
Cattle	3 [0.7-6.4]	NA	2	211	1	0	0,627	
Goat	4.3 [1.7-7.9]	[0-38.2]	3	221	1 [1-1.6]	0 [0-61.6]	0,763	
Mastomys erytholeucus	27.3 [4.4-57.9]	NA	1	11	NA	NA	1	
Rattus rattus	0 [0-2.4]	NA	1	72	NA	NA	1	
Sheep	8.2 [3.9-13.9]	[0-88.4]	3	295	1.4 [1-2.7]	52.3 [0-86.3]	0,123	
**Recent infections**								

	Prevalence. % (95%CI)	95% Prediction interval	N Studies	N Participants	H (95%CI)	I² (95%CI)	P heterogeneity	P difference subtypes
**Country**								0,748
Greece	0 [0-8.9]	NA	1	19	NA	NA	1	
Mauritania	1 [0-5.7]	[0-100]	3	1341	2.3 [1.3-4]	80.4 [38.5-93.8]	0,006	
Replublic of Kosovo	0.2 [0-2.1]	NA	2	267	1.3	44.4	0,18	
**Country income level**								0,748
High-income economies	0 [0-8.9]	NA	1	19	NA	NA	1	
Lower-middle income economies	1 [0-5.7]	[0-100]	3	1341	2.3 [1.3-4]	80.4 [38.5-93.8]	0,006	
Upper-middle-income economies	0.2 [0-2.1]	NA	2	267	1.3	44.4	0,18	
**WHO Region**								0,893
Africa	1 [0-5.7]	[0-100]	3	1341	2.3 [1.3-4]	80.4 [38.5-93.8]	0,006	
Europe	0 [0-8.9]	NA	1	19	NA	NA	1	
**Population characteristics**								0,289
Cattle	0 [0-1]	NA	1	172	NA	NA	1	
Goat	0 [0-4.1]	NA	2	46	1	0	0,905	
Sheep	1.5 [0-5.3]	[0-96.3]	3	1409	2.4 [1.3-4.1]	82 [44.4-94.2]	0,004	
**Past infections**								
**Sampling**								0,241
Non probabilistic	11.4 [9.1-14]	[0-56]	182	68558	9.5 [9.2-9.7]	98.9 [98.8-98.9]	< 0.001	
Probabilistic	15.2 [10.2-21]	[0-57.9]	36	23500	10.6 [10-11.2]	99.1 [99-99.2]	< 0.001	
**Timing of data collection**								0,026
Prospectively	12 [9.8-14.3]	[0-53.3]	198	82113	9.3 [9-9.5]	98.8 [98.8-98.9]	< 0.001	
Retrospectively	22.6 [13.7-33]	[0.1-64.4]	10	8749	8.8 [7.7-10]	98.7 [98.3-99]	< 0.001	
**Country**								< 0.001
Afghanistan	78.1 [70.6-84.9]	NA	2	132	1	0	0,56	
Albania	1.1 [0-8.4]	[0-100]	3	90	1.5 [1-2.8]	56.2 [0-87.5]	0,102	
Bhutan	12 [0-67.7]	NA	2	173	8.1 [5.5-12]	98.5 [96.7-99.3]	< 0.001	
Bulgaria	33.8 [17.6-52.1]	[0-93.3]	8	3888	10.4 [9.1-11.8]	99.1 [98.8-99.3]	< 0.001	
Central Africa Republic	40.4 [35.5-45.3]	NA	1	389	NA	NA	1	
China	26 [3.5-59.4]	NA	2	3294	7.5 [5-11.3]	98.2 [96-99.2]	< 0.001	
Democratic Republic of the Congo	1.2 [0-5.5]	[0-100]	3	838	3.2 [2-5.2]	90.3 [74.2-96.3]	< 0.001	
Egypt	1.1 [0-5.3]	[0-29.4]	11	6421	7.1 [6.2-8.2]	98 [97.4-98.5]	< 0.001	
Gabon	19 [15.8-22.4]	NA	1	532	NA	NA	1	
Germany	2.6 [0-7.7]	NA	1	77	NA	NA	1	
Ghana	0.6 [0-2.6]	NA	1	167	NA	NA	1	

	Prevalence. % (95%CI)	95% Prediction interval	N Studies	N Participants	H (95%CI)	I² (95%CI)	P heterogeneity	P difference subtypes
Greece	9.2 [0.6-24.4]	[0-100]	3	719	3.2 [2-5.3]	90.5 [74.8-96.4]	< 0.001	
Hungary	1.1 [0-3.2]	[0-11.9]	6	2452	2.9 [2.1-4]	88 [76.3-93.9]	< 0.001	
India	19.7 [13.4-26.7]	[0.7-53.2]	16	8120	6 [5.2-6.8]	97.2 [96.4-97.8]	< 0.001	
Iran	21.4 [14.8-28.8]	[0-62.6]	24	18065	10.3 [9.6-11.1]	99.1 [98.9-99.2]	< 0.001	
Iraq	34 [19.6-50.1]	[0-88.3]	7	2493	7.9 [6.7-9.3]	98.4 [97.7-98.9]	< 0.001	
Italy	0 [0-0.3]	NA	1	540	NA	NA	1	
Mali	65.7 [62.8-68.5]	NA	1	1075	NA	NA	1	
Mauritania	26.8 [8.1-51.1]	[0-99.5]	6	1904	8.6 [7.2-10.2]	98.6 [98.1-99]	< 0.001	
Niger	13.1 [1.5-33.6]	[0-95.2]	5	2467	11.6 [9.9-13.6]	99.3 [99-99.5]	< 0.001	
Nigeria	25.7 [23.2-28.2]	NA	1	1164	NA	NA	1	
Oman	18.1 [10.5-27]	[0-62.3]	4	489	2.2 [1.3-3.6]	79.2 [44.4-92.2]	0,002	
Pakistan	4.5 [0.6-11]	[0-35.1]	7	1918	4.3 [3.4-5.5]	94.6 [91.2-96.7]	< 0.001	
Panama	0 [0-4]	NA	1	43	NA	NA	1	
Poland	0 [0-0.3]	NA	1	592	NA	NA	1	
Portugal	0 [0-1.2]	NA	1	141	NA	NA	1	
Replublic of Kosovo	7.6 [1.8-16.3]	[0-46.5]	5	660	2.7 [1.9-4]	86.7 [71.2-93.9]	< 0.001	
Republic of the Congo	3.2 [1.5-5.4]	NA	1	316	NA	NA	1	
Romania	55.9 [17.4-90.7]	[0-100]	3	561	6.4 [4.6-8.8]	97.5 [95.2-98.7]	< 0.001	
Saudi Arabia	2.1 [0.7-4.1]	[0-12.7]	4	2799	1.8 [1.1-3.1]	69.2 [11-89.3]	0,021	
Senegal	3.9 [0-12]	[0-39.7]	7	1116	2.5 [1.8-3.5]	84.4 [69.6-92]	< 0.001	
South Africa	10.1 [6.8-13.7]	[0-35.1]	35	21241	6.3 [5.8-6.8]	97.5 [97-97.8]	< 0.001	
Sudan	15.2 [7.1-25.6]	[0-100]	3	942	4 [2.6-6.2]	93.9 [85.5-97.4]	< 0.001	
Turkey	52.6 [32-72.8]	[0-100]	6	1110	6.6 [5.3-8.1]	97.7 [96.5-98.5]	< 0.001	
United Arab Emirates	6.6 [3.1-11.2]	[0-26]	4	268	1.3 [1-2.2]	37.5 [0-78.5]	0,187	
Zimbabwe	45.5 [42-49]	NA	1	763	NA	NA	1	
**Country income level**								< 0.001
High-income economies	6.6 [3.5-10.5]	[0-35.8]	29	8681	6 [5.5-6.6]	97.2 [96.7-97.7]	< 0.001	
Low-income economies	21 [9.3-35.8]	[0-87.1]	15	5843	12.5 [11.5-13.6]	99.4 [99.2-99.5]	< 0.001	
Lower-middle income economies	10.2 [7-13.8]	[0-43.5]	53	22062	7.5 [7.1-8]	98.2 [98-98.4]	< 0.001	
Upper-middle-income economies	18.8 [15.6-22.2]	[0-55.1]	91	51373	8.8 [8.4-9.1]	98.7 [98.6-98.8]	< 0.001	
**WHO Region**								< 0.001
Africa	7.8 [5.3-10.7]	[0-44.5]	93	36071	8.2 [7.8-8.5]	98.5 [98.4-98.6]	< 0.001	
America	0 [0-4]	NA	1	43	NA	NA	1	
Eastern Mediterranean	13.9 [9.7-18.6]	[0-61.3]	66	33527	11.1 [10.7-11.6]	99.2 [99.1-99.3]	< 0.001	

	Prevalence. % (95%CI)	95% Prediction interval	N Studies	N Participants	H (95%CI)	I² (95%CI)	P heterogeneity	P difference subtypes
Europe	18.7 [11.2-27.6]	[0-77.5]	33	10170	10.3 [9.7-10.9]	99.1 [98.9-99.2]	< 0.001	
South-East Asia	18.7 [12.8-25.5]	[0.3-52.8]	18	8293	5.9 [5.3-6.7]	97.2 [96.4-97.8]	< 0.001	
Western Pacific	26 [3.5-59.4]	NA	2	3294	7.5 [5-11.3]	98.2 [96-99.2]	< 0.001	
**Recrutment setting**								0,06
Rural	9 [6.1-12.5]	[0-43.5]	60	18078	6.8 [6.4-7.2]	97.8 [97.5-98.1]	< 0.001	
Urban	4.4 [1.4-8.7]	[0-25.7]	10	3831	4.9 [4.1-5.9]	95.9 [94-97.2]	< 0.001	
**Order**								< 0.001
Anseriformes	0 [0-1.9]	NA	1	91	NA	NA	1	
Apodiformes	0 [0-11.2]	NA	1	15	NA	NA	1	
Artiodactyla	17.4 [14.5-20.5]	[0-60.5]	143	71013	10.1 [9.8-10.4]	99 [99-99.1]	< 0.001	
Bucerotiformes	27.3 [10.4-48.1]	NA	1	22	NA	NA	1	
Carnivora	1.5 [0-4.8]	[0-13.6]	8	2256	1.7 [1.2-2.5]	65.2 [25.9-83.7]	0,005	
Chiroptera	1.2 [0-3.6]	[0-60.3]	3	526	1.5 [1-2.8]	55.1 [0-87.2]	0,108	
Columbiformes	0 [0-2.2]	NA	2	67	1	0	0,674	
Eulipotyphla	0 [0-7.3]	NA	1	23	NA	NA	1	
Gruiformes	0 [0-12.8]	NA	1	13	NA	NA	1	
Hyracoidea	0 [0-8.9]	NA	1	19	NA	NA	1	
Lagomorpha	8.1 [3.4-14.4]	[0-33.2]	8	751	2.4 [1.8-3.3]	82.9 [67.7-91]	< 0.001	
Macroscelidea	0 [0-1]	NA	2	143	1	0	0,679	
Passeriformes	0 [0-1.3]	[0-6.5]	6	306	1.3 [1-2]	38 [0-75.3]	0,153	
Pelecaniformes	0 [0-11.9]	NA	1	14	NA	NA	1	
Perissodactyla	35.6 [10.6-65.7]	[0-100]	4	404	5.2 [3.8-7.1]	96.3 [93.2-98]	< 0.001	
Primates	0 [0-8]	NA	1	21	NA	NA	1	
Proboscidea	0 [0-1.2]	NA	2	234	1	0	0,856	
Pteroclifores	0 [0-11.9]	NA	1	14	NA	NA	1	
Rodentia	1.4 [0.4-2.9]	[0-9]	19	3349	2.1 [1.7-2.6]	77 [64.5-85.2]	< 0.001	
Struthioniformes	18.2 [8.8-29.9]	NA	2	184	1.9 [1-4]	72 [0-93.7]	0,059	
**Population characteristics**								< 0.001
Aepyceros melampus	1.4 [0.2-3.6]	NA	1	211	NA	NA	1	
Aethomys chrysophilus	9.8 [3.4-18.8]	NA	1	61	NA	NA	1	
Aethomys namaquensis	0 [0-4.9]	NA	1	35	NA	NA	1	
Apodemus agrarius	2.4 [0-9.9]	NA	1	42	NA	NA	1	
Apodemus flavicollis	1.3 [0.7-1.9]	NA	1	1439	NA	NA	1	
Apodemus sylvaticus	0 [0-1.1]	NA	1	156	NA	NA	1	

	**Prevalence. % (95%CI)**	**95% Prediction interval**	**N Studies**	**N Participants**	**H (95%CI)**	**I² (95%CI)**	**P heterogeneity**	**P difference subtypes**
Arvicanthis niloticus	16.3 [6.5-29]	NA	1	43	NA	NA	1	
Baboon	0 [0-8]	NA	1	21	NA	NA	1	
Bat	1.2 [0-3.6]	[0-60.3]	3	526	1.5 [1-2.8]	55.1 [0-87.2]	0,108	
Blue wildebeest	0 [0-5.5]	NA	1	31	NA	NA	1	
Bovine	5.7 [0-21.5]	[0-100]	3	1496	9 [6.9-11.7]	98.8 [97.9-99.3]	< 0.001	
Bubalornis albirostris	0 [0-3.2]	NA	1	54	NA	NA	1	
Buffalo	4 [0-12.4]	[0-50.5]	5	920	4.4 [3.3-5.9]	94.8 [90.6-97.1]	< 0.001	
Camel	11.9 [8.4-15.8]	[2.6-25.8]	9	5520	2.4 [1.8-3.3]	83.3 [69.8-90.8]	< 0.001	
Canis familiaris	6 [5-7.1]	NA	1	1978	NA	NA	1	
Cattle	19.7 [14.5-25.6]	[0-58.8]	33	30792	11.5 [10.8-12.1]	99.2 [99.1-99.3]	< 0.001	
Cattle egret	0 [0-4.4]	NA	1	39	NA	NA	1	
Cheetah	0 [0-11.9]	NA	1	14	NA	NA	1	
Cow	3.8 [0-16.7]	[0-64.3]	6	630	4 [3-5.3]	93.6 [88.7-96.4]	< 0.001	
Crocidura species	0 [0-7.3]	NA	1	23	NA	NA	1	
Dasymys incomtus	1.1 [0-4.5]	NA	1	95	NA	NA	1	
Desmodillus auricularis	0 [0-2.9]	NA	1	58	NA	NA	1	
Elephant	0 [0-7.3]	NA	1	23	NA	NA	1	
Elephantulus species	0 [0-1.5]	NA	1	112	NA	NA	1	
Equus burchelli	17.2 [10.1-25.6]	NA	1	93	NA	NA	1	
Felis caracal	0 [0-9.9]	NA	1	17	NA	NA	1	
Genetta genetta	0 [0-16.5]	NA	1	10	NA	NA	1	
Giraffe	22.7 [11.4-36.4]	NA	1	44	NA	NA	1	
Goat	22.4 [15.3-30.3]	[0-70.7]	30	6259	6.8 [6.2-7.4]	97.8 [97.4-98.2]	< 0.001	
Hare	5.1 [0-29]	NA	2	273	4.8 [2.8-8.1]	95.6 [87.2-98.5]	< 0.001	
Hippopotamus	0 [0-11.2]	NA	1	15	NA	NA	1	
Horse	58.7 [52.6-64.7]	NA	1	252	NA	NA	1	
Impala	10.6 [3.1-21.3]	NA	1	47	NA	NA	1	
Lamprotornis species	18.2 [0.5-47.4]	NA	1	11	NA	NA	1	
Laughing dove	0 [0-11.9]	NA	1	14	NA	NA	1	
Lepus capensis	22.6 [12.9-33.9]	NA	1	62	NA	NA	1	
Lepus saxatilis	14.5 [8.9-21.1]	NA	1	131	NA	NA	1	
Lepus species	14.3 [5.7-25.7]	NA	1	49	NA	NA	1	
Lion	0 [0-1.5]	NA	1	116	NA	NA	1	
Little swift	0 [0-11.2]	NA	1	15	NA	NA	1	

	Prevalence. % (95%CI)	95% Prediction interval	N Studies	N Participants	H (95%CI)	I² (95%CI)	P heterogeneity	P difference subtypes
Livestock	26 [4-58.4]	NA	2	7459	29.2 [24.7-34.4]	99.9 [99.8-99.9]	< 0.001	
Loxodonta africana	0.5 [0-2]	NA	1	211	NA	NA	1	
Lupus europeus	8.6 [5-12.9]	NA	1	198	NA	NA	1	
Macroscelides proboscideus	0 [0-5.5]	NA	1	31	NA	NA	1	
Masked weaver	0 [0-10.5]	NA	1	16	NA	NA	1	
Mastomys coucha	0 [0-15.1]	NA	1	11	NA	NA	1	
Mastomys natalensis	0 [0-4.5]	NA	1	38	NA	NA	1	
Moorhen	0 [0-12.8]	NA	1	13	NA	NA	1	
Myodes glareolus	0.2 [0-1]	NA	1	448	NA	NA	1	
Ostriches	18.2 [8.8-29.9]	NA	2	184	1.9 [1-4]	72 [0-93.7]	0,059	
Otocyon megalotis	0 [0-16.5]	NA	1	10	NA	NA	1	
Otomys angoniensis	0 [0-4.7]	NA	1	36	NA	NA	1	
Otomys unisulcatus	0 [0-3.3]	NA	1	52	NA	NA	1	
Passer luteus	0 [0-8.4]	NA	1	20	NA	NA	1	
Pedetes capensis	12.1 [2.8-25.8]	NA	1	33	NA	NA	1	
Phacochoerus aethiopicus	5 [0.1-14.5]	NA	1	40	NA	NA	1	
Pig	0 [0-6.8]	NA	1	25	NA	NA	1	
Procavia capensis	0 [0-8.9]	NA	1	19	NA	NA	1	
Pronolagus crassicaudatus	0 [0-12.8]	NA	1	13	NA	NA	1	
Pronolagus rupestris	0 [0-6.8]	NA	1	25	NA	NA	1	
Pterocles exustus	0 [0-11.9]	NA	1	14	NA	NA	1	
Rattus rattus	0 [0-4.3]	NA	1	40	NA	NA	1	
Red bishop	0 [0-1.6]	NA	1	110	NA	NA	1	
Redbilled quelea	0 [0-1.8]	NA	1	95	NA	NA	1	
Rhabdomys pumilio	0.6 [0-1.7]	NA	1	344	NA	NA	1	
Rodents	9.6 [5.4-14.7]	NA	1	157	NA	NA	1	
Sable	6.1 [0.8-14.9]	NA	1	49	NA	NA	1	
Sacred ibis	0 [0-11.9]	NA	1	14	NA	NA	1	
Sheep	20.5 [13.5-28.5]	[0-78.7]	42	15789	11.4 [10.8-12]	99.2 [99.1-99.3]	< 0.001	
Small ruminants	10.3 [7.5-13.4]	NA	1	418	NA	NA	1	
Streptopelia species	0 [0-3.2]	NA	1	53	NA	NA	1	
Tatera leucogaster	2.2 [0.6-4.7]	NA	1	224	NA	NA	1	
Tockus erythrorhynchus	27.3 [10.4-48.1]	NA	1	22	NA	NA	1	
Warthog	0 [0-8]	NA	1	21	NA	NA	1	

	Prevalence. % (95%CI)	95% Prediction interval	N Studies	N Participants	H (95%CI)	I² (95%CI)	P heterogeneity	P difference subtypes
White rhino	67.7 [50.1-83.2]	NA	1	31	NA	NA	1	
Wild dog	4.8 [0.6-11.9]	NA	1	62	NA	NA	1	
Xerus inauris	2.7 [0-11.2]	NA	1	37	NA	NA	1	
Yellowbilled duck	0 [0-1.9]	NA	1	91	NA	NA	1	
Zebra	7.1 [0.1-20.3]	NA	1	28	NA	NA	1	

CCHFV: Crimean-congo hemorrhagic fever virus; CI: confidence interval; NA: not applicable; WHO: World Health Organization
