# Supplementary material for: Worldwide epidemiology of Crimean-Congo Hemorrhagic Fever Virus in humans, ticks and other animal species, a systematic review and meta-analysis
Source: PLoS Negl Trop Dis. 2021 Apr 22;15(4):e0009299. doi: 10.1371/journal.pntd.0009299 (PMC8096040; doi:10.1371/journal.pntd.0009299)
Supplement: S9 Table — (PDF) [file pntd.0009299.s013.pdf]

S9 Table. Univariable and multivariable meta‐regression analysis on the human case fatality rate and prevalence of CCHFV in humans, ticks, and other animal species.

Variables		Bivariate Model				Multivariate Model		
	Estimate	P-Value	P-Value Global	OR(95% CI)	Estimate	P-Value	OR [95% CI]	R2
**Case fatality rate in humans**								
**Ongoing infections**								**78,02%**
**Study Design**			0,404					
Case-series	1							
Case control	0,121	0,457		1,13 [0,82 - 1,55]				
Community outbreak	0,275	0,247		1,32 [0,83 - 2,1]				
Cross sectional	0,027	0,84		1,03 [0,79 - 1,34]				
Hospital outbreak	0,178	0,314		1,19 [0,84 - 1,69]				
**Sampling**			**0,058**					
Non probabilistic	1				[redacted]			
Probabilistic	0,286	**0,058**		1,33 [0,99 - 1,79]				
**Timing of data collection**			0,25					
Prospectively	1							
Retrospectively	0,051	0,25		1,05 [0,96 - 1,15]				
**Country income level**			**0**					
Low-income economies	1				1		1	
Lower-middle income economies	-0,122	**0,162**		0,89 [0,75 - 1,05]	-0,2466	**0,0028**	0,78 [0,66 - 0,92]	
Upper-middle-income economies	-0,311	**0**		0,73 [0,65 - 0,83]	-0,2289	<,0001	0,8 [0,71 - 0,89]	
**WHO Region**			**0**					
Africa	1				1		1	
Eastern Mediterranean	-0,157	0,212		0,85 [0,67 - 1,09]	-0,2282	**0,039**	0,8 [0,64 - 0,99]	
Europe	-0,352	**0,004**		0,7 [0,55 - 0,89]	-0,3564	**0,0008**	0,7 [0,57 - 0,86]	
South-East Asia	-0,024	0,871		0,98 [0,73 - 1,31]	-0,0065	0,9661	0,99 [0,73 - 1,34]	
**Age range (years)**			**0**					
Adults: 19+ years	1				[redacted]			
Child: Birth-18 years	-0,237	**0**		0,79 [0,69 - 0,9]				
**Recrutment setting**			0,263					
Rural	1							
Urban	0,106	0,263		1,11 [0,92 - 1,34]				
**Hospitalization**			**0,019**					
Ambulatory	1				[redacted]			
Hospitalized	-0,299	**0,019**		0,74 [0,58 - 0,95]				
**Risk of bias**			0,331					
Low risk of bias	1							
Moderate risk of bias	-0,042	0,331		0,96 [0,88 - 1,04]				
**Mean or Median age Years**	0,0006	0,7903		1 [1 - 1]				
**Gender**	0,0064	**0,0043**		1,01 [1 - 1,01]				
**Recent infections**								
**Timing of data collection**			**0,001**					
Prospectively	1				[redacted]			
Retrospectively	0,388	**0,001**		1,47 [1,18 - 1,83]				
**WHO Region**			**0**					
Eastern Mediterranean	1				1		1	
Europe	-0,386	**0**		0,68 [0,57 - 0,82]	-0,386	**0**	0,68 [0,57 - 0,82]	
**Risk of bias**			**0,116**					
Low risk of bias	1				[redacted]			
Moderate risk of bias	-0,248	**0,116**		0,78 [0,57 - 1,06]				
**CCHFV prevalence in humans**								
**Ongoing infections**								**43,94%**
**Study Design**			0,313					
Community outbreak	1							
Cross sectional	0,097	0,565		1,1 [0,79 - 1,53]				
Hospital outbreak	0,396	**0,138**		1,49 [0,88 - 2,51]				
**Sampling**			**0,063**					
Non probabilistic	1				[redacted]			
Probabilistic	-0,47	**0,063**		0,63 [0,38 - 1,03]				
**Timing of data collection**			**0,027**					
Prospectively	1				[redacted]			
Retrospectively	0,202	**0,027**		1,22 [1,02 - 1,47]				
**Country income level**			**0,001**					
High-income economies	1				[redacted]			
Low-income economies	-0,093	0,613		0,91 [0,64 - 1,3]				
Lower-middle income economies	-0,221	**0,173**		0,8 [0,58 - 1,1]				
Upper-middle-income economies	0,099	0,527		1,1 [0,81 - 1,5]				
**WHO Region**			**0**					
Africa	1				1		1	
Eastern Mediterranean	0,345	**0**		1,41 [1,17 - 1,7]	0,345	**0**	1,41 [1,17 - 1,7]	
Europe	0,55	**0**		1,73 [1,44 - 2,08]	0,55	**0**	1,73 [1,44 - 2,08]	
South-East Asia	0,315	**0,043**		1,37 [1,01 - 1,86]	0,315	**0,043**	1,37 [1,01 - 1,86]	
**Age range (years)**			0,655					
Adults: 19+ years	1							
Child: Birth-18 years	-0,108	0,655		0,9 [0,56 - 1,44]				
**Recrutment setting**			0,685					
Rural	1							

Urban	0,1	0,685		1,11 [0,68 - 1,79]				
**Hospitalization**			**0,173**					
Ambulatory	1				[redacted]			
Hospitalized	0,308	**0,173**		1,36 [0,87 - 2,12]	[redacted]			
**Risk of bias**			0,687					
Low risk of bias	1							
Moderate risk of bias	0,034	0,687		1,03 [0,88 - 1,22]				
**Mean or Median age Years**	0,0102	**0,0789**		1,01 [1 - 1,02]				
**Gender**	-0,01	**0,0038**		0,99 [0,98 - 1]				
**Recent infections**								**35,61%**
**Study Design**			**0,012**					
Cohort (Baseline data)	1				1		1	
Community outbreak	0,179	0,472		1,2 [0,73 - 1,95]	0,3482	**0,1037**	1,42 [0,93 - 2,15]	
Cross sectional	0,31	**0,175**		1,36 [0,87 - 2,13]	0,4238	**0,0242**	1,53 [1,06 - 2,21]	
Hospital outbreak	0,801	**0,005**		2,23 [1,27 - 3,92]	0,9399	**0,0001**	2,56 [1,58 - 4,15]	
**Sampling**			**0,046**					
Non probabilistic	1				1		1	
Probabilistic	-0,242	**0,046**		0,79 [0,62 - 1]	-0,2153	**0,0234**	0,81 [0,67 - 0,97]	
**Timing of data collection**			**0,017**					
Prospectively	1				1		1	
Retrospectively	0,173	**0,017**		1,19 [1,03 - 1,37]	0,1543	**0,0256**	1,17 [1,02 - 1,34]	
**Country income level**			**0,007**					
High-income economies	1				[redacted]			
Low-income economies	-0,317	**0,058**		0,73 [0,53 - 1,01]	[redacted]			
Lower-middle income economies	-0,395	**0,012**		0,67 [0,5 - 0,92]	[redacted]			
Upper-middle-income economies	-0,183	0,229		0,83 [0,62 - 1,12]	[redacted]			
**WHO Region**			**0**					
Africa	1				1		1	
Eastern Mediterranean	0,297	**0**		1,35 [1,16 - 1,56]	0,2155	**0,0029**	1,24 [1,08 - 1,43]	
Europe	0,347	**0**		1,41 [1,22 - 1,64]	0,3539	<,0001	1,42 [1,24 - 1,63]	
South-East Asia	0,175	0,214		1,19 [0,9 - 1,57]	0,1426	0,316	1,15 [0,87 - 1,52]	
**Age range (years)**			**0,013**					
Adults: 19+ years	1				[redacted]			
Child: Birth-18 years	0,428	**0,013**		1,53 [1,1 - 2,14]	[redacted]			
**Recrutment setting**			0,422					
Rural	1							
Urban	0,074	0,422		1,08 [0,9 - 1,29]				
**Hospitalization**			**0**					
Ambulatory	1				[redacted]			
Hospitalized	0,617	**0**		1,85 [1,61 - 2,14]	[redacted]			
**Risk of bias**			**0,049**		[redacted]			
Low risk of bias	1							
Moderate risk of bias	-0,138	**0,049**		0,87 [0,76 - 1]				
**Mean or Median age Years**	-0,0023	0,5405		1 [0,99 - 1,01]				
**Gender**	0,0045	0,2399		1 [1 - 1,01]				
**Past infections**								**16,41%**
**Study Design**			0,526					
Case control	1							
Cohort (Baseline data)	-0,019	0,926		0,98 [0,66 - 1,45]				
Community outbreak	-0,158	0,318		0,85 [0,63 - 1,16]				
Cross sectional	-0,165	0,239		0,85 [0,64 - 1,12]				
Hospital outbreak	-0,098	0,534		0,91 [0,67 - 1,23]				
**Sampling**			0,734					
Non probabilistic	1							
Probabilistic	-0,009	0,734		0,99 [0,94 - 1,04]				
**Timing of data collection**			**0,002**					
Prospectively	1				1		1	
Retrospectively	0,16	**0,002**		1,17 [1,06 - 1,3]	0,1373	**0,0051**	1,15 [1,04 - 1,26]	
**Country income level**			**0,008**					
High-income economies	1				[redacted]			
Low-income economies	-0,081	**0,039**		0,92 [0,85 - 1]	[redacted]			
Lower-middle income economies	-0,017	0,636		0,98 [0,91 - 1,06]	[redacted]			
Upper-middle-income economies	0,033	0,31		1,03 [0,97 - 1,1]	[redacted]			
**WHO Region**			**0**					
Africa	1						1	
Eastern Mediterranean	0,092	**0,001**		1,1 [1,04 - 1,16]	0,0767	**0,0097**	1,08 [1,02 - 1,14]	
Europe	0,107	**0**		1,11 [1,05 - 1,18]	0,101	**0,0009**	1,11 [1,04 - 1,17]	
South-East Asia	-0,105	**0,18**		0,9 [0,77 - 1,05]	-0,1086	**0,1658**	0,9 [0,77 - 1,05]	
Western Pacific	-0,047	0,453		0,95 [0,84 - 1,08]	-0,0505	0,4216	0,95 [0,84 - 1,08]	
**Age range (years)**			0,252					
Adults: 19+ years	1							
Child: Birth-18 years	-0,119	0,252		0,89 [0,72 - 1,09]				
**Recrutment setting**			0,458					
Rural	1							
Urban	-0,028	0,458		0,97 [0,9 - 1,05]				
**Hospitalization**			**0,001**					
Ambulatory	1				[redacted]			
Hospitalized	0,219	**0,001**		1,24 [1,09 - 1,42]	[redacted]			

**Risk of bias**			0,776					
Low risk of bias	1							
Moderate risk of bias	0,008	0,776		1,01 [0,96 - 1,06]				
**Mean or Median age Years**	0,0002	0,9054		1 [1 - 1]				
**Gender**	0,0004	0,6221		1 [1 - 1]				
**CCHFV prevalence in ticks**								
**Ongoing infections**								**8,87%**
**Sampling**			**0,01**					
Non probabilistic	1				[redacted]			
Probabilistic	0,062	**0,01**		1,06 [1,02 - 1,12]				
**Timing of data collection**			0,498					
Prospectively	1							
Retrospectively	-0,089	0,498		0,91 [0,71 - 1,18]				
**Country income level**			**0,095**					
High-income economies	1				[redacted]			
Low-income economies	-0,018	0,853		0,98 [0,81 - 1,19]				
Lower-middle income economies	0,072	**0,062**		1,07 [1 - 1,16]				
Upper-middle-income economies	0,067	**0,021**		1,07 [1,01 - 1,13]				
**WHO Region**			**0**					
Africa	1				1		1	
Eastern Mediterranean	0,033	0,412		1,03 [0,96 - 1,12]	0,033	0,412	1,03 [0,96 - 1,12]	
Europe	-0,085	**0,028**		0,92 [0,85 - 0,99]	-0,085	**0,028**	0,92 [0,85 - 0,99]	
South-East Asia	-0,085	0,406		0,92 [0,75 - 1,12]	-0,085	0,406	0,92 [0,75 - 1,12]	
Western Pacific	-0,18	**0,056**		0,84 [0,69 - 1]	-0,18	**0,056**	0,84 [0,69 - 1]	
**Recrutment setting**			0,98					
Rural	1							
Urban	0,002	0,98		1 [0,85 - 1,18]				
**Gender (Tick)**			**0,071**					
Amblyomma	1				[redacted]			
Argas	-0,127	0,359		0,88 [0,67 - 1,16]				
Dermacentor	-0,138	**0,07**		0,87 [0,75 - 1,01]				
Haemaphysalis	-0,095	0,226		0,91 [0,78 - 1,06]				
Hyalomma	-0,031	0,634		0,97 [0,85 - 1,1]				
Ixodes	-0,143	**0,077**		0,87 [0,74 - 1,02]				
Ornithodoros	0,126	0,35		1,13 [0,87 - 1,48]				
Rhipicephalus	-0,046	0,49		0,96 [0,84 - 1,09]				
**CCHFV prevalence in other animal species**								
**Current infection_Other animals**								**66,86%**
**Timing of data collection**			**0,006**					
Prospectively	1				[redacted]			
Retrospectively	-0,211	**0,006**		0,81 [0,7 - 0,94]				
**Country income level**			0,304					
Lower-middle income economies	1							
Upper-middle-income economies	0,086	0,304		1,09 [0,93 - 1,28]				
**WHO Region**			**0,014**					
Africa	1				1		1	
Europe	-0,106	0,349		0,9 [0,72 - 1,12]	-0,106	0,349	0,9 [0,72 - 1,12]	
South-East Asia	-0,311	**0,018**		0,73 [0,57 - 0,95]	-0,311	**0,018**	0,73 [0,57 - 0,95]	
**Mean or Median age Years**								
**Gender**								
**Recent infections**								[redacted]
**Country income level**			0,807					
High-income economies	1							
Lower-middle income economies	0,021	0,888		1,02 [0,76 - 1,37]				
Upper-middle-income economies	-0,035	0,82		0,97 [0,72 - 1,3]				
**WHO Region**			0,913					
Africa	1							
Europe	-0,018	0,913		0,98 [0,72 - 1,35]				
**Past infections**								**0,00%**
**Sampling**			0,27					
Non probabilistic	1							
Probabilistic	0,05	0,27		1,05 [0,96 - 1,15]				
**Timing of data collection**			**0,082**					
Prospectively	1				[redacted]			
Retrospectively	0,131	**0,082**		1,14 [0,98 - 1,32]				
**Country income level**			**0**					
High-income economies	1				[redacted]			
Low-income economies	0,203	**0,002**		1,23 [1,08 - 1,39]				
Lower-middle income economies	0,064	**0,181**		1,07 [0,97 - 1,17]				
Upper-middle-income economies	0,184	**0**		1,2 [1,1 - 1,31]				
**WHO Region**			**0,007**					
Africa	1				1		1	
America	-0,224	0,374		0,8 [0,49 - 1,31]	-0,1698	0,4383	0,84 [0,55 - 1,3]	
Eastern Mediterranean	0,09	**0,024**		1,09 [1,01 - 1,18]	0,5384	<,0001	1,71 [1,34 - 2,2]	
Europe	0,151	**0,002**		1,16 [1,05 - 1,28]	0,174	**0,0164**	1,19 [1,03 - 1,37]	
South-East Asia	0,157	**0,013**		1,17 [1,03 - 1,33]	0,3076	**0,0124**	1,36 [1,07 - 1,73]	
Western Pacific	0,237	**0,17**		1,27 [0,9 - 1,78]	0,1189	0,5633	1,13 [0,75 - 1,69]	

**Recrutment setting**			**0,142**					
Rural	1						1	
Urban	-0,098	**0,142**		0,91 [0,79 - 1,03]	-0,5626	<,0001	0,57 [0,43 - 0,75]	
**Risk of bias**			0,643					
Low risk of bias	1							
Moderate risk of bias	0,078	0,643		1,08 [0,78 - 1,5]				
