# Supplementary material for: Worldwide epidemiology of Crimean-Congo Hemorrhagic Fever Virus in humans, ticks and other animal species, a systematic review and meta-analysis
Source: PLoS Negl Trop Dis. 2021 Apr 22;15(4):e0009299. doi: 10.1371/journal.pntd.0009299 (PMC8096040; doi:10.1371/journal.pntd.0009299)

S1 Fig: Global case fatality rate estimate of Crimean-Congo hemorrhagic fever virus ongoing, recent and past infections in humans

Study or Subgroup	Events	Total	Weight	Events [95% CI]
**CFR−Ongoing infection (45 Studies)**				
Ahmeti, 2019_Kosovo	11	32	1.7%	34.38 [18.57; 53.19]
Bakir, 2012_Turkey	13	237	2.4%	5.49 [2.95; 9.20]
Bakir, 2015_Turkey	25	404	2.5%	6.19 [4.04; 9.00]
Bakir, 2016_Turkey	6	72	2.1%	8.33 [3.12; 17.26]
Batool, 2009_Iran	19	123	2.3%	15.45 [9.56; 23.07]
Belet, 2014_Turkey	1	47	1.9%	2.13 [0.05; 11.29]
Bilgin, 2014_Turkey	9	128	2.3%	7.03 [3.27; 12.93]
Bozkurt, 2016_Turkey	8	35	1.7%	22.86 [10.42; 40.14]
Çelik, 2016_Turkey	4	33	1.7%	12.12 [3.40; 28.20]
Cevik, 2008_Turkey	11	69	2.1%	15.94 [8.24; 26.74]
Chinikar, 2010_Iran	89	635	2.5%	14.02 [11.41; 16.96]
Dilber, 2009_Turkey	1	19	1.4%	5.26 [0.13; 26.03]
Dokuzoguz, 2013_Turkey	23	281	2.4%	8.19 [5.26; 12.03]
Duygu, 2012_Turkey	20	400	2.5%	5.00 [3.08; 7.62]
Duygu, 2018_Turkey	11	173	2.3%	6.36 [3.22; 11.09]
Eren, 2016_Turkey	11	149	2.3%	7.38 [3.74; 12.83]
Erenler, 2014_Turkey	38	560	2.5%	6.79 [4.85; 9.20]
Erenler, 2015_Turkey	21	97	2.2%	21.65 [13.93; 31.17]
Ergonul, 2006_Turkey	3	19	1.4%	15.79 [3.38; 39.58]
Ertugrul, 2009_Turkey	1	13	1.1%	7.69 [0.19; 36.03]
Gambhir, 2019_India	4	12	1.1%	33.33 [9.92; 65.11]
Gonzalan, 2007_Turkey	1	29	1.6%	3.45 [0.09; 17.76]
Gul, 2015_Turkey	8	48	1.9%	16.67 [7.48; 30.22]
Hatipoglu, 2010_Turkey	5	93	2.2%	5.38 [1.77; 12.10]
Izadi, 2009_Iran	16	63	2.0%	25.40 [15.27; 37.94]
Kadanali, 2012_Turkey	2	13	1.1%	15.38 [1.92; 45.45]
Karakus, 2019_Turkey	13	132	2.3%	9.85 [5.35; 16.25]
Kizilgun, 2013_Turkey	0	41	1.8%	0.00 [0.00; 8.60]
Leblebicioglu, 2016_Turkey	4	25	1.5%	16.00 [4.54; 36.08]
Mofleh, 2012_Afghanistan	10	30	1.7%	33.33 [17.29; 52.81]
Mostafavi, 2014_Iran	126	871	2.5%	14.47 [12.20; 16.98]
Mourya, 2017_India	7	21	1.4%	33.33 [14.59; 56.97]
Niazi, 2019_Afghanistan	14	58	2.0%	24.14 [13.87; 37.17]
No author listed, 2006_Turkey	11	150	2.3%	7.33 [3.72; 12.74]
Ozbey, 2014_Turkey	10	342	2.4%	2.92 [1.41; 5.31]
Sahak, 2019_Afghanistan	127	293	2.4%	43.34 [37.59; 49.23]
Sheikh, 2005_Pakistan	8	83	2.1%	9.64 [4.25; 18.11]
Swanepoel, 1987_South Africa	11	31	1.7%	35.48 [19.23; 54.63]
Tasdelen Fisgin, 2009_Turkey	6	50	1.9%	12.00 [4.53; 24.31]
Tasdelen Fisgin, 2010_Turkey	13	140	2.3%	9.29 [5.04; 15.36]
Tuygun, 2012_Turkey	0	50	1.9%	0.00 [0.00; 7.11]
Yadav, 2014_India	3	10	1.0%	30.00 [6.67; 65.25]
Yilmaz, 2009_Turkey	92	1820	2.5%	5.05 [4.09; 6.16]
Yilmaz, 2015_Turkey	19	150	2.3%	12.67 [7.80; 19.07]
Ziauddin, 2018_Pakistan	5	15	1.2%	33.33 [11.82; 61.62]
**Random effect meta−analysis**		**8096**	**88.4%**	**11.70 [9.08; 14.57]**
Heterogeneity: $Tau^2 = 0.0148$; $Chi^2 = 483.02$, df = 44 (P < 0.0001); $I^2 = 90.9\%$ [88.7%; 92.7%]				
**CFR−Recent infection (6 Studies)**				
Belet, 2014_Turkey	1	46	1.9%	2.17 [0.06; 11.53]
Bokaie, 2008_Iran	2	10	1.0%	20.00 [2.52; 55.61]
Ergonul, 2006_Turkey	1	47	1.9%	2.13 [0.05; 11.29]
Fakoorziba, 2006_Iran	3	13	1.1%	23.08 [5.04; 53.81]
Kadanali, 2012_Turkey	1	52	1.9%	1.92 [0.05; 10.26]
Majeed, 2012_Iraq	4	11	1.0%	36.36 [10.93; 69.21]
**Random effect meta−analysis**		**179**	**8.9%**	**8.01 [1.02; 18.97]**
Heterogeneity: $Tau^2 = 0.0227$; $Chi^2 = 17.72$, df = 5 (P = 0.0033); $I^2 = 71.8\%$ [34.7%; 87.8%]				
**CFR−Past infection (2 Studies)**				
Bokaie, 2008_Iran	2	10	1.0%	20.00 [2.52; 55.61]
Ergonul, 2006_Turkey	0	31	1.7%	0.00 [0.00; 11.22]
**Random effect meta−analysis**		**41**	**2.7%**	**4.74 [0.00; 37.68]**
Heterogeneity: $Tau^2 = 0.0666$; $Chi^2 = 5.2$, df = 1 (P = 0.0226); $I^2 = 80.8\%$ [17.7%; 95.5%]				

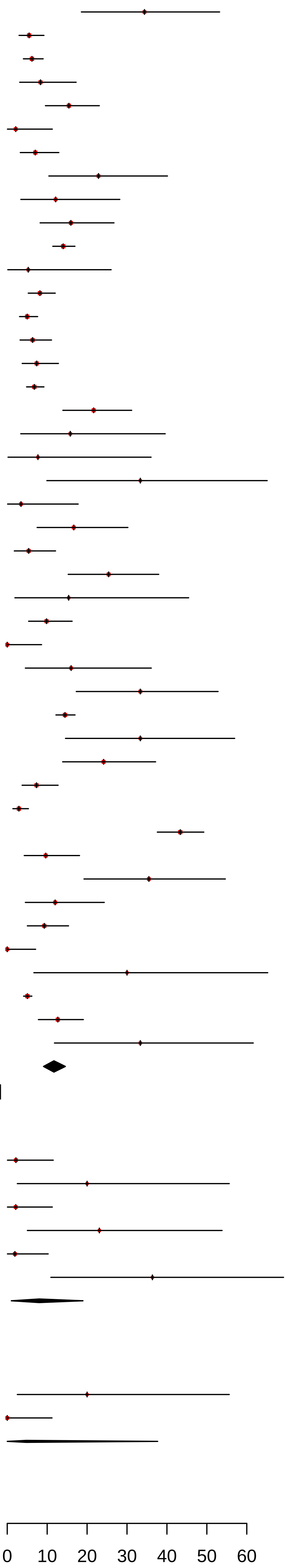

Supplement: S1 Fig — (PDF) [file pntd.0009299.s014.pdf]
