# Supplementary material for: Worldwide epidemiology of Crimean-Congo Hemorrhagic Fever Virus in humans, ticks and other animal species, a systematic review and meta-analysis
Source: PLoS Negl Trop Dis. 2021 Apr 22;15(4):e0009299. doi: 10.1371/journal.pntd.0009299 (PMC8096040; doi:10.1371/journal.pntd.0009299)

S2 Fig: Global prevalence estimate of Crimean-Congo hemorrhagic fever virus ongoing, recent and past infections in humans

Study or Subgroup	Events	Total	Weight	Events [95% CI]
**Humans−Ongoing infection (67 Studies)**				
Alam, 2017_Pakistan	302	1339	0.4%	22.55 [20.34; 24.89]
Almasri, 2019_Saudi Arabia	0	80	0.4%	0.00 [0.00; 4.51]
Aslani, 2017_Iran	157	908	0.4%	17.29 [14.89; 19.91]
Bakir, 2012_Turkey	275	387	0.4%	71.06 [66.26; 75.53]
Baumann, 2019_Mali	2	489	0.4%	0.41 [0.05; 1.47]
Belet, 2014_Turkey	47	167	0.4%	28.14 [21.47; 35.61]
Bob, 2017_Mauritania	1	184	0.4%	0.54 [0.01; 2.99]
Bokaie, 2008_Iran	5	63	0.4%	7.94 [2.63; 17.56]
Bonney, 2013_Ghana	0	285	0.4%	0.00 [0.00; 1.29]
Bower, 2019_Sudan	11	65	0.4%	16.92 [8.76; 28.27]
Bukbuk, 2016_Nigeria	1	380	0.4%	0.26 [0.01; 1.46]
Chinikar, 2007_Iran	80	763	0.4%	10.48 [8.40; 12.88]
Chinikar, 2010_Iran	635	1638	0.4%	38.77 [36.40; 41.18]
Dilber, 2009_Turkey	19	186	0.4%	10.22 [6.26; 15.49]
Erenler, 2014_Turkey	560	970	0.4%	57.73 [54.55; 60.86]
Erenler, 2015_Turkey	97	240	0.4%	40.42 [34.15; 46.92]
Ergönül, 2004_Turkey	9	44	0.4%	20.45 [9.80; 35.30]
Ertugrul, 2009_Turkey	14	26	0.3%	53.85 [33.37; 73.41]
Gadia, 2017_Central Africa Republic	0	198	0.4%	0.00 [0.00; 1.85]
Gandhi, 2011_India	3	225	0.4%	1.33 [0.28; 3.85]
Gonzalan, 2007_Turkey	9	36	0.3%	25.00 [12.12; 42.20]
Gozdas, 2019_Turkey	29	76	0.4%	38.16 [27.25; 50.02]
Hasan, 2014_Pakistan	52	354	0.4%	14.69 [11.17; 18.81]
Hatipoglu, 2010_Turkey	68	121	0.4%	56.20 [46.89; 65.20]
Hekimoglu, 2014_Turkey	16	41	0.4%	39.02 [24.20; 55.50]
Kadanali, 2012_Turkey	13	63	0.4%	20.63 [11.47; 32.70]
Kara, 2016_Turkey	9	21	0.3%	42.86 [21.82; 65.98]
Karakecili, 2018_Turkey	197	206	0.4%	95.63 [91.87; 97.98]
Karlberg, 2015_Iran	11	17	0.3%	64.71 [38.33; 85.79]
Khan, 1997_United Arab Emirates	9	16	0.3%	56.25 [29.88; 80.25]
Khurshid, 2015_Afghanistan	4	18	0.3%	22.22 [6.41; 47.64]
Khurshid, 2015_Pakistan	14	82	0.4%	17.07 [9.66; 26.98]
Kilinc, 2016_Turkey	102	281	0.4%	36.30 [30.67; 42.22]
Leblebicioglu, 2016_Turkey	25	51	0.4%	49.02 [34.75; 63.40]
Malik, 2011_Pakistan	8	88	0.4%	9.09 [4.01; 17.13]
Midilli, 2007_Turkey	10	91	0.4%	10.99 [5.40; 19.28]
Mostafavi, 2014_Iran	871	2536	0.4%	34.35 [32.50; 36.23]
Mourya, 2017_India	21	69	0.4%	30.43 [19.92; 42.69]
Nabeth, 2004_Mauritania	1	84	0.4%	1.19 [0.03; 6.46]
Nabeth, 2004_Mauritania	33	59	0.4%	55.93 [42.40; 68.84]
Nadeem, 2003_Pakistan	8	34	0.3%	23.53 [10.75; 41.17]
Niazi, 2019_Afghanistan	58	63	0.4%	92.06 [82.44; 97.37]
No author listed, 2006_Turkey	150	323	0.4%	46.44 [40.90; 52.04]
Papa, 2008_Albania	11	34	0.3%	32.35 [17.39; 50.53]
Rodriguez, 1997_United Arab Emirates	7	16	0.3%	43.75 [19.75; 70.12]
Sahak, 2019_Afghanistan	293	891	0.4%	32.88 [29.80; 36.08]
Schwarz, 1996_Middle East	4	17	0.3%	23.53 [6.81; 49.90]
Schwarz, 1996_United Arab Emirates	4	16	0.3%	25.00 [7.27; 52.38]
Sharifi−Mood, 2008_Iran	9	34	0.3%	26.47 [12.88; 44.36]
Sharifi−Mood, 2009_Iran	19	123	0.4%	15.45 [9.56; 23.07]
Sheikh, 2005_Pakistan	9	135	0.4%	6.67 [3.09; 12.28]
Sow, 2016_Senegal	0	13845	0.4%	0.00 [0.00; 0.03]
Sunbul, 2015_Turkey	313	4406	0.4%	7.10 [6.36; 7.90]
Swanepoel, 1985_South Africa	1	318	0.4%	0.31 [0.01; 1.74]
Swanepoel, 1985_South Africa	1	130	0.4%	0.77 [0.02; 4.21]
Tanyel, 2016_Turkey	304	420	0.4%	72.38 [67.84; 76.61]
Thomas, 2012_Kosovo	14	56	0.4%	25.00 [14.39; 38.37]
Tumturk, 2019_Turkey	151	399	0.4%	37.84 [33.07; 42.80]
Tuygun, 2012_Turkey	40	53	0.4%	75.47 [61.72; 86.24]
Wasfi, 2016_Tunisia	0	181	0.4%	0.00 [0.00; 2.02]
Yadav, 2014_India	0	26	0.3%	0.00 [0.00; 13.23]
Yadav, 2014_India	10	12	0.3%	83.33 [51.59; 97.91]
Yaqub, 2017_Pakistan	0	480	0.4%	0.00 [0.00; 0.77]
Yashina, 2003_Kazakhstan	2	16	0.3%	12.50 [1.55; 38.35]
Yashina, 2003_Russia	5	15	0.3%	33.33 [11.82; 61.62]
Zhang, 2019_Sierra Leone	1	96	0.4%	1.04 [0.03; 5.67]
Ziauddin, 2018_Pakistan	15	112	0.4%	13.39 [7.69; 21.13]
**Random effect meta−analysis**		**35198**	**24.4%**	**22.54 [15.78; 30.08]**
Heterogeneity: $Tau^2 = 0.1184$; $Chi^2 = 13691.24$, df = 66 (P = 0); $I^2 = 99.5\%$ [99.5%; 99.5%]				
**Humans−Recent infection (56 Studies)**				
Ahmed, 2019_Sudan	3	204	0.4%	1.47 [0.30; 4.24]
Akuffo, 2016_Ghana	1	108	0.4%	0.93 [0.02; 5.05]
Alam, 2013_Pakistan	16	44	0.4%	36.36 [22.41; 52.23]
Andriamandimby, 2011_Madagascar	1	1995	0.4%	0.05 [0.00; 0.28]
Aslani, 2017_Iran	100	908	0.4%	11.01 [9.05; 13.23]
Belet, 2014_Turkey	46	167	0.4%	27.54 [20.93; 34.98]
Bob, 2017_Mauritania	5	184	0.4%	2.72 [0.89; 6.23]
Bokaie, 2008_Iran	10	63	0.4%	15.87 [7.88; 27.26]
Bower, 2019_Sudan	6	65	0.4%	9.23 [3.46; 19.02]
Bukbuk, 2016_Nigeria	49	1189	0.4%	4.12 [3.06; 5.41]
Chapman, 1991_Senegal	2	283	0.4%	0.71 [0.09; 2.53]
Chinikar, 2007_Iran	309	763	0.4%	40.50 [36.99; 44.08]
Christova, 2013_Bulgaria	13	527	0.4%	2.47 [1.32; 4.18]
Cikman, 2016_Turkey	7	372	0.4%	1.88 [0.76; 3.84]
Ergönül, 2004_Turkey	32	44	0.4%	72.73 [57.21; 85.04]
Ergonul, 2007_Turkey	0	75	0.4%	0.00 [0.00; 4.80]
Fakoorziba, 2006_Iran	16	45	0.4%	35.56 [21.87; 51.22]
Gargili, 2011_Turkey	3	193	0.4%	1.55 [0.32; 4.48]
Gonzalez, 1990_Mauritania	1	99	0.4%	1.01 [0.03; 5.50]
Gozalan, 2007_Turkey	25	36	0.3%	69.44 [51.89; 83.65]
Gozdas, 2019_Turkey	2	76	0.4%	2.63 [0.32; 9.18]
Gozel, 2013_Turkey	0	190	0.4%	0.00 [0.00; 1.92]
Gozel, 2014_Turkey	0	132	0.4%	0.00 [0.00; 2.76]
Greiner, 2016_Georgia	1	435	0.4%	0.23 [0.01; 1.27]
Hatami, 2019_Afghanistan	22	108	0.4%	20.37 [13.23; 29.20]
Hatipoglu, 2010_Turkey	67	121	0.4%	55.37 [46.06; 64.41]
Head, 2020_Kazakhstan	3	914	0.4%	0.33 [0.07; 0.96]
Holakouie, 2004_Iran	8	285	0.4%	2.81 [1.22; 5.46]
Izadi, 2008_Iran	0	57	0.4%	0.00 [0.00; 6.27]
Kadanali, 2012_Turkey	52	63	0.4%	82.54 [70.90; 90.95]
Kalvatchev, 2010_Bulgaria	1	16	0.3%	6.25 [0.16; 30.23]
Karlberg, 2015_Iran	5	17	0.3%	29.41 [10.31; 55.96]
Khan, 1997_United Arab Emirates	10	16	0.3%	62.50 [35.43; 84.80]
Khurshid, 2015_Afghanistan	1	18	0.3%	5.56 [0.14; 27.29]
Khurshid, 2015_Pakistan	9	82	0.4%	10.98 [5.14; 19.82]
Kuchuloria, 2014_Georgia	3	14	0.3%	21.43 [4.66; 50.80]
Lwande, 2012_Kenya	1	517	0.4%	0.19 [0.00; 1.07]
Majeed, 2012_Iraq	11	39	0.3%	28.21 [15.00; 44.87]
Mardani, 2007_Iran	0	223	0.4%	0.00 [0.00; 1.64]
Midilli, 2007_Turkey	9	91	0.4%	9.89 [4.62; 17.95]
Midilli, 2009_Turkey	39	741	0.4%	5.26 [3.77; 7.12]
Mourya, 2012_India	4	86	0.4%	4.65 [1.28; 11.48]
Niazi, 2019_Afghanistan	32	63	0.4%	50.79 [37.89; 63.62]
Nur, 1999_Democratic Republic of the Congo	0	45	0.4%	0.00 [0.00; 7.87]
Ozkurt, 2006_Turkey	26	36	0.3%	72.22 [54.81; 85.80]
Rodriguez, 1997_United Arab Emirates	8	16	0.3%	50.00 [24.65; 75.35]
Safronetz, 2016_Mali	18	376	0.4%	4.79 [2.86; 7.46]
Schwarz, 1996_United Arab Emirates	0	16	0.3%	0.00 [0.00; 20.59]
Sheikh, 2005_Pakistan	22	135	0.4%	16.30 [10.50; 23.63]
Sow, 2016_Senegal	0	13845	0.4%	0.00 [0.00; 0.03]
Tigoi, 2015_Kenya	0	379	0.4%	0.00 [0.00; 0.97]
Tumturk, 2019_Turkey	248	399	0.4%	62.16 [57.20; 66.93]
Tuygun, 2012_Turkey	28	40	0.4%	70.00 [53.47; 83.44]
Wasfi, 2016_Tunisia	5	181	0.4%	2.76 [0.90; 6.33]
Yadav, 2014_India	0	26	0.3%	0.00 [0.00; 13.23]
Yadav, 2014_India	3	11	0.3%	27.27 [6.02; 60.97]
**Random effect meta−analysis**		**27173**	**20.4%**	**11.66 [7.59; 16.41]**
Heterogeneity: $Tau^2 = 0.0597$; $Chi^2 = 4779.96$, df = 55 (P = 0); $I^2 = 98.8\%$ [98.7%; 99.0%]				
**Humans−Past infection (147 Studies)**				
Abdiyeva, 2019_Kazakhstan	102	802	0.4%	12.72 [10.49; 15.22]
Akuffo, 2016_Ghana	4	108	0.4%	3.70 [1.02; 9.21]
Al−Nakib, 1984_Kuwait	17	501	0.4%	3.39 [1.99; 5.38]
Al Adhamy, 1992_Iraq	54	441	0.4%	12.24 [9.33; 15.67]
Altaf, 1998_Pakistan	3	40	0.4%	7.50 [1.57; 20.39]
Altaf, 1998_Pakistan	0	10	0.3%	0.00 [0.00; 30.85]
Andriamandimby, 2011_Madagascar	15	1995	0.4%	0.75 [0.42; 1.24]
Antoniadis, 1982_Greece	4	65	0.4%	6.15 [1.70; 15.01]
Athar, 2005_Pakistan	2	190	0.4%	1.05 [0.13; 3.75]
Bayram, 2017_Turkey	53	368	0.4%	14.40 [10.98; 18.41]
Blackburn, 1982_Zimbabwe	18	486	0.4%	3.70 [2.21; 5.79]
Bodur, 2012_Turkey	356	3557	0.4%	10.01 [9.04; 11.04]
Bokaie, 2008_Iran	10	63	0.4%	15.87 [7.88; 27.26]
Botros, 1989_Somalia	0	28	0.3%	0.00 [0.00; 12.34]
Bower, 2019_Sudan	11	65	0.4%	16.92 [8.76; 28.27]
Bryan, 1996_Pakistan	0	212	0.4%	0.00 [0.00; 1.72]
Bukbuk, 2014_Nigeria	7	297	0.4%	2.36 [0.95; 4.80]
Bukbuk, 2016_Nigeria	133	1189	0.4%	11.19 [9.45; 13.12]
Chantal, 1996_Djibouti	0	108	0.4%	0.00 [0.00; 3.36]
Chapman, 1991_Senegal	37	283	0.4%	13.07 [9.38; 17.57]
Chinikar, 2007_Iran	245	763	0.4%	32.11 [28.81; 35.55]
Chinikar, 2008_Iran	397	1010	0.4%	39.31 [36.28; 42.40]
Chinikar, 2012_Iran	12	100	0.4%	12.00 [6.36; 20.02]
Chinikar, 2012_Iran	16	108	0.4%	14.81 [8.71; 22.94]
Christova, 2013_Bulgaria	28	1018	0.4%	2.75 [1.84; 3.95]
Christova, 2013_Bulgaria	20	527	0.4%	3.80 [2.33; 5.80]
Christova, 2017_Bulgaria	55	1500	0.4%	3.67 [2.77; 4.75]
Cikman, 2016_Turkey	52	372	0.4%	13.98 [10.62; 17.92]
Clements, 2019_Uganda	100	1675	0.4%	5.97 [4.88; 7.21]
Darwish, 1983_Pakistan	1	43	0.4%	2.33 [0.06; 12.29]
David−West, 1974_Nigeria	26	250	0.4%	10.40 [6.91; 14.87]
el−Azazy, 1997_Saudi Arabia	16	122	0.4%	13.11 [7.69; 20.42]
el−Azazy, 1997_Saudi Arabia	16	28	0.3%	57.14 [37.18; 75.54]
Enkhtsetseg, 2016_South Sudan	0	632	0.4%	0.00 [0.00; 0.58]
Ergönül, 2004_Turkey	27	44	0.4%	61.36 [45.50; 75.64]
Ergönül, 2006_Turkey	1	83	0.4%	1.20 [0.03; 6.53]
Ergonul, 2007_Turkey	0	75	0.4%	0.00 [0.00; 4.80]
Ertugrul, 2012_Turkey	84	429	0.4%	19.58 [15.93; 23.66]
Fajs, 2014_Replublic of Kosovo	44	1105	0.4%	3.98 [2.91; 5.31]
Filipe, 1985_Portugal	2	258	0.4%	0.78 [0.09; 2.77]
Fisher−Hoch, 1992_South Africa	1	128	0.4%	0.78 [0.02; 4.28]
Fisher−Hoch, 1992_South Africa	6	484	0.4%	1.24 [0.46; 2.68]
Gargili, 2011_Turkey	21	193	0.4%	10.88 [6.86; 16.15]
Gazi, 2016_Turkey	12	324	0.4%	3.70 [1.93; 6.38]
Gergova, 2014_Bulgaria	24	751	0.4%	3.20 [2.06; 4.72]
Gonzalez, 1989_Cameroon	2	1152	0.4%	0.17 [0.02; 0.63]
Gonzalez, 1989_Central Africa Republic	0	327	0.4%	0.00 [0.00; 1.12]
Gonzalez, 1989_Chad	0	334	0.4%	0.00 [0.00; 1.10]
Gonzalez, 1989_Democratic Republic of the Congo	4	728	0.4%	0.55 [0.15; 1.40]
Gonzalez, 1989_Equatorial Guinea	5	688	0.4%	0.73 [0.24; 1.69]
Gonzalez, 1989_Gabon	0	841	0.4%	0.00 [0.00; 0.44]
Gonzalez, 1990_Mauritania	36	99	0.4%	36.36 [26.93; 46.64]
Gozel, 2013_Turkey	1	190	0.4%	0.53 [0.01; 2.90]
Gozel, 2014_Turkey	16	132	0.4%	12.12 [7.09; 18.94]
Greiner, 2016_Georgia	12	435	0.4%	2.76 [1.43; 4.77]
Gunes, 2009_Turkey	2	100	0.4%	2.00 [0.24; 7.04]
Gunes, 2009_Turkey	100	782	0.4%	12.79 [10.53; 15.33]
Hassanein, 1997_Saudi Arabia	3	354	0.4%	0.85 [0.18; 2.46]
Hassaniazad, 2016_Iran	0	44	0.4%	0.00 [0.00; 8.04]
Hatami, 2019_Afghanistan	7	108	0.4%	6.48 [2.65; 12.90]
Head, 2020_Kazakhstan	12	914	0.4%	1.31 [0.68; 2.28]
Holakouie, 2004_Iran	7	285	0.4%	2.46 [0.99; 4.99]
Horváth, 1976_Hungary	17	587	0.4%	2.90 [1.70; 4.60]
Hosseini−Vasoukolaei, 2016_Iran	0	40	0.4%	0.00 [0.00; 8.81]
Izadi, 2006_Iran	7	297	0.4%	2.36 [0.95; 4.80]
Izadi, 2008_Iran	1	57	0.4%	1.75 [0.04; 9.39]
Johnson, 1983_Kenya	4	1822	0.4%	0.22 [0.06; 0.56]
Johnson, 1983_Kenya	2	741	0.4%	0.27 [0.03; 0.97]
Johnson, 1993_Central Africa Republic	4	4295	0.4%	0.09 [0.03; 0.24]
Kalvatchev, 2010_Bulgaria	4	16	0.3%	25.00 [7.27; 52.38]
Kara, 2016_Turkey	0	21	0.3%	0.00 [0.00; 16.11]
Karlberg, 2015_Iran	0	17	0.3%	0.00 [0.00; 19.51]
Khan, 1997_United Arab Emirates	0	80	0.4%	0.00 [0.00; 4.51]
Khan, 1997_United Arab Emirates	0	200	0.4%	0.00 [0.00; 1.83]
Khan, 1997_United Arab Emirates	0	80	0.4%	0.00 [0.00; 4.51]
Khan, 1997_United Arab Emirates	5	77	0.4%	6.49 [2.14; 14.51]
Khan, 1997_United Arab Emirates	7	214	0.4%	3.27 [1.33; 6.62]
Khan, 1997_United Arab Emirates	5	16	0.3%	31.25 [11.02; 58.66]
Kilinc, 2016_Turkey	102	281	0.4%	36.30 [30.67; 42.22]
Koksal, 2014_Turkey	85	625	0.4%	13.60 [11.01; 16.54]
Lani, 2015_Malaysia	1	682	0.4%	0.15 [0.00; 0.81]
Lepers, 1988_Mauritania	1	965	0.4%	0.10 [0.00; 0.58]
Lwande, 2012_Kenya	96	517	0.4%	18.57 [15.31; 22.19]
Magnaval, 2011_Russia	10	90	0.4%	11.11 [5.46; 19.49]
Maltezou, 2009_Greece	0	21	0.3%	0.00 [0.00; 16.11]
Mardani, 2007_Iran	5	223	0.4%	2.24 [0.73; 5.15]
Mathiot, 1989_Madagascar	2	381	0.4%	0.52 [0.06; 1.88]
McCarthy, 1996_Sudan	5	296	0.4%	1.69 [0.55; 3.90]
Memish, 2011_Saudi Arabia	6	1024	0.4%	0.59 [0.22; 1.27]
Midilli, 2009_Turkey	35	741	0.4%	4.72 [3.31; 6.51]
Mohd Shukri, 2015_Malaysia	0	85	0.4%	0.00 [0.00; 4.25]
Mostafavi, 2017_Iran	31	190	0.4%	16.32 [11.36; 22.35]
Mourya, 2019_India	0	307	0.4%	0.00 [0.00; 1.19]
Mourya, 2019_India	23	2705	0.4%	0.85 [0.54; 1.27]
Mourya, 2019_India	2	1966	0.4%	0.10 [0.01; 0.37]
Muianga, 2017_Mozambique	8	300	0.4%	2.67 [1.16; 5.19]
Mustafa, 2011_Afghanistan	37	330	0.4%	11.21 [8.02; 15.12]
Nadeem, 2003_Pakistan	8	34	0.3%	23.53 [10.75; 41.17]
Newman, 2014_Afghanistan	0	467	0.4%	0.00 [0.00; 0.79]
Nur, 1999_Democratic Republic of the Congo	0	45	0.4%	0.00 [0.00; 7.87]
O'Hearn, 2016_Sierra Leone	13	641	0.4%	2.03 [1.08; 3.44]
Ozkurt, 2006_Turkey	15	36	0.3%	41.67 [25.51; 59.24]
Palomar, 2017_Spain	0	228	0.4%	0.00 [0.00; 1.60]
Papa, 2008_Albania	11	34	0.3%	32.35 [17.39; 50.53]
Papa, 2013_Greece	24	166	0.4%	14.46 [9.49; 20.74]
Papa, 2016_Greece	119	3152	0.4%	3.78 [3.14; 4.50]
Rahden, 2019_Sudan	12	464	0.4%	2.59 [1.34; 4.47]
Rodhain, 1989_Uganda	3	132	0.4%	2.27 [0.47; 6.50]
Rodriguez, 1997_United Arab Emirates	5	16	0.3%	31.25 [11.02; 58.66]
Sadeuh−Mba, 2018_Cameroon	6	137	0.4%	4.38 [1.62; 9.29]
Safronetz, 2016_Mali	11	376	0.4%	2.93 [1.47; 5.17]
Saidi, 1975_Iran	46	351	0.4%	13.11 [9.76; 17.09]
Saluzzo, 1985_Mauritania	1	59	0.4%	1.69 [0.04; 9.09]
Sargianou, 2013_Greece	7	207	0.4%	3.38 [1.37; 6.84]
Schoepp, 2014_Sierra Leone	0	220	0.4%	0.00 [0.00; 1.66]
Schwarz, 1995_Oman	8	119	0.4%	6.72 [2.95; 12.82]
Schwarz, 1996_Middle East	0	17	0.3%	0.00 [0.00; 19.51]
Schwarz, 1996_United Arab Emirates	0	16	0.3%	0.00 [0.00; 20.59]
Shahbazi, 2019_Iran	1	250	0.4%	0.40 [0.01; 2.21]
Shahhosseini, 2018_Iran	39	136	0.4%	28.68 [21.25; 37.05]
Sheikh, 2005_Pakistan	52	135	0.4%	38.52 [30.28; 47.28]
Sidira, 2012_Greece	68	1611	0.4%	4.22 [3.29; 5.32]
Sidira, 2013_Greece	6	277	0.4%	2.17 [0.80; 4.65]
Sidira, 2013_Greece	1	51	0.4%	1.96 [0.05; 10.45]
Sun, 2009_China	43	2454	0.4%	1.75 [1.27; 2.35]
Swanepoel, 1983_South Africa	5	74	0.4%	6.76 [2.23; 15.07]
Swanepoel, 1985_South Africa	1	318	0.4%	0.31 [0.01; 1.74]
Swanepoel, 1985_South Africa	17	1370	0.4%	1.24 [0.72; 1.98]
Swanepoel, 1985_South Africa	0	130	0.4%	0.00 [0.00; 2.80]
Tarantola, 2007_Greece	6	277	0.4%	2.17 [0.80; 4.65]
Tarantola, 2007_Greece	0	19	0.3%	0.00 [0.00; 17.65]
Tarantola, 2007_Greece	1	32	0.3%	3.12 [0.08; 16.22]
Telmadarraiy, 2008_Iran	0	21	0.3%	0.00 [0.00; 16.11]
Temocin, 2018_Turkey	2	112	0.4%	1.79 [0.22; 6.30]
Tigoi, 2015_Kenya	97	379	0.4%	25.59 [21.27; 30.30]
Todd, 2016_Afghanistan	33	809	0.4%	4.08 [2.82; 5.68]
Tomori, 1988_Nigeria	1	1677	0.4%	0.06 [0.00; 0.33]
Vawda, 2018_South Africa	2	387	0.4%	0.52 [0.06; 1.85]
Voorhees, 2018_Mongolia	27	1926	0.4%	1.40 [0.93; 2.03]
Wasfi, 2016_Tunisia	0	181	0.4%	0.00 [0.00; 2.02]
Wasfi, 2016_Tunisia	2	38	0.3%	5.26 [0.64; 17.75]
Watts, 1994_Sudan	10	185	0.4%	5.41 [2.62; 9.72]
Williams, 2000_Oman	74	282	0.4%	26.24 [21.20; 31.79]
Wilson, 1990_Senegal	61	1017	0.4%	6.00 [4.62; 7.64]
Xia, 2011_China	60	1657	0.4%	3.62 [2.77; 4.64]
Yagci−Caglayik, 2014_Turkey	25	1066	0.4%	2.35 [1.52; 3.44]
Yaqub, 2017_Pakistan	0	480	0.4%	0.00 [0.00; 0.77]
**Random effect meta−analysis**		**74900**	**55.2%**	**4.31 [3.31; 5.42]**
Heterogeneity: $Tau^2 = 0.0209$; $Chi^2 = 6300.8$, df = 146 (P = 0); $I^2 = 97.7\%$ [97.5%; 97.9%]				

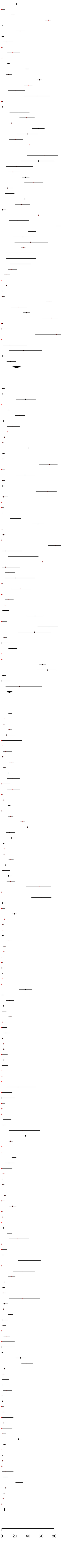

Supplement: S2 Fig — (PDF) [file pntd.0009299.s015.pdf]
