# Supplementary figures and images for: Worldwide epidemiology of Crimean-Congo Hemorrhagic Fever Virus in humans, ticks and other animal species, a systematic review and meta-analysis
Source: PLoS Negl Trop Dis. 2021 Apr 22;15(4):e0009299. doi: 10.1371/journal.pntd.0009299 (PMC8096040; doi:10.1371/journal.pntd.0009299)

S3 Fig. Global prevalence of Crimean-congo hemorrhagic fever virus infections in ticks

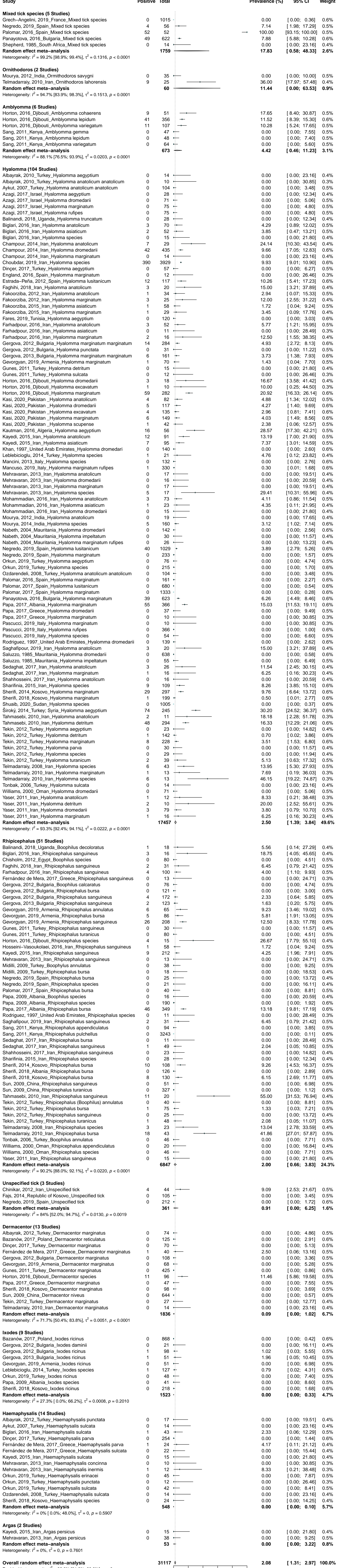

Supplement: S3 Fig — (PDF) [file pntd.0009299.s016.pdf]
