# Supplementary material for: Worldwide epidemiology of Crimean-Congo Hemorrhagic Fever Virus in humans, ticks and other animal species, a systematic review and meta-analysis
Source: PLoS Negl Trop Dis. 2021 Apr 22;15(4):e0009299. doi: 10.1371/journal.pntd.0009299 (PMC8096040; doi:10.1371/journal.pntd.0009299)

S4 Fig: Global prevalence estimate of Crimean-Congo hemorrhagic fever virus ongoing, recent and past infections in other animals species

Study or Subgroup	Events	Total	Weight	Events [95% CI]
**Other animal species−Ongoing infection (10 Studies)**				
Albayrak, 2012_Turkey_Goat	3	63	0.4%	4.76 [0.99; 13.29]
Albayrak, 2012_Turkey_Sheep	6	42	0.4%	14.29 [5.43; 28.54]
Mahzounieh, 2012_Turkey_Goat	0	11	0.3%	0.00 [0.00; 28.49]
Mahzounieh, 2012_Turkey_Sheep	4	93	0.4%	4.30 [1.18; 10.65]
Mourya, 2012_India_Rattus rattus	0	72	0.4%	0.00 [0.00; 4.99]
Saluzzo, 1985_Mauritania_Mastomys erytholeucus	3	11	0.3%	27.27 [6.02; 60.97]
Swanepoel, 1983_South Africa_Cattle	0	10	0.3%	0.00 [0.00; 30.85]
Tuncer, 2014_Turkey_Cattle	10	201	0.5%	4.98 [2.41; 8.96]
Tuncer, 2014_Turkey_Goat	9	147	0.4%	6.12 [2.84; 11.30]
Tuncer, 2014_Turkey_Sheep	15	160	0.4%	9.38 [5.34; 14.99]
**Random effect meta−analysis**		**810**	**4.1%**	**4.55 [1.94; 7.93]**
Heterogeneity: Tau^2 = 0.0053; Chi^2 = 23.43, df = 9 (P = 0.0053); I^2 = 61.6% [23.5%; 80.7%]				
**Other animal species−Recent infection (6 Studies)**				
Gonzalez, 1990_Mauritania_Sheep	53	1219	0.5%	4.35 [3.27; 5.65]
Nabeth, 2004_Mauritania_Goat	0	27	0.4%	0.00 [0.00; 12.77]
Nabeth, 2004_Mauritania_Sheep	0	95	0.4%	0.00 [0.00; 3.81]
Papa, 2018_Greece_Goat	0	19	0.4%	0.00 [0.00; 17.65]
Sherifi, 2016_Kosovo_Cattle	0	172	0.4%	0.00 [0.00; 2.12]
Sherifi, 2016_Kosovo_Sheep	1	95	0.4%	1.05 [0.03; 5.73]
**Random effect meta−analysis**		**1627**	**2.6%**	**0.43 [0.00; 2.92]**
Heterogeneity: Tau^2 = 0.0080; Chi^2 = 27.13, df = 5 (P < 0.0001); I^2 = 81.6% [60.6%; 91.4%]				
**Other animal species−Past infection (218 Studies)**				
Adam, 2013_Sudan_Cattle	21	299	0.5%	7.02 [4.40; 10.54]
Al Adhamy, 1992_Iraq_Animal unspecified	39	365	0.5%	10.68 [7.71; 14.32]
Albayrak, 2012_Turkey_Goat	42	63	0.4%	66.67 [53.66; 78.05]
Albayrak, 2012_Turkey_Sheep	36	42	0.4%	85.71 [71.46; 94.57]
Barthel, 2014_Bulgaria_Cattle	90	127	0.4%	70.87 [62.15; 78.59]
Barthel, 2014_Bulgaria_Goat	9	15	0.4%	60.00 [32.29; 83.66]
Barthel, 2014_Bulgaria_Sheep	179	242	0.5%	73.97 [67.96; 79.38]
Bazanów, 2017_Poland_Bovine	0	592	0.5%	0.00 [0.00; 0.62]
Behrooz, 2006_Iran_Sheep	10	270	0.5%	3.70 [1.79; 6.71]
Bokaie, 2008_Iran_Goat	69	150	0.4%	46.00 [37.84; 54.32]
Bokaie, 2008_Iran_Sheep	231	298	0.5%	77.52 [72.35; 82.13]
Burt, 1993_South Africa_Baboon	0	21	0.4%	0.00 [0.00; 16.11]
Burt, 1993_South Africa_Blue wildebeest	0	31	0.4%	0.00 [0.00; 11.22]
Burt, 1993_South Africa_Buffalo	31	312	0.5%	9.94 [6.85; 13.81]
Burt, 1993_South Africa_Cheetah	0	14	0.4%	0.00 [0.00; 23.16]
Burt, 1993_South Africa_Elephant	0	23	0.4%	0.00 [0.00; 14.82]
Burt, 1993_South Africa_Giraffe	10	44	0.4%	22.73 [11.47; 37.84]
Burt, 1993_South Africa_Hare	0	63	0.4%	0.00 [0.00; 5.69]
Burt, 1993_South Africa_Hippopotamus	0	15	0.4%	0.00 [0.00; 21.80]
Burt, 1993_South Africa_Impala	5	47	0.4%	10.64 [3.55; 23.10]
Burt, 1993_South Africa_Lion	0	116	0.4%	0.00 [0.00; 3.13]
Burt, 1993_South Africa_Sable	3	49	0.4%	6.12 [1.28; 16.87]
Burt, 1993_South Africa_Warthog	0	21	0.4%	0.00 [0.00; 16.11]
Burt, 1993_South Africa_White rhino	21	31	0.4%	67.74 [48.63; 83.32]
Burt, 1993_South Africa_Wild dog	3	62	0.4%	4.84 [1.01; 13.50]
Burt, 1993_South Africa_Zebra	2	28	0.4%	7.14 [0.88; 23.50]
Burt, 1996_South Africa_Cattle	905	2116	0.5%	42.77 [40.65; 44.91]
Ceianu, 2012_Romania_Sheep	131	471	0.5%	27.81 [23.81; 32.10]
Champour, 2014_Iran_Camel	9	170	0.4%	5.29 [2.45; 9.81]
Chinikar, 2008_Iran_Livestock	1803	4284	0.5%	42.09 [40.60; 43.58]
Chinikar, 2010_Iran_Animal unspecified	2014	4846	0.5%	41.56 [40.17; 42.96]
Chinikar, 2012_Iran_Animal unspecified	41	322	0.5%	12.73 [9.29; 16.87]
Christova, 2018_Bulgaria_Cattle	165	843	0.5%	19.57 [16.95; 22.42]
Christova, 2018_Bulgaria_Goat	20	88	0.4%	22.73 [14.47; 32.89]
Christova, 2018_Bulgaria_Sheep	10	130	0.4%	7.69 [3.75; 13.69]
Darwish, 1983_Pakistan_Buffalo	1	22	0.4%	4.55 [0.12; 22.84]
Darwish, 1983_Pakistan_Cow	1	45	0.4%	2.22 [0.06; 11.77]
Darwish, 1983_Pakistan_Goat	0	48	0.4%	0.00 [0.00; 7.40]
Darwish, 1983_Pakistan_Rodents	15	157	0.4%	9.55 [5.45; 15.27]
Darwish, 1983_Pakistan_Sheep	0	46	0.4%	0.00 [0.00; 7.71]
De Liberato, 2018_Italy_Sheep	0	540	0.5%	0.00 [0.00; 0.68]
Faghihi, 2018_Iran_Cow	0	11	0.3%	0.00 [0.00; 28.49]
Faghihi, 2018_Iran_Goat	5	26	0.4%	19.23 [6.55; 39.35]
Faghihi, 2018_Iran_Sheep	16	99	0.4%	16.16 [9.53; 24.91]
Fajs, 2014_Replublic of Kosovo_Cow	55	353	0.5%	15.58 [11.96; 19.79]
Fajs, 2014_Replublic of Kosovo_Goat	2	10	0.3%	20.00 [2.52; 55.61]
Fajs, 2014_Replublic of Kosovo_Sheep	3	30	0.4%	10.00 [2.11; 26.53]
Filipe, 1985_Portugal_Goat	0	141	0.4%	0.00 [0.00; 2.58]
Fisher−Hoch, 1992_South Africa_Cattle	158	420	0.5%	37.62 [32.97; 42.45]
Fisher−Hoch, 1992_South Africa_Goat	46	103	0.4%	44.66 [34.86; 54.78]
Fisher−Hoch, 1992_South Africa_Sheep	258	1689	0.5%	15.28 [13.59; 17.08]
Földes, 2019_Hungary_Apodemus agrarius	1	42	0.4%	2.38 [0.06; 12.57]
Földes, 2019_Hungary_Apodemus flavicollis	18	1439	0.5%	1.25 [0.74; 1.97]
Földes, 2019_Hungary_Apodemus sylvaticus	0	156	0.4%	0.00 [0.00; 2.34]
Földes, 2019_Hungary_Myodes glareolus	1	448	0.5%	0.22 [0.01; 1.24]
Gergova, 2013_Bulgaria_Cattle	140	1775	0.5%	7.89 [6.68; 9.24]
Gonzalez, 1990_Mauritania_Sheep	246	1219	0.5%	20.18 [17.96; 22.55]
Guilherme, 1996_Central Africa Republic_Cattle	157	389	0.5%	40.36 [35.45; 45.42]
Hassanein, 1997_Saudi Arabia_Camel	0	23	0.4%	0.00 [0.00; 14.82]
Hassanein, 1997_Saudi Arabia_Cattle	1	182	0.4%	0.55 [0.01; 3.02]
Hassanein, 1997_Saudi Arabia_Goat	14	432	0.5%	3.24 [1.78; 5.38]
Hassanein, 1997_Saudi Arabia_Sheep	88	2162	0.5%	4.07 [3.28; 4.99]
Horton, 2014_Egypt_Buffalo	0	153	0.4%	0.00 [0.00; 2.38]
Horton, 2014_Egypt_Camel	0	10	0.3%	0.00 [0.00; 30.85]
Horton, 2014_Egypt_Cattle	1	161	0.4%	0.62 [0.02; 3.41]
Horton, 2014_Egypt_Sheep	0	174	0.4%	0.00 [0.00; 2.10]
Horváth, 1976_Hungary_Animal unspecified	0	169	0.4%	0.00 [0.00; 2.16]
Hosseini−Vasoukolaei, 2016_Iran_Sheep	4	84	0.4%	4.76 [1.31; 11.75]
Ibrahim, 2015_Sudan_Cattle	54	282	0.5%	19.15 [14.73; 24.23]
Kasi, 2020_Pakistan_Goat	37	800	0.5%	4.62 [3.28; 6.32]
Kasi, 2020_Pakistan_Sheep	149	800	0.5%	18.62 [15.98; 21.50]
Khan, 1997_United Arab Emirates_Camel	7	94	0.4%	7.45 [3.05; 14.74]
Khan, 1997_United Arab Emirates_Cattle	1	58	0.4%	1.72 [0.04; 9.24]
Khan, 1997_United Arab Emirates_Goat	5	42	0.4%	11.90 [3.98; 25.63]
Khan, 1997_United Arab Emirates_Sheep	6	74	0.4%	8.11 [3.03; 16.82]
Lotfollahzadeh, 2011_Iran_Cattle	52	876	0.5%	5.94 [4.46; 7.71]
Maiga, 2017_Mali_Cattle	706	1075	0.5%	65.67 [62.75; 68.51]
Mariner, 1995_Niger_Camel	48	353	0.5%	13.60 [10.20; 17.62]
Mariner, 1995_Niger_Cattle	553	1201	0.5%	46.04 [43.20; 48.91]
Mariner, 1995_Niger_Goat	11	224	0.5%	4.91 [2.48; 8.62]
Mariner, 1995_Niger_Sheep	8	271	0.5%	2.95 [1.28; 5.73]
Mariner, 1995_Niger_Small ruminants	43	418	0.5%	10.29 [7.55; 13.61]
Mertens, 2016_Bulgaria_Mixed animals	203	668	0.5%	30.39 [26.92; 34.03]
Mertens, 2016_Turkey_Mixed animals	283	497	0.5%	56.94 [52.46; 61.34]
Mohamed, 2008_Egypt_Buffalo	1	264	0.5%	0.38 [0.01; 2.09]
Mohamed, 2008_Egypt_Cattle	12	313	0.5%	3.83 [2.00; 6.60]
Mohamed, 2008_Egypt_Goat	2	175	0.4%	1.14 [0.14; 4.07]
Mohamed, 2008_Egypt_Sheep	17	270	0.5%	6.30 [3.71; 9.89]
Morrill, 1990_Egypt_Camel	600	4301	0.5%	13.95 [12.93; 15.02]
Morrill, 1990_Egypt_Cow	0	200	0.5%	0.00 [0.00; 1.83]
Morrill, 1990_Egypt_Sheep	0	400	0.5%	0.00 [0.00; 0.92]
Mostafavi, 2012_Iran_Sheep	10	270	0.5%	3.70 [1.79; 6.71]
Mostafavi, 2013_Iran_Cattle	273	1091	0.5%	25.02 [22.48; 27.70]
Mostafavi, 2013_Iran_Goat	245	987	0.5%	24.82 [22.16; 27.64]
Mostafavi, 2013_Iran_Sheep	1436	2447	0.5%	58.68 [56.70; 60.64]
Mourya, 2012_India_Buffalo	25	169	0.4%	14.79 [9.81; 21.06]
Mourya, 2012_India_Cattle	3	74	0.4%	4.05 [0.84; 11.39]
Mourya, 2012_India_Goat	26	222	0.5%	11.71 [7.79; 16.69]
Mourya, 2012_India_Pig	0	25	0.4%	0.00 [0.00; 13.72]
Mourya, 2012_India_Sheep	22	49	0.4%	44.90 [30.67; 59.77]
Mourya, 2014_India_Bovine	86	711	0.5%	12.10 [9.79; 14.72]
Mourya, 2014_India_Goat	115	279	0.5%	41.22 [35.38; 47.24]
Mourya, 2014_India_Sheep	77	236	0.5%	32.63 [26.69; 39.01]
Mourya, 2015_India_Goat	94	855	0.5%	10.99 [8.98; 13.29]
Mourya, 2015_India_Mixed animals	260	4781	0.5%	5.44 [4.81; 6.12]
Müller, 2016_Gabon_Mixed animals	101	532	0.5%	18.98 [15.74; 22.58]
Müller, 2016_Germany_Mixed animals	2	77	0.4%	2.60 [0.32; 9.07]
Müller, 2016_Ghana_Bat	1	167	0.4%	0.60 [0.02; 3.29]
Müller, 2016_Panama_Bat	0	43	0.4%	0.00 [0.00; 8.22]
Müller, 2016_Republic of the Congo_Bat	10	316	0.5%	3.16 [1.53; 5.74]
Mustafa, 2011_Afghanistan_Mixed animals	73	92	0.4%	79.35 [69.64; 87.08]
Mustafa, 2011_Afghanistan_Sheep	30	40	0.4%	75.00 [58.80; 87.31]
Nabeth, 2004_Mauritania_Goat	3	27	0.4%	11.11 [2.35; 29.16]
Nabeth, 2004_Mauritania_Sheep	18	95	0.4%	18.95 [11.63; 28.28]
Németh, 2013_Hungary_Lupus europeus	17	198	0.5%	8.59 [5.08; 13.39]
Papa, 2009_Albania_Bird unsepecified	0	42	0.4%	0.00 [0.00; 8.41]
Papa, 2009_Albania_Cattle	0	28	0.4%	0.00 [0.00; 12.34]
Papa, 2009_Albania_Goat	2	20	0.4%	10.00 [1.23; 31.70]
Papa, 2018_Greece_Goat	8	19	0.4%	42.11 [20.25; 66.50]
Qing, 2003_China_Sheep	48	119	0.4%	40.34 [31.45; 49.72]
Raileanu, 2015_Romania_Goat	16	29	0.4%	55.17 [35.69; 73.55]
Raileanu, 2015_Romania_Sheep	51	61	0.4%	83.61 [71.91; 91.85]
Rezazadeh, 2012_Iran_Sheep	26	167	0.4%	15.57 [10.43; 21.97]
Saghafipour, 2019_Iran_Cow	0	11	0.3%	0.00 [0.00; 28.49]
Saghafipour, 2019_Iran_Goat	5	26	0.4%	19.23 [6.55; 39.35]
Saghafipour, 2019_Iran_Sheep	16	99	0.4%	16.16 [9.53; 24.91]
Saidi, 1975_Iran_Mixed animals	357	1424	0.5%	25.07 [22.84; 27.41]
Saluzzo, 1985_Mauritania_Arvicanthis niloticus	7	43	0.4%	16.28 [6.81; 30.70]
Saluzzo, 1985_Mauritania_Cattle	8	25	0.4%	32.00 [14.95; 53.50]
Sas, 2017_Democratic Republic of the Congo_Cattle	2	514	0.5%	0.39 [0.05; 1.40]
Sas, 2017_Democratic Republic of the Congo_Goat	11	186	0.5%	5.91 [2.99; 10.34]
Sas, 2017_Democratic Republic of the Congo_Sheep	0	138	0.4%	0.00 [0.00; 2.64]
Sas, 2017_Mauritania_Cattle	333	495	0.5%	67.27 [62.94; 71.39]
Schuster, 2017_Greece_Cattle	32	619	0.5%	5.17 [3.56; 7.22]
Schuster, 2017_Greece_Sheep	1	81	0.4%	1.23 [0.03; 6.69]
Shepherd, 1987_South Africa, Zimbabwe_Aepyceros melampus	3	211	0.5%	1.42 [0.29; 4.10]
Shepherd, 1987_South Africa, Zimbabwe_Aethomys chrysophilus	6	61	0.4%	9.84 [3.70; 20.19]
Shepherd, 1987_South Africa, Zimbabwe_Aethomys namaquensis	0	35	0.4%	0.00 [0.00; 10.00]
Shepherd, 1987_South Africa, Zimbabwe_Canis familiaris	118	1978	0.5%	5.97 [4.96; 7.10]
Shepherd, 1987_South Africa, Zimbabwe_Crocidura species	0	23	0.4%	0.00 [0.00; 14.82]
Shepherd, 1987_South Africa, Zimbabwe_Dasymys incomtus	1	95	0.4%	1.05 [0.03; 5.73]
Shepherd, 1987_South Africa, Zimbabwe_Desmodillus auricularis	0	58	0.4%	0.00 [0.00; 6.16]
Shepherd, 1987_South Africa, Zimbabwe_Elephantulus species	0	112	0.4%	0.00 [0.00; 3.24]
Shepherd, 1987_South Africa, Zimbabwe_Equus burchelli	16	93	0.4%	17.20 [10.17; 26.43]
Shepherd, 1987_South Africa, Zimbabwe_Felis caracal	0	17	0.4%	0.00 [0.00; 19.51]
Shepherd, 1987_South Africa, Zimbabwe_Genetta genetta	0	10	0.3%	0.00 [0.00; 30.85]
Shepherd, 1987_South Africa, Zimbabwe_Lepus capensis	14	62	0.4%	22.58 [12.93; 34.97]
Shepherd, 1987_South Africa, Zimbabwe_Lepus saxatilis	19	131	0.4%	14.50 [8.96; 21.72]
Shepherd, 1987_South Africa, Zimbabwe_Lepus species	7	49	0.4%	14.29 [5.94; 27.24]
Shepherd, 1987_South Africa, Zimbabwe_Loxodonta africana	1	211	0.5%	0.47 [0.01; 2.61]
Shepherd, 1987_South Africa, Zimbabwe_Macroscelides proboscideus	0	31	0.4%	0.00 [0.00; 11.22]
Shepherd, 1987_South Africa, Zimbabwe_Mastomys coucha	0	11	0.3%	0.00 [0.00; 28.49]
Shepherd, 1987_South Africa, Zimbabwe_Mastomys natalensis	0	38	0.4%	0.00 [0.00; 9.25]
Shepherd, 1987_South Africa, Zimbabwe_Otocyon megalotis	0	10	0.3%	0.00 [0.00; 30.85]
Shepherd, 1987_South Africa, Zimbabwe_Otomys angoniensis	0	36	0.4%	0.00 [0.00; 9.74]
Shepherd, 1987_South Africa, Zimbabwe_Otomys unisulcatus	0	52	0.4%	0.00 [0.00; 6.85]
Shepherd, 1987_South Africa, Zimbabwe_Pedetes capensis	4	33	0.4%	12.12 [3.40; 28.20]
Shepherd, 1987_South Africa, Zimbabwe_Phacochoerus aethiopicus	2	40	0.4%	5.00 [0.61; 16.92]
Shepherd, 1987_South Africa, Zimbabwe_Procavia capensis	0	19	0.4%	0.00 [0.00; 17.65]
Shepherd, 1987_South Africa, Zimbabwe_Pronolagus crassicaudatus	0	13	0.3%	0.00 [0.00; 24.71]
Shepherd, 1987_South Africa, Zimbabwe_Pronolagus rupestris	0	25	0.4%	0.00 [0.00; 13.72]
Shepherd, 1987_South Africa, Zimbabwe_Rattus rattus	0	40	0.4%	0.00 [0.00; 8.81]
Shepherd, 1987_South Africa, Zimbabwe_Rhabdomys pumilio	2	344	0.5%	0.58 [0.07; 2.08]
Shepherd, 1987_South Africa, Zimbabwe_Tatera leucogaster	5	224	0.5%	2.23 [0.73; 5.13]
Shepherd, 1987_South Africa, Zimbabwe_Xerus inauris	1	37	0.4%	2.70 [0.07; 14.16]
Shepherd, 1987_South Africa_Cattle egret	0	39	0.4%	0.00 [0.00; 9.03]
Shepherd, 1987_South Africa_Laughing dove	0	14	0.4%	0.00 [0.00; 23.16]
Shepherd, 1987_South Africa_Little swift	0	15	0.4%	0.00 [0.00; 21.80]
Shepherd, 1987_South Africa_Masked weaver	0	16	0.4%	0.00 [0.00; 20.59]
Shepherd, 1987_South Africa_Moorhen	0	13	0.3%	0.00 [0.00; 24.71]
Shepherd, 1987_South Africa_Ostriches	12	92	0.4%	13.04 [6.93; 21.68]
Shepherd, 1987_South Africa_Ostriches	22	92	0.4%	23.91 [15.63; 33.94]
Shepherd, 1987_South Africa_Red bishop	0	110	0.4%	0.00 [0.00; 3.30]
Shepherd, 1987_South Africa_Redbilled quelea	0	95	0.4%	0.00 [0.00; 3.81]
Shepherd, 1987_South Africa_Sacred ibis	0	14	0.4%	0.00 [0.00; 23.16]
Shepherd, 1987_South Africa_Yellowbilled duck	0	91	0.4%	0.00 [0.00; 3.97]
Sherifi, 2016_Kosovo_Cattle	7	172	0.4%	4.07 [1.65; 8.21]
Sherifi, 2016_Kosovo_Sheep	2	95	0.4%	2.11 [0.26; 7.40]
Shrivastava, 2019_India_Bovine	23	193	0.5%	11.92 [7.71; 17.34]
Shrivastava, 2019_India_Goat	60	249	0.5%	24.10 [18.92; 29.90]
Shrivastava, 2019_India_Sheep	35	198	0.5%	17.68 [12.63; 23.72]
Suliman, 2017_Sudan_Camel	77	361	0.5%	21.33 [17.22; 25.92]
Sun, 2009_China_Livestock	403	3175	0.5%	12.69 [11.55; 13.90]
Swanepoel, 1983_South Africa_Cattle	109	170	0.4%	64.12 [56.42; 71.32]
Swanepoel, 1983_South Africa_Hare	32	210	0.5%	15.24 [10.66; 20.83]
Swanepoel, 1983_South Africa_Sheep	74	270	0.5%	27.41 [22.18; 33.14]
Swanepoel, 1985_South Africa_Cattle	1624	6128	0.5%	26.50 [25.40; 27.63]
Swanepoel, 1987_South Africa_Cattle	2460	8667	0.5%	28.38 [27.44; 29.35]
Swanepoel, 1987_Zimbabwe_Cattle	347	763	0.5%	45.48 [41.90; 49.09]
Tantawi, 1981_Iraq_Camel	23	99	0.4%	23.23 [15.33; 32.79]
Tantawi, 1981_Iraq_Cattle	122	411	0.5%	29.68 [25.31; 34.36]
Tantawi, 1981_Iraq_Goat	279	562	0.5%	49.64 [45.43; 53.86]
Tantawi, 1981_Iraq_Horse	148	252	0.5%	58.73 [52.38; 64.87]
Tantawi, 1981_Iraq_Sheep	443	769	0.5%	57.61 [54.03; 61.13]
Tantawi, 1981_Iraq_Unclassified small mammals	5	35	0.4%	14.29 [4.81; 30.26]
Telmadarraiy, 2008_Iran_Sheep	15	54	0.4%	27.78 [16.46; 41.64]
Telmadarraiy, 2010_Iran_Cow	3	10	0.3%	30.00 [6.67; 65.25]
Telmadarraiy, 2010_Iran_Sheep	18	43	0.4%	41.86 [27.01; 57.87]
Tuncer, 2014_Turkey_Cattle	26	201	0.5%	12.94 [8.63; 18.38]
Tuncer, 2014_Turkey_Goat	97	147	0.4%	65.99 [57.72; 73.59]
Tuncer, 2014_Turkey_Sheep	51	160	0.4%	31.87 [24.74; 39.70]
Umoh, 1983_Nigeria_Cattle	299	1164	0.5%	25.69 [23.20; 28.30]
Wangchuk, 2016_Bhutan_Cattle	0	92	0.4%	0.00 [0.00; 3.93]
Wangchuk, 2016_Bhutan_Goat	31	81	0.4%	38.27 [27.69; 49.74]
Williams, 2000_Oman_Camel	17	109	0.4%	15.60 [9.36; 23.79]
Williams, 2000_Oman_Cattle	1	29	0.4%	3.45 [0.09; 17.76]
Williams, 2000_Oman_Goat	61	225	0.5%	27.11 [21.42; 33.42]
Williams, 2000_Oman_Sheep	29	126	0.4%	23.02 [15.99; 31.35]
Wilson, 1990_Senegal_Sheep	98	942	0.5%	10.40 [8.53; 12.53]
Yadav, 2014_India_Cattle	14	32	0.4%	43.75 [26.36; 62.34]
Yadav, 2014_India_Goat	13	28	0.4%	46.43 [27.51; 66.13]
Yadav, 2014_India_Sheep	9	19	0.4%	47.37 [24.45; 71.14]
Zeller, 1994_Senegal_Bubalornis albirostris	0	54	0.4%	0.00 [0.00; 6.60]
Zeller, 1994_Senegal_Lamprotornis species	2	11	0.3%	18.18 [2.28; 51.78]
Zeller, 1994_Senegal_Passer luteus	0	20	0.4%	0.00 [0.00; 16.84]
Zeller, 1994_Senegal_Pterocles exustus	0	14	0.4%	0.00 [0.00; 23.16]
Zeller, 1994_Senegal_Streptopelia species	0	53	0.4%	0.00 [0.00; 6.72]
Zeller, 1994_Senegal_Tockus erythrorhynchus	6	22	0.4%	27.27 [10.73; 50.22]
**Random effect meta−analysis**		**92058**	**93.4%**	**12.06 [9.93; 14.35]**
Heterogeneity: Tau^2 = 0.0558; Chi^2 = 20184.44, df = 217 (P = 0); I^2 = 98.9% [98.9%; 99.0%]				

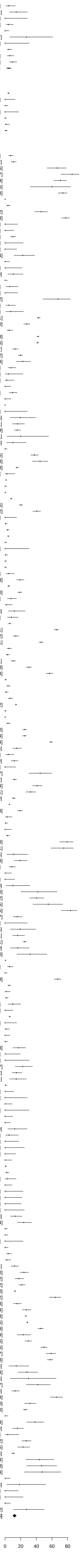

Supplement: S4 Fig — (PDF) [file pntd.0009299.s017.pdf]
