# Supplementary material for: Worldwide epidemiology of Crimean-Congo Hemorrhagic Fever Virus in humans, ticks and other animal species, a systematic review and meta-analysis
Source: PLoS Negl Trop Dis. 2021 Apr 22;15(4):e0009299. doi: 10.1371/journal.pntd.0009299 (PMC8096040; doi:10.1371/journal.pntd.0009299)

S6 Fig. Funnel plot for publication of global Crimean-congo hemorrhagic fever virus case fatality rate in humans with recent infections

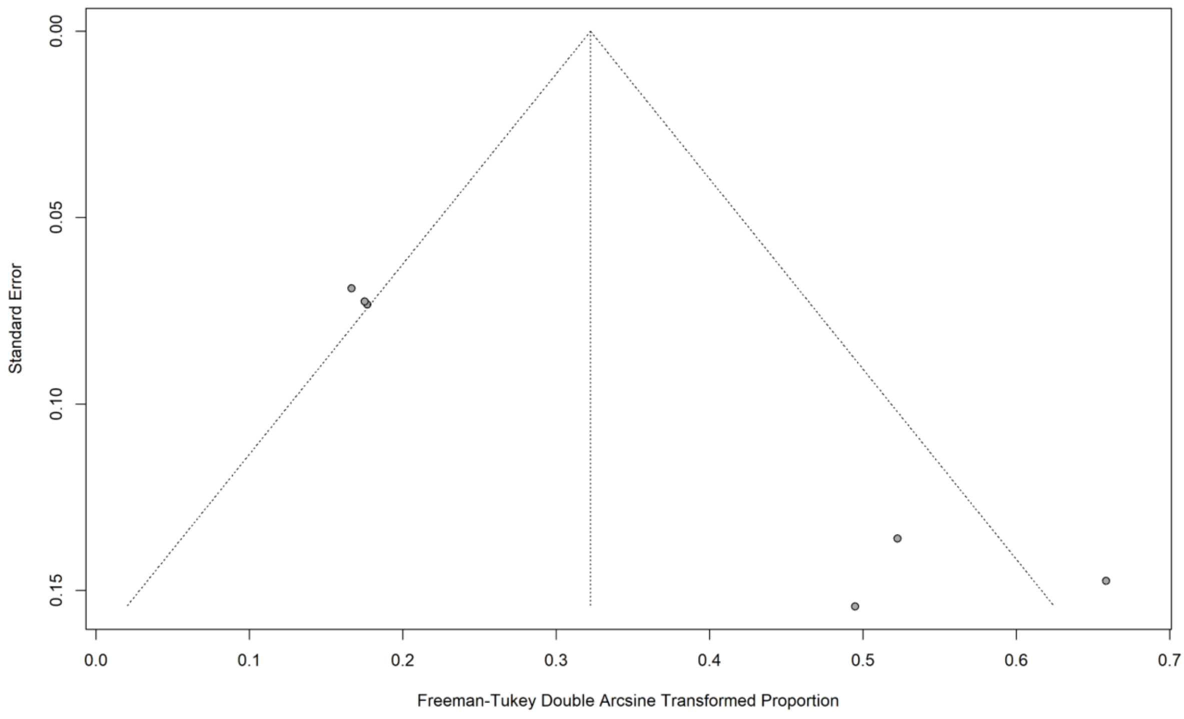

Supplement: S6 Fig — (PDF) [file pntd.0009299.s019.pdf]
