# Supplementary material for: Worldwide epidemiology of Crimean-Congo Hemorrhagic Fever Virus in humans, ticks and other animal species, a systematic review and meta-analysis
Source: PLoS Negl Trop Dis. 2021 Apr 22;15(4):e0009299. doi: 10.1371/journal.pntd.0009299 (PMC8096040; doi:10.1371/journal.pntd.0009299)

S11 Fig. Funnel plot for publication of global prevalence of Crimean-congo hemorrhagic fever virus in ticks with ongoing exposures

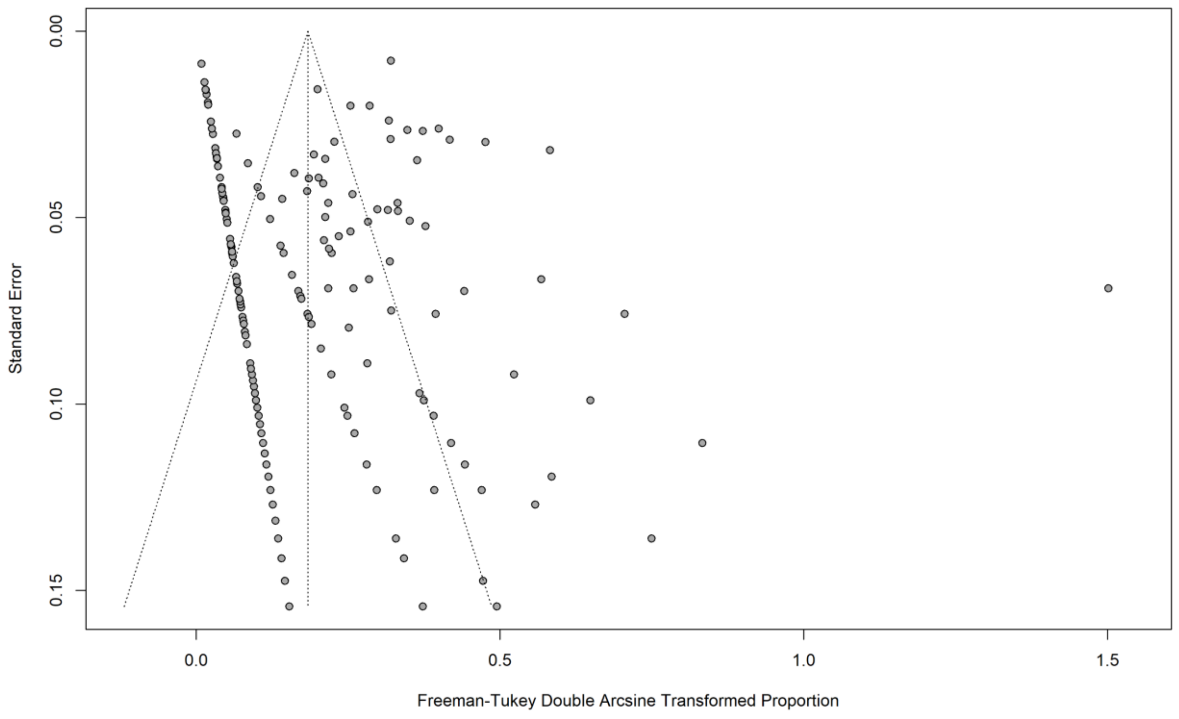

Supplement: S11 Fig — (PDF) [file pntd.0009299.s024.pdf]
