# Supplementary material for: Worldwide epidemiology of Crimean-Congo Hemorrhagic Fever Virus in humans, ticks and other animal species, a systematic review and meta-analysis
Source: PLoS Negl Trop Dis. 2021 Apr 22;15(4):e0009299. doi: 10.1371/journal.pntd.0009299 (PMC8096040; doi:10.1371/journal.pntd.0009299)
Supplement: Attachment — Submitted filename: Response to Reviewers PNTD-S-20-02469.doc [file pntd.0009299.s028.doc]

**Review Comments to the Author**

Editor

Reviewers provided a range of opinions regarding your submission. Please consider making significant revisions to your document to address these concerns. The authors are also encouraged to move some of their smaller figures and tables to the main text where they could provide additional value and clarity to the manuscript. In addition, careful copy-editing must be performed as the PLoS editorial team does not edit manuscripts.

Authors: We improved the presentation of Fig. 1 by illustrating the data for the 4 population categories studied. We removed illustrations according to the types of population because they are associated with a strong heterogeneity and a very high number of categories. These results by population type are now presented only in the supplementary table of the subgroup analysis (S8 Table). We hope in this way to have significantly improved the readability of the manuscript. Thank you. All sections have been re-read and strengthened and we hope the Reviewers will agree with the revised version.

Reviewer #1: The methodology is not always transparent (e.g. the start date of the search: Methods: No date mentioned; Discussion (line 364: “articles published from 1974”; the screening procedure [e. g. lines 135-136: “All the articles identified were first reviewed based on the title and abstract …, and afterwards they were reviewed base on the full text; lines 171-172 suggests that articles were excluded after reading titles and abstracts; “metaregression” (line 162) and “sensitivity analyses” are mentioned, but not described in sufficient detail).

Authors: Thanks for the comment, we've added the search start date.

Reviewer #2: The article by Thierry et al. describes a very thorough analysis on data from peer-reviewed papers on various aspects of CCHF/CCHFV biology. It is extremely well written and the results improve the fields clarity on these subjects. I have only minor suggestions:

Authors: Thank you for appreciation and summary.

1 Line 57: It s incorrect to state that CCFV circulates worldwide. As far as I can tell, and judging by the maps presented, it is not in the New World. Please re-word.

Authors: Thank you for the comment. We modified the sentence.

2. Line 91: Same as above

Authors: Thank you for the comment. We modified the sentence.

In assessment of study quality, please specify the total number of papers for each section (for example see line 182). Add the n size for papers looking at individual ticks, pooled ticks and other animals

Authors: Thanks for the comment, the text has been corrected as indicated.

3 Can the authors add a table listing "other" animals studied to date and a part of this analysis

Authors: The S6 Table already includes this sub table. This is a very large and important table. We could be moved back the table in the main manuscript if the Editor allows it. However, we have added the sentence below in the results to draw attention to it.

Animals of other species studied to date belong to more than 20 orders (more than 80 species).

Reviewer #3: The manucript is a systematic review and meta-analysis on Crimean-Congo hemorrhagic fever virus (CCHFV), with emphasis on global epidemiology. The search and evaluation methods are valid and well-explained.

Authors: Thank you for appreciation and summary.

However, the manuscript definitely requires major edits in form, that are explained below:

- The claims of originality indicated in several statements is unnecessary and do not reflect the content. The authors seem to emphasize case fatality rate for this purpose in many instances in the text, summary, keywords etc., to set this effort apart from other recent reviews on CCHFV (please consider how contradictory the statement in the the very first lines of the introduction (line 55) appears in this regard). Comparison of prevalence rates in previous meta-analyses (as observed in Discussion) is also unnecassary and can be due to differences in methodology.

Authors: Thanks for these comments. We corrected as requested.

- In the text, many statements suffer from awkward phrasing with occasional grammatical errors. A language review by a native speaker is required.

Authors: Thanks for this comment. All sections have been re-read and strengthened and we hope the Reviewer will agree with the revised version.

- Table 1 is hard to follow and is unlikely to fit in a print format.

Authors: Thanks for this comment. We significantly reduced Table 1 by removing the data by population category. This removed information is currently present in the S8 Table.

- Although obviously cited in this format, the references are practically too many. This reviewer wonders whether it is possible to transfer the references to a supplement format. The citations in the text (such as in lines 244-245, 250-251) are impossible to follow.

Authors: Thanks for this suggestion. The manuscript references were submitted as additional texts according to the 4 categories of review estimates (case fatality rate in humans and prevalence in humans, ticks and animals of other species).

- Figures 2 and 3 are hard to follow, maybe due to the text format in the document without titles. The figures except for Fig.1 are practically tables and can be organized as such.

- The format in Fig.6 also prevents understanding of the color-based differences in prevalence etc.

Authors: Thanks for this comment. We significantly modified all figures and supplementary figures.

- There are also occational typing errors (Line 148: “Hoy et al.”; line 162: “meta-regression”), which are minor considering the issues outlined above.

Authors: Thank you for the comment. We modified the sentence.

Reviewer #3: Results are relatively hard to follow due to the problems outlined above.

We removed illustrations according to the types of population because they are associated with a strong heterogeneity and a very high number of categories. We hope in this way to have significantly improved the readability of the manuscript.
