# Supplementary material for: Worldwide epidemiology of Crimean-Congo Hemorrhagic Fever Virus in humans, ticks and other animal species, a systematic review and meta-analysis
Source: PLoS Negl Trop Dis. 2021 Apr 22;15(4):e0009299. doi: 10.1371/journal.pntd.0009299 (PMC8096040; doi:10.1371/journal.pntd.0009299)
Supplement: Attachment — Submitted filename: Response to Reviewers PNTD-S-20-02469.doc [file pntd.0009299.s029.doc]

**Review Comments to the Author**

Editor

Reviewer #2: The authors have adequately addressed my suggestions.

Authors: Thank you

Reviewer #4: Overall, this is a very clearly written and thoughtful review/meta-analysis which creates further knowledge about global variations in CCHFV prevalence and seroprevalence in multiple hosts/vectors.

Authors: Thank you for appreciation and summary.

Reviewer #4: This systematic review of CCHFV prevalence/seroprevalence sets itself apart from others by focussing on four categories: wildlife, livestock, ticks, and humans. The study design follows PRISMA guidelines and has been used in other published studies, with inclusion criteria clearly laid out. The quantitative (and some qualitative) methodologies are appropriate for this type of meta-analysis, with due consideration of potential sources of bias.

Authors: Thank you for appreciation.

Minor comment: I understand the need to filter out articles written in any language other than English or French for translation purposes, but it would be useful to know if this constituted a large or small number of articles. How many were in other languages?

Authors: Thanks for the comment, we added the sentence below in the limitation section of the discussion.

“Another pitfall of this work is the consideration for inclusion of only article written in English or French. A search for studies in the language other than English and French in the 3 databases consulted resulted in about 200 studies to be screened for titles, abstracts and full texts for potential inclusion.”

Reviewer #4: 312 studies ultimately contributed to 802 sources of data in the four categories of interest (wildlife, livestock, ticks, humans). Table 1 is well-presented and shows the summary of meta-analysis results. It may be helpful to have an additional table before this one which summarizes the baseline characteristics, which are currently only laid out in the text. Some of the maps in Figure 4 are not given detailed enough titles. This could be helped with labels for each map (a, b, c, etc.), alongside a more details caption for the figure which lays out exactly what each map shows.

Authors: Tables S6 and S7 support all the details presented in the text for the description of the included studies. Table S6, which presents the summary of the characteristics of the included studies, would have been ideal for insertion in the main manuscript, it is however very large and could thus alter the readability of the manuscript. We have added the letters (a to j) in Fig 4 as suggested and modified the caption accordingly, thank you.

Reviewer #4: The conclusions are well supported by the results presented, with limitations and public health implications explicitly addressed, at least in a general sense. It would be helpful to have specific public health implications laid out by world region, according to the geographic variation seen in the maps in Figure 4.

Authors: Thank you for this suggestion, we have added the sentence below as an additional implication of the results of this review.

“This study shows that the majority of studies on the CCHFV CFR and associated factors was conducted in Turkey followed by Iran, Afghanistan, Pakistan, India. It is therefore urgent to conduct more studies to estimate the CCHFV CFR and the associated factors in other regions and more particularly in low-resource settings where the burden is greater.”

The authors note that caution should be given to the finding that high prevalence of CCHFV were not noted in low-income countries due to poor access to diagnostic resources. Are there any further analyses that may be done that might be able to model/quantify this discrepancy and perhaps estimate the degree of underreporting based on diagnostic capacity? A discussion around this point would be helpful and perhaps guide future studies.

Authors: We fully agree with the reviewer that such an analysis would strengthen the data in this review. Unfortunately, we do not have in the included articles the data to collect to conduct this analysis. Thank you for the suggestion.

Reviewer #4: Minor comment: no need to capitalise “Domestic and Wild Animals” (line 87)

Authors: revised, thank you.

Reviewer #5: The authors have submitted a comprehensive meta analysis and systematic review of CCHF literature on the epidemiology of CCHFV in human, tick and other animals. The methodology is clearly described but there are some areas that the authors may want to provide clarification for the reader.

1.perhaps the authors could consider replacing "ongoing" with "acute". Acute infections is more commonly used to describe CCHFV infections detected using virus, antigen RNA etc

Authors: “Ongoing” was modified to “acute” throughout the manuscript, thank you.

2.line 244, perhaps define what is included in sero-prevalence, eg did you take data from articles describing IgG, IgM and/or both IgG and IgM?

Authors: We added the definition below in the methodology of the manuscript, thank you.

“We defined acute infection rates as "prevalence" and recent and past infection rates as "seroprevalence".”

3. In line 218 the authors indicate that studies mostly included suspected cases and febrile patients, based on this comment, do the participant numbers described in the results include suspected and confirmed cases, for example the overall sero-prevalence in 74 900 participants with past infections was 4.3%? perhaps this could be clarified for all the results provided for humans and other animals.

Authors: The included study population categories were diverse and heterogeneous according to the definitions of the authors of the included studies. All the prevalences according to these population categories are presented in Supplementary Table 8 and the main results of this analysis are presented in the subgroup analysis of the results section. Initially, the integration of these results into the main manuscript in Table 1 was very extensive, which is why now these results are shown as supplementary data. Thank you.

4. line 246, is this seroprevalence you refer to?

Authors: revised, thank you.

5. The analysis of tick data showed prevalence was mostly reported in Hyalomma and Rhipicephalus however in line 325 the authors refer to results from pooled prevalence and comment that the highest prevalence was recorded in the genera Ornithodoros and Amblyomma. this was also highlighted in the abstract and author summary. although CCHFV has been isolated from more than 30 species, the role of tick species other than Hyalomma is not fully understood and does need confirmation. these results need to be discussed further (line 395) and clarified in the context of the current knowledge of known vector species to prevent misinterpretation.

Authors: We added to the results the number of tick species in which CCHFV was detected. We also clarified in the discussion and abstract the uncertainty about the role played by non-Hyalomma species in the epidemiology of CCHFV. Thank you.

Overall the authors have submitted a comprehensive and useful review that will be strengthened by additional discussion in the context of current knowledge on CCHF.

Reviewer #5: the methodology is adequately described

Reviewer #5: The results are comprehensively presented

Reviewer #5: The authors need to provide further discussion to clarify some of their findings. (see general comments below)

Reviewer #5: The use of English could be improved.

Authors: Thanks for the appreciation. All sections were improved and the manuscript edited by a native English speaker for language correction. Thank you.
